# Supplementary material for: The spatiotemporal characteristics of the air pollutants in China from 2015 to 2019
Source: PLoS One. 2020 Aug 21;15(8):e0227469. doi: 10.1371/journal.pone.0227469 (PMC7444542; doi:10.1371/journal.pone.0227469)
Supplement: S1 File — (DOCX) [file pone.0227469.s001.docx]

The spatiotemporal characteristics of the air pollutants in China from 2015 to 2019

Guo Peng 1¶* Umarova Aminat Batalbievna 1＆ Luau Yunqi 1＆

1 Department of Soil, Division of Soil of Physics, Moscow State University, Moscow, Russian Federation

*Email: [956095891@qq.com](mailto:956095891@qq.com)

**Supporting information**

**S1 Table. Statistical analysis of the PM_2.5_ (unit: μg/m^3^) in China in 2015.**

| PM_2.5_ in 2015 | Total cities | Mean | Standard Deviation | Lower 95% CI of Mean | Upper 95% CI of Mean | Min | Median | Max | P90 | P95 | P99 | 25 < Number of cities < 35 |
| --- | --- | --- | --- | --- | --- | --- | --- | --- | --- | --- | --- | --- |
| N | 71.0 | 69.4 | 18.9 | 64.9 | 73.8 | 33.7 | 71.8 | 105.9 | 92.9 | 98.5 | 105.9 | 3 |
| X | 16.0 | 53.4 | 27.9 | 38.5 | 68.2 | 12.0 | 48.6 | 118.2 | 98.4 | 118.2 | 118.2 | 2 |
| E | 48.0 | 48.9 | 13.1 | 45.1 | 52.7 | 25.5 | 52.2 | 90.0 | 60.3 | 63.4 | 90.0 | 10 |
| S | 37.0 | 35.0 | 6.6 | 32.8 | 37.2 | 17.0 | 34.3 | 48.9 | 42.9 | 48.0 | 48.9 | 17 |
| SC | 22.0 | 46.5 | 15.8 | 39.5 | 53.5 | 18.9 | 51.5 | 73.1 | 61.0 | 61.3 | 73.1 | 2 |
| NE | 38.0 | 48.8 | 11.9 | 44.9 | 52.7 | 24.6 | 49.8 | 70.8 | 63.8 | 68.4 | 70.8 | 4 |
| W | 29.0 | 45.8 | 8.2 | 42.7 | 48.9 | 31.3 | 46.6 | 59.8 | 58.5 | 59.0 | 59.8 | 2 |
| YG | 24.0 | 29.0 | 7.2 | 25.9 | 32.0 | 15.8 | 29.4 | 41.7 | 37.9 | 41.5 | 41.7 | 10 |
| C | 55.0 | 53.7 | 10.1 | 51.0 | 56.4 | 33.5 | 52.9 | 72.7 | 69.0 | 70.0 | 72.7 | 2 |
| Q | 11.0 | 40.0 | 13.6 | 30.8 | 49.1 | 18.7 | 42.9 | 57.4 | 54.1 | 57.4 | 57.4 | 0 |
| M | 11.0 | 39.3 | 10.4 | 32.4 | 46.3 | 17.4 | 40.7 | 54.5 | 50.0 | 54.5 | 54.5 | 1 |
| CH | 362.0 | 50.1 | 18.1 | 48.3 | 52.0 | 12.0 | 49.2 | 118.2 | 73.2 | 86.1 | 100.6 | 53 |

**S2 Table. Statistical analysis of the PM_2.5_ (unit: μg/m^3^) in China in 2016.**

| PM_2.5_ in 2016 | Total cities | Mean | Standard Deviation | Lower 95% CI of Mean | Upper 95% CI of Mean | Min | Median | Max | P90 | P95 | P99 |
| --- | --- | --- | --- | --- | --- | --- | --- | --- | --- | --- | --- |
| N | 71.0 | 63.8 | 16.0 | 60.0 | 67.6 | 30.0 | 67.2 | 94.1 | 84.4 | 86.5 | 94.1 |
| X | 16.0 | 60.6 | 37.3 | 40.7 | 80.5 | 10.4 | 54.6 | 156.8 | 107.2 | 156.8 | 156.8 |
| E | 48.0 | 42.1 | 10.1 | 39.1 | 45.0 | 22.1 | 43.9 | 61.7 | 54.8 | 59.8 | 61.7 |
| S | 37.0 | 32.3 | 6.4 | 30.2 | 34.4 | 13.3 | 33.0 | 44.5 | 40.5 | 43.8 | 44.5 |
| SC | 22.0 | 46.2 | 14.5 | 39.8 | 52.6 | 18.8 | 50.6 | 73.1 | 59.8 | 64.1 | 73.1 |
| NE | 38.0 | 39.9 | 9.1 | 36.9 | 42.9 | 16.6 | 41.0 | 54.7 | 50.4 | 52.9 | 54.7 |
| W | 29.0 | 45.2 | 12.8 | 40.4 | 50.1 | 30.3 | 41.6 | 80.8 | 71.1 | 74.2 | 80.8 |
| YG | 24.0 | 28.0 | 7.3 | 24.9 | 31.1 | 15.1 | 26.9 | 43.7 | 39.0 | 43.4 | 43.7 |
| C | 55.0 | 49.5 | 7.6 | 47.4 | 51.5 | 27.7 | 50.2 | 64.0 | 59.8 | 61.6 | 64.0 |
| Q | 11.0 | 35.0 | 12.2 | 26.8 | 43.2 | 17.1 | 31.7 | 53.4 | 49.6 | 53.4 | 53.4 |
| M | 11.0 | 34.1 | 9.5 | 27.7 | 40.5 | 15.2 | 32.5 | 46.4 | 46.2 | 46.4 | 46.4 |
| CH | 362.0 | 46.2 | 17.5 | 44.4 | 48.0 | 10.4 | 44.2 | 156.8 | 71.1 | 76.6 | 92.0 |

**S3 Table. Statistical analysis of the PM_2.5_ (unit: μg/m^3^) in China in 2017.**

| PM_2.5_ in 2017 | Total cities | Mean | Standard Deviation | Lower 95% CI of Mean | Upper 95% CI of Mean | Min | Median | Max | P90 | P95 | P99 |
| --- | --- | --- | --- | --- | --- | --- | --- | --- | --- | --- | --- |
| N | 71.0 | 58.4 | 15.1 | 54.8 | 62.0 | 24.7 | 61.8 | 85.6 | 74.9 | 81.9 | 85.6 |
| X | 16.0 | 53.5 | 25.4 | 40.0 | 67.0 | 13.0 | 50.8 | 99.7 | 91.8 | 99.7 | 99.7 |
| E | 48.0 | 40.4 | 10.1 | 37.5 | 43.4 | 22.5 | 41.3 | 67.9 | 55.2 | 57.0 | 67.9 |
| S | 37.0 | 33.9 | 6.6 | 31.7 | 36.1 | 14.9 | 33.9 | 48.0 | 42.0 | 44.3 | 48.0 |
| SC | 22.0 | 41.4 | 14.1 | 35.1 | 47.7 | 10.0 | 46.1 | 67.9 | 52.9 | 56.6 | 67.9 |
| NE | 38.0 | 39.4 | 8.4 | 36.6 | 42.1 | 17.7 | 39.7 | 57.1 | 49.1 | 49.9 | 57.1 |
| W | 29.0 | 42.6 | 13.3 | 37.5 | 47.6 | 22.9 | 38.4 | 79.0 | 69.0 | 72.9 | 79.0 |
| YG | 24.0 | 26.1 | 6.6 | 23.3 | 28.9 | 10.6 | 26.5 | 39.3 | 32.8 | 33.4 | 39.3 |
| C | 55.0 | 50.0 | 9.0 | 47.5 | 52.4 | 25.9 | 49.5 | 70.4 | 62.4 | 67.5 | 70.4 |
| Q | 11.0 | 28.3 | 9.5 | 21.9 | 34.7 | 13.6 | 27.3 | 47.0 | 38.3 | 47.0 | 47.0 |
| M | 11.0 | 31.4 | 10.7 | 24.2 | 38.6 | 14.8 | 34.2 | 46.1 | 44.1 | 46.1 | 46.1 |
| CH | 362.0 | 43.8 | 15.5 | 42.2 | 45.4 | 10.0 | 42.0 | 99.7 | 66.2 | 70.4 | 83.7 |

**S4 Table. Statistical analysis of the PM_2.5_ (unit: μg/m^3^) in China in 2018.**

| PM_2.5_ in 2018 | Total cities | Mean | Standard Deviation | Lower 95% CI of Mean | Upper 95% CI of Mean | Min | Median | Max | P90 | P95 | P99 |
| --- | --- | --- | --- | --- | --- | --- | --- | --- | --- | --- | --- |
| N | 71.0 | 49.6 | 12.1 | 46.7 | 52.5 | 22.3 | 52.5 | 68.8 | 61.3 | 66.3 | 68.8 |
| X | 16.0 | 49.9 | 28.8 | 34.6 | 65.3 | 8.8 | 49.8 | 117.5 | 94.5 | 117.5 | 117.5 |
| E | 48.0 | 36.2 | 9.5 | 33.5 | 39.0 | 20.5 | 36.0 | 62.1 | 49.9 | 51.4 | 62.1 |
| S | 37.0 | 29.5 | 5.4 | 27.7 | 31.3 | 14.2 | 29.2 | 37.9 | 36.5 | 36.8 | 37.9 |
| SC | 22.0 | 34.4 | 9.7 | 30.1 | 38.8 | 11.4 | 34.4 | 52.3 | 44.5 | 47.1 | 52.3 |
| NE | 38.0 | 31.6 | 7.1 | 29.3 | 33.9 | 16.3 | 32.0 | 45.7 | 39.1 | 42.3 | 45.7 |
| W | 29.0 | 38.4 | 8.3 | 35.3 | 41.6 | 25.0 | 36.7 | 61.2 | 53.5 | 56.7 | 61.2 |
| YG | 24.0 | 24.0 | 5.4 | 21.7 | 26.2 | 11.7 | 24.4 | 33.9 | 30.5 | 30.7 | 33.9 |
| C | 55.0 | 40.6 | 8.2 | 38.4 | 42.8 | 22.7 | 41.4 | 57.3 | 52.9 | 55.6 | 57.3 |
| Q | 11.0 | 25.3 | 9.6 | 18.9 | 31.7 | 13.8 | 24.4 | 42.9 | 40.4 | 42.9 | 42.9 |
| M | 11.0 | 29.2 | 9.0 | 23.1 | 35.3 | 15.5 | 30.9 | 40.7 | 39.0 | 40.7 | 40.7 |
| CH | 362.0 | 37.7 | 13.3 | 36.3 | 39.0 | 8.8 | 35.9 | 117.5 | 56.0 | 59.7 | 68.8 |

**S5 Table. Statistical analysis of the PM_2.5_ (unit: μg/m^3^) in China in 2019.**

| PM_2.5_ in 2019 | Total cities | Mean | S. D. | Lower 95% CI of Mean | Upper 95% CI of Mean | Min | Median | Max | P90 | P95 | P99 | 25 < Number of cities at < 35 | Annual mean from 2015 to 2019 |
| --- | --- | --- | --- | --- | --- | --- | --- | --- | --- | --- | --- | --- | --- |
| N | 71.0 | 49.8 | 13.4 | 46.6 | 53.0 | 23.3 | 52.0 | 106.2 | 59.4 | 63.1 | 106.2 | 6.0 | 58.2 |
| X | 16.0 | 45.0 | 25.7 | 31.3 | 58.6 | 9.4 | 46.7 | 107.5 | 84.8 | 107.5 | 107.5 | 1.0 | 52.5 |
| E | 48.0 | 33.3 | 7.7 | 31.1 | 35.6 | 18.4 | 34.2 | 55.2 | 42.3 | 43.6 | 55.2 | 16.0 | 40.2 |
| S | 37.0 | 26.1 | 4.1 | 24.7 | 27.5 | 13.3 | 27.2 | 33.0 | 31.1 | 32.4 | 33.0 | 24.0 | 31.4 |
| SC | 22.0 | 32.1 | 8.7 | 28.2 | 35.9 | 10.9 | 32.9 | 45.5 | 41.7 | 42.2 | 45.5 | 10.0 | 40.1 |
| NE | 38.0 | 32.1 | 8.3 | 29.4 | 34.9 | 13.4 | 33.1 | 53.2 | 41.1 | 45.0 | 53.2 | 18.0 | 38.4 |
| W | 29.0 | 33.4 | 10.0 | 29.6 | 37.2 | 17.6 | 31.3 | 57.2 | 52.6 | 54.4 | 57.2 | 15.0 | 41.1 |
| YG | 24.0 | 22.3 | 5.0 | 20.1 | 24.4 | 10.3 | 23.0 | 31.5 | 28.7 | 31.5 | 31.5 | 5.0 | 25.9 |
| C | 55.0 | 39.2 | 7.9 | 37.0 | 41.3 | 23.1 | 40.0 | 57.2 | 49.5 | 52.8 | 57.2 | 18.0 | 46.6 |
| Q | 11.0 | 19.4 | 9.2 | 13.3 | 25.6 | 6.0 | 19.1 | 37.0 | 31.9 | 37.0 | 37.0 | 1.0 | 29.6 |
| M | 11.0 | 25.1 | 7.4 | 20.2 | 30.1 | 10.2 | 24.4 | 34.8 | 34.0 | 34.8 | 34.8 | 5.0 | 31.8 |
| CH | 362.0 | 35.6 | 13.7 | 34.2 | 37.0 | 6.0 | 33.5 | 107.5 | 53.8 | 57.0 | 84.8 | 119.0 | 42.7 |

**S6 Table. Absolute change of PM_2.5_ between 2015 and 2019.**

| Absolute change of PM_2.5_ between 2015 and 2019 | Total cities | Mean | Standard Deviation | Lower 95% CI of Mean | Upper 95% CI of Mean | Min | Median | Max | P90 | P95 | P99 |
| --- | --- | --- | --- | --- | --- | --- | --- | --- | --- | --- | --- |
| N | 71.0 | -19.6 | 13.9 | -22.9 | -16.3 | -49.5 | -17.5 | 19.5 | -6.9 | -0.9 | 19.5 |
| X | 16.0 | -8.4 | 11.3 | -14.4 | -2.4 | -33.3 | -6.4 | 9.1 | 7.0 | 9.1 | 9.1 |
| E | 48.0 | -15.6 | 8.6 | -18.1 | -13.1 | -56.5 | -16.7 | -2.9 | -5.3 | -4.1 | -2.9 |
| S | 37.0 | -8.9 | 3.5 | -10.1 | -7.7 | -16.8 | -8.2 | -3.7 | -4.1 | -3.9 | -3.7 |
| SC | 22.0 | -14.4 | 8.8 | -18.3 | -10.5 | -28.7 | -16.0 | 4.2 | -4.3 | -3.3 | 4.2 |
| NE | 38.0 | -16.7 | 8.6 | -19.5 | -13.9 | -33.9 | -17.0 | 0.6 | -5.2 | -2.3 | 0.6 |
| W | 29.0 | -12.3 | 5.3 | -14.3 | -10.3 | -24.4 | -13.4 | -1.8 | -4.0 | -3.0 | -1.8 |
| YG | 24.0 | -6.7 | 7.1 | -9.7 | -3.7 | -23.4 | -5.7 | 3.0 | 1.8 | 2.5 | 3.0 |
| C | 55.0 | -14.5 | 7.7 | -16.6 | -12.4 | -30.0 | -15.5 | 7.4 | -4.8 | -1.0 | 7.4 |
| Q | 11.0 | -20.5 | 7.4 | -25.5 | -15.6 | -35.0 | -20.4 | -10.2 | -12.7 | -10.2 | -10.2 |
| M | 11.0 | -14.2 | 6.0 | -18.2 | -10.2 | -23.6 | -15.1 | -4.3 | -7.2 | -4.3 | -4.3 |
| CH | 362.0 | -14.5 | 9.9 | -15.5 | -13.5 | -56.5 | -13.9 | 19.5 | -3.9 | -1.5 | 7.4 |

**S7 Table. Relative change of PM_2.5_ between 2015 and 2019.**

| Relative change of PM_2.5_ between 2015 and 2019 | Total cities | Mean | S. D. | Lower 95% CI of Mean | Upper 95% CI of Mean | Min | Median | Max | P90 | P95 | P99 |
| --- | --- | --- | --- | --- | --- | --- | --- | --- | --- | --- | --- |
| N | 71.0 | -26.6 | 14.3 | -30.0 | -23.2 | -49.1 | -28.3 | 26.1 | -11.7 | -1.8 | 26.1 |
| X | 16.0 | -17.5 | 17.7 | -26.9 | -8.1 | -54.0 | -21.6 | 14.1 | 9.2 | 14.1 | 14.1 |
| E | 48.0 | -30.3 | 10.1 | -33.2 | -27.3 | -62.7 | -32.8 | -9.8 | -15.5 | -12.5 | -9.8 |
| S | 37.0 | -24.8 | 6.4 | -27.0 | -22.7 | -39.8 | -24.0 | -11.8 | -17.6 | -14.0 | -11.8 |
| SC | 22.0 | -28.3 | 14.4 | -34.7 | -21.9 | -46.8 | -31.1 | 19.7 | -15.5 | -10.7 | 19.7 |
| NE | 38.0 | -33.0 | 13.9 | -37.5 | -28.4 | -57.8 | -34.5 | 2.4 | -12.9 | -6.3 | 2.4 |
| W | 29.0 | -27.7 | 12.0 | -32.3 | -23.1 | -50.1 | -29.1 | -3.0 | -10.2 | -5.2 | -3.0 |
| YG | 24.0 | -20.6 | 19.3 | -28.7 | -12.4 | -56.3 | -23.4 | 11.8 | 6.0 | 10.5 | 11.8 |
| C | 55.0 | -26.2 | 12.9 | -29.7 | -22.8 | -45.2 | -27.6 | 21.8 | -9.3 | -2.0 | 21.8 |
| Q | 11.0 | -52.7 | 10.3 | -59.7 | -45.8 | -67.8 | -55.4 | -34.1 | -35.6 | -34.1 | -34.1 |
| M | 11.0 | -35.8 | 11.3 | -43.4 | -28.2 | -52.3 | -39.9 | -15.7 | -19.6 | -15.7 | -15.7 |
| CH | 363.0 | -27.9 | 14.4 | -29.3 | -26.4 | -67.8 | -29.1 | 26.1 | -10.9 | -3.0 | 21.6 |

**S8 Table. Annual average absolute change of PM_2.5_ from 2015 to 2019.**

| Annual average absolute change of PM_2.5_ | Total cities | Mean | Standard Deviation | Lower 95% CI of Mean | Upper 95% CI of Mean | Min | Median | Max | P90 | P95 | P99 | Number of cities at < 0 μg/m^3^ | Number of cities at > 0 μg/m^3^ |
| --- | --- | --- | --- | --- | --- | --- | --- | --- | --- | --- | --- | --- | --- |
| N | 71.0 | -4.9 | 3.5 | -5.7 | -4.1 | -12.4 | -4.4 | 4.9 | -1.7 | -0.2 | 4.9 | 68.0 | 3.0 |
| X | 16.0 | -2.1 | 2.8 | -3.6 | -0.6 | -8.3 | -1.6 | 2.3 | 1.7 | 2.3 | 2.3 | 14.0 | 2.0 |
| E | 48.0 | -3.9 | 2.2 | -4.5 | -3.3 | -14.1 | -4.2 | -0.7 | -1.3 | -1.0 | -0.7 | 48.0 | 0.0 |
| S | 37.0 | -2.2 | 0.9 | -2.5 | -1.9 | -4.2 | -2.1 | -0.9 | -1.0 | -1.0 | -0.9 | 37.0 | 0.0 |
| SC | 22.0 | -3.6 | 2.2 | -4.6 | -2.6 | -7.2 | -4.0 | 1.0 | -1.1 | -0.8 | 1.0 | 21.0 | 1.0 |
| NE | 38.0 | -4.2 | 2.2 | -4.9 | -3.5 | -8.5 | -4.2 | 0.2 | -1.3 | -0.6 | 0.2 | 37.0 | 1.0 |
| W | 29.0 | -3.1 | 1.3 | -3.6 | -2.6 | -6.1 | -3.4 | -0.4 | -1.0 | -0.7 | -0.4 | 29.0 | 0.0 |
| YG | 24.0 | -1.7 | 1.8 | -2.4 | -0.9 | -5.8 | -1.4 | 0.7 | 0.4 | 0.6 | 0.7 | 20.0 | 4.0 |
| C | 55.0 | -3.6 | 1.9 | -4.2 | -3.1 | -7.5 | -3.9 | 1.8 | -1.2 | -0.2 | 1.8 | 54.0 | 1.0 |
| Q | 11.0 | -5.1 | 1.8 | -6.4 | -3.9 | -8.7 | -5.1 | -2.6 | -3.2 | -2.6 | -2.6 | 11.0 | 0.0 |
| M | 11.0 | -3.5 | 1.5 | -4.6 | -2.5 | -5.9 | -3.8 | -1.1 | -1.8 | -1.1 | -1.1 | 11.0 | 0.0 |
| CH | 362.0 | -3.6 | 2.5 | -3.9 | -3.4 | -14.1 | -3.5 | 4.9 | -1.0 | -0.4 | 1.8 | 350.0 | 12.0 |

**S9 Table. Annual average relative change of PM_2.5_ from 2015 to 2019.**

| Annual average relative change of PM_2.5_ | Total cities | Mean | Standard Deviation | Lower 95% CI of Mean | Upper 95% CI of Mean | Min | Median | Max | P90 | P95 | P99 |
| --- | --- | --- | --- | --- | --- | --- | --- | --- | --- | --- | --- |
| N | 71.0 | -7.0 | 5.1 | -8.2 | -5.8 | -15.3 | -7.3 | 11.1 | -2.0 | 2.0 | 11.1 |
| X | 16.0 | -3.0 | 6.3 | -6.3 | 0.4 | -17.2 | -4.1 | 6.7 | 4.8 | 6.7 | 6.7 |
| E | 48.0 | -8.4 | 3.1 | -9.3 | -7.5 | -15.8 | -8.8 | -0.8 | -4.0 | -3.0 | -0.8 |
| S | 37.0 | -6.5 | 2.1 | -7.2 | -5.8 | -11.4 | -6.4 | -2.5 | -4.3 | -3.3 | -2.5 |
| SC | 22.0 | -7.5 | 4.5 | -9.5 | -5.4 | -14.0 | -8.4 | 5.5 | -2.3 | -1.3 | 5.5 |
| NE | 38.0 | -8.7 | 5.2 | -10.4 | -7.0 | -18.8 | -8.4 | 5.1 | -2.5 | 4.3 | 5.1 |
| W | 29.0 | -7.3 | 4.0 | -8.9 | -5.8 | -14.4 | -7.7 | 1.4 | -1.1 | 0.0 | 1.4 |
| YG | 24.0 | -4.9 | 5.9 | -7.5 | -2.4 | -17.7 | -5.0 | 3.3 | 3.1 | 3.1 | 3.3 |
| C | 55.0 | -6.8 | 4.3 | -8.0 | -5.7 | -13.6 | -7.7 | 8.5 | -1.6 | 0.8 | 8.5 |
| Q | 11.0 | -16.3 | 3.9 | -18.9 | -13.7 | -21.7 | -16.8 | -9.0 | -10.1 | -9.0 | -9.0 |
| M | 11.0 | -9.9 | 3.7 | -12.4 | -7.5 | -15.4 | -10.0 | -3.7 | -4.9 | -3.7 | -3.7 |
| CH | 362.0 | -7.4 | 4.9 | -7.9 | -6.9 | -21.7 | -7.7 | 11.1 | -1.6 | 2.0 | 7.0 |

**S10 Table. Statistical analysis of the PM_10_ (unit: μg/m^3^) in China in 2015.**

| PM_10_ in 2015 | Total cities | Mean | S. D. | Lower 95% CI of Mean | Upper 95% CI of Mean | Min | Median | Max | P90 | P95 | P99 | Number of cities at < 20 | 20 < Number of cities at < 30 | Number of cities at < 70 |
| --- | --- | --- | --- | --- | --- | --- | --- | --- | --- | --- | --- | --- | --- | --- |
| N | 71.0 | 120.0 | 30.5 | 112.8 | 127.2 | 59.0 | 124.8 | 175.9 | 160.9 | 167.2 | 175.9 | 0.0 | 0.0 | 4.0 |
| X | 16.0 | 134.8 | 89.5 | 87.1 | 182.5 | 27.8 | 107.3 | 341.3 | 308.4 | 341.3 | 341.3 | 0.0 | 1.0 | 4.0 |
| E | 48.0 | 78.7 | 21.7 | 72.4 | 85.0 | 41.3 | 80.3 | 146.1 | 102.5 | 107.0 | 146.1 | 0.0 | 0.0 | 14.0 |
| S | 37.0 | 54.3 | 8.3 | 51.6 | 57.1 | 31.7 | 53.4 | 70.6 | 66.4 | 70.5 | 70.6 | 0.0 | 0.0 | 35.0 |
| SC | 22.0 | 75.3 | 19.6 | 66.6 | 84.0 | 33.2 | 81.6 | 107.2 | 91.2 | 102.6 | 107.2 | 0.0 | 0.0 | 7.0 |
| NE | 38.0 | 81.0 | 17.6 | 75.2 | 86.8 | 49.2 | 81.8 | 112.3 | 102.9 | 107.8 | 112.3 | 0.0 | 0.0 | 10.0 |
| W | 29.0 | 97.1 | 17.5 | 90.4 | 103.8 | 65.0 | 102.1 | 127.1 | 123.4 | 126.5 | 127.1 | 0.0 | 0.0 | 2.0 |
| YG | 24.0 | 50.0 | 10.7 | 45.5 | 54.5 | 32.6 | 46.3 | 71.5 | 63.1 | 67.5 | 71.5 | 0.0 | 0.0 | 23.0 |
| C | 55.0 | 83.5 | 15.7 | 79.2 | 87.7 | 46.7 | 85.3 | 115.5 | 105.6 | 108.9 | 115.5 | 0.0 | 0.0 | 11.0 |
| Q | 11.0 | 84.7 | 27.5 | 66.2 | 103.2 | 34.7 | 88.7 | 122.0 | 111.2 | 122.0 | 122.0 | 0.0 | 0.0 | 3.0 |
| M | 11.0 | 85.7 | 22.7 | 70.4 | 101.0 | 52.2 | 85.0 | 130.7 | 109.6 | 130.7 | 130.7 | 0.0 | 0.0 | 4.0 |
| CH | 362.0 | 87.5 | 35.9 | 83.8 | 91.2 | 27.8 | 84.4 | 341.3 | 130.7 | 146.6 | 186.1 | 0.0 | 1.0 | 117.0 |

**S11 Table. Statistical analysis of the PM_10_ (unit: μg/m^3^) in China in 2016.**

| PM_10_ in 2016 | Total cities | Mean | Standard Deviation | Lower 95% CI of Mean | Upper 95% CI of Mean | Min | Median | Max | P90 | P95 | P99 |
| --- | --- | --- | --- | --- | --- | --- | --- | --- | --- | --- | --- |
| N | 71.0 | 114.0 | 25.3 | 108.0 | 119.9 | 60.7 | 116.0 | 160.7 | 144.1 | 150.2 | 160.7 |
| X | 16.0 | 145.9 | 109.1 | 87.8 | 204.1 | 23.8 | 108.1 | 434.5 | 283.0 | 434.5 | 434.5 |
| E | 48.0 | 70.2 | 17.1 | 65.2 | 75.2 | 34.9 | 71.0 | 118.0 | 88.4 | 92.5 | 118.0 |
| S | 37.0 | 50.4 | 8.3 | 47.6 | 53.1 | 27.9 | 50.6 | 67.5 | 60.8 | 62.6 | 67.5 |
| SC | 22.0 | 74.4 | 18.3 | 66.3 | 82.5 | 34.2 | 77.8 | 100.8 | 90.3 | 100.6 | 100.8 |
| NE | 38.0 | 68.3 | 14.9 | 63.4 | 73.2 | 30.2 | 70.3 | 92.1 | 84.2 | 87.7 | 92.1 |
| W | 29.0 | 95.8 | 23.0 | 87.1 | 104.5 | 55.9 | 96.0 | 144.8 | 137.5 | 143.8 | 144.8 |
| YG | 24.0 | 49.0 | 11.8 | 44.0 | 54.0 | 30.2 | 46.5 | 72.2 | 70.0 | 71.0 | 72.2 |
| C | 55.0 | 78.3 | 11.0 | 75.3 | 81.3 | 46.3 | 77.8 | 100.6 | 93.0 | 99.7 | 100.6 |
| Q | 11.0 | 81.9 | 28.5 | 62.7 | 101.0 | 39.2 | 78.1 | 128.1 | 114.8 | 128.1 | 128.1 |
| M | 11.0 | 75.2 | 21.5 | 60.8 | 89.7 | 44.2 | 70.5 | 109.3 | 104.1 | 109.3 | 109.3 |
| CH | 362.0 | 82.5 | 37.8 | 78.6 | 86.5 | 23.8 | 76.6 | 434.5 | 126.2 | 143.1 | 216.9 |

**Table S12. Statistical analysis of the PM_10_ (unit: μg/m^3^) in China in 2017.**

| PM_10_ in 2017 | Total cities | Mean | Standard Deviation | Lower 95% CI of Mean | Upper 95% CI of Mean | Min | Median | Max | P90 | P95 | P99 |
| --- | --- | --- | --- | --- | --- | --- | --- | --- | --- | --- | --- |
| N | 71.0 | 108.2 | 24.8 | 102.3 | 114.1 | 52.1 | 112.7 | 156.9 | 135.7 | 141.1 | 156.9 |
| X | 16.0 | 127.3 | 76.3 | 86.7 | 168.0 | 30.0 | 112.5 | 317.8 | 247.6 | 317.8 | 317.8 |
| E | 48.0 | 68.6 | 17.9 | 63.4 | 73.9 | 36.0 | 67.9 | 124.8 | 90.9 | 95.5 | 124.8 |
| S | 37.0 | 53.2 | 8.9 | 50.2 | 56.1 | 27.8 | 54.2 | 70.6 | 65.1 | 67.1 | 70.6 |
| SC | 22.0 | 68.5 | 18.0 | 60.5 | 76.4 | 22.0 | 72.9 | 93.1 | 86.1 | 88.0 | 93.1 |
| NE | 38.0 | 68.6 | 13.9 | 64.1 | 73.2 | 33.6 | 69.1 | 86.0 | 84.7 | 85.3 | 86.0 |
| W | 29.0 | 95.9 | 22.6 | 87.3 | 104.5 | 57.5 | 94.8 | 139.4 | 131.4 | 136.1 | 139.4 |
| YG | 24.0 | 48.0 | 8.4 | 44.5 | 51.5 | 33.6 | 47.3 | 68.5 | 59.1 | 59.3 | 68.5 |
| C | 55.0 | 80.0 | 13.5 | 76.3 | 83.6 | 52.0 | 79.3 | 109.4 | 100.8 | 107.4 | 109.4 |
| Q | 11.0 | 71.0 | 32.6 | 49.2 | 92.9 | 29.1 | 61.9 | 149.8 | 97.5 | 149.8 | 149.8 |
| M | 11.0 | 74.4 | 24.5 | 57.9 | 90.8 | 42.4 | 73.7 | 112.9 | 99.5 | 112.9 | 112.9 |
| CH | 362.0 | 80.2 | 32.0 | 76.9 | 83.5 | 22.0 | 75.7 | 317.8 | 122.3 | 131.4 | 157.7 |

**S13 Table. Statistical analysis of the PM_10_ (unit: μg/m^3^) in China in 2018.**

| PM_10_ in 2018 | Total cities | Mean | Standard Deviation | Lower 95% CI of Mean | Upper 95% CI of Mean | Min | Median | Max | P90 | P95 | P99 |
| --- | --- | --- | --- | --- | --- | --- | --- | --- | --- | --- | --- |
| N | 72.0 | 100.7 | 21.2 | 95.7 | 105.6 | 46.8 | 108.1 | 135.9 | 120.1 | 130.8 | 135.9 |
| X | 16.0 | 154.0 | 116.9 | 91.7 | 216.3 | 19.2 | 111.5 | 432.2 | 331.2 | 432.2 | 432.2 |
| E | 48.0 | 64.5 | 17.2 | 59.5 | 69.5 | 33.1 | 64.3 | 108.5 | 86.0 | 94.6 | 108.5 |
| S | 37.0 | 50.4 | 8.1 | 47.7 | 53.2 | 28.4 | 50.3 | 64.8 | 61.0 | 62.0 | 64.8 |
| SC | 22.0 | 61.5 | 15.4 | 54.7 | 68.3 | 23.1 | 62.9 | 81.5 | 78.9 | 80.0 | 81.5 |
| NE | 38.0 | 59.8 | 11.7 | 56.0 | 63.6 | 34.5 | 60.5 | 79.6 | 73.7 | 77.0 | 79.6 |
| W | 29.0 | 98.0 | 22.0 | 89.7 | 106.4 | 57.3 | 100.6 | 135.3 | 129.3 | 133.7 | 135.3 |
| YG | 24.0 | 46.2 | 8.1 | 42.8 | 49.6 | 29.5 | 46.5 | 61.2 | 56.1 | 56.4 | 61.2 |
| C | 55.0 | 71.5 | 13.2 | 68.0 | 75.1 | 42.9 | 69.2 | 103.9 | 91.9 | 96.6 | 103.9 |
| Q | 11.0 | 61.4 | 21.5 | 46.9 | 75.8 | 34.3 | 56.1 | 101.1 | 98.4 | 101.1 | 101.1 |
| M | 11.0 | 79.4 | 27.1 | 61.1 | 97.6 | 31.1 | 86.9 | 119.2 | 104.0 | 119.2 | 119.2 |
| CH | 363.0 | 76.4 | 38.1 | 72.5 | 80.3 | 19.2 | 68.1 | 432.2 | 113.7 | 120.4 | 255.8 |

**S14 Table. Statistical analysis of the PM_10_ (unit: μg/m^3^) in China in 2019.**

| PM_10_ in 2019 | Total cities | Mean | S. D. | Lower 95% CI of Mean | Upper 95% CI of Mean | Min | Median | Max | P90 | P95 | P99 | Number of city < 20 | 20< Number of cities at < 30 | Number of cities at < 70 |
| --- | --- | --- | --- | --- | --- | --- | --- | --- | --- | --- | --- | --- | --- | --- |
| N | 72.0 | 93.3 | 21.6 | 88.2 | 98.4 | 50.6 | 95.1 | 167.9 | 115.7 | 123.7 | 167.9 | 0.0 | 0.0 | 10.0 |
| X | 16.0 | 121.7 | 82.9 | 77.5 | 165.9 | 16.2 | 97.7 | 334.9 | 229.4 | 334.9 | 334.9 | 1.0 | 0.0 | 5.0 |
| E | 48.0 | 58.1 | 14.4 | 53.9 | 62.3 | 29.0 | 58.2 | 99.5 | 75.9 | 79.2 | 99.5 | 0.0 | 1.0 | 38.0 |
| S | 37.0 | 43.9 | 6.1 | 41.8 | 45.9 | 26.0 | 44.4 | 54.3 | 49.8 | 52.2 | 54.3 | 0.0 | 1.0 | 37.0 |
| SC | 22.0 | 50.1 | 12.8 | 44.5 | 55.8 | 18.3 | 51.7 | 67.7 | 63.3 | 67.4 | 67.7 | 1.0 | 1.0 | 22.0 |
| NE | 38.0 | 58.8 | 13.5 | 54.4 | 63.3 | 30.5 | 59.4 | 76.9 | 76.2 | 76.6 | 76.9 | 0.0 | 0.0 | 27.0 |
| W | 29.0 | 73.4 | 16.4 | 67.1 | 79.6 | 34.5 | 72.8 | 107.6 | 99.7 | 100.0 | 107.6 | 0.0 | 0.0 | 11.0 |
| YG | 24.0 | 37.9 | 8.9 | 34.1 | 41.6 | 19.3 | 37.8 | 57.4 | 49.8 | 54.6 | 57.4 | 1.0 | 3.0 | 24.0 |
| C | 55.0 | 64.9 | 12.7 | 61.5 | 68.3 | 40.2 | 62.6 | 93.3 | 84.7 | 86.0 | 93.3 | 0.0 | 0.0 | 38.0 |
| Q | 11.0 | 43.5 | 21.4 | 29.1 | 57.9 | 11.6 | 38.0 | 89.8 | 65.1 | 89.8 | 89.8 | 1.0 | 2.0 | 10.0 |
| M | 11.0 | 60.2 | 19.2 | 47.3 | 73.1 | 34.0 | 61.4 | 93.0 | 77.5 | 93.0 | 93.0 | 0.0 | 0.0 | 7.0 |
| CH | 363.0 | 66.6 | 30.8 | 63.4 | 69.7 | 11.6 | 61.3 | 334.9 | 100.0 | 110.2 | 181.0 | 4.0 | 8.0 | 229.0 |

**S15 Table. Absolute change of PM_10_ between 2015 and 2019.**

| Absolute change of PM_10_ between 2015 and 2019 | Total cities | Mean | S. D. | Lower 95% CI of Mean | Upper 95% CI of Mean | Min | Median | Max | P90 | P95 | P99 | Number of cities at < 0 | Number of cities at > 0 |
| --- | --- | --- | --- | --- | --- | --- | --- | --- | --- | --- | --- | --- | --- |
| N | 71.0 | -27.7 | 21.1 | -32.7 | -22.8 | -80.0 | -27.9 | 29.9 | -3.1 | 8.7 | 29.9 | 66.0 | 5.0 |
| X | 16.0 | -13.1 | 25.2 | -26.5 | 0.3 | -79.0 | -9.3 | 25.8 | 18.3 | 25.8 | 25.8 | 14.0 | 2.0 |
| E | 48.0 | -20.7 | 11.1 | -23.9 | -17.4 | -64.8 | -20.8 | 1.8 | -7.1 | -6.1 | 1.8 | 48.0 | 0.0 |
| S | 37.0 | -10.5 | 4.8 | -12.1 | -8.9 | -21.1 | -9.5 | -3.6 | -5.0 | -4.3 | -3.6 | 37.0 | 0.0 |
| SC | 22.0 | -25.1 | 10.0 | -29.6 | -20.7 | -40.7 | -26.3 | -6.4 | -12.1 | -11.3 | -6.4 | 22.0 | 0.0 |
| NE | 38.0 | -22.2 | 9.8 | -25.4 | -19.0 | -39.4 | -20.5 | -3.3 | -7.5 | -6.4 | -3.3 | 38.0 | 0.0 |
| W | 29.0 | -23.7 | 9.3 | -27.3 | -20.2 | -40.0 | -22.9 | -3.9 | -12.4 | -12.1 | -3.9 | 29.0 | 0.0 |
| YG | 24.0 | -12.1 | 11.1 | -16.8 | -7.4 | -37.8 | -10.0 | 17.0 | -1.6 | -0.5 | 17.0 | 21.0 | 3.0 |
| C | 55.0 | -18.6 | 12.3 | -21.9 | -15.3 | -38.4 | -21.4 | 12.6 | -4.1 | 7.1 | 12.6 | 52.0 | 3.0 |
| Q | 11.0 | -41.2 | 20.0 | -54.7 | -27.8 | -85.6 | -38.5 | -20.6 | -21.4 | -20.6 | -20.6 | 11.0 | 0.0 |
| M | 11.0 | -25.5 | 10.0 | -32.2 | -18.8 | -37.7 | -25.7 | -3.4 | -14.6 | -3.4 | -3.4 | 11.0 | 0.0 |
| CH | 363.0 | -21.1 | 15.8 | -22.7 | -19.5 | -85.6 | -20.6 | 29.9 | -5.0 | -1.5 | 18.3 | 349.0 | 13.0 |

**S16 Table. Relative change of PM_10_ between 2015 and 2019.**

| Relative change of PM_10_ between 2015 and 2019 | Total cities | Mean | Standard Deviation | Lower 95% CI of Mean | Upper 95% CI of Mean | Min | Median | Max | P90 | P95 | P99 |
| --- | --- | --- | --- | --- | --- | --- | --- | --- | --- | --- | --- |
| N | 71.0 | -20.5 | 14.5 | -24.0 | -17.1 | -45.5 | -23.4 | 21.7 | -2.5 | 11.4 | 21.7 |
| X | 16.0 | -11.0 | 16.2 | -19.7 | -2.4 | -41.5 | -11.4 | 17.7 | 12.6 | 17.7 | 17.7 |
| E | 48.0 | -25.2 | 8.8 | -27.8 | -22.7 | -44.3 | -26.0 | 2.5 | -14.6 | -11.2 | 2.5 |
| S | 37.0 | -18.9 | 6.6 | -21.1 | -16.7 | -32.5 | -18.4 | -6.8 | -11.1 | -8.3 | -6.8 |
| SC | 22.0 | -33.0 | 9.4 | -37.2 | -28.8 | -54.7 | -35.0 | -12.3 | -21.1 | -20.3 | -12.3 |
| NE | 38.0 | -27.0 | 10.0 | -30.3 | -23.7 | -42.8 | -27.5 | -5.6 | -10.6 | -9.1 | -5.6 |
| W | 29.0 | -24.7 | 9.1 | -28.1 | -21.2 | -47.0 | -22.5 | -3.5 | -15.7 | -10.8 | -3.5 |
| YG | 24.0 | -22.5 | 19.7 | -30.9 | -14.2 | -52.8 | -23.4 | 42.2 | -3.7 | -1.2 | 42.2 |
| C | 55.0 | -21.2 | 13.7 | -24.9 | -17.5 | -39.2 | -26.0 | 21.2 | -4.6 | 8.3 | 21.2 |
| Q | 11.0 | -52.2 | 26.2 | -69.8 | -34.6 | -110.8 | -52.3 | -16.8 | -22.3 | -16.8 | -16.8 |
| M | 11.0 | -30.1 | 11.8 | -38.0 | -22.2 | -49.9 | -28.2 | -4.9 | -22.7 | -4.9 | -4.9 |
| CH | 362.0 | -23.8 | 14.5 | -25.3 | -22.3 | -110.8 | -25.1 | 42.2 | -7.6 | -1.9 | 21.2 |

**S17 Table. Annual average absolute change of PM_10_ from 2015 to 2019.**

| Annual average absolute change PM_10_ | Total cities | Mean | S. D. | Upper 95% CI of Mean | Variance | Min | Median | Max | P90 | P95 | P99 | Number of cities at < 0 | Number of cities at > 0 |
| --- | --- | --- | --- | --- | --- | --- | --- | --- | --- | --- | --- | --- | --- |
| N | 71.0 | -6.9 | 5.3 | -8.2 | -5.7 | -20.0 | -7.0 | 7.5 | -0.8 | 2.2 | 7.5 | 65.0 | 6.0 |
| X | 16.0 | -3.3 | 6.3 | -6.6 | 0.1 | -19.8 | -2.3 | 6.4 | 4.6 | 6.4 | 6.4 | 13.0 | 3.0 |
| E | 48.0 | -5.2 | 2.8 | -6.0 | -4.4 | -16.2 | -5.2 | 0.4 | -1.8 | -1.5 | 0.4 | 47.0 | 1.0 |
| S | 37.0 | -2.6 | 1.2 | -3.0 | -2.2 | -5.3 | -2.4 | -0.9 | -1.3 | -1.1 | -0.9 | 37.0 | 0.0 |
| SC | 22.0 | -6.3 | 2.5 | -7.4 | -5.2 | -10.2 | -6.6 | -1.6 | -3.0 | -2.8 | -1.6 | 22.0 | 0.0 |
| NE | 38.0 | -5.5 | 2.5 | -6.4 | -4.7 | -9.8 | -5.1 | -0.8 | -1.9 | -1.6 | -0.8 | 38.0 | 0.0 |
| W | 29.0 | -5.9 | 2.3 | -6.8 | -5.1 | -10.0 | -5.7 | -1.0 | -3.1 | -3.0 | -1.0 | 29.0 | 0.0 |
| YG | 24.0 | -3.0 | 2.8 | -4.2 | -1.9 | -9.4 | -2.5 | 4.3 | -0.4 | -0.1 | 4.3 | 23.0 | 1.0 |
| C | 55.0 | -4.6 | 3.1 | -5.5 | -3.8 | -9.6 | -5.4 | 3.2 | -1.0 | 1.8 | 3.2 | 51.0 | 4.0 |
| Q | 11.0 | -10.3 | 5.0 | -13.7 | -6.9 | -21.4 | -9.6 | -5.1 | -5.4 | -5.1 | -5.1 | 11.0 | 0.0 |
| M | 11.0 | -6.4 | 2.5 | -8.0 | -4.7 | -9.4 | -6.4 | -0.8 | -3.7 | -0.8 | -0.8 | 11.0 | 0.0 |
| CH | 362.0 | -5.3 | 3.9 | -5.7 | -4.9 | -21.4 | -5.2 | 7.5 | -1.3 | -0.4 | 4.3 | 347.0 | 15.0 |

**S18 Table. Annual average relative change of PM_10_ from 2015 to 2019.**

| Annual average relative change of PM_10_ | Total cities | Mean | S. D. | Lower 95% CI of Mean | Upper 95% CI of Mean | Min | Median | Max | P90 | P95 | P99 |
| --- | --- | --- | --- | --- | --- | --- | --- | --- | --- | --- | --- |
| N | 71.0 | -5.6 | 4.2 | -6.6 | -4.7 | -13.6 | -6.2 | 7.3 | -0.4 | 3.1 | 7.3 |
| X | 16.0 | -0.7 | 5.4 | -3.6 | 2.1 | -10.5 | -0.7 | 9.8 | 8.2 | 9.8 | 9.8 |
| E | 48.0 | -6.8 | 2.6 | -7.6 | -6.1 | -11.5 | -7.1 | 0.9 | -3.4 | -2.3 | 0.9 |
| S | 37.0 | -4.8 | 2.0 | -5.4 | -4.1 | -8.5 | -4.7 | -1.3 | -2.4 | -1.6 | -1.3 |
| SC | 22.0 | -9.1 | 3.2 | -10.6 | -7.7 | -16.5 | -9.2 | -1.6 | -5.4 | -5.2 | -1.6 |
| NE | 38.0 | -7.0 | 3.1 | -8.1 | -6.0 | -12.8 | -7.1 | 0.1 | -1.8 | -1.1 | 0.1 |
| W | 29.0 | -5.9 | 2.9 | -7.0 | -4.8 | -12.8 | -5.4 | 0.7 | -3.0 | -1.1 | 0.7 |
| YG | 24.0 | -5.6 | 5.5 | -7.9 | -3.3 | -16.6 | -5.8 | 10.0 | -0.5 | 0.6 | 10.0 |
| C | 55.0 | -5.6 | 4.2 | -6.7 | -4.4 | -11.3 | -7.1 | 7.5 | -0.1 | 3.2 | 7.5 |
| Q | 11.0 | -14.1 | 15.5 | -14.3 | 6.6 | -23.5 | -8.0 | 21.0 | 18.4 | 21.0 | 21.0 |
| M | 11.0 | -7.3 | 4.4 | -10.3 | -4.4 | -14.7 | -7.4 | 0.5 | -1.7 | 0.5 | 0.5 |
| CH | 362.0 | -5.9 | 4.7 | -6.4 | -5.4 | -23.5 | -6.4 | 21.0 | -0.7 | 1.2 | 9.8 |

**S19 Table. Statistical analysis of the AQI in China in 2015.**

| AQI in 2015 | Total cities | Mean | Standard Deviation | Lower 95% CI of Mean | Upper 95% CI of Mean | Min | Median | Max | P90 | P95 | P99 |
| --- | --- | --- | --- | --- | --- | --- | --- | --- | --- | --- | --- |
| N | 71.0 | 103.7 | 21.2 | 98.7 | 108.7 | 64.5 | 106.2 | 144.3 | 129.1 | 138.3 | 144.3 |
| X | 16.0 | 102.5 | 48.2 | 76.8 | 128.2 | 42.6 | 89.8 | 201.3 | 197.5 | 201.3 | 201.3 |
| E | 48.0 | 76.3 | 16.9 | 71.4 | 81.2 | 45.3 | 80.8 | 126.1 | 91.5 | 97.3 | 126.1 |
| S | 37.0 | 55.8 | 7.1 | 53.5 | 58.2 | 36.5 | 54.7 | 71.3 | 64.0 | 69.3 | 71.3 |
| SC | 22.0 | 72.3 | 16.8 | 64.8 | 79.7 | 36.5 | 77.0 | 100.9 | 88.9 | 90.9 | 100.9 |
| NE | 38.0 | 77.9 | 14.4 | 73.2 | 82.6 | 48.7 | 81.8 | 103.0 | 96.4 | 97.8 | 103.0 |
| W | 29.0 | 82.2 | 8.7 | 78.9 | 85.5 | 61.7 | 82.5 | 97.3 | 92.5 | 95.1 | 97.3 |
| YG | 24.0 | 50.9 | 8.1 | 47.5 | 54.4 | 36.3 | 51.1 | 65.6 | 62.5 | 63.7 | 65.6 |
| C | 55.0 | 78.9 | 13.0 | 75.4 | 82.5 | 50.3 | 78.5 | 104.8 | 98.0 | 100.2 | 104.8 |
| Q | 11.0 | 74.8 | 17.1 | 63.4 | 86.3 | 42.7 | 80.1 | 97.2 | 90.7 | 97.2 | 97.2 |
| M | 11.0 | 75.2 | 14.2 | 65.7 | 84.8 | 49.0 | 75.9 | 99.9 | 89.9 | 99.9 | 99.9 |
| CH | 362.0 | 79.8 | 23.9 | 77.3 | 82.2 | 36.3 | 80.0 | 201.3 | 109.9 | 123.5 | 144.3 |

**S20 Table. Statistical analysis of the AQI in China in 2016.**

| AQI in 2016 | Total cities | Mean | S.D. | Lower 95% CI of Mean | Upper 95% CI of Mean | Min | Median | Max | P90 | P95 | P99 |
| --- | --- | --- | --- | --- | --- | --- | --- | --- | --- | --- | --- |
| N | 71.0 | 98.8 | 17.7 | 94.6 | 103.0 | 62.6 | 102.2 | 130.8 | 120.8 | 122.8 | 130.8 |
| X | 16.0 | 105.7 | 49.5 | 79.3 | 132.0 | 40.1 | 98.8 | 204.6 | 184.1 | 204.6 | 204.6 |
| E | 48.0 | 68.9 | 13.5 | 65.0 | 72.9 | 39.9 | 71.7 | 95.1 | 84.9 | 87.2 | 95.1 |
| S | 37.0 | 52.5 | 6.8 | 50.2 | 54.8 | 33.6 | 52.9 | 65.3 | 61.1 | 65.2 | 65.3 |
| SC | 22.0 | 72.1 | 15.7 | 65.1 | 79.0 | 38.2 | 75.9 | 100.2 | 89.4 | 90.7 | 100.2 |
| NE | 38.0 | 68.1 | 12.6 | 64.0 | 72.3 | 35.5 | 71.5 | 88.0 | 81.8 | 84.5 | 88.0 |
| W | 29.0 | 82.4 | 14.9 | 76.7 | 88.1 | 55.2 | 78.4 | 120.9 | 111.4 | 116.1 | 120.9 |
| YG | 24.0 | 49.7 | 8.7 | 46.0 | 53.4 | 33.8 | 48.3 | 67.2 | 63.5 | 66.4 | 67.2 |
| C | 55.0 | 75.1 | 9.9 | 72.4 | 77.8 | 48.0 | 74.4 | 95.5 | 87.4 | 90.5 | 95.5 |
| Q | 11.0 | 70.4 | 17.6 | 58.6 | 82.2 | 39.3 | 73.2 | 93.2 | 88.2 | 93.2 | 93.2 |
| M | 11.0 | 68.9 | 13.0 | 60.1 | 77.7 | 48.1 | 69.2 | 89.1 | 85.4 | 89.1 | 89.1 |
| CH | 362.0 | 75.6 | 23.1 | 73.2 | 78.0 | 33.6 | 73.4 | 204.6 | 106.5 | 118.7 | 146.7 |

**S21 Table. Statistical analysis of the AQI in China in 2017.**

| AQI in 2017 | Total cities | Mean | S. D. | Lower 95% CI of Mean | Upper 95% CI of Mean | Min | Median | Max | P90 | P95 | P99 |
| --- | --- | --- | --- | --- | --- | --- | --- | --- | --- | --- | --- |
| N | 71.0 | 95.6 | 17.0 | 91.6 | 99.7 | 58.4 | 99.7 | 126.2 | 113.4 | 122.1 | 126.2 |
| X | 16.0 | 100.9 | 39.4 | 79.9 | 121.9 | 45.6 | 99.8 | 187.5 | 161.7 | 187.5 | 187.5 |
| E | 48.0 | 68.6 | 13.4 | 64.8 | 72.5 | 42.9 | 69.4 | 106.2 | 85.2 | 87.7 | 106.2 |
| S | 37.0 | 55.5 | 7.3 | 53.1 | 57.9 | 34.9 | 55.8 | 70.8 | 63.8 | 65.5 | 70.8 |
| SC | 22.0 | 68.2 | 15.1 | 61.5 | 74.9 | 32.4 | 71.7 | 95.9 | 82.7 | 82.8 | 95.9 |
| NE | 38.0 | 68.9 | 12.0 | 64.9 | 72.8 | 39.0 | 71.0 | 87.4 | 83.1 | 84.4 | 87.4 |
| W | 29.0 | 82.1 | 14.6 | 76.5 | 87.6 | 57.2 | 79.1 | 120.7 | 112.7 | 113.0 | 120.7 |
| YG | 24.0 | 49.2 | 6.0 | 46.6 | 51.7 | 38.2 | 48.0 | 63.1 | 57.0 | 59.4 | 63.1 |
| C | 55.0 | 77.0 | 11.5 | 73.9 | 80.1 | 51.7 | 76.4 | 102.7 | 96.6 | 100.7 | 102.7 |
| Q | 11.0 | 63.8 | 10.5 | 56.8 | 70.9 | 53.2 | 60.2 | 84.5 | 79.0 | 84.5 | 84.5 |
| M | 11.0 | 67.7 | 14.6 | 57.9 | 77.5 | 46.3 | 67.0 | 86.4 | 84.2 | 86.4 | 86.4 |
| CH | 362.0 | 74.9 | 21.1 | 72.7 | 77.1 | 32.4 | 73.1 | 187.5 | 103.1 | 112.4 | 126.2 |

**S22 Table. Statistical analysis of the AQI in China in 2018.**

| AQI in 2018 | Total cities | Mean | S. D. | Lower 95% CI of Mean | Upper 95% CI of Mean | Min | Median | Max | P90 | P95 | P99 |
| --- | --- | --- | --- | --- | --- | --- | --- | --- | --- | --- | --- |
| N | 71.0 | 90.4 | 15.4 | 86.8 | 94.1 | 54.8 | 94.8 | 115.1 | 106.3 | 110.1 | 115.1 |
| X | 16.0 | 106.9 | 49.9 | 80.3 | 133.5 | 36.8 | 100.4 | 217.2 | 180.1 | 217.2 | 217.2 |
| E | 48.0 | 65.8 | 13.6 | 61.8 | 69.7 | 41.2 | 66.9 | 102.3 | 83.6 | 85.5 | 102.3 |
| S | 37.0 | 53.2 | 6.2 | 51.1 | 55.2 | 34.0 | 52.7 | 62.8 | 60.7 | 61.3 | 62.8 |
| SC | 22.0 | 62.5 | 11.6 | 57.3 | 67.6 | 34.1 | 63.2 | 84.3 | 74.3 | 75.8 | 84.3 |
| NE | 38.0 | 61.3 | 9.9 | 58.0 | 64.5 | 39.5 | 61.4 | 79.7 | 75.1 | 76.2 | 79.7 |
| W | 29.0 | 81.4 | 12.9 | 76.5 | 86.3 | 55.5 | 81.0 | 112.0 | 104.3 | 105.5 | 112.0 |
| YG | 24.0 | 47.8 | 5.0 | 45.7 | 49.9 | 37.3 | 47.4 | 55.2 | 54.8 | 55.1 | 55.2 |
| C | 55.0 | 70.4 | 11.8 | 67.2 | 73.6 | 43.6 | 68.0 | 94.8 | 87.3 | 93.6 | 94.8 |
| Q | 11.0 | 60.7 | 11.1 | 53.2 | 68.2 | 49.8 | 57.3 | 81.9 | 81.6 | 81.9 | 81.9 |
| M | 11.0 | 69.5 | 15.6 | 59.0 | 79.9 | 40.8 | 74.1 | 89.3 | 82.6 | 89.3 | 89.3 |
| CH | 362.0 | 71.2 | 21.7 | 68.9 | 73.4 | 34.0 | 67.2 | 217.2 | 99.8 | 104.3 | 153.0 |

**S23 Table. Statistical analysis of the AQI in China in 2019.**

| AQI in 2019 | Total cities | Mean | S. D. | Lower 95% CI of Mean | Upper 95% CI of Mean | Min | Median | Max | P90 | P95 | P99 |
| --- | --- | --- | --- | --- | --- | --- | --- | --- | --- | --- | --- |
| N | 71.0 | 84.0 | 15.7 | 80.3 | 87.8 | 53.6 | 86.7 | 142.3 | 95.5 | 102.4 | 142.3 |
| X | 16.0 | 90.9 | 41.0 | 69.1 | 112.8 | 32.0 | 87.0 | 183.8 | 151.6 | 183.8 | 183.8 |
| E | 48.0 | 57.8 | 11.3 | 54.5 | 61.1 | 35.2 | 60.1 | 87.8 | 70.7 | 73.6 | 87.8 |
| S | 37.0 | 45.2 | 4.6 | 43.7 | 46.8 | 30.4 | 46.2 | 53.1 | 50.0 | 50.3 | 53.1 |
| SC | 22.0 | 52.8 | 10.1 | 48.4 | 57.3 | 27.2 | 55.3 | 68.5 | 62.1 | 64.3 | 68.5 |
| NE | 38.0 | 58.2 | 11.3 | 54.5 | 61.9 | 34.4 | 58.9 | 80.8 | 71.1 | 75.2 | 80.8 |
| W | 29.0 | 65.5 | 11.7 | 61.0 | 69.9 | 39.8 | 63.5 | 91.6 | 89.6 | 89.9 | 91.6 |
| YG | 24.0 | 41.7 | 5.0 | 39.6 | 43.9 | 33.4 | 42.2 | 51.4 | 48.5 | 50.9 | 51.4 |
| C | 55.0 | 64.1 | 11.2 | 61.1 | 67.1 | 43.4 | 64.8 | 87.4 | 81.7 | 83.0 | 87.4 |
| Q | 11.0 | 48.1 | 13.2 | 39.2 | 57.0 | 28.1 | 48.4 | 77.3 | 59.2 | 77.3 | 77.3 |
| M | 11.0 | 56.0 | 12.5 | 47.6 | 64.4 | 36.5 | 59.3 | 73.8 | 67.7 | 73.8 | 73.8 |
| CH | 362.0 | 63.0 | 20.0 | 61.0 | 65.1 | 27.2 | 60.7 | 183.8 | 90.0 | 94.0 | 133.6 |

**S24 Table. Absolute change of AQI between 2015 and 2019.**

| Absolute change AQI between 2015 and 2019 | Total cities | Mean | S. D. | Lower 95% CI of Mean | Upper 95% CI of Mean | Min | Median | Max | P90 | P95 | P99 |
| --- | --- | --- | --- | --- | --- | --- | --- | --- | --- | --- | --- |
| N | 71.0 | -19.7 | 15.3 | -23.3 | -16.0 | -52.1 | -19.8 | 27.5 | -5.3 | 6.2 | 27.5 |
| X | 16.0 | -11.6 | 13.7 | -18.9 | -4.3 | -46.0 | -8.8 | 8.4 | 8.1 | 8.4 | 8.4 |
| E | 48.0 | -18.5 | 9.4 | -21.3 | -15.8 | -59.6 | -18.9 | -3.3 | -5.7 | -4.4 | -3.3 |
| S | 37.0 | -10.6 | 4.2 | -12.0 | -9.2 | -21.6 | -10.0 | -4.2 | -6.2 | -4.8 | -4.2 |
| SC | 22.0 | -19.4 | 8.8 | -23.3 | -15.5 | -33.5 | -20.4 | -5.7 | -7.7 | -6.1 | -5.7 |
| NE | 38.0 | -19.7 | 8.6 | -22.5 | -16.8 | -35.6 | -17.4 | -3.8 | -7.2 | -4.4 | -3.8 |
| W | 29.0 | -16.7 | 6.7 | -19.3 | -14.2 | -26.4 | -17.5 | -0.9 | -5.5 | -2.2 | -0.9 |
| YG | 24.0 | -9.2 | 8.8 | -12.9 | -5.5 | -29.4 | -8.5 | 9.2 | 0.8 | 2.2 | 9.2 |
| C | 55.0 | -14.8 | 9.3 | -17.3 | -12.3 | -32.0 | -15.9 | 12.1 | -4.1 | 4.7 | 12.1 |
| Q | 11.0 | -26.7 | 13.4 | -35.8 | -17.7 | -57.2 | -24.2 | -11.6 | -13.4 | -11.6 | -11.6 |
| M | 11.0 | -19.2 | 6.6 | -23.7 | -14.8 | -28.3 | -18.0 | -8.7 | -9.0 | -8.7 | -8.7 |
| CH | 362.0 | -16.7 | 11.0 | -17.9 | -15.6 | -59.6 | -16.7 | 27.5 | -5.3 | -2.8 | 9.2 |

**S25 Table. Relative change of AQI between 2015 and 2019.**

| Relative change of AQI between 2015 and 2019 | Total cities | Mean | S. D. | Lower 95% CI of Mean | Upper 95% CI of Mean | Min | Median | Max | P90 | P95 | P99 |
| --- | --- | --- | --- | --- | --- | --- | --- | --- | --- | --- | --- |
| N | 71.0 | -17.8 | 11.9 | -20.6 | -15.0 | -37.0 | -18.5 | 25.9 | -5.8 | 7.1 | 25.9 |
| X | 16.0 | -10.7 | 10.0 | -16.1 | -5.4 | -25.0 | -10.9 | 9.7 | 7.8 | 9.7 | 9.7 |
| E | 48.0 | -23.3 | 8.3 | -25.7 | -20.8 | -47.3 | -24.3 | -6.6 | -10.9 | -7.7 | -6.6 |
| S | 37.0 | -18.6 | 5.5 | -20.4 | -16.7 | -31.2 | -17.3 | -7.7 | -12.8 | -9.0 | -7.7 |
| SC | 22.0 | -25.8 | 8.0 | -29.4 | -22.3 | -38.1 | -25.4 | -10.5 | -15.5 | -14.4 | -10.5 |
| NE | 38.0 | -24.9 | 9.0 | -27.8 | -21.9 | -41.4 | -25.6 | -6.3 | -8.1 | -7.0 | -6.3 |
| W | 29.0 | -20.7 | 8.4 | -23.9 | -17.5 | -35.5 | -21.6 | -1.0 | -5.8 | -2.4 | -1.0 |
| YG | 24.0 | -16.3 | 14.9 | -22.6 | -10.1 | -44.9 | -17.2 | 22.0 | 1.7 | 5.2 | 22.0 |
| C | 55.0 | -18.2 | 11.1 | -21.2 | -15.2 | -35.3 | -19.1 | 22.9 | -6.3 | 6.0 | 22.9 |
| Q | 11.0 | -34.9 | 12.1 | -43.1 | -26.8 | -58.9 | -34.3 | -14.7 | -23.7 | -14.7 | -14.7 |
| M | 11.0 | -25.7 | 8.6 | -31.5 | -19.9 | -41.3 | -24.6 | -12.8 | -18.4 | -12.8 | -12.8 |
| CH | 362.0 | -20.5 | 10.9 | -21.6 | -19.3 | -58.9 | -21.1 | 25.9 | -7.9 | -3.6 | 17.4 |

**S26 Table. Annual average absolute change of AQI from 2015 to 2019.**

| Annual average absolute change of AQI | Total cities | Mean | S. D. | Lower 95% CI of Mean | Upper 95% CI of Mean | Min | Median | Max | P90 | P95 | P99 | Number of cities at < 0 | Number of cities at > 0 |
| --- | --- | --- | --- | --- | --- | --- | --- | --- | --- | --- | --- | --- | --- |
| N | 71.0 | -4.9 | 3.8 | -5.8 | -4.0 | -13.0 | -5.0 | 6.9 | -1.3 | 1.6 | 6.9 | 66.0 | 5.0 |
| X | 16.0 | -2.9 | 3.4 | -4.7 | -1.1 | -11.5 | -2.2 | 2.1 | 2.0 | 2.1 | 2.1 | 14.0 | 2.0 |
| E | 48.0 | -4.6 | 2.4 | -5.3 | -3.9 | -14.9 | -4.7 | -0.8 | -1.4 | -1.1 | -0.8 | 48.0 | 0.0 |
| S | 37.0 | -2.7 | 1.1 | -3.2 | -2.3 | -5.4 | -2.5 | -1.0 | -1.5 | -1.2 | -1.0 | 37.0 | 0.0 |
| SC | 22.0 | -4.9 | 2.2 | -5.8 | -3.9 | -8.4 | -5.1 | -1.4 | -1.9 | -1.5 | -1.4 | 22.0 | 0.0 |
| NE | 38.0 | -4.9 | 2.2 | -5.6 | -4.2 | -8.9 | -4.3 | -0.9 | -1.8 | -1.1 | -0.9 | 38.0 | 0.0 |
| W | 29.0 | -4.2 | 1.7 | -4.8 | -3.5 | -6.6 | -4.4 | -0.2 | -1.4 | -0.5 | -0.2 | 29.0 | 0.0 |
| YG | 24.0 | -2.3 | 2.2 | -3.2 | -1.4 | -7.4 | -2.1 | 2.3 | 0.2 | 0.6 | 2.3 | 21.0 | 3.0 |
| C | 55.0 | -3.7 | 2.3 | -4.3 | -3.1 | -8.0 | -4.0 | 3.0 | -1.0 | 1.2 | 3.0 | 52.0 | 3.0 |
| Q | 11.0 | -6.7 | 3.4 | -8.9 | -4.4 | -14.3 | -6.1 | -2.9 | -3.3 | -2.9 | -2.9 | 11.0 | 0.0 |
| M | 11.0 | -4.8 | 1.6 | -5.9 | -3.7 | -7.1 | -4.5 | -2.2 | -2.3 | -2.2 | -2.2 | 11.0 | 0.0 |
| CH | 362.0 | -4.2 | 2.8 | -4.5 | -3.9 | -14.9 | -4.2 | 6.9 | -1.3 | -0.7 | 2.3 | 349.0 | 13.0 |

**S27 Table. Annual relative change of AQI from 2015 to 2019.**

| Annual average relative change of AQI | Total cities | Mean | Standard Deviation | Lower 95% CI of Mean | Upper 95% CI of Mean | Min | Median | Max | P90 | P95 | P99 |
| --- | --- | --- | --- | --- | --- | --- | --- | --- | --- | --- | --- |
| N | 71.0 | -4.7 | 3.5 | -5.6 | -3.9 | -10.9 | -4.9 | 7.0 | -1.2 | 2.0 | 7.0 |
| X | 16.0 | -2.1 | 2.8 | -3.6 | -0.6 | -6.3 | -2.3 | 3.9 | 2.8 | 3.9 | 3.9 |
| E | 48.0 | -6.3 | 2.5 | -7.0 | -5.5 | -12.8 | -6.5 | -1.1 | -2.4 | -1.7 | -1.1 |
| S | 37.0 | -4.7 | 1.7 | -5.3 | -4.1 | -8.6 | -4.4 | -1.5 | -3.0 | -1.6 | -1.5 |
| SC | 22.0 | -6.9 | 2.6 | -8.0 | -5.8 | -11.2 | -6.9 | -2.6 | -3.1 | -3.0 | -2.6 |
| NE | 38.0 | -6.6 | 2.8 | -7.5 | -5.7 | -12.3 | -6.8 | -0.1 | -1.7 | -0.9 | -0.1 |
| W | 29.0 | -5.1 | 2.5 | -6.1 | -4.2 | -9.5 | -5.5 | 1.0 | -0.8 | 0.7 | 1.0 |
| YG | 24.0 | -4.1 | 4.2 | -5.9 | -2.4 | -13.4 | -4.2 | 5.5 | 0.5 | 1.5 | 5.5 |
| C | 55.0 | -4.7 | 3.3 | -5.6 | -3.8 | -10.2 | -5.0 | 7.2 | -0.8 | 2.1 | 7.2 |
| Q | 11.0 | -9.4 | 4.8 | -12.6 | -6.2 | -19.4 | -7.7 | -3.8 | -3.9 | -3.8 | -3.8 |
| M | 11.0 | -6.7 | 3.2 | -8.8 | -4.5 | -12.4 | -6.6 | -2.5 | -2.6 | -2.5 | -2.5 |
| CH | 362.0 | -5.3 | 3.3 | -5.7 | -5.0 | -19.4 | -5.5 | 7.2 | -1.6 | -0.1 | 5.5 |

**S28 Table. Statistical analysis of the SO_2_ (unit: μg/m^3^) in China in 2015.**

| SO_2_ in 2015 | Total cities | Mean | S. D. | Lower 95% CI of Mean | Upper 95% CI of Mean | Min | Median | Max | P90 | P95 | P99 | Number of cities at < 20 |
| --- | --- | --- | --- | --- | --- | --- | --- | --- | --- | --- | --- | --- |
| N | 71.0 | 40.6 | 16.0 | 36.8 | 44.4 | 12.6 | 40.5 | 81.7 | 60.0 | 68.0 | 81.7 | 6.0 |
| X | 16.0 | 18.0 | 15.1 | 9.9 | 26.0 | 7.5 | 16.7 | 72.1 | 20.5 | 72.1 | 72.1 | 14.0 |
| E | 48.0 | 19.2 | 7.3 | 17.1 | 21.4 | 5.5 | 20.2 | 37.7 | 28.3 | 29.3 | 37.7 | 24.0 |
| S | 37.0 | 13.8 | 5.5 | 12.0 | 15.6 | 2.8 | 13.0 | 25.4 | 20.6 | 24.0 | 25.4 | 32.0 |
| SC | 22.0 | 17.2 | 6.8 | 14.2 | 20.2 | 6.4 | 15.5 | 32.8 | 24.7 | 30.0 | 32.8 | 15.0 |
| NE | 38.0 | 28.8 | 12.5 | 24.7 | 32.9 | 8.4 | 27.0 | 61.2 | 48.5 | 57.6 | 61.2 | 10.0 |
| W | 29.0 | 27.2 | 13.7 | 22.0 | 32.4 | 12.7 | 22.9 | 70.7 | 50.9 | 57.3 | 70.7 | 7.0 |
| YG | 24.0 | 17.9 | 8.1 | 14.5 | 21.3 | 6.1 | 15.8 | 36.8 | 34.2 | 35.6 | 36.8 | 17.0 |
| C | 55.0 | 22.6 | 8.1 | 20.4 | 24.8 | 8.9 | 22.3 | 42.9 | 33.3 | 38.1 | 42.9 | 22.0 |
| Q | 11.0 | 20.4 | 8.5 | 14.7 | 26.1 | 9.4 | 18.9 | 34.1 | 33.0 | 34.1 | 34.1 | 6.0 |
| M | 11.0 | 28.3 | 16.4 | 17.3 | 39.3 | 10.0 | 25.1 | 62.2 | 47.9 | 62.2 | 62.2 | 4.0 |
| CH | 362.0 | 25.1 | 14.3 | 23.6 | 26.5 | 2.8 | 21.1 | 81.7 | 46.3 | 56.5 | 71.7 | 157.0 |

**S29 Table. Statistical analysis of the SO_2_ (unit: μg/m^3^) in China in 2016.**

| SO_2_ in 2016 | Total cities | Mean | Standard Deviation | Lower 95% CI of Mean | Upper 95% CI of Mean | Min | Median | Max | P90 | P95 | P99 |
| --- | --- | --- | --- | --- | --- | --- | --- | --- | --- | --- | --- |
| N | 71.0 | 35.2 | 16.8 | 31.2 | 39.1 | 9.5 | 31.1 | 87.4 | 61.4 | 66.1 | 87.4 |
| X | 16.0 | 14.5 | 9.6 | 9.4 | 19.6 | 4.9 | 13.9 | 46.2 | 21.4 | 46.2 | 46.2 |
| E | 48.0 | 16.2 | 7.7 | 13.9 | 18.4 | 5.5 | 16.1 | 50.2 | 23.8 | 25.6 | 50.2 |
| S | 37.0 | 12.3 | 4.7 | 10.7 | 13.8 | 2.8 | 11.7 | 22.8 | 18.9 | 21.0 | 22.8 |
| SC | 22.0 | 16.1 | 6.8 | 13.0 | 19.1 | 4.1 | 14.9 | 37.8 | 22.7 | 27.8 | 37.8 |
| NE | 38.0 | 24.8 | 10.3 | 21.4 | 28.1 | 8.0 | 24.9 | 51.5 | 38.5 | 46.9 | 51.5 |
| W | 29.0 | 24.5 | 12.6 | 19.7 | 29.3 | 10.3 | 20.9 | 66.6 | 41.4 | 54.7 | 66.6 |
| YG | 24.0 | 15.0 | 5.6 | 12.6 | 17.3 | 6.6 | 13.7 | 26.9 | 22.5 | 22.7 | 26.9 |
| C | 55.0 | 18.9 | 6.8 | 17.1 | 20.8 | 6.4 | 18.7 | 42.1 | 27.1 | 30.5 | 42.1 |
| Q | 11.0 | 18.5 | 6.0 | 14.5 | 22.6 | 7.3 | 18.8 | 25.7 | 25.5 | 25.7 | 25.7 |
| M | 11.0 | 23.8 | 13.5 | 14.7 | 32.8 | 6.2 | 24.5 | 55.3 | 32.1 | 55.3 | 55.3 |
| CH | 362.0 | 21.6 | 13.0 | 20.3 | 23.0 | 2.8 | 18.2 | 87.4 | 38.1 | 47.3 | 66.6 |

**S30 Table. Statistical analysis of the SO_2_ (unit: μg/m^3^) in China in 2017.**

| SO_2_ in 2017 | Total cities | Mean | Standard Deviation | Lower 95% CI of Mean | Upper 95% CI of Mean | Min | Median | Max | P90 | P95 | P99 |
| --- | --- | --- | --- | --- | --- | --- | --- | --- | --- | --- | --- |
| N | 71.0 | 26.7 | 15.3 | 23.1 | 30.3 | 7.2 | 23.7 | 83.3 | 46.1 | 51.5 | 83.3 |
| X | 16.0 | 13.2 | 7.3 | 9.4 | 17.1 | 4.6 | 12.2 | 35.1 | 23.1 | 35.1 | 35.1 |
| E | 48.0 | 12.9 | 3.8 | 11.8 | 14.0 | 5.9 | 12.7 | 21.6 | 17.9 | 19.6 | 21.6 |
| S | 37.0 | 11.4 | 3.8 | 10.1 | 12.6 | 2.3 | 11.4 | 19.4 | 17.3 | 18.7 | 19.4 |
| SC | 22.0 | 13.4 | 6.1 | 10.7 | 16.1 | 3.7 | 11.6 | 35.2 | 18.1 | 18.9 | 35.2 |
| NE | 38.0 | 20.6 | 9.0 | 17.6 | 23.5 | 7.7 | 20.1 | 44.8 | 30.9 | 43.0 | 44.8 |
| W | 29.0 | 20.8 | 11.0 | 16.7 | 25.0 | 10.2 | 18.4 | 51.5 | 44.8 | 51.4 | 51.5 |
| YG | 24.0 | 13.2 | 4.9 | 11.1 | 15.2 | 5.6 | 12.1 | 26.3 | 19.1 | 19.6 | 26.3 |
| C | 55.0 | 15.9 | 6.0 | 14.3 | 17.5 | 4.0 | 15.0 | 34.4 | 25.7 | 26.9 | 34.4 |
| Q | 11.0 | 17.0 | 5.4 | 13.3 | 20.6 | 7.4 | 17.6 | 26.7 | 20.5 | 26.7 | 26.7 |
| M | 11.0 | 21.0 | 12.7 | 12.5 | 29.6 | 3.5 | 19.9 | 50.6 | 28.5 | 50.6 | 50.6 |
| CH | 362.0 | 17.8 | 10.6 | 16.7 | 18.9 | 2.3 | 15.0 | 83.3 | 28.5 | 39.4 | 51.5 |

**S31 Table. Statistical analysis of the SO_2_ (unit: μg/m^3^) in China in 2018.**

| SO_2_ in 2018 | Total cities | Mean | Standard Deviation | Lower 95% CI of Mean | Upper 95% CI of Mean | Min | Median | Max | P90 | P95 | P99 |
| --- | --- | --- | --- | --- | --- | --- | --- | --- | --- | --- | --- |
| N | 71.0 | 17.9 | 8.3 | 15.9 | 19.8 | 5.6 | 16.9 | 48.2 | 29.3 | 33.6 | 48.2 |
| X | 16.0 | 10.6 | 4.7 | 8.1 | 13.1 | 4.4 | 10.6 | 20.6 | 20.4 | 20.6 | 20.6 |
| E | 48.0 | 10.3 | 3.2 | 9.3 | 11.2 | 5.6 | 9.8 | 19.0 | 15.3 | 16.1 | 19.0 |
| S | 37.0 | 10.3 | 3.3 | 9.2 | 11.4 | 3.2 | 10.0 | 18.1 | 14.3 | 16.7 | 18.1 |
| SC | 22.0 | 11.3 | 6.9 | 8.3 | 14.4 | 3.7 | 9.6 | 38.5 | 15.2 | 17.0 | 38.5 |
| NE | 38.0 | 15.7 | 7.4 | 13.3 | 18.1 | 5.7 | 13.6 | 37.5 | 26.0 | 35.6 | 37.5 |
| W | 29.0 | 16.3 | 8.4 | 13.1 | 19.5 | 7.7 | 13.9 | 41.7 | 36.0 | 36.6 | 41.7 |
| YG | 24.0 | 11.2 | 3.8 | 9.6 | 12.9 | 5.3 | 11.2 | 18.8 | 16.3 | 16.5 | 18.8 |
| C | 55.0 | 12.1 | 4.2 | 10.9 | 13.2 | 3.6 | 11.3 | 22.4 | 17.6 | 19.4 | 22.4 |
| Q | 11.0 | 13.6 | 5.5 | 10.0 | 17.3 | 4.4 | 15.0 | 22.6 | 17.5 | 22.6 | 22.6 |
| M | 11.0 | 16.4 | 8.7 | 10.6 | 22.3 | 3.4 | 18.6 | 34.2 | 23.0 | 34.2 | 34.2 |
| CH | 362.0 | 13.5 | 6.8 | 12.8 | 14.2 | 3.2 | 12.1 | 48.2 | 20.8 | 26.0 | 38.5 |

**S32 Table. Statistical analysis of the SO_2_ (unit: μg/m^3^) in China in 2019.**

| SO_2_ in 2019 | Total cities | Mean | S. D. | Lower 95% CI of Mean | Upper 95% CI of Mean | Min | Median | Max | P90 | P95 | P99 | Number of cities at < 20 |
| --- | --- | --- | --- | --- | --- | --- | --- | --- | --- | --- | --- | --- |
| N | 71.0 | 14.3 | 6.3 | 12.8 | 15.8 | 4.2 | 13.1 | 32.3 | 22.2 | 28.9 | 32.3 | 60.0 |
| X | 16.0 | 8.4 | 3.0 | 6.9 | 10.0 | 3.8 | 8.2 | 15.5 | 12.7 | 15.5 | 15.5 | 16.0 |
| E | 48.0 | 7.8 | 2.3 | 7.1 | 8.5 | 3.0 | 7.4 | 11.9 | 10.9 | 11.3 | 11.9 | 48.0 |
| S | 37.0 | 8.6 | 2.7 | 7.7 | 9.5 | 3.5 | 8.2 | 15.2 | 11.8 | 13.7 | 15.2 | 37.0 |
| SC | 22.0 | 8.9 | 5.1 | 6.6 | 11.2 | 3.9 | 7.6 | 29.0 | 13.3 | 14.1 | 29.0 | 21.0 |
| NE | 38.0 | 12.8 | 5.9 | 10.8 | 14.7 | 5.0 | 10.5 | 26.5 | 23.0 | 26.4 | 26.5 | 34.0 |
| W | 29.0 | 12.4 | 6.6 | 9.9 | 14.9 | 6.6 | 10.5 | 38.7 | 19.8 | 26.5 | 38.7 | 27.0 |
| YG | 24.0 | 9.3 | 3.3 | 7.9 | 10.7 | 3.9 | 9.3 | 18.1 | 12.3 | 13.5 | 18.1 | 24.0 |
| C | 55.0 | 9.4 | 3.1 | 8.5 | 10.2 | 3.4 | 8.9 | 17.1 | 14.0 | 14.5 | 17.1 | 55.0 |
| Q | 11.0 | 10.7 | 4.3 | 7.9 | 13.6 | 4.3 | 9.4 | 18.4 | 15.5 | 18.4 | 18.4 | 11.0 |
| M | 11.0 | 14.4 | 8.2 | 8.9 | 19.9 | 2.9 | 13.0 | 32.8 | 20.7 | 32.8 | 32.8 | 9.0 |
| CH | 362.0 | 10.8 | 5.3 | 10.2 | 11.3 | 2.9 | 9.5 | 38.7 | 17.1 | 20.7 | 29.5 | 342.0 |

**S33 Table. Absolute change of SO_2_ between 2015 and 2019.**

| Absolute change of SO_2_ between 2015 and 2019 | Total cities | Mean | S. D. | Lower 95% CI of Mean | Upper 95% CI of Mean | Min | Median | Max | P90 | P95 | P99 | Number of cities at < 0 | Number of cities at > 0 |
| --- | --- | --- | --- | --- | --- | --- | --- | --- | --- | --- | --- | --- | --- |
| N | 71.0 | -26.3 | 11.7 | -29.1 | -23.5 | -60.2 | -26.1 | -7.9 | -12.2 | -8.8 | -7.9 | 71.0 | 0.0 |
| X | 16.0 | -9.5 | 13.8 | -16.9 | -2.2 | -59.3 | -7.5 | -0.4 | -1.8 | -0.4 | -0.4 | 16.0 | 0.0 |
| E | 48.0 | -11.5 | 6.2 | -13.3 | -9.6 | -27.3 | -11.5 | 0.4 | -2.1 | -0.9 | 0.4 | 47.0 | 1.0 |
| S | 37.0 | -5.2 | 3.9 | -6.5 | -3.9 | -13.2 | -4.4 | 1.2 | -0.4 | 0.7 | 1.2 | 34.0 | 3.0 |
| SC | 22.0 | -8.3 | 5.9 | -10.9 | -5.7 | -23.4 | -8.7 | 0.9 | -1.1 | 0.8 | 0.9 | 20.0 | 2.0 |
| NE | 38.0 | -16.1 | 8.5 | -18.9 | -13.3 | -41.5 | -14.5 | -0.6 | -6.9 | -2.3 | -0.6 | 38.0 | 0.0 |
| W | 29.0 | -14.8 | 10.3 | -18.7 | -10.8 | -44.2 | -12.8 | -0.1 | -2.8 | -1.9 | -0.1 | 29.0 | 0.0 |
| YG | 24.0 | -8.6 | 7.8 | -11.9 | -5.3 | -27.1 | -6.5 | 5.6 | -2.1 | 3.3 | 5.6 | 22.0 | 2.0 |
| C | 55.0 | -13.2 | 6.9 | -15.1 | -11.4 | -28.9 | -11.3 | 0.0 | -6.3 | -4.1 | 0.0 | 55.0 | 0.0 |
| Q | 11.0 | -9.7 | 8.2 | -15.2 | -4.2 | -29.8 | -8.2 | -2.5 | -3.3 | -2.5 | -2.5 | 11.0 | 0.0 |
| M | 11.0 | -13.9 | 9.8 | -20.5 | -7.3 | -30.2 | -8.5 | -1.9 | -6.0 | -1.9 | -1.9 | 11.0 | 0.0 |
| CH | 362.0 | -14.3 | 10.9 | -15.4 | -13.2 | -60.2 | -11.7 | 5.6 | -2.8 | -1.2 | 0.9 | 354.0 | 8.0 |

**S34 Table. Relative change of SO_2_ between 2015 and 2019.**

| Relative change of SO_2_ between 2015 and 2019 | Total cities | Mean | S. D. | Lower 95% CI of Mean | Upper 95% CI of Mean | Min | Median | Max | P90 | P95 | P99 |
| --- | --- | --- | --- | --- | --- | --- | --- | --- | --- | --- | --- |
| N | 71.0 | -63.9 | 9.7 | -66.2 | -61.5 | -83.7 | -66.5 | -31.6 | -50.3 | -45.3 | -31.6 |
| X | 16.0 | -42.9 | 20.6 | -53.9 | -31.9 | -82.3 | -46.8 | -4.7 | -13.4 | -4.7 | -4.7 |
| E | 48.0 | -54.5 | 18.8 | -59.9 | -49.1 | -86.0 | -60.4 | 8.0 | -22.8 | -9.0 | 8.0 |
| S | 37.0 | -32.2 | 20.8 | -39.1 | -25.3 | -66.6 | -36.7 | 25.4 | -7.6 | 21.3 | 25.4 |
| SC | 22.0 | -44.8 | 24.8 | -55.8 | -33.8 | -77.7 | -53.5 | 12.5 | -10.8 | 6.6 | 12.5 |
| NE | 38.0 | -53.8 | 14.8 | -58.7 | -48.9 | -75.5 | -55.6 | -7.0 | -34.5 | -27.2 | -7.0 |
| W | 29.0 | -50.9 | 18.6 | -58.0 | -43.8 | -79.3 | -57.3 | -0.8 | -21.8 | -14.6 | -0.8 |
| YG | 24.0 | -40.1 | 31.9 | -53.6 | -26.6 | -77.9 | -45.7 | 54.0 | -22.5 | 44.5 | 54.0 |
| C | 55.0 | -55.7 | 15.9 | -60.0 | -51.4 | -79.4 | -57.4 | -0.3 | -37.4 | -28.9 | -0.3 |
| Q | 11.0 | -42.6 | 18.6 | -55.2 | -30.1 | -87.4 | -39.6 | -22.3 | -26.3 | -22.3 | -22.3 |
| M | 11.0 | -47.3 | 17.3 | -58.9 | -35.7 | -74.4 | -47.4 | -19.3 | -23.8 | -19.3 | -19.3 |
| CH | 362.0 | -51.2 | 20.5 | -53.4 | -49.1 | -87.4 | -55.5 | 54.0 | -23.9 | -11.6 | 21.3 |

**S35 Table. Annual average absolute change of SO_2_ from 2015 to 2019.**

| Annual average absolute change of SO_2_ | Total cities | Mean | S. D. | Lower 95% CI of Mean | Upper 95% CI of Mean | Min | Median | Max | P90 | P95 | P99 | Number of cities at < 0 | Number of cities at > 0 |
| --- | --- | --- | --- | --- | --- | --- | --- | --- | --- | --- | --- | --- | --- |
| N | 71.0 | -26.3 | 11.7 | -29.1 | -23.5 | -60.2 | -26.1 | -7.9 | -12.2 | -8.8 | -7.9 | 71.0 | 0.0 |
| X | 16.0 | -9.5 | 13.8 | -16.9 | -2.2 | -59.3 | -7.5 | -0.4 | -1.8 | -0.4 | -0.4 | 16.0 | 0.0 |
| E | 48.0 | -11.4 | 6.2 | -13.3 | -9.6 | -27.3 | -11.5 | 0.4 | -2.1 | -0.9 | 0.4 | 47.0 | 1.0 |
| S | 37.0 | -5.2 | 3.8 | -6.5 | -3.9 | -13.2 | -4.4 | 1.2 | -0.4 | 0.7 | 1.2 | 34.0 | 3.0 |
| SC | 22.0 | -8.3 | 5.9 | -10.9 | -5.7 | -23.4 | -8.7 | 0.9 | -1.1 | 0.8 | 0.9 | 20.0 | 2.0 |
| NE | 38.0 | -16.1 | 8.5 | -18.9 | -13.3 | -41.5 | -14.5 | -0.6 | -6.9 | -2.3 | -0.6 | 38.0 | 0.0 |
| W | 29.0 | -14.8 | 10.3 | -18.7 | -10.8 | -44.2 | -12.8 | -0.1 | -2.8 | -1.9 | -0.1 | 29.0 | 0.0 |
| YG | 24.0 | -8.6 | 7.8 | -11.9 | -5.3 | -27.1 | -6.4 | 5.6 | -2.1 | 3.3 | 5.6 | 22.0 | 2.0 |
| C | 55.0 | -13.2 | 6.9 | -15.1 | -11.4 | -28.9 | -11.3 | 0.0 | -6.3 | -4.1 | 0.0 | 55.0 | 0.0 |
| Q | 11.0 | -9.7 | 8.2 | -15.2 | -4.2 | -29.8 | -8.2 | -2.5 | -3.3 | -2.5 | -2.5 | 11.0 | 0.0 |
| M | 11.0 | -13.9 | 9.8 | -20.5 | -7.3 | -30.2 | -8.5 | -1.9 | -6.0 | -1.9 | -1.9 | 11.0 | 0.0 |
| CH | 362.0 | -14.3 | 10.9 | -15.4 | -13.2 | -60.2 | -11.7 | 5.6 | -2.8 | -1.2 | 0.9 | 354.0 | 8.0 |

**S36 Table. Annual average relative change of SO_2_ from 2015 to 2019.**

| Annual average relative change of SO_2_ | Total cities | Mean | Standard Deviation | Lower 95% CI of Mean | Upper 95% CI of Mean | Min | Median | Max | P90 | P95 | P99 |
| --- | --- | --- | --- | --- | --- | --- | --- | --- | --- | --- | --- |
| N | 71.0 | -21.8 | 5.4 | -23.1 | -20.5 | -35.5 | -23.3 | -7.0 | -14.7 | -12.4 | -7.0 |
| X | 16.0 | -13.3 | 8.9 | -18.1 | -8.5 | -34.9 | -14.1 | 0.0 | -2.8 | 0.0 | 0.0 |
| E | 48.0 | -17.5 | 7.6 | -19.7 | -15.3 | -29.8 | -19.3 | 2.6 | -4.8 | -1.6 | 2.6 |
| S | 37.0 | -8.9 | 7.1 | -11.3 | -6.5 | -22.6 | -9.6 | 11.0 | -0.5 | 7.4 | 11.0 |
| SC | 22.0 | -13.5 | 10.4 | -18.1 | -8.9 | -30.0 | -16.6 | 12.0 | -1.8 | 5.6 | 12.0 |
| NE | 38.0 | -16.8 | 7.2 | -19.2 | -14.4 | -27.8 | -17.5 | 2.1 | -7.4 | -0.8 | 2.1 |
| W | 29.0 | -16.0 | 7.4 | -18.8 | -13.2 | -31.5 | -17.3 | 0.0 | -5.3 | -3.2 | 0.0 |
| YG | 24.0 | -12.5 | 11.1 | -17.1 | -7.8 | -29.7 | -12.9 | 14.1 | -3.7 | 14.1 | 14.1 |
| C | 55.0 | -17.8 | 7.5 | -19.8 | -15.8 | -32.0 | -17.9 | 5.3 | -9.6 | -6.3 | 5.3 |
| Q | 11.0 | -11.8 | 9.9 | -18.5 | -5.2 | -35.9 | -11.3 | 1.4 | -1.1 | 1.4 | 1.4 |
| M | 11.0 | -14.3 | 7.4 | -19.3 | -9.4 | -26.5 | -14.1 | -1.1 | -6.1 | -1.1 | -1.1 |
| CH | 362.0 | -16.3 | 8.5 | -17.2 | -15.4 | -35.9 | -17.2 | 14.1 | -5.2 | -1.1 | 11.0 |

**S37 Table. Statistical analysis of the CO (unit: mg/m^3^) in China in 2015.**

| CO in 2015 | Total cities | Mean | S. D. | Lower 95% CI of Mean | Upper 95% CI of Mean | Min | Median | Max | P90 | P95 | P99 | Number of cities at < 1 |
| --- | --- | --- | --- | --- | --- | --- | --- | --- | --- | --- | --- | --- |
| N | 71.0 | 1.39 | 0.45 | 1.29 | 1.50 | 0.45 | 1.37 | 2.75 | 1.96 | 2.11 | 2.75 | 17.0 |
| X | 16.0 | 1.25 | 0.26 | 1.11 | 1.39 | 0.93 | 1.23 | 1.82 | 1.66 | 1.82 | 1.82 | 3.0 |
| E | 48.0 | 0.93 | 0.21 | 0.87 | 1.00 | 0.59 | 0.92 | 1.82 | 1.15 | 1.24 | 1.82 | 33.0 |
| S | 37.0 | 0.97 | 0.14 | 0.92 | 1.02 | 0.63 | 0.96 | 1.25 | 1.16 | 1.25 | 1.25 | 21.0 |
| SC | 22.0 | 0.91 | 0.24 | 0.80 | 1.02 | 0.47 | 0.94 | 1.68 | 1.06 | 1.10 | 1.68 | 15.0 |
| NE | 38.0 | 1.00 | 0.33 | 0.89 | 1.10 | 0.43 | 0.96 | 1.82 | 1.51 | 1.62 | 1.82 | 22.0 |
| W | 29.0 | 1.17 | 0.33 | 1.04 | 1.29 | 0.74 | 1.14 | 1.78 | 1.76 | 1.78 | 1.78 | 11.0 |
| YG | 24.0 | 0.80 | 0.18 | 0.72 | 0.87 | 0.52 | 0.75 | 1.36 | 0.99 | 1.05 | 1.36 | 22.0 |
| C | 55.0 | 1.07 | 0.28 | 1.00 | 1.15 | 0.21 | 1.05 | 1.65 | 1.46 | 1.57 | 1.65 | 21.0 |
| Q | 11.0 | 0.97 | 0.47 | 0.65 | 1.29 | 0.48 | 0.78 | 2.09 | 1.39 | 2.09 | 2.09 | 7.0 |
| M | 11.0 | 0.87 | 0.28 | 0.68 | 1.05 | 0.50 | 0.74 | 1.39 | 1.28 | 1.39 | 1.39 | 8.0 |
| CH | 362.0 | 1.08 | 0.36 | 1.04 | 1.11 | 0.21 | 1.00 | 2.75 | 1.59 | 1.80 | 2.11 | 180.0 |

**S38 Table. Statistical analysis of the CO (unit: mg/m^3^) in China in 2016.**

| CO in 2016 | Total cities | Mean | Standard Deviation | Lower 95% CI of Mean | Upper 95% CI of Mean | Min | Median | Max | P90 | P95 | P99 |
| --- | --- | --- | --- | --- | --- | --- | --- | --- | --- | --- | --- |
| N | 71.0 | 1.34 | 0.42 | 1.24 | 1.44 | 0.57 | 1.31 | 2.52 | 1.96 | 2.06 | 2.52 |
| X | 16.0 | 1.30 | 0.23 | 1.18 | 1.43 | 0.92 | 1.26 | 1.85 | 1.66 | 1.85 | 1.85 |
| E | 48.0 | 0.87 | 0.16 | 0.82 | 0.91 | 0.59 | 0.85 | 1.33 | 1.10 | 1.16 | 1.33 |
| S | 37.0 | 0.90 | 0.13 | 0.85 | 0.94 | 0.54 | 0.90 | 1.13 | 1.05 | 1.07 | 1.13 |
| SC | 22.0 | 0.90 | 0.19 | 0.81 | 0.99 | 0.52 | 0.90 | 1.51 | 1.07 | 1.09 | 1.51 |
| NE | 38.0 | 0.96 | 0.29 | 0.87 | 1.06 | 0.46 | 0.90 | 1.83 | 1.35 | 1.43 | 1.83 |
| W | 29.0 | 1.05 | 0.26 | 0.95 | 1.15 | 0.62 | 1.07 | 1.66 | 1.38 | 1.50 | 1.66 |
| YG | 24.0 | 0.80 | 0.20 | 0.72 | 0.89 | 0.51 | 0.79 | 1.51 | 0.97 | 1.00 | 1.51 |
| C | 55.0 | 1.02 | 0.25 | 0.95 | 1.09 | 0.51 | 0.98 | 1.61 | 1.33 | 1.48 | 1.61 |
| Q | 11.0 | 0.92 | 0.35 | 0.68 | 1.16 | 0.53 | 0.76 | 1.39 | 1.37 | 1.39 | 1.39 |
| M | 11.0 | 0.81 | 0.29 | 0.62 | 1.01 | 0.45 | 0.69 | 1.34 | 1.20 | 1.34 | 1.34 |
| CH | 362.0 | 1.03 | 0.33 | 0.99 | 1.06 | 0.45 | 0.94 | 2.52 | 1.49 | 1.61 | 2.06 |

**S39 Table. Statistical analysis of the CO (unit: mg/m^3^) in China in 2017.**

| CO in 2017 | Total cities | Mean | S. D. | Lower 95% CI of Mean | Upper 95% CI of Mean | Min | Median | Max | P90 | P95 | P99 |
| --- | --- | --- | --- | --- | --- | --- | --- | --- | --- | --- | --- |
| N | 71.0 | 1.20 | 0.34 | 1.12 | 1.28 | 0.60 | 1.20 | 2.07 | 1.67 | 1.85 | 2.07 |
| X | 16.0 | 1.27 | 0.24 | 1.14 | 1.40 | 0.89 | 1.23 | 1.83 | 1.51 | 1.83 | 1.83 |
| E | 48.0 | 0.81 | 0.11 | 0.78 | 0.85 | 0.51 | 0.81 | 1.01 | 0.99 | 1.00 | 1.01 |
| S | 37.0 | 0.87 | 0.12 | 0.83 | 0.91 | 0.57 | 0.87 | 1.06 | 1.03 | 1.06 | 1.06 |
| SC | 22.0 | 0.82 | 0.24 | 0.72 | 0.93 | 0.48 | 0.82 | 1.62 | 0.95 | 1.17 | 1.62 |
| NE | 38.0 | 0.87 | 0.23 | 0.79 | 0.94 | 0.40 | 0.88 | 1.31 | 1.19 | 1.30 | 1.31 |
| W | 29.0 | 0.93 | 0.28 | 0.82 | 1.04 | 0.53 | 0.87 | 1.54 | 1.44 | 1.49 | 1.54 |
| YG | 24.0 | 0.81 | 0.16 | 0.74 | 0.88 | 0.51 | 0.79 | 1.10 | 1.01 | 1.02 | 1.10 |
| C | 55.0 | 0.99 | 0.21 | 0.93 | 1.05 | 0.63 | 0.98 | 1.62 | 1.27 | 1.33 | 1.62 |
| Q | 11.0 | 0.67 | 0.20 | 0.53 | 0.81 | 0.35 | 0.63 | 1.10 | 0.88 | 1.10 | 1.10 |
| M | 11.0 | 0.83 | 0.32 | 0.61 | 1.05 | 0.50 | 0.73 | 1.34 | 1.26 | 1.34 | 1.34 |
| CH | 362.0 | 0.95 | 0.29 | 0.92 | 0.98 | 0.35 | 0.89 | 2.07 | 1.35 | 1.49 | 1.85 |

**S40 Table. Statistical analysis of the CO (unit: mg/m^3^) in China in 2018.**

| CO in 2018 | Total cities | Mean | Standard Deviation | Lower 95% CI of Mean | Upper 95% CI of Mean | Min | Median | Max | P90 | P95 | P99 |
| --- | --- | --- | --- | --- | --- | --- | --- | --- | --- | --- | --- |
| N | 71.0 | 1.01 | 0.25 | 0.95 | 1.07 | 0.50 | 0.97 | 1.76 | 1.35 | 1.42 | 1.76 |
| X | 16.0 | 1.03 | 0.25 | 0.89 | 1.16 | 0.63 | 1.05 | 1.46 | 1.29 | 1.46 | 1.46 |
| E | 48.0 | 0.77 | 0.13 | 0.73 | 0.81 | 0.54 | 0.74 | 1.08 | 0.98 | 0.99 | 1.08 |
| S | 37.0 | 0.81 | 0.13 | 0.77 | 0.85 | 0.58 | 0.79 | 1.10 | 0.97 | 1.02 | 1.10 |
| SC | 22.0 | 0.75 | 0.22 | 0.66 | 0.85 | 0.40 | 0.74 | 1.53 | 0.90 | 0.90 | 1.53 |
| NE | 38.0 | 0.77 | 0.22 | 0.69 | 0.84 | 0.40 | 0.74 | 1.26 | 1.04 | 1.23 | 1.26 |
| W | 29.0 | 0.81 | 0.24 | 0.72 | 0.91 | 0.45 | 0.77 | 1.35 | 1.23 | 1.23 | 1.35 |
| YG | 24.0 | 0.75 | 0.15 | 0.69 | 0.82 | 0.56 | 0.72 | 1.19 | 0.91 | 0.97 | 1.19 |
| C | 55.0 | 0.91 | 0.19 | 0.85 | 0.96 | 0.57 | 0.91 | 1.45 | 1.16 | 1.24 | 1.45 |
| Q | 11.0 | 0.80 | 0.29 | 0.61 | 1.00 | 0.52 | 0.77 | 1.41 | 1.13 | 1.41 | 1.41 |
| M | 11.0 | 0.71 | 0.27 | 0.53 | 0.89 | 0.39 | 0.59 | 1.15 | 1.12 | 1.15 | 1.15 |
| CH | 362.0 | 0.85 | 0.23 | 0.83 | 0.88 | 0.39 | 0.81 | 1.76 | 1.16 | 1.26 | 1.53 |

**S41 Table. Statistical analysis of the CO (unit: mg/m^3^) in China in 2019.**

| CO in 2019 | Total cities | Mean | S. D. | Lower 95% CI of Mean | Upper 95% CI of Mean | Min | Median | Max | P90 | P95 | P99 | Number of cities at < 1 |
| --- | --- | --- | --- | --- | --- | --- | --- | --- | --- | --- | --- | --- |
| N | 71.0 | 0.89 | 0.21 | 0.84 | 0.94 | 0.52 | 0.84 | 1.53 | 1.19 | 1.35 | 1.53 | 55.0 |
| X | 16.0 | 0.82 | 0.21 | 0.71 | 0.93 | 0.44 | 0.83 | 1.15 | 1.07 | 1.15 | 1.15 | 12.0 |
| E | 48.0 | 0.72 | 0.13 | 0.68 | 0.75 | 0.50 | 0.70 | 1.13 | 0.89 | 1.02 | 1.13 | 45.0 |
| S | 37.0 | 0.77 | 0.11 | 0.74 | 0.81 | 0.49 | 0.79 | 1.03 | 0.92 | 0.97 | 1.03 | 36.0 |
| SC | 22.0 | 0.69 | 0.16 | 0.62 | 0.76 | 0.36 | 0.70 | 1.20 | 0.80 | 0.84 | 1.20 | 21.0 |
| NE | 38.0 | 0.71 | 0.20 | 0.64 | 0.77 | 0.41 | 0.70 | 1.16 | 0.98 | 1.13 | 1.16 | 35.0 |
| W | 29.0 | 0.70 | 0.14 | 0.65 | 0.75 | 0.39 | 0.68 | 0.93 | 0.90 | 0.91 | 0.93 | 29.0 |
| YG | 24.0 | 0.65 | 0.13 | 0.60 | 0.70 | 0.45 | 0.62 | 0.98 | 0.81 | 0.82 | 0.98 | 24.0 |
| C | 55.0 | 0.82 | 0.14 | 0.78 | 0.85 | 0.54 | 0.82 | 1.24 | 0.98 | 1.12 | 1.24 | 50.0 |
| Q | 11.0 | 0.67 | 0.20 | 0.53 | 0.81 | 0.35 | 0.63 | 1.10 | 0.88 | 1.10 | 1.10 | 10.0 |
| M | 11.0 | 0.61 | 0.26 | 0.44 | 0.78 | 0.23 | 0.59 | 1.00 | 0.92 | 1.00 | 1.00 | 11.0 |
| CH | 362.0 | 0.76 | 0.19 | 0.74 | 0.78 | 0.23 | 0.76 | 1.53 | 0.99 | 1.12 | 1.35 | 328.0 |

**S42 Table. Absolute change of CO between 2015 and 2019.**

| Absolute change CO between 2015 and 2019 | Total cities | Mean | S. D. | Lower 95% CI of Mean | Upper 95% CI of Mean | Min | Median | Max | P90 | P95 | P99 | Number of cities at < 0 | Number of cities at >0 |
| --- | --- | --- | --- | --- | --- | --- | --- | --- | --- | --- | --- | --- | --- |
| N | 71.0 | -0.51 | 0.38 | -0.60 | -0.42 | -1.87 | -0.48 | 0.17 | -0.07 | 0.08 | 0.17 | 66.00 | 5.00 |
| X | 16.0 | -0.43 | 0.21 | -0.54 | -0.32 | -0.76 | -0.40 | -0.09 | -0.16 | -0.09 | -0.09 | 16.00 | 0.00 |
| E | 48.0 | -0.22 | 0.18 | -0.27 | -0.17 | -1.11 | -0.20 | 0.01 | -0.07 | -0.03 | 0.01 | 47.00 | 1.00 |
| S | 37.0 | -0.19 | 0.10 | -0.23 | -0.16 | -0.48 | -0.19 | -0.01 | -0.06 | -0.03 | -0.01 | 37.00 | 0.00 |
| SC | 22.0 | -0.22 | 0.14 | -0.28 | -0.16 | -0.48 | -0.24 | 0.03 | -0.01 | 0.02 | 0.03 | 20.00 | 2.00 |
| NE | 38.0 | -0.29 | 0.20 | -0.36 | -0.22 | -0.88 | -0.28 | 0.16 | -0.04 | 0.08 | 0.16 | 35.00 | 3.00 |
| W | 29.0 | -0.47 | 0.29 | -0.58 | -0.36 | -1.21 | -0.41 | 0.08 | -0.17 | -0.04 | 0.08 | 28.00 | 1.00 |
| YG | 24.0 | -0.15 | 0.15 | -0.21 | -0.08 | -0.44 | -0.10 | 0.09 | 0.04 | 0.08 | 0.09 | 20.00 | 4.00 |
| C | 55.0 | -0.26 | 0.23 | -0.32 | -0.20 | -0.84 | -0.22 | 0.35 | -0.04 | 0.11 | 0.35 | 51.00 | 4.00 |
| Q | 11.0 | -0.30 | 0.43 | -0.59 | -0.01 | -1.35 | -0.16 | 0.18 | 0.02 | 0.18 | 0.18 | 9.00 | 2.00 |
| M | 11.0 | -0.26 | 0.18 | -0.38 | -0.14 | -0.45 | -0.32 | 0.01 | 0.00 | 0.01 | 0.01 | 10.00 | 1.00 |
| CH | 362.0 | -0.31 | 0.28 | -0.34 | -0.29 | -1.87 | -0.25 | 0.35 | -0.04 | 0.02 | 0.17 | 339.00 | 23.00 |

**S43 Table. Relative change of CO between 2015 and 2019.**

| Relative change CO between 2015 and 2019 | Total cities | Mean | S. D. | Lower 95% CI of Mean | Upper 95% CI of Mean | Min | Median | Max | P90 | P95 | P99 |
| --- | --- | --- | --- | --- | --- | --- | --- | --- | --- | --- | --- |
| N | 71.0 | -32.0 | 19.3 | -36.6 | -27.4 | -67.8 | -35.3 | 28.6 | -7.3 | 7.7 | 28.6 |
| X | 16.0 | -33.5 | 14.7 | -41.3 | -25.6 | -62.1 | -29.8 | -9.6 | -17.5 | -9.6 | -9.6 |
| E | 48.0 | -21.8 | 11.9 | -25.3 | -18.4 | -60.7 | -21.6 | 1.3 | -8.7 | -2.7 | 1.3 |
| S | 37.0 | -19.5 | 8.6 | -22.4 | -16.6 | -38.8 | -18.9 | -1.5 | -6.9 | -3.2 | -1.5 |
| SC | 22.0 | -22.7 | 12.8 | -28.4 | -17.0 | -44.5 | -25.3 | 3.7 | -0.7 | 2.3 | 3.7 |
| NE | 38.0 | -26.4 | 16.0 | -31.7 | -21.1 | -48.5 | -29.4 | 20.2 | -7.6 | 14.1 | 20.2 |
| W | 29.0 | -37.2 | 15.8 | -43.2 | -31.2 | -67.9 | -40.1 | 10.0 | -17.9 | -5.2 | 10.0 |
| YG | 24.0 | -16.7 | 16.5 | -23.7 | -9.8 | -42.0 | -14.3 | 12.4 | 7.4 | 10.8 | 12.4 |
| C | 55.0 | -18.8 | 29.1 | -26.7 | -11.0 | -50.8 | -20.9 | 164.1 | -5.0 | 10.2 | 164.1 |
| Q | 11.0 | -21.9 | 29.3 | -41.5 | -2.2 | -68.2 | -21.3 | 36.5 | 2.1 | 36.5 | 36.5 |
| M | 11.0 | -30.0 | 21.0 | -44.1 | -15.9 | -66.0 | -27.9 | 1.8 | -0.6 | 1.8 | 1.8 |
| CH | 362.0 | -25.3 | 19.6 | -27.3 | -23.3 | -68.2 | -25.7 | 164.1 | -5.7 | 2.3 | 28.6 |

**S44 Table. Annual average absolute change of CO from 2015 to 2019.**

| Annual average absolute change of CO | Total cities | Mean | S. D. | Lower 95% CI of Mean | Upper 95% CI of Mean | Min | Median | Max | P90 | P95 | P99 | Number of cities at < 0 | Number of cities at >0 |
| --- | --- | --- | --- | --- | --- | --- | --- | --- | --- | --- | --- | --- | --- |
| N | 71.0 | -0.13 | 0.09 | -0.15 | -0.10 | -0.47 | -0.12 | 0.04 | -0.02 | 0.02 | 0.04 | 66.00 | 5.00 |
| X | 16.0 | -0.11 | 0.05 | -0.14 | -0.08 | -0.19 | -0.10 | -0.02 | -0.04 | -0.02 | -0.02 | 16.00 | 0.00 |
| E | 48.0 | -0.05 | 0.05 | -0.07 | -0.04 | -0.28 | -0.05 | 0.00 | -0.02 | -0.01 | 0.00 | 47.00 | 1.00 |
| S | 37.0 | -0.05 | 0.03 | -0.06 | -0.04 | -0.12 | -0.05 | 0.00 | -0.01 | -0.01 | 0.00 | 37.00 | 0.00 |
| SC | 22.0 | -0.06 | 0.03 | -0.07 | -0.04 | -0.12 | -0.06 | 0.01 | 0.00 | 0.00 | 0.01 | 20.00 | 2.00 |
| NE | 38.0 | -0.07 | 0.05 | -0.09 | -0.06 | -0.22 | -0.07 | 0.04 | -0.01 | 0.02 | 0.04 | 35.00 | 3.00 |
| W | 29.0 | -0.12 | 0.07 | -0.14 | -0.09 | -0.30 | -0.10 | 0.02 | -0.04 | -0.01 | 0.02 | 28.00 | 1.00 |
| YG | 24.0 | -0.04 | 0.04 | -0.05 | -0.02 | -0.11 | -0.03 | 0.02 | 0.01 | 0.02 | 0.02 | 20.00 | 4.00 |
| C | 55.0 | -0.06 | 0.06 | -0.08 | -0.05 | -0.21 | -0.06 | 0.09 | -0.01 | 0.03 | 0.09 | 51.00 | 4.00 |
| Q | 11.0 | -0.08 | 0.11 | -0.15 | 0.00 | -0.34 | -0.04 | 0.04 | 0.00 | 0.04 | 0.04 | 9.00 | 2.00 |
| M | 11.0 | -0.06 | 0.04 | -0.09 | -0.04 | -0.11 | -0.08 | 0.00 | 0.00 | 0.00 | 0.00 | 10.00 | 1.00 |
| CH | 362.0 | -0.08 | 0.07 | -0.09 | -0.07 | -0.47 | -0.06 | 0.09 | -0.01 | 0.00 | 0.04 | 339.00 | 23.00 |

**S45 Table. Annual average relative change of CO from 2015 to 2019.**

| Annual average relative change CO | Total cities | Mean | Standard Deviation | Lower 95% CI of Mean | Upper 95% CI of Mean | Min | Median | Max | P90 | P95 | P99 |
| --- | --- | --- | --- | --- | --- | --- | --- | --- | --- | --- | --- |
| N | 71.0 | -9.2 | 6.2 | -10.7 | -7.8 | -22.7 | -9.5 | 6.5 | -1.2 | 3.0 | 6.5 |
| X | 16.0 | -8.5 | 4.6 | -11.0 | -6.1 | -16.3 | -7.5 | -0.3 | -3.1 | -0.3 | -0.3 |
| E | 48.0 | -5.8 | 3.5 | -6.8 | -4.8 | -16.4 | -5.5 | 0.6 | -1.7 | -0.5 | 0.6 |
| S | 37.0 | -4.8 | 3.1 | -5.8 | -3.8 | -10.6 | -4.9 | 6.4 | -0.7 | 0.0 | 6.4 |
| SC | 22.0 | -5.7 | 4.1 | -7.5 | -3.9 | -13.5 | -6.8 | 2.8 | 0.7 | 1.0 | 2.8 |
| NE | 38.0 | -6.8 | 4.8 | -8.4 | -5.3 | -13.9 | -7.8 | 6.2 | -0.9 | 5.5 | 6.2 |
| W | 29.0 | -10.6 | 5.5 | -12.7 | -8.6 | -24.4 | -10.4 | 2.8 | -4.7 | -0.9 | 2.8 |
| YG | 24.0 | -3.8 | 5.0 | -5.9 | -1.7 | -11.6 | -3.5 | 7.8 | 2.7 | 4.1 | 7.8 |
| C | 55.0 | -4.9 | 7.5 | -6.9 | -2.9 | -15.7 | -5.1 | 38.8 | -0.4 | 3.3 | 38.8 |
| Q | 11.0 | -3.7 | 9.6 | -10.1 | 2.7 | -19.9 | -4.3 | 10.8 | 10.0 | 10.8 | 10.8 |
| M | 11.0 | -8.3 | 6.6 | -12.8 | -3.8 | -21.5 | -7.6 | 0.9 | 0.7 | 0.9 | 0.9 |
| CH | 362.0 | -6.7 | 5.9 | -7.3 | -6.1 | -24.4 | -6.5 | 38.8 | -0.4 | 2.7 | 8.2 |

**S46 Table. Statistical analysis of the NO_2_ (unit: μg/m^3^) in China in 2015.**

| NO_2_ in 2015 | Total cities | Mean | Standard Deviation | Lower 95% CI of Mean | Upper 95% CI of Mean | Min | Median | Max | P90 | P95 | P99 | Number of cities at < 40 μg/m^3^ |
| --- | --- | --- | --- | --- | --- | --- | --- | --- | --- | --- | --- | --- |
| N | 71.0 | 38.0 | 10.0 | 35.6 | 40.4 | 12.7 | 38.3 | 61.0 | 49.5 | 54.9 | 61.0 | 39.0 |
| X | 16.0 | 26.3 | 10.5 | 20.7 | 31.9 | 12.2 | 29.2 | 50.2 | 35.2 | 50.2 | 50.2 | 15.0 |
| E | 48.0 | 34.8 | 9.9 | 32.0 | 37.7 | 12.3 | 36.5 | 51.5 | 45.1 | 49.6 | 51.5 | 28.0 |
| S | 37.0 | 22.6 | 7.9 | 19.9 | 25.2 | 12.0 | 20.9 | 44.2 | 33.4 | 40.1 | 44.2 | 35.0 |
| SC | 22.0 | 28.1 | 8.4 | 24.4 | 31.8 | 9.0 | 28.8 | 48.7 | 37.6 | 42.8 | 48.7 | 20.0 |
| NE | 38.0 | 28.3 | 8.5 | 25.5 | 31.1 | 14.8 | 27.9 | 48.7 | 40.2 | 45.7 | 48.7 | 34.0 |
| W | 29.0 | 29.3 | 8.6 | 26.1 | 32.6 | 16.6 | 27.8 | 47.0 | 42.4 | 44.4 | 47.0 | 25.0 |
| YG | 24.0 | 18.0 | 5.5 | 15.7 | 20.3 | 7.5 | 17.1 | 28.3 | 25.7 | 27.1 | 28.3 | 24.0 |
| C | 55.0 | 27.3 | 7.7 | 25.2 | 29.3 | 13.3 | 27.2 | 52.9 | 36.1 | 37.0 | 52.9 | 53.0 |
| Q | 11.0 | 19.9 | 9.0 | 13.8 | 26.0 | 10.0 | 17.6 | 39.2 | 32.9 | 39.2 | 39.2 | 11.0 |
| M | 11.0 | 24.6 | 9.7 | 18.1 | 31.1 | 9.6 | 24.4 | 40.5 | 38.3 | 40.5 | 40.5 | 10.0 |
| CH | 362.0 | 29.2 | 10.6 | 28.2 | 30.3 | 7.5 | 28.4 | 61.0 | 44.1 | 47.9 | 54.9 | 294.0 |

**S47 Table. Statistical analysis of the NO_2_ (unit: μg/m^3^) in China in 2016.**

| NO_2_ in 2016 | Total cities | Mean | Standard Deviation | Lower 95% CI of Mean | Upper 95% CI of Mean | Min | Median | Max | P90 | P95 | P99 |
| --- | --- | --- | --- | --- | --- | --- | --- | --- | --- | --- | --- |
| N | 71.0 | 38.4 | 9.8 | 36.1 | 40.7 | 16.4 | 38.1 | 60.7 | 51.6 | 54.1 | 60.7 |
| X | 16.0 | 28.1 | 10.7 | 22.4 | 33.8 | 13.2 | 29.8 | 51.5 | 38.5 | 51.5 | 51.5 |
| E | 48.0 | 33.0 | 9.1 | 30.4 | 35.7 | 13.9 | 33.5 | 53.8 | 43.9 | 48.2 | 53.8 |
| S | 37.0 | 23.1 | 8.1 | 20.4 | 25.8 | 12.1 | 21.7 | 43.6 | 34.1 | 41.0 | 43.6 |
| SC | 22.0 | 29.0 | 8.6 | 25.2 | 32.7 | 11.8 | 27.7 | 49.7 | 39.5 | 44.3 | 49.7 |
| NE | 38.0 | 26.5 | 6.7 | 24.3 | 28.8 | 13.0 | 26.8 | 41.8 | 36.5 | 38.6 | 41.8 |
| W | 29.0 | 30.4 | 9.7 | 26.8 | 34.1 | 16.4 | 27.7 | 51.1 | 47.3 | 49.0 | 51.1 |
| YG | 24.0 | 18.3 | 5.5 | 16.0 | 20.6 | 10.5 | 17.7 | 31.3 | 25.2 | 26.4 | 31.3 |
| C | 55.0 | 28.4 | 8.1 | 26.2 | 30.6 | 14.0 | 28.0 | 44.1 | 38.9 | 42.1 | 44.1 |
| Q | 11.0 | 20.7 | 9.7 | 14.2 | 27.2 | 12.4 | 16.9 | 41.1 | 36.8 | 41.1 | 41.1 |
| M | 11.0 | 24.3 | 9.8 | 17.8 | 30.9 | 11.1 | 22.9 | 41.3 | 38.9 | 41.3 | 41.3 |
| CH | 362.0 | 29.4 | 10.4 | 28.3 | 30.5 | 10.5 | 28.4 | 60.7 | 43.2 | 47.8 | 54.1 |

**S48 Table. Statistical analysis of the NO_2_ (unit: μg/m^3^) in China in 2017.**

| NO_2_ in 2017 | Total cities | Mean | Standard Deviation | Lower 95% CI of Mean | Upper 95% CI of Mean | Min | Median | Max | P90 | P95 | P99 |
| --- | --- | --- | --- | --- | --- | --- | --- | --- | --- | --- | --- |
| N | 71.0 | 38.4 | 9.3 | 36.2 | 40.6 | 14.9 | 39.3 | 58.4 | 49.9 | 51.9 | 58.4 |
| X | 16.0 | 30.0 | 11.1 | 24.1 | 35.9 | 13.2 | 30.5 | 49.0 | 43.1 | 49.0 | 49.0 |
| E | 48.0 | 34.4 | 9.1 | 31.7 | 37.0 | 16.0 | 34.2 | 54.8 | 45.7 | 47.3 | 54.8 |
| S | 37.0 | 24.8 | 9.2 | 21.7 | 27.9 | 11.1 | 24.5 | 49.0 | 38.2 | 44.1 | 49.0 |
| SC | 22.0 | 30.0 | 8.4 | 26.3 | 33.7 | 8.2 | 30.5 | 47.8 | 38.2 | 44.2 | 47.8 |
| NE | 38.0 | 26.5 | 7.1 | 24.2 | 28.9 | 10.5 | 26.5 | 42.6 | 37.3 | 38.8 | 42.6 |
| W | 29.0 | 31.9 | 11.1 | 27.7 | 36.2 | 15.2 | 27.9 | 57.9 | 50.9 | 53.4 | 57.9 |
| YG | 24.0 | 19.2 | 4.8 | 17.1 | 21.2 | 12.0 | 18.6 | 29.2 | 24.9 | 28.2 | 29.2 |
| C | 55.0 | 29.6 | 8.9 | 27.2 | 32.0 | 15.4 | 26.8 | 49.8 | 40.0 | 47.2 | 49.8 |
| Q | 11.0 | 20.6 | 7.9 | 15.3 | 25.9 | 13.8 | 17.1 | 36.4 | 34.4 | 36.4 | 36.4 |
| M | 11.0 | 25.7 | 10.6 | 18.6 | 32.8 | 12.2 | 26.8 | 45.0 | 41.8 | 45.0 | 45.0 |
| CH | 362.0 | 30.3 | 10.5 | 29.2 | 31.4 | 8.2 | 29.2 | 58.4 | 45.0 | 48.5 | 54.8 |

**S49 Table. Statistical analysis of the NO_2_ (unit: μg/m^3^) in China in 2018.**

| NO_2_ in 2018 | Total cities | Mean | Standard Deviation | Lower 95% CI of Mean | Upper 95% CI of Mean | Min | Median | Max | P90 | P95 | P99 |
| --- | --- | --- | --- | --- | --- | --- | --- | --- | --- | --- | --- |
| N | 71.0 | 35.1 | 8.0 | 33.2 | 37.0 | 14.2 | 34.4 | 54.1 | 44.7 | 47.0 | 54.1 |
| X | 16.0 | 25.9 | 9.7 | 20.7 | 31.1 | 10.3 | 29.0 | 44.0 | 33.7 | 44.0 | 44.0 |
| E | 48.0 | 32.5 | 9.0 | 29.9 | 35.1 | 13.9 | 33.9 | 54.4 | 42.3 | 44.7 | 54.4 |
| S | 37.0 | 23.2 | 8.4 | 20.4 | 26.0 | 10.9 | 20.9 | 45.9 | 35.6 | 39.6 | 45.9 |
| SC | 22.0 | 27.8 | 8.2 | 24.2 | 31.5 | 8.0 | 29.2 | 42.2 | 37.2 | 41.2 | 42.2 |
| NE | 38.0 | 23.6 | 6.5 | 21.4 | 25.8 | 10.9 | 24.1 | 36.1 | 32.5 | 34.7 | 36.1 |
| W | 29.0 | 29.1 | 10.1 | 25.3 | 32.9 | 15.0 | 25.2 | 53.1 | 46.2 | 46.5 | 53.1 |
| YG | 24.0 | 17.4 | 5.0 | 15.3 | 19.6 | 9.0 | 17.6 | 29.7 | 22.7 | 25.5 | 29.7 |
| C | 55.0 | 26.9 | 7.4 | 24.9 | 28.9 | 13.4 | 26.2 | 42.9 | 38.7 | 40.0 | 42.9 |
| Q | 11.0 | 19.1 | 8.6 | 13.2 | 24.9 | 9.3 | 16.3 | 38.0 | 32.5 | 38.0 | 38.0 |
| M | 11.0 | 22.6 | 10.1 | 15.8 | 29.4 | 10.6 | 24.3 | 39.5 | 37.6 | 39.5 | 39.5 |
| CH | 362.0 | 27.7 | 9.6 | 26.7 | 28.7 | 8.0 | 27.2 | 54.4 | 41.1 | 44.0 | 49.5 |

**S50 Table. Statistical analysis of the NO_2_ (unit: μg/m^3^) in China in 2019.**

| NO_2_ in 2019 | Total cities | Mean | S. D. | Lower 95% CI of Mean | Upper 95% CI of Mean | Min | Median | Max | P90 | P95 | P99 | Number of cities at < 40 μg/m^3^ |
| --- | --- | --- | --- | --- | --- | --- | --- | --- | --- | --- | --- | --- |
| N | 71.0 | 33.1 | 7.0 | 31.5 | 34.8 | 17.7 | 33.4 | 51.5 | 41.8 | 43.9 | 51.5 | 59.0 |
| X | 16.0 | 25.1 | 8.4 | 20.6 | 29.6 | 9.3 | 25.9 | 40.4 | 35.0 | 40.4 | 40.4 | 15.0 |
| E | 48.0 | 28.9 | 8.0 | 26.6 | 31.2 | 12.5 | 30.2 | 43.9 | 39.9 | 40.1 | 43.9 | 44.0 |
| S | 37.0 | 20.8 | 7.4 | 18.3 | 23.3 | 8.1 | 20.7 | 40.4 | 30.7 | 38.0 | 40.4 | 36.0 |
| SC | 22.0 | 25.5 | 7.8 | 22.0 | 29.0 | 6.5 | 24.5 | 41.4 | 36.7 | 37.4 | 41.4 | 21.0 |
| NE | 38.0 | 21.5 | 6.8 | 19.3 | 23.8 | 9.7 | 20.8 | 33.5 | 30.8 | 31.8 | 33.5 | 38.0 |
| W | 29.0 | 27.3 | 8.9 | 23.9 | 30.7 | 13.8 | 24.3 | 46.7 | 40.4 | 45.9 | 46.7 | 25.0 |
| YG | 24.0 | 15.7 | 4.5 | 13.8 | 17.6 | 7.7 | 15.9 | 26.7 | 21.5 | 23.3 | 26.7 | 24.0 |
| C | 55.0 | 24.6 | 6.4 | 22.9 | 26.4 | 12.3 | 24.0 | 40.0 | 32.4 | 38.2 | 40.0 | 54.0 |
| Q | 11.0 | 17.8 | 9.0 | 11.7 | 23.8 | 9.2 | 13.5 | 38.5 | 30.5 | 38.5 | 38.5 | 11.0 |
| M | 11.0 | 21.6 | 9.8 | 15.0 | 28.2 | 9.5 | 23.7 | 36.9 | 35.2 | 36.9 | 36.9 | 11.0 |
| CH | 362.0 | 25.5 | 8.9 | 24.6 | 26.5 | 6.5 | 24.8 | 51.5 | 38.0 | 40.4 | 45.9 | 338.0 |

**S51 Table. Absolute change of NO_2_ between 2015 and 2019.**

| Absolute change of NO_2_ between 2015 and 2019 | Total cities | Mean | S. D. | Lower 95% CI of Mean | Upper 95% CI of Mean | Min | Median | Max | P90 | P95 | P99 | Number of cities at < 0 | Number of cities at > 0 |
| --- | --- | --- | --- | --- | --- | --- | --- | --- | --- | --- | --- | --- | --- |
| N | 71.0 | -4.9 | 6.9 | -6.5 | -3.3 | -21.0 | -4.6 | 17.9 | 3.2 | 6.1 | 17.9 | 53.0 | 18.0 |
| X | 16.0 | -1.2 | 5.1 | -4.0 | 1.5 | -9.8 | -1.3 | 11.3 | 4.7 | 11.3 | 11.3 | 10.0 | 6.0 |
| E | 48.0 | -5.9 | 5.4 | -7.5 | -4.4 | -22.1 | -6.1 | 5.7 | 0.3 | 3.8 | 5.7 | 43.0 | 5.0 |
| S | 37.0 | -1.8 | 2.6 | -2.6 | -0.9 | -9.0 | -1.9 | 5.2 | 1.6 | 3.0 | 5.2 | 29.0 | 8.0 |
| SC | 22.0 | -2.6 | 4.0 | -4.3 | -0.8 | -12.0 | -2.6 | 5.1 | 2.8 | 3.7 | 5.1 | 18.0 | 4.0 |
| NE | 38.0 | -6.7 | 4.2 | -8.1 | -5.4 | -18.3 | -5.8 | 1.9 | -3.1 | -0.2 | 1.9 | 37.0 | 1.0 |
| W | 29.0 | -2.0 | 5.8 | -4.2 | 0.2 | -15.7 | -3.3 | 11.3 | 6.7 | 7.6 | 11.3 | 21.0 | 8.0 |
| YG | 24.0 | -2.3 | 3.9 | -4.0 | -0.6 | -9.6 | -2.3 | 9.3 | 1.6 | 4.7 | 9.3 | 21.0 | 3.0 |
| C | 55.0 | -2.6 | 5.1 | -4.0 | -1.3 | -23.0 | -2.8 | 10.3 | 3.7 | 6.7 | 10.3 | 43.0 | 12.0 |
| Q | 11.0 | -2.2 | 5.2 | -5.7 | 1.4 | -11.7 | -1.6 | 3.2 | 3.0 | 3.2 | 3.2 | 7.0 | 4.0 |
| M | 11.0 | -3.0 | 4.1 | -5.7 | -0.2 | -10.0 | -2.2 | 2.0 | 1.4 | 2.0 | 2.0 | 7.0 | 4.0 |
| CH | 362.0 | -3.7 | 5.4 | -4.3 | -3.1 | -23.0 | -3.4 | 17.9 | 2.8 | 4.7 | 10.3 | 289.0 | 73.0 |

**S52 Table. Relative change of NO_2_ between 2015 and 2019.**

| Relative change of NO_2_ between 2015 and 2019 | Total cities | Mean | S. D. | Lower 95% CI of Mean | Upper 95% CI of Mean | Min | Median | Max | P90 | P95 | P99 |
| --- | --- | --- | --- | --- | --- | --- | --- | --- | --- | --- | --- |
| N | 71.0 | -9.9 | 18.4 | -14.2 | -5.6 | -36.8 | -13.0 | 69.1 | 11.4 | 23.3 | 69.1 |
| X | 16.0 | -0.1 | 28.2 | -15.1 | 15.0 | -40.7 | -6.8 | 85.0 | 25.5 | 85.0 | 85.0 |
| E | 48.0 | -15.6 | 13.7 | -19.6 | -11.7 | -44.5 | -16.8 | 26.2 | 2.3 | 12.5 | 26.2 |
| S | 37.0 | -7.3 | 13.9 | -11.9 | -2.6 | -38.9 | -9.0 | 43.6 | 7.6 | 20.5 | 43.6 |
| SC | 22.0 | -9.1 | 14.1 | -15.3 | -2.8 | -28.5 | -12.2 | 27.5 | 9.9 | 10.2 | 27.5 |
| NE | 38.0 | -23.5 | 12.5 | -27.6 | -19.4 | -48.3 | -24.9 | 10.0 | -10.4 | -1.0 | 10.0 |
| W | 29.0 | -5.5 | 20.7 | -13.3 | 2.4 | -43.9 | -9.9 | 38.6 | 31.8 | 37.7 | 38.6 |
| YG | 24.0 | -8.5 | 32.4 | -22.2 | 5.2 | -45.4 | -13.5 | 123.6 | 8.8 | 35.0 | 123.6 |
| C | 55.0 | -7.9 | 16.8 | -12.4 | -3.3 | -43.5 | -11.3 | 53.6 | 13.8 | 27.6 | 53.6 |
| Q | 11.0 | -7.1 | 27.9 | -25.8 | 11.6 | -56.0 | -7.3 | 28.1 | 23.1 | 28.1 | 28.1 |
| M | 11.0 | -12.2 | 17.9 | -24.3 | -0.2 | -45.8 | -13.3 | 7.6 | 7.1 | 7.6 | 7.6 |
| CH | 362.0 | -10.6 | 19.3 | -12.6 | -8.6 | -56.0 | -13.1 | 123.6 | 10.4 | 24.5 | 53.6 |

**S53 Table. Annual average absolute change of NO_2_ from 2015 to 2019.**

| Annual average absolute change of NO_2_ | Total cities | Mean | S. D. | Lower 95% CI of Mean | Upper 95% CI of Mean | Min | Median | Max | P90 | P95 | P99 | Number of cities at < 0 | Number of cities at > 0 |
| --- | --- | --- | --- | --- | --- | --- | --- | --- | --- | --- | --- | --- | --- |
| N | 71.0 | -4.9 | 6.9 | -6.5 | -3.3 | -21.0 | -4.6 | 17.9 | 3.2 | 6.1 | 17.9 | 53.0 | 18.0 |
| X | 16.0 | -1.2 | 5.2 | -4.0 | 1.5 | -9.8 | -1.3 | 11.3 | 4.7 | 11.3 | 11.3 | 10.0 | 6.0 |
| E | 48.0 | -5.9 | 5.4 | -7.5 | -4.4 | -22.1 | -6.1 | 5.7 | 0.3 | 3.8 | 5.7 | 43.0 | 5.0 |
| S | 37.0 | -1.8 | 2.6 | -2.6 | -0.9 | -9.0 | -1.9 | 5.2 | 1.6 | 3.0 | 5.2 | 29.0 | 8.0 |
| SC | 22.0 | -2.6 | 4.0 | -4.3 | -0.8 | -12.0 | -2.5 | 5.1 | 2.8 | 3.7 | 5.1 | 18.0 | 4.0 |
| NE | 38.0 | -6.7 | 4.1 | -8.1 | -5.4 | -18.3 | -5.7 | 1.9 | -3.1 | -0.2 | 1.9 | 37.0 | 1.0 |
| W | 29.0 | -2.0 | 5.8 | -4.2 | 0.2 | -15.7 | -3.3 | 11.3 | 6.7 | 7.6 | 11.3 | 21.0 | 8.0 |
| YG | 24.0 | -2.3 | 3.9 | -4.0 | -0.6 | -9.6 | -2.3 | 9.3 | 1.6 | 4.7 | 9.3 | 21.0 | 3.0 |
| C | 55.0 | -2.6 | 5.1 | -4.0 | -1.3 | -23.0 | -2.8 | 10.3 | 3.7 | 6.7 | 10.3 | 43.0 | 12.0 |
| Q | 11.0 | -2.1 | 5.2 | -5.7 | 1.4 | -11.7 | -1.6 | 3.2 | 3.0 | 3.2 | 3.2 | 7.0 | 4.0 |
| M | 11.0 | -2.9 | 4.1 | -5.7 | -0.2 | -10.0 | -2.2 | 2.0 | 1.4 | 2.0 | 2.0 | 7.0 | 4.0 |
| CH | 362.0 | -3.7 | 5.4 | -4.3 | -3.1 | -23.0 | -3.4 | 17.9 | 2.8 | 4.7 | 10.3 | 289.0 | 73.0 |

**S54 Table. Annual average relative change of NO_2_ from 2015 to 2019.**

| Annual average relative change of NO_2_ | Total cities | Mean | Standard Deviation | Lower 95% CI of Mean | Upper 95% CI of Mean | Min | Median | Max | P90 | P95 | P99 |
| --- | --- | --- | --- | --- | --- | --- | --- | --- | --- | --- | --- |
| N | 71.0 | -2.5 | 5.0 | -3.6 | -1.3 | -10.7 | -2.9 | 19.2 | 3.8 | 6.0 | 19.2 |
| X | 16.0 | 0.7 | 7.2 | -3.2 | 4.5 | -10.1 | -1.1 | 20.7 | 9.6 | 20.7 | 20.7 |
| E | 48.0 | -3.8 | 3.8 | -4.9 | -2.7 | -12.7 | -4.1 | 7.5 | 1.4 | 4.0 | 7.5 |
| S | 37.0 | -1.5 | 3.8 | -2.8 | -0.3 | -11.1 | -1.8 | 10.8 | 2.4 | 5.5 | 10.8 |
| SC | 22.0 | -1.6 | 3.9 | -3.4 | 0.1 | -7.1 | -2.3 | 7.6 | 3.6 | 3.7 | 7.6 |
| NE | 38.0 | -6.2 | 3.9 | -7.5 | -5.0 | -15.1 | -6.1 | 2.8 | -2.3 | 2.1 | 2.8 |
| W | 29.0 | -1.3 | 5.5 | -3.4 | 0.8 | -12.6 | -2.1 | 10.8 | 7.8 | 9.3 | 10.8 |
| YG | 24.0 | -1.9 | 7.4 | -5.0 | 1.2 | -11.3 | -3.1 | 25.7 | 2.4 | 8.0 | 25.7 |
| C | 55.0 | -1.5 | 4.8 | -2.8 | -0.2 | -12.6 | -2.3 | 16.8 | 4.4 | 7.8 | 16.8 |
| Q | 11.0 | -1.4 | 8.4 | -7.1 | 4.2 | -16.6 | -1.6 | 10.3 | 7.0 | 10.3 | 10.3 |
| M | 11.0 | -2.5 | 6.1 | -6.6 | 1.6 | -14.0 | -3.3 | 7.9 | 3.1 | 7.9 | 7.9 |
| CH | 362.0 | -2.5 | 5.3 | -3.0 | -1.9 | -16.6 | -3.0 | 25.7 | 3.8 | 7.0 | 16.8 |

**S55 Table. Statistical analysis of the O_3_ (unit: μg/m^3^) in China in 2015.**

| O_3_ in 2015 | Total cities | Mean | S. D. | Lower 95% CI of Mean | Upper 95% CI of Mean | Min | Median | Max | P90 | P95 | P99 | Number of cities at > 60 |
| --- | --- | --- | --- | --- | --- | --- | --- | --- | --- | --- | --- | --- |
| N | 71.0 | 58.6 | 10.5 | 56.2 | 61.1 | 35.2 | 60.2 | 79.0 | 70.8 | 76.0 | 79.0 | 37.0 |
| X | 16.0 | 55.4 | 14.7 | 47.6 | 63.3 | 28.1 | 57.4 | 74.1 | 70.0 | 74.1 | 74.1 | 7.0 |
| E | 48.0 | 58.8 | 10.9 | 55.6 | 61.9 | 27.6 | 58.4 | 81.5 | 74.4 | 76.0 | 81.5 | 21.0 |
| S | 37.0 | 52.4 | 9.5 | 49.2 | 55.5 | 28.6 | 53.8 | 70.2 | 65.6 | 69.8 | 70.2 | 7.0 |
| SC | 22.0 | 49.1 | 10.1 | 44.6 | 53.6 | 35.5 | 47.5 | 69.3 | 65.6 | 67.8 | 69.3 | 3.0 |
| NE | 38.0 | 56.0 | 8.6 | 53.2 | 58.8 | 41.2 | 56.2 | 80.1 | 69.6 | 73.5 | 80.1 | 10.0 |
| W | 29.0 | 57.1 | 11.3 | 52.8 | 61.4 | 36.6 | 55.2 | 81.2 | 74.6 | 76.1 | 81.2 | 12.0 |
| YG | 24.0 | 51.0 | 10.3 | 46.7 | 55.4 | 36.5 | 48.5 | 75.7 | 66.9 | 69.3 | 75.7 | 5.0 |
| C | 55.0 | 51.0 | 9.1 | 48.5 | 53.5 | 33.0 | 51.1 | 67.8 | 61.9 | 67.2 | 67.8 | 9.0 |
| Q | 11.0 | 72.7 | 16.7 | 61.5 | 83.9 | 51.8 | 72.8 | 97.3 | 93.6 | 97.3 | 97.3 | 7.0 |
| M | 11.0 | 63.2 | 15.2 | 53.0 | 73.4 | 44.3 | 59.8 | 94.1 | 76.2 | 94.1 | 94.1 | 5.0 |
| CH | 362.0 | 55.8 | 11.6 | 54.6 | 57.0 | 27.6 | 55.7 | 97.3 | 70.0 | 75.7 | 90.1 | 123.0 |

**S56 Table. Statistical analysis of the O_3_ (unit: μg/m^3^) in China in 2016.**

| O_3_ in 2016 | Total cities | Mean | Standard Deviation | Lower 95% CI of Mean | Upper 95% CI of Mean | Min | Median | Max | P90 | P95 | P99 |
| --- | --- | --- | --- | --- | --- | --- | --- | --- | --- | --- | --- |
| N | 71.0 | 62.5 | 9.0 | 60.4 | 64.6 | 34.3 | 63.5 | 78.1 | 74.5 | 76.2 | 78.1 |
| X | 16.0 | 59.0 | 12.1 | 52.5 | 65.4 | 34.7 | 59.3 | 76.2 | 73.1 | 76.2 | 76.2 |
| E | 48.0 | 59.9 | 10.0 | 57.0 | 62.8 | 34.9 | 59.7 | 82.2 | 73.9 | 76.6 | 82.2 |
| S | 37.0 | 52.6 | 7.1 | 50.2 | 55.0 | 35.9 | 51.5 | 68.4 | 62.7 | 67.7 | 68.4 |
| SC | 22.0 | 52.2 | 8.2 | 48.6 | 55.9 | 39.9 | 50.8 | 66.2 | 64.2 | 65.0 | 66.2 |
| NE | 38.0 | 58.5 | 10.6 | 55.0 | 62.0 | 41.9 | 58.0 | 81.3 | 74.0 | 79.5 | 81.3 |
| W | 29.0 | 62.3 | 9.3 | 58.7 | 65.8 | 48.8 | 61.7 | 81.0 | 75.5 | 78.1 | 81.0 |
| YG | 24.0 | 51.4 | 10.5 | 46.9 | 55.8 | 32.2 | 48.4 | 74.6 | 64.8 | 67.6 | 74.6 |
| C | 55.0 | 56.7 | 7.6 | 54.6 | 58.7 | 30.4 | 57.5 | 71.3 | 64.8 | 69.8 | 71.3 |
| Q | 11.0 | 65.1 | 21.1 | 50.9 | 79.3 | 30.1 | 73.9 | 95.4 | 83.1 | 95.4 | 95.4 |
| M | 11.0 | 67.3 | 11.4 | 59.6 | 75.0 | 54.5 | 65.4 | 94.8 | 79.7 | 94.8 | 94.8 |
| CH | 362.0 | 58.5 | 10.6 | 57.4 | 59.6 | 30.1 | 58.3 | 95.4 | 73.2 | 76.2 | 82.2 |

**S57 Table. Statistical analysis of the O_3_ (unit: μg/m^3^) in China in 2017.**

| O_3_ in 2017 | Total cities | Mean | S. D. | Lower 95% CI of Mean | Upper 95% CI of Mean | Min | Median | Max | P90 | P95 | P99 |
| --- | --- | --- | --- | --- | --- | --- | --- | --- | --- | --- | --- |
| N | 71.0 | 69.8 | 7.5 | 68.1 | 71.6 | 44.7 | 69.3 | 85.7 | 78.6 | 82.7 | 85.7 |
| X | 16.0 | 66.2 | 12.9 | 59.3 | 73.1 | 45.1 | 67.0 | 104.4 | 72.0 | 104.4 | 104.4 |
| E | 48.0 | 66.1 | 9.0 | 63.5 | 68.7 | 46.8 | 67.3 | 86.1 | 77.4 | 78.1 | 86.1 |
| S | 37.0 | 56.8 | 7.2 | 54.4 | 59.2 | 42.3 | 56.4 | 72.3 | 66.2 | 69.4 | 72.3 |
| SC | 22.0 | 55.5 | 8.3 | 51.8 | 59.1 | 42.3 | 54.0 | 72.2 | 66.7 | 66.8 | 72.2 |
| NE | 38.0 | 61.9 | 8.8 | 59.0 | 64.8 | 48.4 | 60.3 | 82.6 | 73.0 | 82.3 | 82.6 |
| W | 29.0 | 64.9 | 10.7 | 60.8 | 69.0 | 48.1 | 65.0 | 88.1 | 83.0 | 85.1 | 88.1 |
| YG | 24.0 | 54.0 | 10.3 | 49.7 | 58.4 | 37.6 | 53.4 | 72.1 | 70.4 | 71.8 | 72.1 |
| C | 55.0 | 60.7 | 9.1 | 58.2 | 63.1 | 37.4 | 60.6 | 80.3 | 72.4 | 74.1 | 80.3 |
| Q | 11.0 | 73.6 | 13.9 | 64.3 | 82.9 | 44.8 | 74.2 | 89.4 | 88.3 | 89.4 | 89.4 |
| M | 11.0 | 71.1 | 14.1 | 61.6 | 80.6 | 60.3 | 66.7 | 105.1 | 84.8 | 105.1 | 105.1 |
| CH | 362.0 | 63.5 | 10.7 | 62.4 | 64.6 | 37.4 | 63.5 | 105.1 | 76.5 | 81.9 | 88.3 |

**S58 Table. Statistical analysis of the O_3_ (unit: μg/m^3^) in China in 2018.**

| O_3_ in 2018 | Total cities | Mean | Standard Deviation | Lower 95% CI of Mean | Upper 95% CI of Mean | Min | Median | Max | P90 | P95 | P99 |
| --- | --- | --- | --- | --- | --- | --- | --- | --- | --- | --- | --- |
| N | 71.0 | 72.3 | 7.3 | 70.6 | 74.0 | 49.7 | 72.7 | 88.6 | 81.1 | 84.0 | 88.6 |
| X | 16.0 | 63.2 | 11.6 | 57.0 | 69.4 | 37.4 | 61.6 | 89.5 | 77.2 | 89.5 | 89.5 |
| E | 48.0 | 65.0 | 7.4 | 62.9 | 67.2 | 46.7 | 66.3 | 76.8 | 74.3 | 76.3 | 76.8 |
| S | 37.0 | 57.8 | 8.5 | 55.0 | 60.7 | 42.5 | 57.2 | 77.7 | 71.2 | 76.3 | 77.7 |
| SC | 22.0 | 58.5 | 7.9 | 55.0 | 62.0 | 45.1 | 58.0 | 75.8 | 68.0 | 68.2 | 75.8 |
| NE | 38.0 | 62.5 | 7.1 | 60.1 | 64.8 | 50.4 | 61.7 | 79.7 | 72.4 | 77.6 | 79.7 |
| W | 29.0 | 66.4 | 9.0 | 63.0 | 69.8 | 51.6 | 65.9 | 84.7 | 80.0 | 81.7 | 84.7 |
| YG | 24.0 | 56.0 | 9.1 | 52.2 | 59.9 | 37.9 | 54.7 | 71.3 | 69.2 | 70.5 | 71.3 |
| C | 55.0 | 63.0 | 9.9 | 60.4 | 65.7 | 33.7 | 64.2 | 78.9 | 77.0 | 78.0 | 78.9 |
| Q | 11.0 | 76.0 | 9.9 | 69.4 | 82.7 | 58.9 | 74.3 | 90.0 | 87.9 | 90.0 | 90.0 |
| M | 11.0 | 72.4 | 13.6 | 63.3 | 81.5 | 57.7 | 73.7 | 104.6 | 81.5 | 104.6 | 104.6 |
| CH | 362.0 | 64.7 | 10.2 | 63.7 | 65.8 | 33.7 | 64.4 | 104.6 | 77.6 | 80.3 | 88.6 |

**S59 Table. Statistical analysis of the O_3_ (unit: μg/m^3^) in China in 2019.**

| O_3_ in 2019 | Total cities | Mean | S. D. | Lower 95% CI of Mean | Upper 95% CI of Mean | Min | Median | Max | P90 | P95 | P99 | Number of cities at > 60 |
| --- | --- | --- | --- | --- | --- | --- | --- | --- | --- | --- | --- | --- |
| N | 71.0 | 73.6 | 8.3 | 71.6 | 75.6 | 42.6 | 74.6 | 92.0 | 82.3 | 84.7 | 92.0 | 67.0 |
| X | 16.0 | 65.1 | 6.7 | 61.5 | 68.7 | 55.7 | 65.4 | 79.0 | 74.6 | 79.0 | 79.0 | 12.0 |
| E | 48.0 | 65.1 | 8.8 | 62.5 | 67.6 | 30.8 | 67.8 | 76.9 | 72.3 | 73.1 | 76.9 | 38.0 |
| S | 37.0 | 56.5 | 8.6 | 53.6 | 59.3 | 40.7 | 54.5 | 76.6 | 66.7 | 76.3 | 76.6 | 13.0 |
| SC | 22.0 | 52.7 | 6.3 | 50.0 | 55.5 | 42.6 | 53.5 | 70.7 | 58.8 | 59.5 | 70.7 | 1.0 |
| NE | 38.0 | 60.0 | 8.1 | 57.4 | 62.7 | 43.0 | 59.2 | 78.5 | 71.7 | 77.6 | 78.5 | 19.0 |
| W | 29.0 | 64.7 | 10.8 | 60.6 | 68.8 | 46.8 | 63.8 | 85.6 | 84.0 | 84.2 | 85.6 | 19.0 |
| YG | 24.0 | 57.1 | 9.4 | 53.1 | 61.0 | 40.6 | 56.7 | 72.5 | 71.6 | 71.9 | 72.5 | 10.0 |
| C | 55.0 | 64.5 | 9.5 | 61.9 | 67.1 | 40.2 | 65.2 | 80.1 | 77.5 | 78.9 | 80.1 | 38.0 |
| Q | 11.0 | 76.8 | 14.1 | 67.3 | 86.3 | 59.3 | 75.6 | 103.6 | 95.7 | 103.6 | 103.6 | 9.0 |
| M | 11.0 | 69.6 | 13.7 | 60.4 | 78.8 | 54.0 | 66.6 | 102.4 | 78.6 | 102.4 | 102.4 | 9.0 |
| CH | 362.0 | 64.4 | 11.1 | 63.3 | 65.6 | 30.8 | 65.2 | 103.6 | 78.5 | 80.2 | 92.0 | 235.0 |

**S60 Table. Absolute change of O_3_ between 2015 and 2019.**

| Absolute change of O_3_ between 2015 and 2019 | Total cities | Mean | S. D. | Lower 95% CI of Mean | Upper 95% CI of Mean | Min | Median | Max | P90 | P95 | P99 | Number of cities at < 0 | Number of cities at > 0 |
| --- | --- | --- | --- | --- | --- | --- | --- | --- | --- | --- | --- | --- | --- |
| N | 71.0 | 15.0 | 10.7 | 12.4 | 17.5 | -10.9 | 14.3 | 49.3 | 28.9 | 31.4 | 49.3 | 6.0 | 65.0 |
| X | 16.0 | 9.6 | 13.2 | 2.6 | 16.7 | -6.4 | 9.4 | 40.8 | 32.4 | 40.8 | 40.8 | 5.0 | 11.0 |
| E | 48.0 | 6.3 | 8.3 | 3.9 | 8.7 | -21.0 | 7.4 | 28.3 | 16.1 | 17.6 | 28.3 | 9.0 | 39.0 |
| S | 37.0 | 4.1 | 6.7 | 1.9 | 6.3 | -11.3 | 4.9 | 17.6 | 11.2 | 16.1 | 17.6 | 12.0 | 25.0 |
| SC | 22.0 | 3.6 | 9.4 | -0.5 | 7.8 | -16.6 | 4.6 | 17.7 | 15.3 | 16.9 | 17.7 | 7.0 | 15.0 |
| NE | 38.0 | 4.0 | 6.9 | 1.8 | 6.3 | -7.2 | 4.5 | 30.5 | 11.0 | 17.1 | 30.5 | 11.0 | 27.0 |
| W | 29.0 | 7.6 | 8.1 | 4.5 | 10.6 | -7.6 | 9.3 | 32.3 | 15.9 | 17.7 | 32.3 | 5.0 | 24.0 |
| YG | 24.0 | 6.0 | 8.8 | 2.3 | 9.7 | -5.9 | 4.1 | 27.0 | 20.2 | 24.6 | 27.0 | 6.0 | 18.0 |
| C | 55.0 | 13.5 | 10.2 | 10.8 | 16.3 | -15.2 | 12.3 | 45.6 | 26.1 | 31.3 | 45.6 | 2.0 | 53.0 |
| Q | 11.0 | 4.1 | 6.5 | -0.3 | 8.4 | -4.7 | 4.7 | 16.8 | 10.6 | 16.8 | 16.8 | 3.0 | 8.0 |
| M | 11.0 | 6.4 | 7.5 | 1.4 | 11.4 | -5.7 | 7.2 | 20.7 | 16.1 | 20.7 | 20.7 | 1.0 | 10.0 |
| CH | 362.0 | 8.6 | 10.0 | 7.6 | 9.7 | -21.0 | 7.9 | 49.3 | 22.7 | 27.0 | 35.8 | 67.0 | 295.0 |

**S61 Table. Relative change of O_3_ between 2015 and 2019.**

| Relative change O_3_ between 2015 and 2019 | Total cities | Mean | S. D. | Lower 95% CI of Mean | Upper 95% CI of Mean | Min | Median | Max | P90 | P95 | P99 | Number of cities at < 0 | Number of cities at > 0 |
| --- | --- | --- | --- | --- | --- | --- | --- | --- | --- | --- | --- | --- | --- |
| N | 71.0 | 29.0 | 25.5 | 23.0 | 35.0 | -13.9 | 22.4 | 140.1 | 60.6 | 71.7 | 140.1 | 6.0 | 65.0 |
| X | 16.0 | 26.5 | 42.0 | 4.1 | 48.8 | -9.3 | 15.8 | 145.5 | 99.3 | 145.5 | 145.5 | 5.0 | 11.0 |
| E | 48.0 | 13.2 | 19.7 | 7.4 | 18.9 | -40.5 | 13.5 | 102.5 | 29.1 | 37.9 | 102.5 | 9.0 | 39.0 |
| S | 37.0 | 9.5 | 15.6 | 4.4 | 14.7 | -18.0 | 8.7 | 56.5 | 26.7 | 43.1 | 56.5 | 12.0 | 25.0 |
| SC | 22.0 | 10.7 | 20.7 | 1.6 | 19.9 | -26.5 | 9.3 | 48.9 | 38.7 | 44.1 | 48.9 | 7.0 | 15.0 |
| NE | 38.0 | 8.3 | 15.1 | 3.4 | 13.3 | -14.3 | 7.7 | 74.0 | 24.2 | 32.0 | 74.0 | 11.0 | 27.0 |
| W | 29.0 | 15.2 | 18.9 | 8.0 | 22.4 | -12.5 | 12.9 | 88.1 | 33.9 | 34.5 | 88.1 | 5.0 | 24.0 |
| YG | 24.0 | 14.1 | 21.4 | 5.0 | 23.1 | -12.7 | 8.1 | 67.0 | 47.6 | 66.0 | 67.0 | 6.0 | 18.0 |
| C | 55.0 | 29.5 | 26.3 | 22.4 | 36.6 | -27.3 | 23.3 | 138.0 | 58.3 | 70.6 | 138.0 | 2.0 | 53.0 |
| Q | 11.0 | 7.1 | 11.0 | -0.3 | 14.5 | -5.8 | 6.2 | 31.3 | 18.9 | 31.3 | 31.3 | 3.0 | 8.0 |
| M | 11.0 | 12.1 | 16.1 | 1.3 | 23.0 | -9.6 | 8.8 | 45.0 | 36.2 | 45.0 | 45.0 | 1.0 | 10.0 |
| CH | 362.0 | 18.3 | 23.9 | 15.9 | 20.8 | -40.5 | 14.6 | 145.5 | 47.6 | 62.1 | 102.5 | 67.0 | 295.0 |

**S62 Table. Annual average absolute change of O_3_ from 2015 to 2019.**

| Annual average absolute change of O_3_ | Total cities | Mean | S. D. | Lower 95% CI of Mean | Upper 95% CI of Mean | Min | Median | Max | P90 | P95 | P99 | Number of cities at < 0 | Number of cities at > 0 |
| --- | --- | --- | --- | --- | --- | --- | --- | --- | --- | --- | --- | --- | --- |
| N | 71.0 | 15.0 | 10.7 | 12.4 | 17.5 | -10.9 | 14.3 | 49.3 | 28.9 | 31.4 | 49.3 | 6.0 | 65.0 |
| X | 16.0 | 9.6 | 13.2 | 2.6 | 16.7 | -6.4 | 9.3 | 40.8 | 32.4 | 40.8 | 40.8 | 5.0 | 11.0 |
| E | 48.0 | 6.3 | 8.3 | 3.9 | 8.7 | -21.0 | 7.4 | 28.3 | 16.1 | 17.6 | 28.3 | 9.0 | 39.0 |
| S | 37.0 | 4.1 | 6.7 | 1.9 | 6.4 | -11.3 | 4.9 | 17.6 | 11.2 | 16.1 | 17.6 | 12.0 | 25.0 |
| SC | 22.0 | 3.6 | 9.4 | -0.5 | 7.8 | -16.6 | 4.5 | 17.7 | 15.3 | 16.9 | 17.7 | 7.0 | 15.0 |
| NE | 38.0 | 4.0 | 6.9 | 1.8 | 6.3 | -7.2 | 4.4 | 30.5 | 11.0 | 17.1 | 30.5 | 11.0 | 27.0 |
| W | 29.0 | 7.6 | 8.1 | 4.5 | 10.6 | -7.6 | 9.3 | 32.3 | 15.9 | 17.7 | 32.3 | 5.0 | 24.0 |
| YG | 24.0 | 6.0 | 8.8 | 2.3 | 9.7 | -5.9 | 4.0 | 27.0 | 20.2 | 24.6 | 27.0 | 6.0 | 18.0 |
| C | 55.0 | 13.5 | 10.2 | 10.8 | 16.3 | -15.2 | 12.3 | 45.6 | 26.1 | 31.3 | 45.6 | 2.0 | 53.0 |
| Q | 11.0 | 4.1 | 6.5 | -0.3 | 8.4 | -4.7 | 4.7 | 16.8 | 10.6 | 16.8 | 16.8 | 3.0 | 8.0 |
| M | 11.0 | 6.4 | 7.5 | 1.4 | 11.4 | -5.7 | 7.2 | 20.7 | 16.1 | 20.7 | 20.7 | 1.0 | 10.0 |
| CH | 362.0 | 8.6 | 10.0 | 7.6 | 9.7 | -21.0 | 7.9 | 49.3 | 22.7 | 27.0 | 35.8 | 67.0 | 295.0 |

**S63 Table. Annual average relative change of O_3_ from 2015 to 2019.**

| Annual average relative change of O_3_ | Total cities | Mean | Standard Deviation | Lower 95% CI of Mean | Upper 95% CI of Mean | Min | Median | Max | P90 | P95 | P99 |
| --- | --- | --- | --- | --- | --- | --- | --- | --- | --- | --- | --- |
| N | 71.0 | 6.7 | 5.1 | 5.5 | 7.9 | -3.6 | 5.4 | 27.0 | 12.8 | 14.7 | 27.0 |
| X | 16.0 | 7.0 | 9.2 | 2.1 | 11.9 | -2.0 | 5.7 | 32.1 | 22.6 | 32.1 | 32.1 |
| E | 48.0 | 4.0 | 5.3 | 2.5 | 5.5 | -8.8 | 3.5 | 28.7 | 8.6 | 11.5 | 28.7 |
| S | 37.0 | 2.4 | 3.7 | 1.2 | 3.7 | -4.7 | 2.3 | 13.3 | 6.2 | 9.6 | 13.3 |
| SC | 22.0 | 3.2 | 5.5 | 0.7 | 5.6 | -7.2 | 2.8 | 13.2 | 9.5 | 13.0 | 13.2 |
| NE | 38.0 | 2.5 | 3.6 | 1.3 | 3.6 | -3.3 | 2.3 | 16.0 | 7.4 | 9.3 | 16.0 |
| W | 29.0 | 4.2 | 4.6 | 2.4 | 6.0 | -3.0 | 4.4 | 19.8 | 8.4 | 12.0 | 19.8 |
| YG | 24.0 | 3.8 | 4.7 | 1.8 | 5.8 | -2.7 | 2.8 | 16.0 | 10.7 | 13.9 | 16.0 |
| C | 55.0 | 7.0 | 5.8 | 5.4 | 8.6 | -5.2 | 5.6 | 31.7 | 12.8 | 16.8 | 31.7 |
| Q | 11.0 | 5.8 | 7.5 | 0.8 | 10.9 | -1.1 | 4.9 | 25.0 | 12.0 | 25.0 | 25.0 |
| M | 11.0 | 3.2 | 4.0 | 0.5 | 5.8 | -2.2 | 2.3 | 11.3 | 9.3 | 11.3 | 11.3 |
| CH | 362.0 | 4.8 | 5.5 | 4.2 | 5.3 | -8.8 | 3.9 | 32.1 | 12.0 | 13.9 | 27.0 |
