# Supplementary material for: The spatiotemporal characteristics of the air pollutants in China from 2015 to 2019
Source: PLoS One. 2020 Aug 21;15(8):e0227469. doi: 10.1371/journal.pone.0227469 (PMC7444542; doi:10.1371/journal.pone.0227469)
Supplement: Attachment — Submitted filename: Response to Reviewers.docx [file pone.0227469.s002.docx]

Dear Zongbo Shi

Academic Editor

PLOS ONE,

Revision required [PONE-D-19-35106] - [EMID:b2ec848001643238]

Please find attached a revised version of our manuscript Study on the Spatiotemporal Characteristics of the Air Pollutants in China from 2014 to 2019.

The reviews's comments were highly insightful and enabled us to greatly improve the quality of our manuscript.

We hope that the revisions in the manuscript and our accompanying responses will be sufficient to make our manuscript suitable for publication in the Journal of PLOS ONE.

*Reviewer #1: The main issue with this manuscript is the quality of the language. The authors clearly struggle with both grammar and choice of vocabulary, making it quite difficult to follow their arguments. Since there is a lot of ambiguous phrasing with unclear word choices, the questions of whether the manuscript is technically sound and whether the data supports the conclusions cannot be fully answered at this point. There are several sections which are completely unintelligible to me. I appreciate that it can be quite difficult to write in a non-native language and that some mistakes are to be expected. However, in this case it really impedes proper understanding of the scientific content. I highly recommend that the authors try to get some assistance with the language when revising the manuscript.*

Response: We agree with this suggestion, we have improved the English writing by American Journal Experts in the revised manuscript.

*Additional comments:
While the authors have made all the data publicly available, the origin of the data is not clearly described. I am assuming that the data was extracted from databases of national/regional measurement networks, however this is not clear from the description and the reference links provided go to Chinese webpages, making them inaccessible to a large percentage of PLOS One readers. In addition, units are missing, and it would be helpful to provide the English names of the different locations in addition to the Chinese characters.*

Response: We agree with this suggestion, we provided his English name and his website(page 2-3, lines 41-54). In this article, the air pollution parameter data of 367 cities from 2014 to 2019 are sourced from the China National Environmental Monitoring Centre (English name).The website of the China Environmental Monitoring Centre is <http://www.cnemc.cn/> or <http://www.cnemc.cn/en/>.

And provided the data acquisition address of the China Environmental Monitoring Centre,making them inaccessible to a large percentage of PLOS One readers.The data acquisition address of the China Environmental Monitoring Centre is http://106.37.208.233:20035/. On this page, you can obtain real-time data on 7 air pollution parameters (PM2.5, PM10, air quality index (AQI), CO, NO2, O3, and SO2), including the mass concentration, of 367 cities, or you can go to <https://www.zq12369.com/> to acquire real-time historical data from the China Environmental Monitoring Centre.

*Additional comments:*

*The general idea behind the statistical analysis seems valid, but again there is a lack of information here regarding the input parameters of the radial basis function interpolation (e.g which basis function was used?). If the authors feel that this level of detail will not be of interest to most readers, it should at least go into the supplement.*

Response: We agree with this suggestion,information here regarding the input parameters of the radial basis function interpolation (page 4-5, lines 66-87).

This paper uses ArcGIS software to perform radial basis function (RBF) interpolation on the obtained air pollutant parameter data based on the abovementioned map of China.

This paper uses RBF interpolation. RBF interpolation is conducted to generate a smooth surface based on the large number of data points. These functions produce good results for gently changing surfaces, such as the elevation. There are a variety of RBFs to choose from in the RBF interpolation method. The selected RBF in this study is a regular spline function. First, the spline function is explained, after which the regular spline function is described. The interpolation method applied via the spline function tool estimates values using a mathematical function minimizing the total curvature of the surface. This produces a smooth surface that only passes through the input points. The algorithm of the spline function tool uses the following surface interpolation equation:

$$S\left( x,y \right)=T\left( x,y \right)+\sum_{j=1}^{N} \lambda_{j}R_{r_{j}}$$

where j = 1, 2, ..., N, N is the number of points, $\lambda_{j}$ is the coefficient obtained by solving a system of linear equations, and $r_{j}$ is the distance between point (x, y) and the j-th point.

The definitions of T(x, y) and R(r) will differ depending on the RBF selected. The regular spline function is defined as:

$$T\left( x,y \right)=a_{1}+a_{2}x+a_{3}y$$

where $a_{i}$ is the coefficient obtained by reconstructing the system of linear equations, and

$$R\left( r \right)=\frac{1}{2\pi}\left\{ \frac{r^{2}}{4}\left[ \ln\left( \frac{r}{2\tau} \right)+c-1 \right]+\tau^{2}\left[ K_{o}\left( \frac{r}{\tau} \right)+c+\ln\left( \frac{r}{2\pi} \right) \right] \right\}$$

where R is the distance between the point and the sample, $\tau^{2}$ is a weight parameter, $K_{o}$ is the modified Bessel function, and c is a constant, which is equal to 0.577215.

*Additional comments:*

*The results of the analysis are not put into* context *and there are no comparisons with other studies of air pollution trends.*

Response: We agree with this suggestion,rewritten the results of the analysis and put them into context (page 33-34, lines 619-658).

The PM2.5, PM10, AQI, CO, NO2, and SO2 levels in China exhibit a stable spatial distribution and persistence over time. PM2.5, PM10, AQI, CO, NO2, and SO2 are mostly concentrated in Beijing-Tianjin-Hebei and its surroundings and in Xinjiang from 2014 to 2019. The average concentration monotonically decreases (except CO).

A high NO2 level also occurs in areas where the economy is well developed and the vehicle population is large, including in the Yangtze River Delta, such as Shanghai, in the Pearl River Delta, such as Guangzhou, and in the Sichuan Basin, such as Chongqing.

The O3 level has consistently been high in Inner Mongolia, Qinghai, Gansu and Shandong from 2014 to 2019.

There is often a significant correlation between the mass concentrations of the different types of particulate matter. The R coefficient of the correlation between PM2.5 and PM10 is 0.88. There is a particularly significant linear positive correlation between these particulates, which indicates that they experience similar changes in the ambient air pollution and practically follow the same migration and transformation laws.

There is a significant positive linear correlation between the atmospheric particulate matter, AQI and pollution gases NO2 and SO2. The correlation coefficient between the atmospheric particulate matter (PM2.5 and PM10), AQI and SO2 is 0.97, 0.81, and 0.88, respectively, and the correlation coefficient between the atmospheric particulate matter (PM2.5 and PM10), AQI and NO2 is 0.82, 0.62, and 0.72, respectively, which is due to the mutual conversion caused by the chemical reaction between the particulate matter and gaseous pollutants.

There exist certain positive and negative correlations between the gaseous pollutants. There is a particularly significant linear positive correlation between N02 and SO2 (the correlation coefficient is 0.90), indicating that these two pollutants follow the same evolution law, and their influencing factors may be similar. N02 and SO2 are initially influenced by factors such as coal combustion and motor vehicle emissions, and there is no correlation between O3 and the other three gaseous pollutants.

The pollution sources in Beijing-Tianjin-Hebei area mainly include decomposition, coal burning, industrial production, and dust. The industrial structure has been adjusted in favor of the tertiary industry, and high-pollution, high-energy consumption secondary industries have been reduced but still account for a large proportion. Regarding the pollution and landforms in Xinjiang, there is a close relationship between static weather conditions, sandstorms, inversion layer, precipitation, dust and industrial sources, human activities, and mobile and nonroad mobile sources.

And comparisons with other studies of air pollution trends (page 19-23, lines 341-414).

**Study on the Correlation Between the Particulate Matter and the AQI**

There is often a significant correlation between the mass concentrations of different types of particulate matter. Table 8 summarizes the correlation between the hourly PM2.5 and PM10 mass concentrations from 2014 to 2019. The slope of the fitting equation between PM2.5 and PM10 is approximately 1, namely, 1.24. The correlation coefficient R is 0.88. The goodness of fit parameter R^2^ is 0.77. The mass concentration ratio of PM2.5 to PM10 is 46%, which is close to half, indicating that the degree of PM2.5 pollution in China is more severe. There is a particularly notable linear positive correlation between the two types of particulate matter, which indicates that they cause similar changes in ambient air pollution and practically follow the same migration and transformation laws. This is related to Liu Jie's research on the correlation of atmospheric particulates, and the results are consistent [4-5].

**Study on the Correlation Between the Atmospheric Particulate Matter, AQI and Gaseous Pollutants**

From the correlation study between the particulate matter and gaseous pollutants, as summarized in Table 9, there is a significant linear positive correlation between the atmospheric particulate matter, AQI and pollution gases (NO2 and SO2). The correlation coefficient between the atmospheric particulate matter (PM2.5 and PM10), AQI and SO2 is 0.97, 0.81, and 0.88, respectively, while the correlation coefficient between the atmospheric particulate matter (PM2.5 and PM10), AQI and NO2 is 0.82, 0.62, and 0.72, respectively. The above results are also consistent with the findings of Li Zhan et al. [6]. The atmospheric particulate matter (PM2.5 and PM10) and AQI are positively correlated with the atmospheric pollution gases NO2 and SO2, which usually occurs due to the mutual conversion caused by the chemical reaction between the particulate matter and the gaseous pollutants [7- 8]. It was found that nitrate (sulfate) produced by NO2 (SO2) would be transformed into secondary particulate matter (PM2.5 and PM10) over time, and with increasing NO2 and SO2 atmospheric pollution, the atmospheric particulate matter PM2.5 and PM10 also increased. One of the reasons for the increase in atmospheric pollutants NO2 and SO2 is the large vehicle flows and high exhaust emissions [9-10].

**Study on the Correlation Between the Gaseous Pollutants**

The study of the mutual evolution of the gaseous pollutants is more complicated. Generally, there is no simple law describing their mass concentrations in the short term, but the correlation can be studied through the long-term change trend of their mass concentrations to determine their individual relationships. As indicated in Table 10, there are certain positive and negative correlations between the gaseous pollutants. In Table 10, there is a particularly significant linear positive correlation between N02 and SO2 (a correlation coefficient of 0.90), indicating that these two pollutants follow the same evolution law, and their influencing factors may be similar. According to the analysis of the influencing factors of these two pollutants [11], the initial pollution of these two types can be determined. Both of these pollutants are influenced by factors such as coal combustion and motor vehicle emissions. There is no correlation between O3 and the other three gaseous pollutants [12]. Therefore, the evolution trends between the atmospheric particles, those between the atmospheric particles and gaseous pollutants and those between the gaseous pollutants are more complex. In addition, in-depth research is needed in many aspects, such as chemical reactions.

*Reviewer #2: This paper describes the spatial-temporal variation of atmospheric pollutants in the mainland of China during 2014 to 2019. The data in this paper covers hundreds of cities in China and 6 years. From this standpoint the article is useful and can be used to document levels at present day. However, I am not quite sure if the article fits the scope of the PLOS ONE because it looks like a data analysis report. I would suggest the author to expand the discussion on the reason of the variations.*

Response: We agree with this suggestion, so we have expanded the discussion on the reason of the variations(page 23-32, lines 416-617).

**Study on the Correlation Between the Particulate Matter and the AQI**

There is often a significant correlation between the mass concentrations of different types of particulate matter. Table 8 summarizes the correlation between the hourly PM2.5 and PM10 mass concentrations from 2014 to 2019. The slope of the fitting equation between PM2.5 and PM10 is approximately 1, namely, 1.24. The correlation coefficient R is 0.88. The goodness of fit parameter R^2^ is 0.77. The mass concentration ratio of PM2.5 to PM10 is 46%, which is close to half, indicating that the degree of PM2.5 pollution in China is more severe. There is a particularly notable linear positive correlation between the two types of particulate matter, which indicates that they cause similar changes in ambient air pollution and practically follow the same migration and transformation laws. This is related to Liu Jie's research on the correlation of atmospheric particulates, and the results are consistent [4-5].

**Study on the Correlation Between the Atmospheric Particulate Matter, AQI and Gaseous Pollutants**

From the correlation study between the particulate matter and gaseous pollutants, as summarized in Table 9, there is a significant linear positive correlation between the atmospheric particulate matter, AQI and pollution gases (NO2 and SO2). The correlation coefficient between the atmospheric particulate matter (PM2.5 and PM10), AQI and SO2 is 0.97, 0.81, and 0.88, respectively, while the correlation coefficient between the atmospheric particulate matter (PM2.5 and PM10), AQI and NO2 is 0.82, 0.62, and 0.72, respectively. The above results are also consistent with the findings of Li Zhan et al. [6]. The atmospheric particulate matter (PM2.5 and PM10) and AQI are positively correlated with the atmospheric pollution gases NO2 and SO2, which usually occurs due to the mutual conversion caused by the chemical reaction between the particulate matter and the gaseous pollutants [7- 8]. It was found that nitrate (sulfate) produced by NO2 (SO2) would be transformed into secondary particulate matter (PM2.5 and PM10) over time, and with increasing NO2 and SO2 atmospheric pollution, the atmospheric particulate matter PM2.5 and PM10 also increased. One of the reasons for the increase in atmospheric pollutants NO2 and SO2 is the large vehicle flows and high exhaust emissions [9-10].

**Study on the Correlation Between the Gaseous Pollutants**

The study of the mutual evolution of the gaseous pollutants is more complicated. Generally, there is no simple law describing their mass concentrations in the short term, but the correlation can be studied through the long-term change trend of their mass concentrations to determine their individual relationships. As indicated in Table 10, there are certain positive and negative correlations between the gaseous pollutants. In Table 10, there is a particularly significant linear positive correlation between N02 and SO2 (a correlation coefficient of 0.90), indicating that these two pollutants follow the same evolution law, and their influencing factors may be similar. According to the analysis of the influencing factors of these two pollutants [11], the initial pollution of these two types can be determined. Both of these pollutants are influenced by factors such as coal combustion and motor vehicle emissions. There is no correlation between O3 and the other three gaseous pollutants [12]. Therefore, the evolution trends between the atmospheric particles, those between the atmospheric particles and gaseous pollutants and those between the gaseous pollutants are more complex. In addition, in-depth research is needed in many aspects, such as chemical reactions.

Causes of Air Pollution in the Beijing-Tianjin-Hebei Region

The unique geographical environment of the Beijing-Tianjin-Hebei region coupled with the rapid development of urban agglomerations and the formation of local atmospheric circulation formation conditions make result in the air pollution in the Beijing-Tianjin-Hebei region show exhibiting a certain particular spatiotemporal distribution distribution [13]. In 2014, the energy consumption level of Beijing-Tianjin-Hebei accounted for 12% of the country's total energy consumption level of China. There are still more numerous heavyhigh-energy consumption heavy industries in the Beijing-Tianjin-Hebei region. Compared to 2010, In 2014, the overall GDP increased by 52% in 2014 compared to 2010. The strength magnitude of the country's consumption in China of the accumulated resources in per unit of land area of per unit,per unit timeover time and the intensity of pollutant emissions are very high [14].

The In the Beijing-Tianjin-Hebei region, takes Beijing is selected as an example. The analysis results of theBeijing ’s Beijing’s air pollution sources in Beijing released in 2014 in 2014 [15] show reveal that aboutapproximately 28% to 36% of the atmospheric PM2.5 sources come from regional transmissiontransport. Local emissions account for aboutapproximately 64%-72%, including 31.1% of from motor vehicles, 22.4% of from coal, 18.1% of from industrial production, 14.3% of from dust, and 14.1% from other activities, such as catering, livestock and poultry breeding, and auto repair accounted automobile repair, accounting for 14.1%. It can be seen that vehicle Vehicle exhaust is the most important source of local pollution. The structures of the primary industry, secondary industry, and tertiary industriesy of in Beijing were adjusted from 0.9: 24. 0: 75. 1 in 2010 in 2010 to 0.7: 21. 4: 77. 9 in 2014. The tertiary industry areis dominateddominates, and compared to 2010, their its proportion has increased by nearly 3% compared with 2010, indicating that Beijing's the industrial structure of Beijing is still optimizingbeing optimized. The source of air pollution is mainly caused by the industrial production process of the secondary industry and vehicle exhaust emissions.

Notes: The primary industry mainly refers to those industries that produce foodstuffs and other biological materials, including crops, forestry, animal husbandry, aquaculture and other industries that directly target natural objects. The secondary industry mainly refers to the processing and manufacturing industry, which uses consumes the basic materials provided by nature and the primary industry for during processing. The tertiary industry refers to those other industries other than the primary and secondary industries, and has a wide range, mainly including nonmaterial-material production sectors such as transportation, communications, commerce, catering, finance, education, and public services.

The results of a new round of PM2.5 source analysis completed by the Beijing Environmental Protection Monitoring Center in 2018 in 2018 were released to the public, and. tThe main conclusions of this study indicate that 2/3 of the main sources of PM2.5 in Beijing throughout the year are local and 1/3 are regional transmissions. From the characteristics of the city ’scity’sthe current local atmospheric PM2.5 sources in Beijing, in terms of the local emissions contribution of local emissions, mobile sources are account for 45%, flying fugitive dust sources account for are 16%, industrial sources account for are 12%, living surface sources account for are 12%, coal sources account for are 3%, and agricultural, natural sourcesand, others sources account for aboutapproximately 12% of the PM2.5 concentration. The results show that at this stage, the coal-fired source has basically withdrawn from the ranks ofstopped being the city's main source of atmospheric PM2.5 in Beijing [58].

TIn the Beijing-Tianjin-Hebei region, takes Tianjin is selected as an example. The analysis results of the air pollution sources in Tianjin in 2014 in 2014 showed [16] that the regional transmission of atmospheric PM2.5 sources accounted for 22%-34% of the pollution. The remaining 66% to 78% came from local emissions. Among them, dust sources accounted for 30%, coal-fired sources accounted for 27%, motorized sources accounted for 20%, industrial production sources accounted for 17%, and other sources accounted for 6%. Dust is the primary source of pollution, followed by coal combustion and motor vehicles. The rRoads, storage yards, construction sites, etc. in Tianjin are the main sources of dust. In addition, construction projects in the railway, chemical, and power industries will also generate dust. In 2014, the three industrial structures in Tianjin were adjusted from 1.6: 53.1: 45.3 in 2010 in 2010 to 1.3: 49.4: 49.3. The secondary industry dropped decreased by 3.7% percentage points, and the tertiary industry increased by 4% percentage points. Until 2014, the proportions of the secondary and tertiary industries in Tianjin was basically flatpractically remained stable. Comparing In comparing the data, it is found that the proportion of the Tianjin's secondary industry in Tianjin is still the highest despite a its decline. Thermal power, iron and steel, petrochemical, cement, building materials, etc., as typical heavy high-pollution emission industries, are the key sources of pollution caused bycausing the air pollution in Tianjin.

The Aanalytical results of the air pollution sources in Hebei Province in the Beijing-Tianjin-Hebei region show reveal that [17], taking with Shijiazhuang city as an example, regional transmission accounts for 23% to 30% of the PM2.5 sourcespollution, and local pollution emissions accounts for 70% to 77%, of which coal combustion accounts for 28.5%, industrial production accounts for 25.2%, fugitive dust accounts for 22.5%, motor vehicles account for 15.0%, and other sources such as biomass burning, and catering, etc. account for 8.8% of the PM2.5 pollution. Coal combustion is the primary source of the air pollutants in Shijiazhuang and even in Hebei Province. Large The high coal consumption and irrational coal combustion structurestructures are the main causes of soot pollution. In 2014, the three industrial structures in Hebei Province were adjusted from 12.57: 52.5: 34.93 in 2010 in 2010 to 11.7: 51.1: 37.2. The primary industry declined decreased slightly, and the secondary industry decreased by 1.4 %percentage points, and while the tertiary industry increased by approximately 2-3 %percentage pointpoints. The proportion of the secondary industry in Hebei Province is still higher than 50%. This irrational industrial structure makes Hebei Province the region with the worst air pollution in the Beijing-Tianjin-Hebei. In addition, Hebei has a large high proportion of high-pollution and high-energy- consuming consumption industries, including steel, cement, electricity, glass, pharmaceuticals, metallurgy, petrochemicals, and building materials. Among them, the coal consumption in the steel, building materials and power industries accounted for 89.6% of the province's provincial total energy consumption, and the air pollutant emissions in from the steel, cement, power, and glass industries accounted for aboutapproximately 60% of the Hebei's total pollution in Hebei. [18]. As the most important pollution industry in Hebei, steel is the focus of air pollution control.

Causes of Air Pollution in Xinjiang

Landform analysis

Xinjiang is far from the sea, surrounded by mountains and basins, and deserts and grasslands are widely distributed. Taking With the topography of Urumqi as an example, its southeast and west sides are surrounded by mountains with an average elevation of 680 metersm. The slope drop gradient is largehigh, with the south is being high, while the north is low, and the middle is are low. The topographical conditions are very highly detrimental to the atmospheric circulation of the atmosphere and pollutant the diffusion of pollutants. The Urumqi region is located in the northern part of the Tianshan Mountains and the southern edge of the Jungle Basin. It is the city farthest from the ocean in the world. It has a typical desert continental climate and a fragile ecological environment. The vegetation coverage around across the urban area is low, and there are many bare groundsareas, which are liable to form dusty weather conditions.

Small Weak winds

The frequent occurrence of calm and small weak winds in Xinjiang (wind speeds greater higher than or equal to 0.5 m/s are and less lower than 1.5 m/s are characterize small weak winds), which is an important factor for the poor air environmental quality factor. In the case of small weak winds, the horizontal movement of the atmosphere is slow, which is not conducive to the lateral diffusion of atmospheric pollutants, and it is easy to form regional pollution accumulation[19regional pollution accumulation easily occurs [19-20]. Wind has a certain diffusion and purification ability, and the wind speed and pollutant concentration are basically negatively correlated. Taking With Urumqi as an example, the frequency of static wind atmospheric conditions in the heating period is as high as aboutapproximately 26%, and in December and January, it is as high as 30% or more. Even if there is wind, the wind speed is very small low and rarely exceeds level 3 [21]. Such meteorological conditions are extremely detrimental to the spread of pollutants dispersal. If there are several consecutive days of static wind weather conditions, the pollutants will continue to accumulate, and the air quality will deteriorate sharply [22].

Sand storms

Sandstorms are a typical weather phenomenon in Xinjiang. Sandstorms and floating dust occur throughout the year. Sandstorms and floating dust weather conditions will cause high concentrations of atmospheric particulate matter concentrations, which will seriously affect all the regionsregions’regional air quality in cities [23]. The source region of western China, with the Taklimakan Desert as the main bodyregion, and the source region of northern China, with the Badain Jaran Desert as the center, contributed 70% of the total dust emissions in Asia. The sSandstorms in Northwest China are dominated by dry settlement [24].Regional The regional distribution of the sandstorms in Northwest China is very uneven, while the sandstorm frequency in the northern region of Xinjiang is basically unchanged, and that in the rest of the region is decreasing [25].

Inversion layer

Among the weather types in Urumqi, there are mostly neutral and stable weather types of weather. The frequency of temperature inversion is as high as 90% in winter. The severe air pollution in winter is directly related to thisthese stable meteorological conditions. The frequently occurring inversion layer that occurs frequently is like is similar to a large pot lid, covering the city, making thereby trapping atmospheric pollutants trapped in theat low altitudealtitudes and making them difficult to spreaddisperse. There is a clear positive correlation between inverse temperature inversion and pollutants pollution [22].

Precipitation

Taking Thethe Urumqi region, as an example, it is located in an arid and semiarid-arid environment with little precipitation and large high evaporation. Precipitation has a purifying effect on the air and can effectively purify urban air. There is a clear negative correlation between precipitation and pollutantspollution, which is one of the key factors determining the air quality of air. For the SO2, NO2 and PM10 levels in Urumqi., The the daily average concentration and precipitation are analyzed. The analysis results show that the wet removal ability of rain is greater higher than that of snow. rainfallRainfall and snowfall both have wet removal abilityabilities for these three air pollutants [26],, and the strongest highest removal ability is for S02., This is followed by that for PM10.

Dust source

In 2017, with the new area took Turfan City city as as an example of a new area, the emission source of PM10 emission level was 1.169 million tons, and while that of the PM2.5 emissions was 132,000 tons. The source of sSoil dust is the primary source of the particulate emissions in Turpan. Turpan is a sparsely populated area. According to statistics (Turpan Statistical Yearbook (2017)) statistics, the area of land other than urban construction land,and transportation land, water areas and water conservancy facility sites is covers 68,900 square kilometers, with a large area of bare soil. In addition, the Turpan Basin is surrounded by Gobis and deserts. Gobis and deserts account for 76.73% of the area, and there is less vegetation and green land. When windy and sand weather conditions occurs, it is very easy to cause large dust, and frequent dust storms are easily formed. The dust Dust is not only the a source of atmospheric particulate matters, but is also receptors a receptor of particulate matter, resulting in natural dust fall of ground soil dust and sand dust transmission, contributecontributing greatly to the concentration of particulate matter concentration in the atmosphere [27].

Industrial source

Taking With Turpan Ccity in 2017 as an example, in 2017, the PM10 emissions from industrial sources were 19,200 tons, and the PM2.5 emissions were totaled 4,900 tons,the. The main PM10 contribution sources of PM10 are non-metallicthe nonmetallic mineral products industry and power thermal power production, accounting for 81.4% and 15.2%, respectively, of the PM10 pollution;. The PM2.5 contribution of the non-metallicnonmetallic mineral products industry contribution rate is 85.9%, The and the contributions rate of power thermal power production and petroleum processing is are comparable. [27].

Source of life

Taking With Turfan Ccity in 2017 as an example, in 2017, the PM10 emissions of PM10 in thefrom living sourcesourceshuman activities was 94.58 million tons, and the PM2.5 emission amount of PM2.5 was 73.18 million tons. The main sources of the emissions of atmospheric particulate matter PM10 and PM2.5 in the living sourceterms of human activities were was coal combustion and PM 10 and PM 2.5 emissions. Civilian coal combustion technology is poor, and there isare no scientific and effective emission control measures, and while the amount of fine particulate matter PM2.5 emitted by from coal combustion is even higher than that of emitted from industrial activities. During the heating season, civilian coal combustion and central heating caused a large amount of pollutants to be emitted, and a large number amounts of primary aerosols, heavy metals, and water-soluble ions were are directly emitted directly as primary particulate matter. At the same time, gaseous precursors such as SO2 and NO2 were are released. Adverse weather conditions It is easy to form seasily result in severe pollutioneverely polluted weather under adverse weather conditions. The PM2.5 released from food sources is mainly from the cooking process of involving edible oils and foods. Under high- temperature conditions, particulate matter is formed by chemical reactions such as thermal oxidation and thermal decomposition. Most of the food and beverage sources are occur in densely populated areas, and pollutants are emitted near the ground, which has an important notable impact on the health of the population. According to activity- level survey information, there were a total of 3,443 catering companies in Turpan in 2017. The total PM10 emissions were 301.6 tons, and the total PM2.5 emissions were 241.3 tons. In addition, the exhaust gas from restaurant fumes contains a large number of volatile organic pollutantspollutant VOCs, among which oxygenates and olefins have a high photochemical activity,, which are precursors to form secondary particulate matter, and have and have an importanta notable impact on atmospheric particulate pollution [28].

Mobile source

    At present, vehicle exhaust is one of the main sources of the atmospheric particulate matter in China [29-30], especially PM2.5 fine particulate matter PM2.5. Motor vehicles also emit gaseous pollutants such as NOx and VOCs, which are converted into secondary particulate matter through photochemical reactions. Compared with to coarse particles, fine particles have a larger specific surface area, which thus capable of absorbsing and carryingies more toxic substances, which is can be harmful to human health [29-32]. The rRoad sources in Turpan in 2017 in 2017 emitted 249.19 tons of PM10 and 224.5 tons of PM2.5, respectively [33].

OffNonroad-road mobile source

     Turpan's The nonroad-road mobile sources in Turpan are mainly divided into three categories: engineering machinery, agricultural machinery and civilian aviation aircraft. Compared with to road mobile sources, nonroad-road mobile sources usually use diesel and heavy oil as their the main fuels. They have the characteristics of a high fuel consumption, long service life, low technical level and low maintenance rate. Most of the pollutants emitted are NOx and particulate matter [34-35].

*Journal Requirements:*

*4. We note that Figures 1-7 in your submission contain satellite images which may be copyrighted. All PLOS content is published under the Creative Commons Attribution License (CC BY 4.0), which means that the manuscript, images, and Supporting Information files will be freely available online, and any third party is permitted to access, download, copy, distribute, and use these materials in any way, even commercially, with proper attribution. For these reasons, we cannot publish previously copyrighted maps or satellite images created using proprietary data, such as Google software (Google Maps, Street View, and Earth). For more information, see our copyright guidelines: http://journals.plos.org/plosone/s/licenses-and-copyright.*

  We used resources for replacing copyrighted map figures, USGS National Map Viewer (public domain): http://viewer.nationalmap.gov/viewer/ (page 3, lines 46-48).
