# Supplementary material for: The spatiotemporal characteristics of the air pollutants in China from 2015 to 2019
Source: PLoS One. 2020 Aug 21;15(8):e0227469. doi: 10.1371/journal.pone.0227469 (PMC7444542; doi:10.1371/journal.pone.0227469)
Supplement: Attachment — Submitted filename: PONE-D-19-35106_R1 Shi.docx [file pone.0227469.s003.docx]

PLOS ONE

Study on the Spatiotemporal Characteristics of the Air Pollutants in China from 2014 to

2019

--Manuscript Draft--

| Manuscript Number: | PONE-D-19-35106R1 |
| --- | --- |
| Article Type: | Research Article |
| Full Title: | Study on the Spatiotemporal Characteristics of the Air Pollutants in China from 2014 to 2019 |
| Short Title: | Spatial-temporal Variation of Atmospheric Pollution |
| Corresponding Author: | guo peng  Moscow of university  Moscow, RUSSIAN FEDERATION |
| Keywords: | Atmospheric particulate matter; pollution gas; spatiotemporal change; Beijing-TianjinHebei; Xinjiang |
| Abstract: | This article is based on hourly mass concentration data on 7 air pollution parameters (PM2.5, PM10, air quality index (AQI), CO, NO2, O3, and SO2) for 367 cities in China from 2014 to 2019. The study found that the concentration of the air pollutants in China (PM2.5, PM10, AQI, CO, NO2, and SO2) exhibited a consistent spatial and temporal distribution in China, with high concentrations observed in Beijing-Tianjin-Hebei and its surrounding areas and Xinjiang from 2014 to 2019. O3 showed a different spatial pattern, which showed a higher concentration in Inner Mongolia, Qinghai, Gansu and Shandong. The average CO and O3 concentrations monotonically decreased across China. Based on the abovementioned spatiotemporal characteristics of the atmospheric pollutants, we analyzed the correlations among the seven parameters. There was a positive correlation among three pollutants, i.e., PM2.5 and PM10 and the AQI. PM2.5 and PM10 and AQI are also positively correlated with NO2 and SO2. There was also a positive  correlation between atmospheric pollution gases NO2 and SO2, while the correlation between O3 and every other pollutant was negative. The pollution sources in the Beijing-Tianjin-Hebei region and Xinjiang were studied. |
| Order of Authors: | guo peng |
|  | Yunqi Luan |
|  | Aminat Batalbievna Umarova |
| Opposed Reviewers: |  |
| Response to Reviewers: | Dear Zongbo Shi  Academic Editor  PLOS ONE,  Revision required [PONE-D-19-35106] - [EMID:b2ec848001643238]  Please find attached a revised version of our manuscript Study on the Spatiotemporal Characteristics of the Air Pollutants in China from 2014 to 2019.  The reviews's comments were highly insightful and enabled us to greatly improve the quality of our manuscript.  We hope that the revisions in the manuscript and our accompanying responses will be sufficient to make our manuscript suitable for publication in the Journal of PLOS ONE.  Reviewer #1: The main issue with this manuscript is the quality of the language. The authors clearly struggle with both grammar and choice of vocabulary, making it quite difficult to follow their arguments. Since there is a lot of ambiguous phrasing with |

|  | unclear word choices, the questions of whether the manuscript is technically sound and whether the data supports the conclusions cannot be fully answered at this point. There are several sections which are completely unintelligible to me. I appreciate that it can be quite difficult to write in a non-native language and that some mistakes are to be expected. However, in this case it really impedes proper understanding of the scientific content. I highly recommend that the authors try to get some assistance with the language when revising the manuscript.  Response: We agree with this suggestion, we have improved the English writing by American Journal Experts in the revised manuscript.  Additional comments:  While the authors have made all the data publicly available, the origin of the data is not clearly described. I am assuming that the data was extracted from databases of national/regional measurement networks, however this is not clear from the description and the reference links provided go to Chinese webpages, making them inaccessible to a large percentage of PLOS One readers. In addition, units are missing, and it would be helpful to provide the English names of the different locations in addition to the Chinese characters.  Response: We agree with this suggestion, we provided his English name and his website(page 2-3, lines 35-49). In this article, the air pollution parameter data of 367 cities from 2014 to 2019 are sourced from the China National Environmental  Monitoring Centre (English name).The website of the China Environmental Monitoring Centre is http://www.cnemc.cn/ or http://www.cnemc.cn/en/.  And provided the data acquisition address of the China Environmental Monitoring Centre,making them inaccessible to a large percentage of PLOS One readers.The data acquisition address of the China Environmental Monitoring Centre is http://106.37.208.233:20035/. On this page, you can obtain real-time data on 7 air pollution parameters (PM2.5, PM10, air quality index (AQI), CO, NO2, O3, and SO2), including the mass concentration, of 367 cities, or you can go to  https://www.zq12369.com/ to acquire real-time historical data from the China Environmental Monitoring Centre.  Additional comments:  The general idea behind the statistical analysis seems valid, but again there is a lack of information here regarding the input parameters of the radial basis function  interpolation (e.g which basis function was used?). If the authors feel that this level of detail will not be of interest to most readers, it should at least go into the supplement. Response:We agree with this suggestion,information here regarding the input parameters of the radial basis function interpolation (page 3-4, lines 50-64).  This paper uses ArcGIS software to perform radial basis function (RBF) interpolation on the obtained air pollutant parameter data based on the abovementioned map of China.  This paper uses RBF interpolation. RBF interpolation is conducted to generate a smooth surface based on the large number of data points. These functions produce good results for gently changing surfaces, such as the elevation. There are a variety of RBFs to choose from in the RBF interpolation method. The selected RBF in this study is a regular spline function. First, the spline function is explained, after which the regular spline function is described. The interpolation method applied via the spline function tool estimates values using a mathematical function minimizing the total curvature of the surface. This produces a smooth surface that only passes through the input points. The algorithm of the spline function tool uses the following surface interpolation equation:  where j = 1, 2, ..., N, N is the number of points, is the coefficient obtained by solving a system of linear equations, and is the distance between point (x, y) and the j-th point. The definitions of T(x, y) and R(r) will differ depending on the RBF selected. The regular spline function is defined as:  where is the coefficient obtained by reconstructing the system of linear equations, and  where R is the distance between the point and the sample, is a weight parameter, is the modified Bessel function, and c is a constant, which is equal to 0.577215. |
| --- | --- |

|  | Additional comments:  The results of the analysis are not put into and there are no comparisons with other studies of air pollution trends.  Response: We agree with this suggestion,rewritten the results of the analysis and put them into context (page 27-28, lines 452-477).  The PM2.5, PM10, AQI, CO, NO2, and SO2 levels in China exhibit a stable spatial distribution and persistence over time. PM2.5, PM10, AQI, CO, NO2, and SO2 are mostly concentrated in Beijing-Tianjin-Hebei and its surroundings and in Xinjiang from 2014 to 2019. The average concentration monotonically decreases (except CO). A high NO2 level also occurs in areas where the economy is well developed and the vehicle population is large, including in the Yangtze River Delta, such as Shanghai, in the Pearl River Delta, such as Guangzhou, and in the Sichuan Basin, such as Chongqing.  The O3 level has consistently been high in Inner Mongolia, Qinghai, Gansu and Shandong from 2014 to 2019.  There is often a significant correlation between the mass concentrations of the different types of particulate matter. The R coefficient of the correlation between PM2.5 and PM10 is 0.88. There is a particularly significant linear positive correlation between these particulates, which indicates that they experience similar changes in the ambient air pollution and practically follow the same migration and transformation laws. There is a significant positive linear correlation between the atmospheric particulate matter, AQI and pollution gases NO2 and SO2. The correlation coefficient between the atmospheric particulate matter (PM2.5 and PM10), AQI and SO2 is 0.97, 0.81, and 0.88, respectively, and the correlation coefficient between the atmospheric particulate matter (PM2.5 and PM10), AQI and NO2 is 0.82, 0.62, and 0.72, respectively, which is due to the mutual conversion caused by the chemical reaction between the particulate matter and gaseous pollutants.  There exist certain positive and negative correlations between the gaseous pollutants. There is a particularly significant linear positive correlation between N02 and SO2 (the correlation coefficient is 0.90), indicating that these two pollutants follow the same evolution law, and their influencing factors may be similar. N02 and SO2 are initially influenced by factors such as coal combustion and motor vehicle emissions, and there is no correlation between O3 and the other three gaseous pollutants.  The pollution sources in Beijing-Tianjin-Hebei area mainly include decomposition, coal burning, industrial production, and dust. The industrial structure has been adjusted in favor of the tertiary industry, and high-pollution, high-energy consumption secondary industries have been reduced but still account for a large proportion. Regarding the pollution and landforms in Xinjiang, there is a close relationship between static weather conditions, sandstorms, inversion layer, precipitation, dust and industrial sources, human activities, and mobile and nonroad mobile sources.  And comparisons with other studies of air pollution trends (page 17-20, lines 257-308)  .  Study on the Correlation Between the Particulate Matter and the AQI  There is often a significant correlation between the mass concentrations of different types of particulate matter. Table 8 summarizes the correlation between the hourly PM2.5 and PM10 mass concentrations from 2014 to 2019. The slope of the fitting equation between PM2.5 and PM10 is approximately 1, namely, 1.24. The correlation coefficient R is 0.88. The goodness of fit parameter R2 is 0.77. The mass  concentration ratio of PM2.5 to PM10 is 46%, which is close to half, indicating that the degree of PM2.5 pollution in China is more severe. There is a particularly notable linear positive correlation between the two types of particulate matter, which indicates that they cause similar changes in ambient air pollution and practically follow the same migration and transformation laws. This is related to Liu Jie's research on the correlation of atmospheric particulates, and the results are consistent [4-5]. Study on the Correlation Between the Atmospheric Particulate Matter, AQI and  Gaseous Pollutants  From the correlation study between the particulate matter and gaseous pollutants, as summarized in Table 9, there is a significant linear positive correlation between the atmospheric particulate matter, AQI and pollution gases (NO2 and SO2). The  correlation coefficient between the atmospheric particulate matter (PM2.5 and PM10), AQI and SO2 is 0.97, 0.81, and 0.88, respectively, while the correlation coefficient between the atmospheric particulate matter (PM2.5 and PM10), AQI and NO2 is 0.82, 0.62, and 0.72, respectively. The above results are also consistent with the findings of  Li Zhan et al. [6]. The atmospheric particulate matter (PM2.5 and PM10) and AQI are |
| --- | --- |

|  | positively correlated with the atmospheric pollution gases NO2 and SO2, which usually occurs due to the mutual conversion caused by the chemical reaction between the particulate matter and the gaseous pollutants [7- 8]. It was found that nitrate (sulfate) produced by NO2 (SO2) would be transformed into secondary particulate matter (PM2.5 and PM10) over time, and with increasing NO2 and SO2 atmospheric pollution, the atmospheric particulate matter PM2.5 and PM10 also increased. One of the reasons for the increase in atmospheric pollutants NO2 and SO2 is the large vehicle flows and high exhaust emissions [9-10].  Study on the Correlation Between the Gaseous Pollutants  The study of the mutual evolution of the gaseous pollutants is more complicated. Generally, there is no simple law describing their mass concentrations in the short term, but the correlation can be studied through the long-term change trend of their mass concentrations to determine their individual relationships. As indicated in Table  10, there are certain positive and negative correlations between the gaseous pollutants. In Table 10, there is a particularly significant linear positive correlation between N02 and SO2 (a correlation coefficient of 0.90), indicating that these two pollutants follow the same evolution law, and their influencing factors may be similar. According to the analysis of the influencing factors of these two pollutants [11], the initial pollution of these two types can be determined. Both of these pollutants are influenced by factors such as coal combustion and motor vehicle emissions. There is no correlation between O3 and the other three gaseous pollutants [12]. Therefore, the evolution trends between the atmospheric particles, those between the atmospheric particles and gaseous pollutants and those between the gaseous pollutants are more complex. In addition, in-depth research is needed in many aspects, such as chemical reactions.  Reviewer #2: This paper describes the spatial-temporal variation of atmospheric pollutants in the mainland of China during 2014 to 2019. The data in this paper covers hundreds of cities in China and 6 years. From this standpoint the article is useful and can be used to document levels at present day. However, I am not quite sure if the article fits the scope of the PLOS ONE because it looks like a data analysis report. I would suggest the author to expand the discussion on the reason of the variations. Response: We agree with this suggestion, so we have expanded the discussion on the reason of the variations(page 17-26, lines 257-450)  .  Study on the Correlation Between the Particulate Matter and the AQI  There is often a significant correlation between the mass concentrations of different types of particulate matter. Table 8 summarizes the correlation between the hourly PM2.5 and PM10 mass concentrations from 2014 to 2019. The slope of the fitting equation between PM2.5 and PM10 is approximately 1, namely, 1.24. The correlation coefficient R is 0.88. The goodness of fit parameter R2 is 0.77. The mass  concentration ratio of PM2.5 to PM10 is 46%, which is close to half, indicating that the degree of PM2.5 pollution in China is more severe. There is a particularly notable linear positive correlation between the two types of particulate matter, which indicates that they cause similar changes in ambient air pollution and practically follow the same migration and transformation laws. This is related to Liu Jie's research on the correlation of atmospheric particulates, and the results are consistent [4-5]. Study on the Correlation Between the Atmospheric Particulate Matter, AQI and  Gaseous Pollutants  From the correlation study between the particulate matter and gaseous pollutants, as summarized in Table 9, there is a significant linear positive correlation between the atmospheric particulate matter, AQI and pollution gases (NO2 and SO2). The  correlation coefficient between the atmospheric particulate matter (PM2.5 and PM10), AQI and SO2 is 0.97, 0.81, and 0.88, respectively, while the correlation coefficient between the atmospheric particulate matter (PM2.5 and PM10), AQI and NO2 is 0.82, 0.62, and 0.72, respectively. The above results are also consistent with the findings of Li Zhan et al. [6]. The atmospheric particulate matter (PM2.5 and PM10) and AQI are positively correlated with the atmospheric pollution gases NO2 and SO2, which usually occurs due to the mutual conversion caused by the chemical reaction between the particulate matter and the gaseous pollutants [7- 8]. It was found that nitrate (sulfate) produced by NO2 (SO2) would be transformed into secondary particulate matter (PM2.5 and PM10) over time, and with increasing NO2 and SO2 atmospheric pollution, the atmospheric particulate matter PM2.5 and PM10 also increased. One of the reasons for the increase in atmospheric pollutants NO2 and SO2 is the large vehicle flows and high exhaust emissions [9-10]. |
| --- | --- |

|  | Study on the Correlation Between the Gaseous Pollutants  The study of the mutual evolution of the gaseous pollutants is more complicated. Generally, there is no simple law describing their mass concentrations in the short term, but the correlation can be studied through the long-term change trend of their mass concentrations to determine their individual relationships. As indicated in Table  10, there are certain positive and negative correlations between the gaseous pollutants. In Table 10, there is a particularly significant linear positive correlation between N02 and SO2 (a correlation coefficient of 0.90), indicating that these two pollutants follow the same evolution law, and their influencing factors may be similar. According to the analysis of the influencing factors of these two pollutants [11], the initial pollution of these two types can be determined. Both of these pollutants are influenced by factors such as coal combustion and motor vehicle emissions. There is no correlation between O3 and the other three gaseous pollutants [12]. Therefore, the evolution trends between the atmospheric particles, those between the atmospheric particles and gaseous pollutants and those between the gaseous pollutants are more complex. In addition, in-depth research is needed in many aspects, such as chemical reactions.  Causes of Air Pollution in the Beijing-Tianjin-Hebei Region  The unique geographical environment of the Beijing-Tianjin-Hebei region coupled with the rapid development of urban agglomerations and the formation of local atmospheric circulation formation conditions make result in the air pollution in the Beijing-TianjinHebei region show exhibiting a certain particular spatiotemporal distribution distribution [13]. In 2014, the energy consumption level of Beijing-Tianjin-Hebei accounted for 12% of the country's total energy consumption level of China. There are still more numerous heavyhigh-energy consumption heavy industries in the Beijing-Tianjin-Hebei region. Compared to 2010, In 2014, the overall GDP increased by 52% in 2014 compared to  2010. The strength magnitude of the country's consumption in China of the accumulated resources in per unit of land area of per unit,per unit timeover time and the intensity of pollutant emissions are very high [14].  The In the Beijing-Tianjin-Hebei region, takes Beijing is selected as an example. The analysis results of theBeijing ’s Beijing’s air pollution sources in Beijing released in 2014 in 2014 [15] show reveal that aboutapproximately 28% to 36% of the atmospheric PM2.5 sources come from regional transmissiontransport. Local emissions account for aboutapproximately 64%-72%, including 31.1% of from motor vehicles, 22.4% of from coal, 18.1% of from industrial production, 14.3% of from dust, and 14.1% from other activities, such as catering, livestock and poultry breeding, and auto repair accounted automobile repair, accounting for 14.1%. It can be seen that vehicle Vehicle exhaust is the most important source of local pollution. The structures of the primary industry, secondary industry, and tertiary industriesy of in Beijing were adjusted from 0.9: 24. 0:  75. 1 in 2010 in 2010 to 0.7: 21. 4: 77. 9 in 2014. The tertiary industry areis dominateddominates, and compared to 2010, their its proportion has increased by nearly 3% compared with 2010, indicating that Beijing's the industrial structure of Beijing is still optimizingbeing optimized. The source of air pollution is mainly caused by the industrial production process of the secondary industry and vehicle exhaust emissions.  Notes: The primary industry mainly refers to those industries that produce foodstuffs and other biological materials, including crops, forestry, animal husbandry, aquaculture and other industries that directly target natural objects. The secondary industry mainly refers to the processing and manufacturing industry, which uses consumes the basic materials provided by nature and the primary industry for during processing. The tertiary industry refers to those other industries other than the primary and secondary industries, and has a wide range, mainly including nonmaterial-material production sectors such as transportation, communications, commerce, catering, finance, education, and public services.  The results of a new round of PM2.5 source analysis completed by the Beijing Environmental Protection Monitoring Center in 2018 in 2018 were released to the public, and. tThe main conclusions of this study indicate that 2/3 of the main sources of PM2.5 in Beijing throughout the year are local and 1/3 are regional transmissions. From the characteristics of the city ’scity’sthe current local atmospheric PM2.5 sources in Beijing, in terms of the local emissions contribution of local emissions, mobile sources are account for 45%, flying fugitive dust sources account for are 16%, industrial sources account for are 12%, living surface sources account for are 12%, coal sources account for are 3%, and agricultural, natural sourcesand, others sources account for aboutapproximately 12% of the PM2.5 concentration. The results show that |
| --- | --- |

|  | at this stage, the coal-fired source has basically withdrawn from the ranks ofstopped being the city's main source of atmospheric PM2.5 in Beijing [58].  TIn the Beijing-Tianjin-Hebei region, takes Tianjin is selected as an example. The analysis results of the air pollution sources in Tianjin in 2014 in 2014 showed [16] that the regional transmission of atmospheric PM2.5 sources accounted for 22%-34% of the pollution. The remaining 66% to 78% came from local emissions. Among them, dust sources accounted for 30%, coal-fired sources accounted for 27%, motorized sources accounted for 20%, industrial production sources accounted for 17%, and other sources accounted for 6%. Dust is the primary source of pollution, followed by coal combustion and motor vehicles. The rRoads, storage yards, construction sites, etc. in Tianjin are the main sources of dust. In addition, construction projects in the railway, chemical, and power industries will also generate dust. In 2014, the three industrial structures in Tianjin were adjusted from 1.6: 53.1: 45.3 in 2010 in 2010 to 1.3: 49.4: 49.3. The secondary industry dropped decreased by 3.7% percentage points, and the tertiary industry increased by 4% percentage points. Until 2014, the proportions of the secondary and tertiary industries in Tianjin was basically flatpractically remained stable. Comparing In comparing the data, it is found that the proportion of the Tianjin's secondary industry in Tianjin is still the highest despite a its decline. Thermal power, iron and steel, petrochemical, cement, building materials, etc., as typical heavy high-pollution emission industries, are the key sources of pollution caused bycausing the air pollution in Tianjin.  The Aanalytical results of the air pollution sources in Hebei Province in the BeijingTianjin-Hebei region show reveal that [17], taking with Shijiazhuang city as an example, regional transmission accounts for 23% to 30% of the PM2.5 sourcespollution, and local pollution emissions accounts for 70% to 77%, of which coal combustion accounts for 28.5%, industrial production accounts for 25.2%, fugitive dust accounts for 22.5%, motor vehicles account for 15.0%, and other sources such as biomass burning, and catering, etc. account for 8.8% of the PM2.5 pollution. Coal combustion is the primary source of the air pollutants in Shijiazhuang and even in Hebei Province. Large The high coal consumption and irrational coal combustion structurestructures are the main causes of soot pollution. In 2014, the three industrial structures in Hebei Province were adjusted from 12.57: 52.5: 34.93 in 2010 in 2010 to 11.7: 51.1: 37.2. The primary industry declined decreased slightly, and the secondary industry decreased by 1.4 %percentage points, and while the tertiary industry increased by approximately 2-3 %percentage pointpoints. The proportion of the secondary industry in Hebei Province is still higher than 50%. This irrational industrial structure makes Hebei Province the region with the worst air pollution in the Beijing-Tianjin-Hebei. In addition, Hebei has a large high proportion of high-pollution and high-energy- consuming consumption industries, including steel, cement, electricity, glass, pharmaceuticals, metallurgy, petrochemicals, and building materials. Among them, the coal consumption in the steel, building materials and power industries accounted for 89.6% of the province's provincial total energy consumption, and the air pollutant emissions in from the steel, cement, power, and glass industries accounted for aboutapproximately 60% of the Hebei's total pollution in Hebei. [18]. As the most important pollution industry in Hebei, steel is the focus of air pollution control.  Causes of Air Pollution in Xinjiang  Landform analysis  Xinjiang is far from the sea, surrounded by mountains and basins, and deserts and grasslands are widely distributed. Taking With the topography of Urumqi as an example, its southeast and west sides are surrounded by mountains with an average elevation of 680 metersm. The slope drop gradient is largehigh, with the south is being high, while the north is low, and the middle is are low. The topographical conditions are very highly detrimental to the atmospheric circulation of the atmosphere and pollutant the diffusion of pollutants. The Urumqi region is located in the northern part of the Tianshan Mountains and the southern edge of the Jungle Basin. It is the city farthest from the ocean in the world. It has a typical desert continental climate and a fragile ecological environment. The vegetation coverage around across the urban area is low, and there are many bare groundsareas, which are liable to form dusty weather conditions.  Small Weak winds  The frequent occurrence of calm and small weak winds in Xinjiang (wind speeds greater higher than or equal to 0.5 m/s are and less lower than 1.5 m/s are characterize small weak winds), which is an important factor for the poor air |
| --- | --- |

|  | environmental quality factor. In the case of small weak winds, the horizontal movement of the atmosphere is slow, which is not conducive to the lateral diffusion of atmospheric pollutants, and it is easy to form regional pollution accumulation[19regional pollution accumulation easily occurs [19-20]. Wind has a certain diffusion and purification ability, and the wind speed and pollutant concentration are basically negatively correlated. Taking With Urumqi as an example, the frequency of static wind atmospheric conditions in the heating period is as high as aboutapproximately 26%, and in December and January, it is as high as 30% or more. Even if there is wind, the wind speed is very small low and rarely exceeds level 3 [21]. Such meteorological conditions are extremely detrimental to the spread of pollutants dispersal. If there are several consecutive days of static wind weather conditions, the pollutants will continue to accumulate, and the air quality will deteriorate sharply [22].  Sand storms  Sandstorms are a typical weather phenomenon in Xinjiang. Sandstorms and floating dust occur throughout the year. Sandstorms and floating dust weather conditions will cause high concentrations of atmospheric particulate matter concentrations, which will seriously affect all the regionsregions’regional air quality in cities [23]. The source region of western China, with the Taklimakan Desert as the main bodyregion, and the source region of northern China, with the Badain Jaran Desert as the center, contributed 70% of the total dust emissions in Asia. The sSandstorms in Northwest China are dominated by dry settlement [24].Regional The regional distribution of the sandstorms in Northwest China is very uneven, while the sandstorm frequency in the northern region of Xinjiang is basically unchanged, and that in the rest of the region is decreasing [25]. Inversion layer  Among the weather types in Urumqi, there are mostly neutral and stable weather types of weather. The frequency of temperature inversion is as high as 90% in winter. The severe air pollution in winter is directly related to thisthese stable meteorological conditions. The frequently occurring inversion layer that occurs frequently is like is similar to a large pot lid, covering the city, making thereby trapping atmospheric pollutants trapped in theat low altitudealtitudes and making them difficult to spreaddisperse. There is a clear positive correlation between inverse temperature inversion and pollutants pollution [22].  Precipitation  Taking Thethe Urumqi region, as an example, it is located in an arid and semiarid-arid environment with little precipitation and large high evaporation. Precipitation has a purifying effect on the air and can effectively purify urban air. There is a clear negative correlation between precipitation and pollutantspollution, which is one of the key factors determining the air quality of air. For the SO2, NO2 and PM10 levels in Urumqi., The the daily average concentration and precipitation are analyzed. The analysis results show that the wet removal ability of rain is greater higher than that of snow. rainfallRainfall and snowfall both have wet removal abilityabilities for these three air pollutants [26],, and the strongest highest removal ability is for S02., This is followed by that for PM10. Dust source  In 2017, with the new area took Turfan City city as as an example of a new area, the emission source of PM10 emission level was 1.169 million tons, and while that of the PM2.5 emissions was 132,000 tons. The source of sSoil dust is the primary source of the particulate emissions in Turpan. Turpan is a sparsely populated area. According to statistics (Turpan Statistical Yearbook (2017)) statistics, the area of land other than urban construction land,and transportation land, water areas and water conservancy facility sites is covers 68,900 square kilometers, with a large area of bare soil. In addition, the Turpan Basin is surrounded by Gobis and deserts. Gobis and deserts account for 76.73% of the area, and there is less vegetation and green land. When windy and sand weather conditions occurs, it is very easy to cause large dust, and frequent dust storms are easily formed. The dust Dust is not only the a source of atmospheric particulate matters, but is also receptors a receptor of particulate matter, resulting in natural dust fall of ground soil dust and sand dust transmission,  contributecontributing greatly to the concentration of particulate matter concentration in the atmosphere [27]. Industrial source  Taking With Turpan Ccity in 2017 as an example, in 2017, the PM10 emissions from industrial sources were 19,200 tons, and the PM2.5 emissions were totaled 4,900 tons,the. The main PM10 contribution sources of PM10 are non-metallicthe nonmetallic |
| --- | --- |

|  | mineral products industry and power thermal power production, accounting for 81.4% and 15.2%, respectively, of the PM10 pollution;. The PM2.5 contribution of the nonmetallicnonmetallic mineral products industry contribution rate is 85.9%, The and the contributions rate of power thermal power production and petroleum processing is are comparable. [27]. Source of life  Taking With Turfan Ccity in 2017 as an example, in 2017, the PM10 emissions of  PM10 in thefrom living sourcesourceshuman activities was 94.58 million tons, and the PM2.5 emission amount of PM2.5 was 73.18 million tons. The main sources of the emissions of atmospheric particulate matter PM10 and PM2.5 in the living sourceterms of human activities were was coal combustion and PM 10 and PM 2.5 emissions. Civilian coal combustion technology is poor, and there isare no scientific and effective emission control measures, and while the amount of fine particulate matter PM2.5 emitted by from coal combustion is even higher than that of emitted from industrial activities. During the heating season, civilian coal combustion and central heating caused a large amount of pollutants to be emitted, and a large number amounts of primary aerosols, heavy metals, and water-soluble ions were are directly emitted directly as primary particulate matter. At the same time, gaseous precursors such as SO2 and NO2 were are released. Adverse weather conditions It is easy to form seasily result in severe pollutioneverely polluted weather under adverse weather conditions. The PM2.5 released from food sources is mainly from the cooking process of involving edible oils and foods. Under high- temperature conditions, particulate matter is formed by chemical reactions such as thermal oxidation and thermal decomposition. Most of the food and beverage sources are occur in densely populated areas, and pollutants are emitted near the ground, which has an important notable impact on the health of the population. According to activity- level survey information, there were a total of 3,443 catering companies in Turpan in 2017. The total PM10 emissions were 301.6 tons, and the total PM2.5 emissions were 241.3 tons. In addition, the exhaust gas from restaurant fumes contains a large number of volatile organic pollutantspollutant VOCs, among which oxygenates and olefins have a high photochemical activity,, which are precursors to form secondary particulate matter, and have and have an importanta notable impact on atmospheric particulate pollution [28].  Mobile source  At present, vehicle exhaust is one of the main sources of the atmospheric particulate matter in China [29-30], especially PM2.5 fine particulate matter PM2.5. Motor vehicles also emit gaseous pollutants such as NOx and VOCs, which are converted into secondary particulate matter through photochemical reactions. Compared with to coarse particles, fine particles have a larger specific surface area, which thus capable of absorbsing and carryingies more toxic substances, which is can be harmful to human health [29-32]. The rRoad sources in Turpan in 2017 in 2017 emitted 249.19 tons of PM10 and 224.5 tons of PM2.5, respectively [33].  OffNonroad-road mobile source  Turpan's The nonroad-road mobile sources in Turpan are mainly divided into three categories: engineering machinery, agricultural machinery and civilian aviation aircraft. Compared with to road mobile sources, nonroad-road mobile sources usually use diesel and heavy oil as their the main fuels. They have the characteristics of a high fuel consumption, long service life, low technical level and low maintenance rate. Most of the pollutants emitted are NOx and particulate matter [34-35].  Journal Requirements:  4. We note that Figures 1-7 in your submission contain satellite images which may be copyrighted. All PLOS content is published under the Creative Commons Attribution License (CC BY 4.0), which means that the manuscript, images, and Supporting Information files will be freely available online, and any third party is permitted to access, download, copy, distribute, and use these materials in any way, even commercially, with proper attribution. For these reasons, we cannot publish previously copyrighted maps or satellite images created using proprietary data, such as Google software (Google Maps, Street View, and Earth). For more information, see our copyright guidelines: http://journals.plos.org/plosone/s/licenses-and-copyright.  We used resources for replacing copyrighted map figures, USGS National Map  Viewer (public domain): http://viewer.nationalmap.gov/viewer/ (page 3, lines 47-49). |
| --- | --- |
| Additional Information: |  |
| Question | Response |

| Financial Disclosure  Enter a financial disclosure statement that describes the sources of funding for the work included in this submission. Review the [submission guidelines](http://journals.plos.org/plosone/s/submission-guidelines#loc-financial-disclosure-statement) for detailed requirements. View published research articles from [PLOS ONE](http://journals.plos.org/plosone/) for specific examples.  This statement is required for submission and will appear in the published article if the submission is accepted. Please make sure it is accurate. | The funders had no role in study design, data collection and analysis, decision to publish, or preparation of the manuscript. |
| --- | --- |
| Unfunded studies Enter: The author(s) received no specific funding for this work. Funded studies Enter a statement with the following details: - Initials of the authors who received each award - Grant numbers awarded to each author - The full name of each funder - URL of each funder website - Did the sponsors or funders play any role in the study design, data collection and analysis, decision to publish, or preparation of the manuscript? - NO - Include this sentence at the end of your statement: The funders had no role in study design, data collection and analysis, decision to publish, or preparation of the manuscript. - YES - Specify the role(s) played. |  |
| * typeset |  |
| Competing Interests  Use the instructions below to enter a competing interest statement for this submission. On behalf of all authors, disclose any [competing interests](http://journals.plos.org/plosone/s/competing-interests) that could be perceived to bias this work—acknowledging all financial support and any other relevant financial or nonfinancial competing interests.  This statement will appear in the published article if the submission is accepted. Please make sure it is | The authors have declared that no competing interests exist. |

| accurate. View published research articles from [PLOS ONE](http://journals.plos.org/plosone/) for specific examples. |  |
| --- | --- |
| NO authors have competing interests Enter: The authors have declared that no competing interests exist. Authors with competing interests Enter competing interest details beginning with this statement: I have read the journal's policy and the authors of this manuscript have the following competing interests: [insert competing interests here] |  |
| * typeset |  |
| Ethics Statement  Enter an ethics statement for this submission. This statement is required if the study involved:   - Human participants - Human specimens or tissue - Vertebrate animals or cephalopods - Vertebrate embryos or tissues - Field research   Write "N/A" if the submission does not require an ethics statement.  General guidance is provided below. Consult the [submission guidelines](http://journals.plos.org/plosone/s/submission-guidelines#loc-guidelines-for-specific-study-types) for detailed instructions. Make sure that all information entered here is included in the Methods section of the manuscript. | "N/A" |

Format for specific study types

Human Subject Research (involving human

participants and/or tissue)

Give the name of the institutional review

board or ethics committee that approved the

study

•

Include the approval number and/or a

statement indicating approval of this

research

•

Indicate the form of consent obtained

(

written/oral) or the reason that consent was

not obtained (e.g. the data were analyzed

anonymously)

•

Animal Research (involving vertebrate

animals, embryos or tissues)

Provide the name of the Institutional Animal

Care and Use Committee (IACUC) or other

relevant ethics board that reviewed the

study protocol, and indicate whether they

approved this research or granted a formal

waiver of ethical approval

•

Include an approval number if one was

obtained

•

If the study involved

non-human primates

,

add

additional details

about animal welfare

and steps taken to ameliorate suffering

•

If anesthesia, euthanasia, or any kind of

animal sacrifice is part of the study, include

briefly which substances and/or methods

were applied

•

Field Research

Include the following details if this study

involves the collection of plant, animal, or

other materials from a natural setting:

Field permit number

•

Name of the institution or relevant body that

granted permission

•

Data Availability

Authors are required to make all data

underlying the findings described fully

available, without restriction, and from the

time of publication. PLOS allows rare

exceptions to address legal and ethical

concerns. See the

[PLOS Data Polic](http://journals.plos.org/plosone/s/data-availability)

[y](http://journals.plos.org/plosone/s/data-availability)

and

[FA](http://journals.plos.org/plosone/s/data-availability#loc-faqs-for-data-policy)

[Q](http://journals.plos.org/plosone/s/data-availability#loc-faqs-for-data-policy)

for detailed information.

Yes - all data are fully available without restriction

| A Data Availability Statement describing where the data can be found is required at submission. Your answers to this question constitute the Data Availability Statement and will be published in the article, if accepted. |  |
| --- | --- |
| Important: Stating ‘data available on request from the author’ is not sufficient. If your data are only available upon request, select ‘No’ for the first question and explain your exceptional situation in the text box. |  |
| Do the authors confirm that all data underlying the findings described in their manuscript are fully available without restriction? |  |
| Describe where the data may be found in full sentences. If you are copying our sample text, replace any instances of XXX with the appropriate details.   - If the data are held or will be held in a public repository, include URLs, accession numbers or DOIs. If this information will only be available after acceptance, indicate this by ticking the box below. For example: All XXX files   are available from the XXX database (accession number(s) XXX, XXX.).   - If the data are all contained within the manuscript and/or Supporting Information files, enter the following: All relevant data are within the manuscript and its Supporting Information files. - If neither of these applies but you are able to provide details of access elsewhere, with or without limitations, please do so. For example:   Data cannot be shared publicly because of [XXX]. Data are available from the XXX Institutional Data Access / Ethics Committee (contact via XXX) for researchers who meet the criteria for access to confidential data.  The data underlying the results presented in the study are available from (include the name of the third party | All relevant data are within the manuscript and its Supporting Information files. |

| and contact information or URL).  • This text is appropriate if the data are owned by a third party and authors do not have permission to share the data.  * typeset |  |
| --- | --- |
| Additional data availability information: |  |

Cover Letter

Dear Dr./Ms./Mr.

I would like to request you to consider the attached manuscript entitled [Study on the Spatiotemporal Characteristics of the Air Pollutants in China from 2014 to 2019 ] for publication in PLOS ONE.

This article is based on hourly data on 7 air pollution parameters (PM2.5, PM10, air quality index (AQI), CO, NO2, O3, and SO2), including mass concentration, for 367 cities in China from 2014 to 2019. The study found that the concentration of the air pollutants in China (PM2.5, PM10, AQI, CO, NO2, and SO2) exhibited a stable spatial distribution and persistence over time, mainly concentrated in Beijing-Tianjin-Hebei and its surrounding areas and Xinjiang from 2014 to 2019 (except for O3, because O3 was primarily concentrated in Inner Mongolia, Qinghai, Gansu and Shandong). The average CO and O3 concentrations monotonically decreased. The spatiotemporal characteristics of the above seven air pollution parameters from 2014 to 2019 supported the abovementioned spatial distribution characteristics of the concentration and the long-term persistence of the atmospheric pollutants in China. Based on the abovementioned spatiotemporal characteristics of the atmospheric pollutants, we analyzed the correlations among them and identified notable correlations. There was a positive correlation among three pollutants, i.e., atmospheric particulate matter PM2.5 and PM10 and the AQI, and there existed a positive correlation among atmospheric particulate matter PM2.5 and PM10, AQI and atmospheric pollution gases NO2 and SO2. There was also a positive correlation between atmospheric pollution gases NO2 and SO2, while the correlation between O3 and every other pollutant was negative. The pollution sources in the

Beijing-Tianjin-Hebei region and Xinjiang were studied.

I believe that the findings of this study are relevant to the scope of your journal and will be of interest to its readership.I have provided tables summarizing the findings.Do let me know if you wish to have a look at them.

This manuscipt has not been published or presented elsewhere in part or in entirety.and is not under consideration by another journal.There are no conflicts of interst to declare.

Amended Funding Statement: the study was unfunded. I did not receive any funding: author(s) received no specific funding for this work. I look forward to hearing from you.

Sincerely

Guo Peng

Manuscript	[Click here to access/download;Manuscript;Manuscript.docx](https://www.editorialmanager.com/pone/download.aspx?id=26278422&guid=db882c1d-a93c-43ed-94d4-952791a8b76f&scheme=1)

1 ***Study on the Spatiotemporal Characteristics of the Air***
2 ***Pollutants in China from 2014 to 2019***
3 Guo Peng ^1¶*^ Umarova Aminat Batalbievna ^1＆^ Luau Yunqi ^1＆^
4 ^1^ Department of Soil, Division of Soil of Physics, Moscow State University, Moscow, 5 Russian Federation

6	E-mail: 956095891@qq.com

7

# 8 Abstract

9 This article is based on hourly data on 7 air pollution parameters (PM2.5, PM10, air
10 quality index (AQI), CO, NO2, O3, and SO2), including mass concentration, for 367
11 cities in China from 2014 to 2019. The study found that the concentration of the air
12 pollutants in China (PM2.5, PM10, AQI, CO, NO2, and SO2) exhibited a stable
13 spatial	distribution	and	persistence	over	time,	mainly	concentrated	in
14 Beijing-Tianjin-Hebei and its surrounding areas and Xinjiang from 2014 to 2019
15 (except for O3, because O3 was primarily concentrated in Inner Mongolia, Qinghai,
16 Gansu and Shandong). The average CO and O3 concentrations monotonically
17 decreased. The spatiotemporal characteristics of the above seven air pollution
18 parameters from 2014 to 2019 supported the abovementioned spatial distribution
19 characteristics of the concentration and the long-term persistence of the atmospheric
20 pollutants in China. Based on the abovementioned spatiotemporal characteristics of

the atmospheric pollutants, we analyzed the correlations among them and identified notable correlations. There was a positive correlation among three pollutants, i.e., atmospheric particulate matter PM2.5 and PM10 and the AQI, and there existed a positive correlation among atmospheric particulate matter PM2.5 and PM10, AQI and atmospheric pollution gases NO2 and SO2. There was also a positive correlation between atmospheric pollution gases NO2 and SO2, while the correlation between O3 and every other pollutant was negative. The pollution sources in the Beijing-Tianjin-Hebei region and Xinjiang were studied.

**Keywords: Atmospheric particulate matter, pollution gas, spatiotemporal change, Beijing-Tianjin-Hebei, Xinjiang**

# Introduction

This article is dedicated to analyzing the data recorded in the era of big data to determine the spatiotemporal characteristics of the air pollution across China. Through quantitative research, we can provide the residents of China with a clear understanding of their living environment. It is hoped that our research can be used to solve certain serious current environmental problems that have attracted much attention and provide scientific solutions to these environmental problems.

The introduction should:

- Provide background that puts the manuscript into context and allows readers outside the field to understand the purpose and significance of the study
- Define the problem addressed and why it is important
- Include a brief review of the key literature
- Note any relevant controversies or disagreements in the field
- Conclude with a brief statement of the overall aim of the work and a comment about whether that aim was achieved

# Materials and methods

In this article, the air pollution parameter data of 367 cities from 2014 to 2019 are sourced from the China National Environmental Monitoring Centre [1]. The website of the China Environmental Monitoring Centre is http://www.cnemc.cn/, and the map of China in this article is obtained from the USGS National Map Viewer

(public	domain)	[3].	The	corresponding	website	is

[http://viewer.nationalmap.gov/viewer/.](http://viewer.nationalmap.gov/viewer/)

The data acquisition address of the China Environmental Monitoring Centre is [http://106.37.208.233:20035/.](http://106.37.208.233:20035/) On this page, you can obtain real-time data on 7 air pollution parameters (PM2.5, PM10, air quality index (AQI), CO, NO2, O3, and SO2), including	the	mass	concentration,	of	367	cities,	or	you	can	go	to

<https://www.zq12369.com/> [2] to acquire real-time historical data from the China Environmental Monitoring Centre.

For an introduction and further details of the monitoring, control and

background points of the data in this article, please refer to the National Ambi ent Air Monitoring Network (Cities Above the Prefecture Level) Setting Plan o f the Ministry of Environmental Protection. The corresponding website address is <http://www.mee.gov.cn/gkml/hbb/bwj/201204/t20120401_250935.htm.> The moni toring points are located in areas with high pollutant concentrations that may a ffect human health and in areas where the main fixed pollution sources notably impact the ambient air quality. The control points are far away from urban b uilt-up areas and major pollution sources, while the background points are loca ted in clean areas not affected by human activities and reflect national-level air quality background levels.

This paper uses ArcGIS software to perform radial basis function (RBF) interpolation on the obtained air pollutant parameter data based on the abovementioned map of China.

This paper uses RBF interpolation. RBF interpolation is conducted to

generate a smooth surface based on the large number of data points. These functions produce good results for gently changing surfaces, such as the elevation. There are a variety of RBFs to choose from in the RBF interpolation method. The selected RBF in this study is a regular spline function. First, the spline function is explained, after which the regular spline function is described. The interpolation method applied via the spline function tool estimates values using a mathematical function minimizing the total curvature of the surface. This produces a smooth surface that only passes through the input points. The algorithm of the spline function tool uses the following surface interpolation equation:

where j = 1, 2, ..., N, N is the number of points, 𝜆_𝑗_ is the coefficient obtained by solving a system of linear equations, and 𝑟_𝑗_ is the distance between point (x, y) and the j-th point.

The definitions of T(x, y) and R(r) will differ depending on the RBF selected. The regular spline function is defined as:

(𝑥, 𝑦) = 𝑎_1_ + 𝑎_2_𝑥 + 𝑎_3_𝑦

where 𝑎_𝑖_ is the coefficient obtained by reconstructing the system of linear equations, and

where R is the distance between the point and the sample, 𝜏^2^ is a weight parameter, 𝐾_𝜊_ is the modified Bessel function, and c is a constant, which is equal to 0.577215.

# Results and Discussions

## Spatiotemporal distribution of PM2.5 across mainland China from 2014 to 2019

Figure 1 shows that from 2014 to 2019, a high PM2.5 mass concentration generally occurred in Beijing-Tianjin-Hebei and its surrounding region (including Shandong, Henan, Shanxi, and Liaoning Provinces) in the eastern part of northern China and in Xinjiang in the western part of northern China. As indicated in

Table 1, the average mass concentration of PM2.5 (unit: μg·m^-3^) from 2014 to 2019 is

44.8, 42.03, 40.7, 35.17, 32.11, and 29.13. The PM2.5 concentration monotonically decreased from 2014 to 2019. As summarized in Table 1-1, the 10 cities with the worst PM2.5 pollution are highlighted among the 367 cities each year. According to the statistics, nearly 5/6 of these highlighted cities are located in Beijing-Tianjin-Hebei and its surrounding areas, and the remainder are located in the Xinjiang region. Xingtai and Baoding in the Beijing-Tianjin-Hebei region of China are cities with high PM2.5 concentrations for 6 consecutive years, while Kashgar and Hotan in the Xinjiang region are cities with high PM2.5 concentrations for 5 consecutive years.

Beijing-Tianjin-Hebei encompasses Beijing, Tianjin, and Hebei Provinces, and the surrounding areas of Beijing-Tianjin-Hebei include the regions bordered by

Beijing, Tianjin, and Hebei, such as Shandong, Henan, Shanxi, and Liaoning Provinces.

**Figure 1: Map of China**

In Figure 0, Beijing-Tianjin-Hebei includes Beijing, Tianjin, and Hebei Provinces, and the surrounding areas of Beijing-Tianjin-Hebei include the regions bordered by Beijing, Tianjin, and Hebei, such as Shandong, Henan, Shanxi, and Liaoning Provinces. Coordinate system: Asia North Albers Equal Area Conic; projection: Albers; datum: WGS 1984; false easting: .0000; false northing: .0000; central meridian: 105.0000; standard parallel 1: 25.0000; standard parallel 2: 48.0000; latitude of origin: .0000; unit: m.

**Figure 1: Spatiotemporal changes in the annual average PM2.5 concentration in**

**China from 2014 to 2019**

**Table 1: Statistical analysis of the PM2.5 mass concentration in China from 2014**

### to 2019

| PM2.5 (μg·m-3) | 2014 | 2015 | 2016 | 2017 | 2018 | 2019 |
| --- | --- | --- | --- | --- | --- | --- |
| Minimum value | 15.8 | 10.82 | 10.51 | 9.67 | 7.01 | 6.43 |
| Maximum value | 115.96 | 117.69 | 156.33 | 99.93 | 116.96 | 106.64 |
| Average value | 44.84 | 42.03 | 40.69 | 35.17 | 32.11 | 29.13 |
| Standard deviation | 9.07 | 12.28 | 15.23 | 11.67 | 12.08 | 10.46 |

### Table 1-1: Top 10 cities and their regions by the annual PM2.5 concentration

| 2014 PM2.5 | | 2015 PM2.5 | | 2016 PM2.5 | | 2017 PM2.5 | | 2018 PM2.5 | | 2019 PM2.5 | |
| --- | --- | --- | --- | --- | --- | --- | --- | --- | --- | --- | --- |
| City | Concentratio n/(μg·m^-3^) | City | Concentratio n/(μg·m^-3^) | City | Concentratio n/(μg·m^-3^) | City | Concentratio n/(μg·m^-3^) | City | Concentration  /(μg·m^-3^) | City | Concentration  /(μg·m^-3^) |
| Baoding (5) | 117 | Kashi area (5) | 118 | Hetian area (5) | 157 | Kashi area (5) | 100 | Hetian area (5) | 117 | Hetian area (5) | 108 |
| Xingtai (6) | 105 | Hetian area (6) | 119 | Kashi area (6) | 158 | Hetian area (5) | 92 | Kashi area (5) | 94 | Laiwu (2) | 106 |
| Handan (4) | 100 | Dezhou (2) | 101 | Shijiazhuang (4) | 94 | Handan (4) | 86 | Akesu area (2) | 74 | Shangqiu (1) | 94 |
| Shijiazhuang (4) | 99 | Xingtai (6) | 101 | Baoding (5) | 92 | Baoding (5) | 84 | Anyang (4) | 69 | Kashi area (5) | 85 |
| Dezhou (2) | 97 | Hengshui (4) | 98 | Akesu area (2) | 91 | Linfen (3) | 82 | Shijiazhuang (4) | 68 | Anyang (4) | 67 |
| Hengshui (4) | 96 | Hetian area (5) | 98 | Hengshui (4) | 87 | Anyang (4) | 82 | Linfen (3) | 67 | Handan (4) | 63 |
| Liaocheng (3) | 94 | Liaocheng (3) | 98 | Xingtai (6) | 87 | Shijiazhuang (4) | 82 | Xingtai (6) | 66 | Xingtai (6) | 62 |
| Laiwu (2) | 90 | Zhengzhou (1) | 95 | Liaocheng (3) | 86 | Xingtai (6) | 81 | Handan (4) | 65 | Luoyang (1) | 60 |
| Tangshan (1) | 90 | Heze (1) | 94 | Jiazuo (1) | 85 | Xianyang (1) | 79 | Baoding (5) | 65 | Linfen (3) | 59 |
| Langfang (1) | 89 | Xinxiang (1) | 93 | Anyang (4) | 85 | Hengshui (4) | 77 | Xuzhou (1) | 62 | Puyang (1) | 59 |

(1), (2), (3), (4), (5), (6): the ranking of the top ten cities from 2014 to 2019; red: the cities in the Beijing-Tianjin-Hebei region; blue: the cities in Xinjiang; green: the surrounding cities of Beijing-Tianjin-Hebei; black: the cities located in the other regions.

Figure 2 reveals that from 2014 to 2019, a high AQI also generally occurred in the Beijing-Tianjin-Hebei region and its surroundings in the eastern part of northern China and in Xinjiang in the western part of northern China. Table 2 demonstrates that the average value of the AQI from 2014 to 2019 is 77.9, 76.8, 73.8, 71.0, 68.1, and 55.0, which indicates a monotonic decline in the AQI from 2014 to 2019. Table 2-1 lists the 10 cities with the highest AQI value from among the 367 cities each year, and these 10 cities are sorted by the AQI value. According to the statistics, nearly 1/2 of these highlighted cities are located in the Beijing-Tianjin-Hebei region and its surrounding areas, and most of the rest are located in Xinjiang.

Baoding, Xingtai, and Handan in the Beijing-Tianjin-Hebei region and Kashgar, Hotan Aksu, and Kezhou in the Xinjiang region have been identified as Chinese cities with high AQIs for 4 to 5 years.

**Figure 2: Spatiotemporal changes in the annual average AQI value in China**

**from 2014 to 2019**

### Table 2: Statistical analysis of the AQI in China from 2014 to 2019

| AQI (μg·m-3) | 2014 | 2015 | 2016 | 2017 | 2018 | 2019 |
| --- | --- | --- | --- | --- | --- | --- |
| Minimum value | 36.06 | 36.4 | 33.62 | 32.36 | 6.84 | 6.9 |
| Maximum value | 161.12 | 200.74 | 204.31 | 187.13 | 216.74 | 183.31 |
| Average value | 77.89 | 76.75 | 73.8 | 71.03 | 68.05 | 54.95 |
| Standard deviation | 12.43 | 21.42 | 23.34 | 19.68 | 25.31 | 20.68 |

### Table 2-1: Top 10 cities and their regions by the AQI value for each year

| 2014 AQI | | 2015 AQI | | 2016 AQI | | 2017 AQI | | 2018 AQI | | 2019 AQI | |
| --- | --- | --- | --- | --- | --- | --- | --- | --- | --- | --- | --- |
| City | AQI | City | AQI | City | AQI | City | AQI | City | AQI | City | AQI |
| Baoding (4) | 162 | Hetian area (5) | 201 | Kashi area (5) | 205 | Hetian area (5) | 187 | Hetian area (5) | 217 | Hetian area (5) | 184 |
| Xingtai (5) | 146 | Kashi area (5) | 198 | Hetian area (5) | 184 | Kashi area (5) | 162 | Kashi area (5) | 180 | Kashi area (5) | 152 |
| Hengshui (3) | 143 | Akesu area (5) | 148 | Akesu area (5) | 155 | Akesu area (5) | 135 | Kezhou area (5) | 155 | Laiwu (1) | 142 |
| Handan (4) | 140 | Baoding (4) | 144 | Kezhou area (5) | 147 | Handan (4) | 126 | Akesu area (5) | 153 | Shangqiu (1) | 134 |
| Shijiazhuang (4) | 138 | Hengshui (3) | 141 | Kuerle (3) | 133 | Anyang (3) | 123 | Tulufan area (4) | 122 | Akesu area (5) | 120 |
| Dezhou (3) | 131 | Dezhou (3) | 139 | Shijiazhuang (3) | 131 | Xingtai (5) | 122 | Kuerle (3) | 121 | Kezhou area (5) | 118 |
| Liaozheng (2) | 130 | Xingtai (5) | 138 | Baoding(4) | 128 | Baoding (4) | 122 | Anyang (3) | 115 | Kuerle (3) | 116 |
| Langfang (1) | 127 | Kezhou area (5) | 137 | Tulufan area (4) | 127 | Shijiazhuang (3) | 122 | Xingtai (5) | 114 | Tulufan area (4) | 112 |
| Tangshan (1) | 126 | Zhengzhou (1) | 135 | Hengshui (3) | 126 | Xianyang (2) | 121 | Handan (4) | 114 | Anyang (3) | 106 |
| Heze (1) | 125 | Liaozheng (2) | 134 | Dezhou (3) | 123 | Kezhou area (5) | 120 | Wujiaqu (1) | 113 | Handan (4) | 102 |

(1), (2), (3), (4), (5), (6): the ranking of the top ten cities from 2014 to 2019; red: the cities in the Beijing-Tianjin-Hebei region; blue: the cities in Xinjiang; green: the surrounding cities of Beijing-Tianjin-Hebei; black: the cities located in the other regions.

In Figure 3, it can be observed that from 2014 to 2019, a high PM10 mass concentration generally occurred in the Beijing-Tianjin-Hebei region and its surrounding areas in the eastern part of northern China and Xinjiang in the western part of northern China. Table 3 indicates that the average PM10 mass concentration (unit: μg·m^-3^) for each year from 2014 to 2019 is 88.51, 88.2, 87.53, 77.96, 82.09, and 66.31, reflecting a monotonic decline in the PM10 concentration. In Table 3-1, 10 cities with the most severe PM10 pollution are identified out of the 367 cities annually, and these 10 cities are then sorted according to the PM10 concentration. According to the statistical analysis results, nearly 1/2 of these identified cities are located in the Beijing-Tianjin-Hebei region and its surroundings, and most of the rest are located in the Xinjiang region. Handan in the Beijing-Tianjin-Hebei region and Kashgar, Hetian, Aksu, and Kezhou in the Xinjiang region in China are identified as cities with high PM10 concentrations for 5 to 6 years.

**Figure 3: Spatiotemporal changes in the annual average PM10 concentration in**

**China from 2014 to 2019**

**Table 3: Statistical analysis of the PM10 mass concentration in China from 2014**

### to 2019

| PM10 (μg·m-3) | 2014 | 2015 | 2016 | 2017 | 2018 | 2019 |
| --- | --- | --- | --- | --- | --- | --- |
| Minimum value | 32.25 | 26.07 | 23.96 | 21.77 | 6.78 | 6.95 |
| Maximum value | 196 | 340.08 | 433.56 | 317.27 | 432.23 | 333.97 |
| Average value | 88.51 | 88.2 | 87.53 | 77.96 | 82.09 | 66.31 |
| Standard deviation | 19.15 | 37.43 | 46.1 | 34.36 | 52.43 | 36.34 |

**Table 3-1: Top 10 cities and their regions by the PM10 concentration annually**

| 2014 PM10 | | 2015 PM10 | | 2016 PM10 | | 2017 PM10 | | 2018 PM10 | | 2019 PM10 | |
| --- | --- | --- | --- | --- | --- | --- | --- | --- | --- | --- | --- |
| City | Concentrati on/(μg·m^-3^) | City | Concentrati on/(μg·m^-3^) | City | Concentrati on/(μg·m^-3^) | City | Concentrati on/(μg·m^-3^) | City | Concentrati on/(μg·m^-3^) | City | Concentrati on/(μg·m^-3^) |
| Baoding (2) | 198 | Hetian area (5) | 341 | Kasha area (5) | 435 | Hetian area (5) | 318 | Hetian area (5) | 432 | Hetian area (5) | 335 |
| Xingtai (2) | 187 | Kasha area (5) | 308 | Hetian area (5) | 283 | Kasha area (5) | 248 | Kasha area (5) | 331 | Kasha area (5) | 229 |
| Hengshui (2) | 176 | Akesu area (5) | 217 | Akesu area (5) | 246 | Akesu area (5) | 198 | Kezhou (5) | 291 | Kezhou (5) | 182 |
| Handan (6) | 174 | Kezhou(5) | 186 | Kezhou (5) | 217 | Tulufan area (4) | 158 | Akesu area (5) | 256 | Akesu area (5) | 181 |
| Shijiazhuang (3) | 172 | Hengshui (2) | 176 | Kuerle (5) | 210 | Handan (6) | 157 | Tulufan area (4) | 180 | Kuerle (5) | 171 |
| Liaocheng(2) | 165 | Baoding (2) | 175 | Tulufan area (4) | 170 | Kezhou (5) | 154 | Kuerle (5) | 171 | Laiwu (1) | 168 |
| Linyi (1) | 163 | Xingtai (1) | 172 | Shijiazhuang (3) | 161 | Shijiazhuang (3) | 153 | Wujiaqu (1) | 136 | Tulufan area (4) | 164 |
| Zibo (1) | 161 | Handan (6) | 167 | Dezhou(2) | 152 | Xingtai (2) | 152 | Handan (6) | 136 | Handan (6) | 124 |
| Jinan (1) | 158 | Zhengzhou (1) | 166 | Handan (6) | 151 | Pingdu (1) | 150 | Xingtai (2) | 136 | Shangqiu (1) | 124 |
| Kuerle (5) | 152 | Dezhou (2) | 165 | Liaocheng (2) | 150 | Kuerle (5) | 142 | Weinan (1) | 135 | Zaozhuang (1) | 117 |

(1), (2), (3), (4), (5), (6): the ranking of the top ten cities from 2014 to 2019; red: the cities in the Beijing-Tianjin-Hebei region; blue: the cities in Xinjiang; green: the surrounding cities of Beijing-Tianjin-Hebei; black: the cities located in the other regions.

In summary, we found that the spatiotemporal distributions of atmospheric particulate matter PM2.5 and PM10 and the AQI are almost the same (mainly concentrated in the Beijing-Tianjin-Hebei region and the surrounding areas and in Xinjiang). Hereafter, we will analyze the correlation between the different particulate matter spatiotemporal distributions below to further verify our point of view.

Figure 4 shows that from 2014 to 2019, higher CO mass concentrations were generally observed in the Beijing-Tianjin-Hebei region and its surrounding areas and Xinjiang. It is worth noting that a high CO mass concentration was observed in Tibet in 2018 and 2019 whereas its concentration reduced 2014 to 2018.

As summarized in Table 4, the average CO concentration (unit: mg·m^-3^) on a yearly basis from 2014 to 2019 is 1.05, 0.99, 0.94, 0.88, 1.19, and 1.09. The CO concentration in 2014 and 2019 did not monotonically decrease (the CO concentration decreased from 2014 to 2018, after which it increased from 2018 to 2019). Table 4-1 10 lists the cities with the most severe CO pollution out of the 367 cities each year, and these 10 cities are then sorted by the CO concentration. According to the statistics, nearly 5/6 of these screened cities are located in Beijing-Tianjin-Hebei and its surrounding region. Linfen and Changzhi in Shanxi Province and Anyang city in Henan Province in China are identified as cities with a high CO concentration for 6 consecutive years.

**Figure 4: Spatiotemporal changes in the annual average CO concentration in**

**China from 2014 to 2019**

**Table 4: Statistical analysis of the CO mass concentration in China from 2014 to**

### 2019

| CO (mg·m-3) | 2014 | 2015 | 2016 | 2017 | 2018 | 2019 |
| --- | --- | --- | --- | --- | --- | --- |
| Minimum value | 0.42 | 0.41 | 0.44 | 0.36 | 0.33 | 0.24 |
| Maximum value | 2.18 | 2.5 | 2.45 | 2.04 | 7.04 | 7.01 |
| Average value | 1.05 | 0.99 | 0.94 | 0.88 | 1.19 | 1.09 |
| Standard deviation | 0.16 | 0.19 | 0.19 | 0.18 | 0.97 | 0.93 |

### Table 4-1: Top 10 cities and their regions by the CO concentration each year

| 2014 CO | | 2015 CO | | 2016 CO | | 2017 CO | | 2018 CO | | 2019 CO | |
| --- | --- | --- | --- | --- | --- | --- | --- | --- | --- | --- | --- |
| City | Concentrati on/(μg·m^-3^) | City | Concentrati on/(μg·m^-3^) | City | Concentrati on/(μg·m^-3^) | City | Concentrati on/(μg·m^-3^) | City | Concentrati on/(μg·m^-3^) | City | Concentrati on/(μg·m^-3^) |
| Penglai (2) | 2.25 | Lvliang (1) | 2.75 | Linfen (6) | 2.52 | Linfen (6) | 2.07 | Linfen (6) | 1.76 | Laiwu (1) | 1.53 |
| Linfen (6) | 2.20 | Linfen (6) | 2.36 | Tangshan (6) | 2.27 | Jincheng (4) | 1.98 | Tangshan (6) | 1.69 | Linfen (6) | 1.46 |
| Binzhou (2) | 2.13 | Tangshan (6) | 2.13 | Anyang (5) | 2.17 | Tangshan (6) | 1.96 | Panzhihua (2) | 1.53 | Tangshan (6) | 1.37 |
| Zibo (4) | 2.11 | Zibo (4) | 2.11 | Yuncheng (3) | 2.06 | Anyang (5) | 1.85 | Anyang (5) | 1.53 | Datong (2) | 1.35 |
| Benxi (1) | 2.09 | Binzhou (2) | 2.10 | Jincheng (4) | 2.00 | Dekehasake (2) | 1.83 | Dekehasake (2) | 1.46 | Changzhi (5) | 1.25 |
| Tangshan (6) | 2.06 | Naqu area (1) | 2.09 | Luoyang (1) | 2.00 | Zibo (4) | 1.76 | Loudi (1) | 1.45 | Ganzhou (2) | 1.24 |
| Changzhi (5) | 2.05 | Anyang (5) | 2.06 | Changzhi (5) | 2.00 | Yuncheng (3) | 1.71 | Datong (2) | 1.42 | Jincheng (4) | 1.23 |
| Liaocheng (1) | 2.00 | Zhoukou (1) | 2.02 | Hebi (2) | 1.96 | Hebi (2) | 1.68 | Ganzhou (2) | 1.42 | Anyang (5) | 1.21 |
| Yanan (1) | 1.93 | Yuncheng (3) | 1.96 | Zibo (4) | 1.90 | Changzhi (5) | 1.67 | Xining (1) | 1.41 | Panzhihua (2) | 1.20 |
| Baoding (1) | 1.93 | Changzhi (5) | 1.96 | Yilihasake (1) | 1.85 | Xingtai | 1.67 | Jincheng(4) | 1.38 | Penglai (2) | 1.19 |

(1), (2), (3), (4), (5), (6): the ranking of the top ten cities from 2014 to 2019; red: the cities in the Beijing-Tianjin-Hebei region; blue: the cities in Xinjiang; green: the surrounding cities of Beijing-Tianjin-Hebei; black: the cities located in the other regions.

In Figure 5, it is observed that from 2014 to 2019, a higher NO2 mass concentration generally occurred in the Beijing-Tianjin-Hebei region and its surroundings in the eastern part of northern China and in Xinjiang in the western part of northern China. Table 5 lists the average values of the NO2 mass concentration (unit: μg·m^-3^) for each year from 2014 to 2019, namely, 31.2, 23.51, 23.97, 23.95, 21.29, and 20.17. The NO2 concentration in 2014 and 2019 exhibited a monotonous downward trend. As summarized in Table 5-1, the 10 cities with the most severe NO2 pollution are identified out of the 367 cities on an annual basis, and these 10 cities are then sorted according to the NO2concentration. According to the statistics, nearly 2/3 of the identified cities are located in the Beijing-Tianjin-Hebei region and its surrounding areas. Baoding and Tangshan in the Beijing-Tianjin-Hebei area in China have been identified as cities with the highest NO2 concentration for 6 consecutive years. Zhengzhou in Henan Province bordering the Beijing-Tianjin-Hebei area in

China has been identified as having a high NO2 concentration for 5 years.

A high NO2 concentration is observed in areas where the economy is developed and the vehicle population is large (for example, in the Yangtze River Delta such as Shanghai, in the Pearl River Delta such as Guangzhou, and in the Sichuan Basin such as Chongqing, which are all economically developed areas). Motor vehicle exhaust is a major source of [29-30]. This explains why the spatiotemporal distribution of NO2 is slightly different from those of atmospheric particulate matter PM2.5 and PM10 and AQI.

**Figure 5: Spatiotemporal changes in the annual average NO2 concentration in**

**China from 2014 to 2019**

**Table 5: Statistical analysis of the NO2 mass concentration in China from 2014 to**

### 2019

| NO2 (μg·m-3) | 2014 | 2015 | 2016 | 2017 | 2018 | 2019 |
| --- | --- | --- | --- | --- | --- | --- |
| Minimum value | 12.31 | 7.72 | 8.96 | 6.84 | 6.99 | 6.92 |
| Maximum value | 56.49 | 59.19 | 60.37 | 58.08 | 53.85 | 50.35 |
| Average value | 31.2 | 23.51 | 23.97 | 23.95 | 21.29 | 20.17 |
| Standard deviation | 4.3 | 6.09 | 5.8 | 6.45 | 6.21 | 5.68 |

### Table 5-1: Top 10 cities and their regions by the NO2 concentration annually

| 2014 NO2 | | 2015 NO2 | | 2016 NO2 | | 2017 NO2 | | 2018 NO2 | | 2019 NO2 | |
| --- | --- | --- | --- | --- | --- | --- | --- | --- | --- | --- | --- |
| City | Concentrati on/(μg·m^-3^) | City | Concentrati on/(μg·m^-3^) | City | Concentrati on/(μg·m^-3^) | City | Concentrati on/(μg·m^-3^) | City | Concentrati on/(μg·m^-3^) | City | Concentrati on/(μg·m^-3^) |
| Zibo (3) | 65 | Zibo (3) | 61 | Xingtai (6) | 61 | Tangshan (6) | 58 | Huzhou (3) | 54 | Laiwu (2) | 51 |
| Tangshan (6) | 58 | Tangshan (6) | 60 | Tangshan (6) | 58 | Xian (3) | 58 | Tangshan (6) | 54 | Tangshan (6) | 49 |
| Linyi (1) | 57 | Xingtai (6) | 59 | Baoding (3) | 57 | Xingtai (6) | 56 | Xian (3) | 53 | Yunlin (1) | 47 |
| Xingtai (6) | 56 | Zhengzhou (5) | 55 | Handan (2) | 54 | Huzhou (3) | 55 | Changzhou (1) | 49 | Xian (3) | 46 |
| Jinan (3) | 53 | Baoding (3) | 53 | Huzhou (3) | 54 | Xianyang (2) | 53 | Tianjin (2) | 49 | Taiyuan (2) | 44 |
| Baoding (3) | 52 | Linxiazhou (1) | 53 | Shijiazhuang (2) | 54 | Tianjin (2) | 53 | Xingtai (6) | 47 | Lvliang (1) | 44 |
| Beijing (1) | 52 | Suzhou (2) | 51 | Zhengzhou (5) | 53 | Zhengzhou (5) | 52 | Xinxiang (2) | 47 | Huzhou(2) | 44 |
| Suzhou (2) | 51 | Jinan (3) | 51 | Zibo (3) | 52 | Weinan (1) | 51 | Xianyang (2) | 47 | Jinan (3) | 43 |
| Laiwu (2) | 51 | Zhuji (1) | 51 | Hebi (1) | 52 | Handan (2) | 51 | Taiyuan (2) | 46 | Xingtai (6) | 42 |
| Qinhuangdao (2) | 50 | Xinxiang (2) | 51 | Wulumuqi (1) | 52 | Shijiazhuang (2) | 50 | Zhengzhou (5) | 46 | Zhengzhou (5) | 42 |

(1), (2), (3), (4), (5), (6): the ranking of the top ten cities from 2014 to 2019; red: the cities in the Beijing-Tianjin-Hebei region; blue: the cities in Xinjiang; green: the surrounding cities of Beijing-Tianjin-Hebei; black: the cities located in the other regions.

Figure 6 highlights that a higher O3 mass concentration has stably occurred in Inner Mongolia, Qinghai, and Gansu in northern China from 2014 to 2019, as supported by the data in Table 6. The average O3 mass concentration (unit: μg·m^-3^) from 2014 to 2019 is 59.42, 59.12, 59.98, 67.82, 62.53, 61.67. From 2014 to 2019, the O3 concentration fluctuated and only slightly increased in 2017. Table 6-1 identifies the 10 cities with the most severe O3 pollution out of the 367 cities each year, and these 10 cities are then sorted by the O3 concentration. According to the statistics, more than 1/3 of these identified cities are located in the Inner Mongolia, Qinghai, and Gansu regions in northern China. Haibei Prefecture in Qinghai and Alxa in Inner Mongolia in China have been identified as cities with a high O3 concentration for 4 years.

In addition to the more uniform distribution of the O3 concentration in Inner

Mongolia, Qinghai, and Gansu in northwestern China, a O3 concentration was observed in Shandong Province from 2014 to 2019.

The O3 spatiotemporal distribution is significantly different from those of the other air pollution parameters studied in this paper. O3 is a secondary pollutant formed from volatile organic compounds (VOCs) nitrogen oxides (NOx) [37]. It is not surprising that it has a different spatial distribution as other pollutants.

**Figure 6: Spatiotemporal changes in the annual average O3 concentration in China from 2014 to 2019**

**Table 6: Statistical analysis of the O3 mass concentration in China from 2014 to**

### 2019

| O3 (μg·m-3) | 2014 | 2015 | 2016 | 2017 | 2018 | 2019 |
| --- | --- | --- | --- | --- | --- | --- |
| Minimum value | 30.83 | 28.42 | 32.47 | 37.92 | 7.76 | 6.93 |
| Maximum value | 82.33 | 92.65 | 95.03 | 103.52 | 103.01 | 103.05 |
| Average value | 59.42 | 59.12 | 59.98 | 67.82 | 62.53 | 61.67 |
| Standard deviation | 4.83 | 6.66 | 6.78 | 8.38 | 10.11 | 11.98 |

### Table 6-1: Top 10 cities and regions by the O3 concentration each year

| 2014 O3 | | 2015 O3 | | 2016 O3 | | 2017 O3 | | 2018 O3 | | 2019 O3 | |
| --- | --- | --- | --- | --- | --- | --- | --- | --- | --- | --- | --- |
| City | Concentrati on/(μg·m^-3^) | City | Concentrati on/(μg·m^-3^) | City | Concentrati on/(μg·m^-3^) | City | Concentrati on/(μg·m^-3^) | City | Concentrati on/(μg·m^-3^) | City | Concentrati on/(μg·m^-3^) |
| Rongcheng(3) | 93 | Haixizhou (3) | 97 | Haibeizhou (4) | 95 | Enshizhou (1) | 37 | Alashanmeng (4) | 105 | Haixizhou (3) | 104 |
| Weihai(4) | 92 | Alashanmeng (4) | 94 | Alashanmeng (4) | 95 | Xiangxi (1) | 38 | Guoluozhou (3) | 90 | Alashanmeng (4) | 102 |
| Penglai(1) | 87 | Hainanzhou (3) | 94 | Hainanzhou (3) | 83 | Qiandongnanzhou (1) | 38 | Kezhou (1) | 89 | Haibeizhou (4) | 96 |
| Linan(1) | 85 | Haibeizhou (4) | 90 | Yixing (1) | 82 | Xishuangbanna (1) | 40 | Weihai (4) | 89 | Weihai (4) | 92 |
| Jiaonan(2) | 82 | Yanzheng (1) | 82 | Guoluozhou (3) | 81 | Qianxinanzhou (1) | 40 | Haixizhou (3) | 88 | Hainanzhou (3) | 89 |
| Weifang(1) | 81 | Jiayuguan (2) | 81 | Yingkou (2) | 81 | Nujiang (1) | 41 | Jiaozuo (2) | 87 | Jiaozuo (2) | 89 |
| Wendeng(1) | 81 | Guoluozhou (3) | 81 | Jiayuguan (2) | 81 | Chongqing (1) | 42 | Haibeizhou (4) | 87 | Rongcheng (3) | 89 |
| Dongying(1) | 80 | Yingkou (2) | 80 | Eerduosi (2) | 80 | Wuzhou (1) | 42 | Rongcheng (3) | 85 | Jinchang (1) | 86 |
| Eerduosi(2) | 80 | Lasa (1) | 80 | Jiaonan (1) | 80 | Hechi (1) | 43 | Qingyang (1) | 85 | Changzhi (2) | 85 |
| Qingdao(1) | 80 | Weihai (4) | 79 | Dalian (1) | 79 | Pingxiang (1) | 43 | Changzhi (2) | 84 | Jincheng (1) | 84 |

(1), (2), (3), (4), (5), (6): The ranking of the top ten cities from 2014 to 2019; red: the cities in Qinghai; green: the cities in Shandong; black: the cities located in the other regions.

Figure 7 reveals that from 2014 to 2019, a higher SO2 mass concentration was generally observed in the Beijing-Tianjin-Hebei region and its surrounding areas in the eastern part of northern China and in Xinjiang in the northwest of mainland China.

Table 7 lists the average SO2 mass concentrations (unit: μg·m^-3^) for each year from 2014 to 2019, i.e., 24.43, 20.02, 17.01, 14.67, 11.59, and 9.47. The SO2 concentration in 2014 and 2019 exhibited a monotonous downward trend. In Table 7-1, the 10 cities with the most severe SO2 pollution are highlighted from among the 367 cities each year, and these 10 cities are then sorted according to the SO2 concentration. According to the statistics, more than 1/2 of these highlighted cities are in the Shanxi region bordering the Beijing-Tianjin-Hebei to the west in northern China, while nearly 1/6 of the cities are in the Shandong region to the east of Beijing-Tianjin-Hebei. In the Shanxi region in China, Linfen has been identified as having a high SO2 concentration for 6 consecutive years.

The temporal and spatial distributions of pollution gas SO2, atmospheric particulate matter PM2.5 and PM10, AQI, and pollution gases SO2 and NO2 are almost the same (primarily centered in Beijing-Tianjin-Hebei and its surrounding areas and in Xinjiang), which we will examine below.

**Figure 7: Spatiotemporal changes in the annual average SO2 concentration in**

**China from 2014 to 2019**

**Table 7: Statistical analysis of the SO2 mass concentration in China from 2014 to**

### 2019

| SO2 (μg·m-3) | 2014 | 2015 | 2016 | 2017 | 2018 | 2019 |
| --- | --- | --- | --- | --- | --- | --- |
| Minimum value | 2.23 | 2.81 | 17.01 | 2.37 | 3.19 | 3.36 |
| Maximum value | 83.3 | 75.61 | 87.08 | 82.42 | 47.56 | 36.92 |
| Average value | 24.43 | 20.02 | 17.01 | 14.67 | 11.59 | 9.47 |
| Standard deviation | 8.46 | 8.47 | 7.55 | 6.21 | 4.05 | 3.18 |

**Table 7-1: Top 10 cities and their regions by the SO2 concentration on a yearly**

### basis

| 2014 SO2 | | 2015 SO2 | | 2016 SO2 | | 2017 SO2 | | 2018 SO2 | | 2019 SO2 | |
| --- | --- | --- | --- | --- | --- | --- | --- | --- | --- | --- | --- |
| City | Concentrati on/(μg·m^-3^) | City | Concentrati on/(μg·m^-3^) | City | Concentrati on/(μg·m^-3^) | City | Concentrati on/(μg·m^-3^) | City | Concentrati on/(μg·m^-3^) | City | Concentrati on/(μg·m^-3^) |
| Zibo (2) | 108 | Zibo (2) | 82 | Jinzhong (4) | 87 | Jinzhong (4) | 83 | Linfen (6) | 48 | Baiyin (2) | 39 |
| Yangquan (3) | 85 | Shuozhou (3) | 76 | Linfen (6) | 82 | Linfen (6) | 79 | Baiyin (2) | 42 | Wuhai (4) | 33 |
| Laiwu (2) | 81 | Hetiandiqu (1) | 72 | Shuozhou (3) | 67 | Lvliang (5) | 68 | Lvliang (5) | 39 | Laiwu (2) | 32 |
| Taiyuan (4) | 66 | Jinzhong (4) | 72 | Shijiazui (5) | 67 | Shijiazui (5) | 51 | Panzhihua (2) | 38 | Shuozhou (3) | 29 |
| Jining (1) | 64 | Shijiazui (5) | 71 | Jincheng (1) | 66 | Taiyuan (4) | 51 | Jinzhou(2) | 37 | Panzhihua (2) | 29 |
| Dongying (1) | 64 | Taiyuan (4) | 68 | Yuncheng (2) | 66 | Yinchuang (2) | 51 | Shijiazui (5) | 37 | Xinzhou (2) | 29 |
| Zaozhuang (1) | 63 | Lvliang (5) | 68 | Taiyuan(4) | 65 | Yuncheng (2) | 51 | Jinzhong (4) | 36 | Lvliang (5) | 29 |
| Linfen (6) | 63 | Zhangqiu (1) | 64 | Lvliang (5) | 62 | Wuhai (4) | 51 | Yinchuang (2) | 36 | Datong (1) | 28 |
| Shijiazui (5) | 59 | Linfen (6) | 63 | Yangquan (3) | 61 | Yangquan (3) | 49 | Huludao (1) | 36 | Linfen (6) | 27 |
| Shenyang (1) | 57 | Wuhai (4) | 62 | Changzhi (1) | 61 | Xinzhou (2) | 48 | Wuhai (4) | 34 | Jinzhou (2) | 27 |

(1), (2), (3), (4), (5), (6): the ranking of the top ten cities from 2014 to 2019; red: the cities in the Beijing-Tianjin-Hebei region; blue: the cities in Xinjiang; green: the surrounding cities of Beijing-Tianjin-Hebei; black: the cities located in the other regions.

## Correlation Between PM2.5, PM10 and AQI

There is often a significant correlation between the mass concentrations of different types of particulate matter. Table 8 summarizes the correlation between the hourly PM2.5 and PM10 mass concentrations from 2014 to 2019. The ratio of PM10 to PM2.5 is 1.24 and the correlation coefficient R^2^ is 0.77. The mass concentration ratio of PM2.5 to PM10 is 0.46, . There is a particularly notable linear positive correlation between the two types of particulate matter, which indicates that they cause similar changes in ambient air pollution and practically follow the same migration and transformation laws. This is related to Liu Jie's research on the correlation of atmospheric particulates, and the results are consistent [4-5].

**Table 8: Correlation between the particulate matter and AQI levels from**

### 2014 to 2019

| Between particles | Fitting equation | Correlation coefficient R | Goodness of fit  R2 | Degrees of freedom n-2 | Significance level | | Mass concentration ratio |
| --- | --- | --- | --- | --- | --- | --- | --- |
|  |  |  |  |  | R_0_.05 | R_0_.01 |  |
| x: PM2.5;y: AQI | y = 1.24x + 23.96 | 0.91 | 0.83 | 50638 | 0.062 | 0.081 | - |
| x: PM2.5;y: PM10 | y = 1.24x + 35.63 | 0.88 | 0.77 | 50638 | 0.062 | 0.081 | 0.46 |
| x: AQI;y: PM10 | y = 0.98x + 12.55 | 0.95 | 0.91 | 50638 | 0.062 | 0.081 | - |

Correlation coefficient R: the Pearson product-moment correlation

coefficient is adopted to measure the correlation between two variables X and Y (linear correlation), whose value is between -1 and 1, and the closer the value of R is to 1, the more positive the correlation is; the goodness of fit: how well the regression line fits the observations. The statistic for measuring the goodness of fit is the determination coefficient R², and the maximum value of R² is 1. The closer the value of R² is to 1, the better the regression line fits the observed data. The smaller the value, the worse the regression line fits the observed data. The degrees of freedom: this refers to the number of unrestricted conditions or variables, and when calculating a certain statistic, usually, df = n-k, where n is the number of samples and k is the number of restricted conditions or variables; significance level: estimate of the overall probability that the parameter falls within a certain interval; mass concentration ratio: the ratio of PM2.5 to PM10 (PM2.5 are particles with an equivalent air dynamic diameter of 2.5 microns or smaller, while PM10 is smaller than 10 microns in diameter, and PM10 includes PM2.5).

## Correlation Between the Atmospheric PM2.5 Gaseous Pollutants

From the correlation study between the particulate matter and gaseous

pollutants, as summarized in Table 9, there is a significant linear positive correlation between xxx and xx, xx and xx and xx and xx which are 0.97, 0.81, and 0.88, respectively,. The correlation coefficient between the atmospheric particulate matter (PM2.5 and PM10), AQI and NO2 is 0.82, 0.62, and 0.72, respectively. The above results are also consistent with the findings of Li et al. [6]. The atmospheric particulate matter (PM2.5 and PM10) are positively correlated with the atmospheric pollution gases NO2 and SO2, which usually occurs due to the mutual conversion caused by the chemical reaction between the particulate matter and the gaseous pollutants [7- 8]. It was found that nitrate (sulfate) produced by NO2 (SO2) would be transformed into secondary particulate matter (PM2.5 and PM10) over time, and with increasing NO2 and SO2 atmospheric pollution, the atmospheric particulate matter PM2.5 and PM10 also increased. One of the reasons for the increase in atmospheric pollutants NO2 and SO2 is the large vehicle flows and high exhaust emissions [9-10].

**Table 9: Correlation between the particulate matter and gaseous pollutant**

### concentrations from 2014 to 2019

| Particulate matter and gaseous pollutants | Fitting equation | Correlation coefficient R | Goodness of fit  R2 | Degrees of freedom n-2 | Significance level | |
| --- | --- | --- | --- | --- | --- | --- |
|  |  |  |  |  | R_0_.05 | R_0_.01 |
| x: CO;y: PM2.5 | y = -21.83x + 59.67 | -0.39 | 0.16 | 50638 | 0.062 | 0.081 |
| x: CO;y: AQI | y = -29.37x + 100.47 | -0.39 | 0.15 | 50638 | 0.062 | 0.081 |
| x: CO;y: PM10 | y = -15.37x + 97.50 | -0.20 | 0.04 | 50638 | 0.062 | 0.081 |
| x: NO2;y: PM2.5 | y = 1.31x + 5.78 | 0.82 | 0.68 | 50638 | 0.062 | 0.081 |
| x: NO2;y: AQI | y = 1.57x + 32.74 | 0.72 | 0.52 | 50638 | 0.062 | 0.081 |
| x: NO2;y: PM10 | y = 1.40x + 48.14 | 0.62 | 0.39 | 50638 | 0.062 | 0.081 |
| x: O3;y: PM2.5 | y = -0.99x + 98.38 | -0.52 | 0.27 | 50638 | 0.062 | 0.081 |
| x: O3;y: AQI | y = -0.70x + 113.51 | -0.27 | 0.07 | 50638 | 0.062 | 0.081 |
| x: O3;y: PM10 | y = -1.22x + 156.95 | -0.46 | 0.21 | 50638 | 0.062 | 0.081 |
| x: SO2;y: PM2.5 | y = 1.09x + 19.73 | 0.97 | 0.95 | 50638 | 0.062 | 0.081 |
| x: SO2;y: AQI | y = 1.33x + 48.81 | 0.88 | 0.77 | 50638 | 0.062 | 0.081 |
| x: SO2;y: PM10 | y = 1.27x + 61.17 | 0.81 | 0.66 | 50638 | 0.062 | 0.081 |

## Correlation Between Gaseous Pollutants

There is a particularly significant linear positive correlation between NO_2_ and SO_2_ (a correlation coefficient of 0.90). NO2 and SO2 are mainly from coal combustion and motor vehicle emissions. There is no correlation between O3 and the other three gaseous pollutants [12].

**Table 10: Correlation analysis of the gaseous pollutants from 2014 to 2019**

| Between gaseous pollutants | Fitting equation | Correlation coefficient R | Goodness of fit  R2 | Degrees of freedom n-2 | Significance level | |
| --- | --- | --- | --- | --- | --- | --- |
|  |  |  |  |  | R_0_.05 | R_0_.01 |
| x: CO;y: NO2 | y = -7.95x + 32.15 | -0.23 | 0.05 | 50638 | 0.062 | 0.081 |
| x: CO;y: O3 | y = -9.30x + 71.28 | -0.32 | 0.10 | 50638 | 0.062 | 0.081 |
| x: CO;y: SO2 | y = -15.81x + 32.38 | -0.32 | 0.10 | 50638 | 0.062 | 0.081 |
| x: NO2;y: O3 | y = -0.24x + 67.44 | -0.28 | 0.08 | 50638 | 0.062 | 0.081 |
| x:NO2;y: SO2 | y = 1.29x - 14.82 | 0.90 | 0.81 | 50638 | 0.062 | 0.081 |
| x: O3;y: SO2 | y = -0.81x + 66.36 | -0.48 | 0.23 | 50638 | 0.062 | 0.081 |

## Causes of Air Pollution in the Beijing-Tianjin-Hebei Region

The unique geographical environment of the Beijing-Tianjin-Hebei region coupled with the rapid development of urban agglomerations and the local atmospheric circulation formation conditions result in the air pollution in the Beijing-Tianjin-Hebei region [13]. In 2014, the energy consumption level of Beijing-Tianjin-Hebei accounted for 12% of the total energy consumption level of China. There are still numerous high-energy consumption heavy industries in the Beijing-Tianjin-Hebei region. Compared to 2010, the overall GDP increased by 52% in 2014. The magnitude of the consumption in China of the accumulated resources per unit of land area over time and the intensity of pollutant emissions are very high [14].

In the Beijing-Tianjin-Hebei region, Beijing is selected as an example. The analysis results of the air pollution sources in Beijing released in 2014 [15] reveal that approximately 28% to 36% of the atmospheric PM2.5 come from regional transport. Local emissions account for approximately 64%-72%, including 31.1% from motor vehicles, 22.4% from coal, 18.1% from industrial production, 14.3% from dust and 14.1% from other activities, such as catering, livestock and poultry breeding, and automobile repair. Vehicle exhaust is the most important source of local pollution. The structures of the primary, secondary and tertiary industries in Beijing were adjusted from 0.9:24.0:75.1 in 2010 to 0.7: 21.4:77.9 in 2014. The tertiary industry dominates, and compared to 2010, its proportion has increased by nearly 3%, indicating that the industrial structure of Beijing is still being optimized. The air pollution is mainly caused by the industrial production process of the secondary industry and vehicle exhaust emissions.

The primary industry mainly refers to those industries that produce foodstuffs and other biological materials, including crops, forestry, animal husbandry, aquaculture and other industries that directly target natural objects. The secondary industry mainly refers to the processing and manufacturing industry, which consumes the basic materials provided by nature and the primary industry during processing. The tertiary industry refers to those industries other than the primary and secondary industries and has a wide range, mainly including nonmaterial production sectors such as transportation, communications, commerce, catering, finance, education, and public services.

The results of a new round of PM2.5 source analysis completed by the Beijing Environmental Protection Monitoring Center in 2018 were released to the public, and the main conclusions of this study indicate that 2/3 of the main sources of PM2.5 in Beijing throughout the year are local and 1/3 are regional transmissions. From the characteristics of the current local atmospheric PM2.5 sources in Beijing, in terms of the contribution of local emissions, mobile sources account for 45%, fugitive dust sources account for 16%, industrial sources account for 12%, living surface sources account for 12%, coal sources account for 3%, and agricultural, natural and other sources account for approximately 12% of the PM2.5 concentration. The results show that at this stage, the coal-fired source has basically stopped being the main source of atmospheric PM2.5 in Beijing [58].

In the Beijing-Tianjin-Hebei region, Tianjin is selected as an example. The analysis results of the air pollution sources in Tianjin in 2014 showed [16] that the regional transmission of atmospheric PM2.5 sources accounted for 22%-34% of the pollution. The remaining 66% to 78% came from local emissions. Among them, dust sources accounted for 30%, coal-fired sources accounted for 27%, motorized sources accounted for 20%, industrial production sources accounted for 17%, and other sources accounted for 6%. Dust is the primary source of pollution, followed by coal combustion and motor vehicles. The roads, storage yards, construction sites, etc. in Tianjin are the main sources of dust. In addition, construction projects in the railway, chemical, and power industries will also generate dust. In 2014, the three industrial structures in Tianjin were adjusted from 1.6:53.1:45.3 in 2010 to 1.3:49.4:49.3. The secondary industry decreased 3.7%, and the tertiary industry increased 4%. Until 2014, the proportions of the secondary and tertiary industries in Tianjin practically remained stable. In comparing the data, it is found that the proportion of the secondary industry in Tianjin is still the highest despite its decline. Thermal power, iron and steel, petrochemical, cement, building materials, etc., as typical high-pollution emission industries, are the key sources causing the air pollution in Tianjin.

The analytical results of the air pollution sources in Hebei Province in the Beijing-Tianjin-Hebei region reveal that [17], with Shijiazhuang city as an example, regional transmission accounts for 23% to 30% of the PM2.5 pollution, and local emissions account for 70% to 77%, of which coal combustion accounts for 28.5%, industrial production accounts for 25.2%, fugitive dust accounts for 22.5%, motor vehicles account for 15.0%, and other sources such as biomass burning and catering account for 8.8% of the PM2.5 pollution. Coal combustion is the primary source of the air pollutants in Shijiazhuang and even in Hebei Province. The high coal consumption and irrational coal combustion structure are the main causes of soot pollution. In 2014, the three industrial structures in Hebei Province were adjusted from 12.57:52.5:34.93 in 2010 to 11.7:51.1:37.2. The primary industry decreased slightly, and the secondary industry decreased 1.4%, while the tertiary industry increased approximately 2-3%. The proportion of the secondary industry in Hebei Province is still higher than 50%. This irrational industrial structure makes Hebei Province the region with the worst air pollution in the Beijing-Tianjin-Hebei. In addition, Hebei has a high proportion of high-pollution and high-energy consumption industries, including steel, cement, electricity, glass, pharmaceuticals, metallurgy, petrochemicals, and building materials. Among them, the coal consumption in the steel, building materials and power industries accounted for 89.6% of the provincial total energy consumption, and the air pollutant emissions from the steel, cement, power, and glass industries accounted for approximately 60% of the total pollution in Hebei [18]. As the most important pollution industry in Hebei, steel is the focus of air pollution control.

## Causes of Air Pollution in Xinjiang

### Landform analysis

Xinjiang is far from the sea, surrounded by mountains and basins, and

deserts and grasslands are widely distributed. With the topography of Urumqi as an example, its southeast and west sides are surrounded by mountains with an average elevation of 680 m. The slope gradient is high, with the south being high, while the north and the middle are low. The topographical conditions are highly detrimental to the atmospheric circulation and pollutant diffusion. The Urumqi region is located in the northern part of the Tianshan Mountains and the southern edge of the Jungle Basin. It is the city farthest from the ocean in the world. It has a typical desert continental climate and a fragile ecological environment. The vegetation coverage across the urban area is low, and there are many bare areas, which are liable to form dusty weather conditions.

### Weak winds

The frequent occurrence of calm and weak winds in Xinjiang (wind speeds higher than or equal to 0.5 m/s and lower than 1.5 m/s characterize weak winds) is an important factor for the poor air environmental quality. In the case of weak winds, the horizontal movement of the atmosphere is slow, which is not conducive to the lateral diffusion of atmospheric pollutants, and regional pollution accumulation easily occurs [19-20]. Wind has a certain diffusion and purification ability, and the wind speed and pollutant concentration are basically negatively correlated. With Urumqi as an example, the frequency of static atmospheric conditions in the heating period is as high as approximately 26%, and in December and January, it is as high as 30% or more. Even if there is wind, the wind speed is very low and rarely exceeds level 3

[21]. Such meteorological conditions are extremely detrimental to pollutant dispersal. If there are several consecutive days of static weather conditions, pollutants will continue to accumulate, and the air quality will deteriorate sharply [22].

### Sand storms

Sandstorms are a typical weather phenomenon in Xinjiang. Sandstorms and floating dust occur throughout the year. Sandstorms and floating dust weather conditions will cause high atmospheric particulate matter concentrations, which will seriously affect the regional air quality in cities [23]. The source region of western China, with the Taklimakan Desert as the main region, and the source region of northern China, with the Badain Jaran Desert as the center, contributed 70% of the total dust emissions in

Asia. The sandstorms in Northwest China are dominated by dry settlement [24]. The regional distribution of the sandstorms in Northwest China is very uneven, while the sandstorm frequency in the northern region of Xinjiang is basically unchanged, and that in the rest of the region is decreasing [25].

### Inversion layer

Among the weather types in Urumqi, there are mostly neutral and stable weather types. The frequency of temperature inversion is as high as 90% in winter. The severe air pollution in winter is directly related to these stable meteorological conditions. The frequently occurring inversion layer is similar to a large pot lid covering the city, thereby trapping atmospheric pollutants at low altitudes and making them difficult to disperse. There is a clear positive correlation between temperature inversion and pollution [22].

### Precipitation

The Urumqi region, as an example, is located in an arid and semiarid environment with little precipitation and high evaporation. Precipitation has a purifying effect on the air and can effectively purify urban air. There is a clear negative correlation between precipitation and pollution, which is one of the key factors determining the air quality. For the SO2, NO2 and PM10 levels in Urumqi, the daily average concentration and precipitation are analyzed. The analysis results show that the wet removal ability of rain is higher than that of snow. Rainfall and snowfall both have wet removal abilities for these three air pollutants [26], and the highest removal ability is for S02, followed by that for PM10.

### Dust source

In 2017, with Turfan city as an example of a new area, the PM10 emission level was 1.169 million tons, while that of the PM2.5 emissions was 132,000 tons. Soil dust is the primary source of the particulate emissions in Turpan. Turpan is a sparsely populated area. According to statistics (Turpan Statistical Yearbook (2017)), the area of land other than urban construction and transportation land, water areas and water conservancy facility sites covers 68,900 square kilometers, with a large area of bare soil. In addition, the Turpan Basin is surrounded by Gobis and deserts. Gobis and deserts account for 76.73% of the area, and there is less vegetation and green land. When windy and sand weather conditions occur, large and frequent dust storms are easily formed. Dust is not only a source of atmospheric particulate matter but is also a receptor of particulate matter, resulting in natural dustfall of ground soil dust and sand dust transmission, contributing greatly to the particulate matter concentration in the atmosphere [27].

### Industrial source

With Turpan city as an example, in 2017, the PM10 emissions from industrial sources were 19,200 tons, and the PM2.5 emissions totaled 4,900 tons. The main PM10 contribution sources are the nonmetallic mineral products industry and thermal power production, accounting for 81.4% and 15.2%, respectively, of the PM10 pollution. The PM2.5 contribution of the nonmetallic mineral products industry is

85.9%, and the contributions of thermal power production and petroleum processing are comparable [27].

### Source of life

With Turfan city as an example, in 2017, the PM10 emissions from human activities was 94.58 million tons, and the PM2.5 emission amount was 73.18 million tons. The main source of the emissions of atmospheric particulate matter PM10 and PM2.5 in terms of human activities was coal combustion. Civilian coal combustion technology is poor, and there are no scientific and effective emission control measures, while the amount of fine particulate matter PM2.5 emitted from coal combustion is even higher than that emitted from industrial activities. During the heating season, civilian coal combustion and central heating cause a large amount of pollutants to be emitted, and large amounts of primary aerosols, heavy metals, and water-soluble ions are directly emitted as primary particulate matter. At the same time, gaseous precursors such as SO2 and NO2 are released. Adverse weather conditions easily result in severe pollution. The PM2.5 released from food sources is mainly from the cooking process involving edible oils and foods. Under high-temperature conditions, particulate matter is formed by chemical reactions such as thermal oxidation and decomposition. Most food and beverage sources occur in densely populated areas, and pollutants are emitted near the ground, which has an notable impact on the health of the population. According to activity-level survey information, there were a total of

3,443 catering companies in Turpan in 2017. The total PM10 emissions were 301.6 tons, and the total PM2.5 emissions were 241.3 tons. In addition, the exhaust gas from restaurants contains a large number of VOCs, among which oxygenates and olefins have a high photochemical activity, which are precursors to secondary particulate matter and have a notable impact on atmospheric particulate pollution [28].

### Mobile source

At present, vehicle exhaust is one of the main sources of the atmospheric particulate matter in China [29-30], especially fine particulate matter PM2.5. Motor vehicles also emit gaseous pollutants such as NOx and VOCs, which are converted into secondary particulate matter through photochemical reactions. Compared to coarse particles, fine particles have a larger specific surface area, thus capable of absorbing and carrying more toxic substances, which can be harmful to human health [29-32]. The road sources in Turpan in 2017 emitted 249.19 tons of PM10 and 224.5 tons of PM2.5 [33].

### Nonroad mobile source

The nonroad mobile sources in Turpan are mainly divided into three categories: engineering machinery, agricultural machinery and civilian aviation aircraft. Compared to road mobile sources, nonroad mobile sources usually use diesel and heavy oil as the main fuel. They have the characteristics of a high fuel consumption, long service life, low technical level and low maintenance rate. Most of the pollutants emitted are NOx and particulate matter [34-35].

# Conclusion

The PM2.5, PM10, AQI, CO, NO2, and SO2 levels in China exhibit a stable spatial distribution and persistence over time. PM2.5, PM10, AQI, CO, NO2, and

SO2 are mostly concentrated in Beijing-Tianjin-Hebei and its surroundings and in

Xinjiang from 2014 to 2019. The average concentration monotonically decreases (except CO).

A high NO2 level also occurs in areas where the economy is well developed and the vehicle population is large, including in the Yangtze River Delta, such as Shanghai, in the Pearl River Delta, such as Guangzhou, and in the Sichuan Basin, such as Chongqing.

The O3 level has consistently been high in Inner Mongolia, Qinghai, Gansu and

Shandong from 2014 to 2019.

There is often a significant correlation between the mass concentrations of the different types of particulate matter. The R coefficient of the correlation between PM2.5 and PM10 is 0.88. There is a particularly significant linear positive correlation between these particulates, which indicates that they experience similar changes in the ambient air pollution and practically follow the same migration and transformation laws.

There is a significant positive linear correlation between the atmospheric particulate matter, AQI and pollution gases NO2 and SO2. The correlation coefficient between the atmospheric particulate matter (PM2.5 and PM10), AQI and SO2 is 0.97,

0.81, and 0.88, respectively, and the correlation coefficient between the atmospheric particulate matter (PM2.5 and PM10), AQI and NO2 is 0.82, 0.62, and 0.72, respectively, which is due to the mutual conversion caused by the chemical reaction between the particulate matter and gaseous pollutants.

There exist certain positive and negative correlations between the gaseous pollutants. There is a particularly significant linear positive correlation between N02 and SO2 (the correlation coefficient is 0.90), indicating that these two pollutants follow the same evolution law, and their influencing factors may be similar. N02 and SO2 are initially influenced by factors such as coal combustion and motor vehicle emissions, and there is no correlation between O3 and the other three gaseous pollutants.

The pollution sources in Beijing-Tianjin-Hebei area mainly include decomposition, coal burning, industrial production, and dust. The industrial structure has been adjusted in favor of the tertiary industry, and high-pollution, high-energy consumption secondary industries have been reduced but still account for a large proportion. Regarding the pollution and landforms in Xinjiang, there is a close relationship between static weather conditions, sandstorms, inversion layer, precipitation, dust and industrial sources, human activities, and mobile and nonroad mobile sources.

**Acknowledgments**

I would like to express my gratitude to all those who helped me during the writing of this thesis. My deepest gratitude goes and foremost to Professor Umarova Aminat Batalbievna, my supervisor, for her constant encouragement and guidance.

# References

1. China National Environmental Monitoring Centre.http://www.cnemc.cn/.
2. China Hangzhou Zhenqi Environmental Protection.[https://www.zq12369.com/.](https://www.zq12369.com/)
3. U.S. Geological Survey.https://www.usgs.gov/.
4. Liu Jie,Yang,Peng,Lv,Wensheng, Pollution characteristics of particulate and interpolation analysis of its spatial distribution in the Beijing [J]. Journal of University of Scienceand Technology Beijing,2014, 36(9):1269-1279.
5. Liu Jie,Yang,Peng,Lv,Wensheng,Liu Agudamu,Liu Junxiu,Study on the variation of mass concentration of atmospheric particles in the 6th district of Beijing City Journal of Safety and Environmen,2015,15(6):333-339.
6. Pandis S N; Harley R A, Cass G R, et al. Secondary organic aerosol formation and transport[J]. Atmospheric Environment Part A, 1992, 26(13):2269-2282.
7. West J J, Ansari A S, Pandis S N. Marginal PMz.s:nonliner aerosol mass response to sulfate reductions in the eastern United States[J]. Journal of the Air&Waste Manage Association, 1999, 49(12):1415-1424.
8. Chan Y C, Simpson R W, Mctainsh G H, et al. Characterization of chemical species in PM2.5 and PM10 aerosols in Brisbane, Australia[J]. Atmospheric Environment, 1997, 31(22):3773-3785.
9. Yu S, Xiaoyan T, Chen F, et al. Source apportionment on fine particles in

Beijing[J]. Chinese Journal of Environmental Science, 2002, 23(6):11-16.

10. Liu Jie, Zhang Xiaoling, Xu Xiaofeng, et al. Comparative analysis of suburban

SO2, NOX, O3 and PM2.5 changes in Beijing [J]. Environmental Science, 2008, 29(4):1059-1065.

11. Communique of the State of Beijing Environment in 2018.
12. Liu Jie,Yang,Peng,Lv,Wensheng,LIU Agudamu,LIU Junxiu,Study on the variation of mass concentration of atmospheric particles in the 6th district of Beijing City Journal of Safety and Environmen,2015,15(6):333-339.
13. Wang Yanhuan. Analysis of the external causes of urban smog and its impact on the public[D]. Hefei: University of Science and Technology of China，2015.
14. Mao Xianqiang, Xing Youkai, Hu Tao, et al. Analysis of environmental and economic pathways for coordinated emission reduction of sulfur, nitrogen and carbon in China's power industry[j].Chinese Environmental Science,2012, (4) :

748-756.

15. Communique of the State of Beijing Environment in 2014.
16. Communique of the State of Tianjin Environment in 2014.
17. Communique of the State of Hebei Environment in 2014.
18. Mao Hengqing.Effects of meteorological conditions on the diffusion of atmospheric pollutants [J] .Shandong Meteorology, 1991, 4:2.
19. Li Yan, Wang Youqiang, Liu Qiang.Effects of weather and climate conditions on the distribution of atmospheric pollutants [J]. Anhui Agricultural Sciences, 2009.

37(4): 1781-1782.

20. Law of the People's Republic of China on Air Pollution Control, 2000.
21. Shi Haoling. Analysis of Air Pollution Status and Major Pollutants Sources in

Urumqi (M). Xinjiang Agricultural University. 2012.

22. Tao Jianhong.Climatic Characteristics and Impact of Sand and Dust Weather in

Northwest China [D] .Nanjing University of Information Technology,2007.

23. Zhang Xiaoqi.Re-review on the source area of loess material and its transportation method in the Loess Plateau of China [J] .Quaternary Research,2007, 27(2): 181-186.
24. Wang Cunzhong, Niu Shengjie, Wang Lanning.Characteristics of sandstorm changes in China in the past 50 years [J] .China Desert,2010, 30(4):933-939.
25. 2000-2010 Statistical Yearbook of Chinese Cities, Statistical Yearbook of Autonomous Regions, Statistical Yearbook of Urumqi City, Statistical Yearbook of Urumqi County, Yearbook of Water Resources of Autonomous Regions [R].
26. Liu Qiaojing.Pollution characteristics and source analysis of atmospheric particulate matter in Turpan city (M). Zhejiang University. 2018.
27. Zhao X, Hu Q, Wang X, et al. Composition profiles of organic aerosols from

Chinese residential cooking: case study in urban Guangzhou, south China [J].Journal of Atmospheric Chemistry. 2015, 72(1): 1-18.

28. Kong Shaofei, Bai Zhipeng, Research Progress of Motor Vehicle Exhaust

Composition Spectrum in Source Analysis of Atmospheric Particulate Matter [J] .Environmental Science and Technology. 2013(10): 26-33.

29. Yao Z, Shen X, Ye Y, et al. On-road emission characteristics of VOCs from diesel trucks in Beijing, China[J]. Atmospheric Environment. 2015, 103: 87-93.
30. Sun Guojin.Characteristics of PM2.5 and NOx Emissions from Motor Vehicles and Countermeasures for Emission Reduction [D] .Zhejiang University, 2013.
31. Louie P K K, Ho J W K, Tsang R C W, et al. VOCs and OVOCs distribution and control policy implications in Pearl Environment. 2013, 76: 125-135.Delta region, China [J]. Atmospheric Environment. 2013, 76: 125-135.
32. Shen Xianbao.Research on the characteristics of fine particulates in smoke plume of diesel vehicles [D]. Tsinghua University, 2015.
33. Tan Hua, Liu Juan, Shen Yin, et al. Research on air pollutants emission inventory of dock port farming machinery [J] .Environmental Science and Management.

2013 (06): 82-87.

34. Lu Jun, Huang Cheng, Hu Yuyao, et al. Inventory of atmospheric pollutants from non-road mobile machinery in typical cities in the Yangtze River Delta region [J]. Environmental Science.2017, 38(7): 2738-2746.
35. Li Zhan, Yang Huigai, Jiang Yan, Yin Yuanchang, Wang Hongqian, Wang Bo, Wang Bin,The Study on Spatial Distribution of Atmospheric Contaminant

Concentrations and Optimization of Air Quality Monitoring Sites in Beijing City[j]. Environmental Monitoring in China2015, 31(1):74-78.

36. Luo Xiongbiao. Sources and Control of Ozone Pollutants [J]. Resource

Conservation and Environmental Protection, 2015 (8): 137-137.

# Supporting information

Study on the Spatiotemporal Characteristics of the Air Pollutants in China from 2014

to 2019

Guo Peng ^1¶*^ Umarova Aminat Batalbievna ^1＆^ Luau Yunqi ^1＆^

^1^ Department of Soil, Division of Soil of Physics, Moscow State University, Moscow,

Russian Federation

F-mail: 956095891@qq.com

File S1. Statistical analysis of the PM2.5, AQI, PM10, CO, NO2, O3, SO2 mass concentration in China from 2014 to 2019 (Table S1-S7). Correlation between the particulate matter and AQI levels from 2014 to 2019 (Table S8). Correlation between the particulate matter and gaseous pollutant concentrations from 2014 to 2019. (Table

S9). Correlation analysis of the gaseous pollutants from 2014 to 2019 (Table S10).

Spatiotemporal changes in the annual average PM2.5, AQI, PM10, CO, NO2, O3,

SO2 concentration in China from 2014 to 2019 (Figure S1-S7 ).

**Table S1: Statistical analysis of the PM2.5 mass concentration in China from**

## 2014 to 2019

| PM2.5 (μg·m-3) | 2014 | 2015 | 2016 | 2017 | 2018 | 2019 |
| --- | --- | --- | --- | --- | --- | --- |
| Minimum value | 15.8 | 10.82 | 10.51 | 9.67 | 7.01 | 6.43 |
| Maximum value | 115.96 | 117.69 | 156.33 | 99.93 | 116.96 | 106.64 |
| Average value | 44.84 | 42.03 | 40.69 | 35.17 | 32.11 | 29.13 |
| Standard deviation | 9.07 | 12.28 | 15.23 | 11.67 | 12.08 | 10.46 |

**Top 10 cities and their regions by the annual PM2.5 concentration**

| 2014 PM2.5 | | 2015 PM2.5 | | 2016 PM2.5 | | 2017 PM2.5 | | 2018 PM2.5 | | 2019 PM2.5 | |
| --- | --- | --- | --- | --- | --- | --- | --- | --- | --- | --- | --- |
| City | Concentratio n/(μg·m^-3^) | City | Concentratio n/(μg·m^-3^) | City | Concentratio n/(μg·m^-3^) | City | Concentratio n/(μg·m^-3^) | City | Concentration  /(μg·m^-3^) | City | Concentration  /(μg·m^-3^) |
| Baoding (5) | 117 | Kashi area (5) | 118 | Hetian area (5) | 157 | Kashi area (5) | 100 | Hetian area (5) | 117 | Hetian area (5) | 108 |
| Xingtai (6) | 105 | Hetian area (6) | 119 | Kashi area (6) | 158 | Hetian area (5) | 92 | Kashi area (5) | 94 | Laiwu (2) | 106 |
| Handan (4) | 100 | Dezhou (2) | 101 | Shijiazhuang (4) | 94 | Handan (4) | 86 | Akesu area (2) | 74 | Shangqiu (1) | 94 |
| Shijiazhuang (4) | 99 | Xingtai (6) | 101 | Baoding (5) | 92 | Baoding (5) | 84 | Anyang (4) | 69 | Kashi area (5) | 85 |
| Dezhou (2) | 97 | Hengshui (4) | 98 | Akesu area (2) | 91 | Linfen (3) | 82 | Shijiazhuang (4) | 68 | Anyang (4) | 67 |
| Hengshui (4) | 96 | Hetian area (5) | 98 | Hengshui (4) | 87 | Anyang (4) | 82 | Linfen (3) | 67 | Handan (4) | 63 |
| Liaocheng (3) | 94 | Liaocheng (3) | 98 | Xingtai (6) | 87 | Shijiazhuang (4) | 82 | Xingtai (6) | 66 | Xingtai (6) | 62 |
| Laiwu (2) | 90 | Zhengzhou (1) | 95 | Liaocheng (3) | 86 | Xingtai (6) | 81 | Handan (4) | 65 | Luoyang (1) | 60 |
| Tangshan (1) | 90 | Heze (1) | 94 | Jiazuo (1) | 85 | Xianyang (1) | 79 | Baoding (5) | 65 | Linfen (3) | 59 |
| Langfang (1) | 89 | Xinxiang (1) | 93 | Anyang (4) | 85 | Hengshui (4) | 77 | Xuzhou (1) | 62 | Puyang (1) | 59 |

References

1. China National Environmental Monitoring Centre.http://www.cnemc.cn/.
2. China Hangzhou Zhenqi Environmental Protection.[https://www.zq12369.com/.](https://www.zq12369.com/)

**Table S2: Statistical analysis of the AQI in China from 2014 to 2019**

| AQI (μg·m-3) | 2014 | 2015 | 2016 | 2017 | 2018 | 2019 |
| --- | --- | --- | --- | --- | --- | --- |
| Minimum value | 36.06 | 36.4 | 33.62 | 32.36 | 6.84 | 6.9 |
| Maximum value | 161.12 | 200.74 | 204.31 | 187.13 | 216.74 | 183.31 |
| Average value | 77.89 | 76.75 | 73.8 | 71.03 | 68.05 | 54.95 |
| Standard deviation | 12.43 | 21.42 | 23.34 | 19.68 | 25.31 | 20.68 |

**Top 10 cities and their regions by the AQI value for each year**

| 2014 AQI | | 2015 AQI | | 2016 AQI | | 2017 AQI | | 2018 AQI | | 2019 AQI | |
| --- | --- | --- | --- | --- | --- | --- | --- | --- | --- | --- | --- |
| City | AQI | City | AQI | City | AQI | City | AQI | City | AQI | City | AQI |
| Baoding (4) | 162 | Hetian area (5) | 201 | Kashi area (5) | 205 | Hetian area (5) | 187 | Hetian area (5) | 217 | Hetian area (5) | 184 |
| Xingtai (5) | 146 | Kashi area (5) | 198 | Hetian area (5) | 184 | Kashi area (5) | 162 | Kashi area (5) | 180 | Kashi area (5) | 152 |
| Hengshui (3) | 143 | Akesu area (5) | 148 | Akesu area (5) | 155 | Akesu area (5) | 135 | Kezhou area (5) | 155 | Laiwu (1) | 142 |
| Handan (4) | 140 | Baoding (4) | 144 | Kezhou area (5) | 147 | Handan (4) | 126 | Akesu area (5) | 153 | Shangqiu (1) | 134 |
| Shijiazhuang (4) | 138 | Hengshui (3) | 141 | Kuerle (3) | 133 | Anyang (3) | 123 | Tulufan area (4) | 122 | Akesu area (5) | 120 |
| Dezhou (3) | 131 | Dezhou (3) | 139 | Shijiazhuang (3) | 131 | Xingtai (5) | 122 | Kuerle (3) | 121 | Kezhou area (5) | 118 |
| Liaozheng (2) | 130 | Xingtai (5) | 138 | Baoding(4) | 128 | Baoding (4) | 122 | Anyang (3) | 115 | Kuerle (3) | 116 |
| Langfang (1) | 127 | Kezhou area (5) | 137 | Tulufan area (4) | 127 | Shijiazhuang (3) | 122 | Xingtai (5) | 114 | Tulufan area (4) | 112 |
| Tangshan (1) | 126 | Zhengzhou (1) | 135 | Hengshui (3) | 126 | Xianyang (2) | 121 | Handan (4) | 114 | Anyang (3) | 106 |
| Heze (1) | 125 | Liaozheng (2) | 134 | Dezhou (3) | 123 | Kezhou area (5) | 120 | Wujiaqu (1) | 113 | Handan (4) | 102 |

References

1. China National Environmental Monitoring Centre.http://www.cnemc.cn/.
2. China Hangzhou Zhenqi Environmental Protection.[https://www.zq12369.com/.](https://www.zq12369.com/)

**Table S3: Statistical analysis of the PM10 mass concentration in China from 2014 to 2019**

| PM10 (μg·m-3) | 2014 | 2015 | 2016 | 2017 | 2018 | 2019 |
| --- | --- | --- | --- | --- | --- | --- |
| Minimum value | 32.25 | 26.07 | 23.96 | 21.77 | 6.78 | 6.95 |
| Maximum value | 196 | 340.08 | 433.56 | 317.27 | 432.23 | 333.97 |
| Average value | 88.51 | 88.2 | 87.53 | 77.96 | 82.09 | 66.31 |
| Standard deviation | 19.15 | 37.43 | 46.1 | 34.36 | 52.43 | 36.34 |

**Top 10 cities and their regions by the PM10 concentration annually**

| 2014 PM10 | | 2015 PM10 | | 2016 PM10 | | 2017 PM10 | | 2018 PM10 | | 2019 PM10 | |
| --- | --- | --- | --- | --- | --- | --- | --- | --- | --- | --- | --- |
| City | Concentrati on/(μg·m^-3^) | City | Concentrati on/(μg·m^-3^) | City | Concentrati on/(μg·m^-3^) | City | Concentrati on/(μg·m^-3^) | City | Concentrati on/(μg·m^-3^) | City | Concentrati on/(μg·m^-3^) |
| Baoding (2) | 198 | Hetian area (5) | 341 | Kasha area (5) | 435 | Hetian area (5) | 318 | Hetian area (5) | 432 | Hetian area (5) | 335 |
| Xingtai (2) | 187 | Kasha area (5) | 308 | Hetian area (5) | 283 | Kasha area (5) | 248 | Kasha area (5) | 331 | Kasha area (5) | 229 |
| Hengshui (2) | 176 | Akesu area (5) | 217 | Akesu area (5) | 246 | Akesu area (5) | 198 | Kezhou (5) | 291 | Kezhou (5) | 182 |
| Handan (6) | 174 | Kezhou(5) | 186 | Kezhou (5) | 217 | Tulufan area (4) | 158 | Akesu area (5) | 256 | Akesu area (5) | 181 |
| Shijiazhuang (3) | 172 | Hengshui (2) | 176 | Kuerle (5) | 210 | Handan (6) | 157 | Tulufan area (4) | 180 | Kuerle (5) | 171 |
| Liaocheng(2) | 165 | Baoding (2) | 175 | Tulufan area (4) | 170 | Kezhou (5) | 154 | Kuerle (5) | 171 | Laiwu (1) | 168 |
| Linyi (1) | 163 | Xingtai (1) | 172 | Shijiazhuang (3) | 161 | Shijiazhuang (3) | 153 | Wujiaqu (1) | 136 | Tulufan area (4) | 164 |
| Zibo (1) | 161 | Handan (6) | 167 | Dezhou(2) | 152 | Xingtai (2) | 152 | Handan (6) | 136 | Handan (6) | 124 |
| Jinan (1) | 158 | Zhengzhou (1) | 166 | Handan (6) | 151 | Pingdu (1) | 150 | Xingtai (2) | 136 | Shangqiu (1) | 124 |
| Kuerle (5) | 152 | Dezhou (2) | 165 | Liaocheng (2) | 150 | Kuerle (5) | 142 | Weinan (1) | 135 | Zaozhuang (1) | 117 |

References

1. China National Environmental Monitoring Centre.http://www.cnemc.cn/.
2. China Hangzhou Zhenqi Environmental Protection.[https://www.zq12369.com/.](https://www.zq12369.com/)

**Table S4: Statistical analysis of the CO mass concentration in China from 2014 to 2019**

| CO (mg·m-3) | 2014 | 2015 | 2016 | 2017 | 2018 | 2019 |
| --- | --- | --- | --- | --- | --- | --- |
| Minimum value | 0.42 | 0.41 | 0.44 | 0.36 | 0.33 | 0.24 |
| Maximum value | 2.18 | 2.5 | 2.45 | 2.04 | 7.04 | 7.01 |
| Average value | 1.05 | 0.99 | 0.94 | 0.88 | 1.19 | 1.09 |
| Standard deviation | 0.16 | 0.19 | 0.19 | 0.18 | 0.97 | 0.93 |

**Top 10 cities and their regions by the CO concentration each year**

| 2014 CO | | 2015 CO | | 2016 CO | | 2017 CO | | 2018 CO | | 2019 CO | |
| --- | --- | --- | --- | --- | --- | --- | --- | --- | --- | --- | --- |
| City | Concentrati on/(μg·m^-3^) | City | Concentrati on/(μg·m^-3^) | City | Concentrati on/(μg·m^-3^) | City | Concentrati on/(μg·m^-3^) | City | Concentrati on/(μg·m^-3^) | City | Concentrati on/(μg·m^-3^) |
| Penglai (2) | 2.25 | Lvliang (1) | 2.75 | Linfen (6) | 2.52 | Linfen (6) | 2.07 | Linfen (6) | 1.76 | Laiwu (1) | 1.53 |
| Linfen (6) | 2.20 | Linfen (6) | 2.36 | Tangshan (6) | 2.27 | Jincheng (4) | 1.98 | Tangshan (6) | 1.69 | Linfen (6) | 1.46 |
| Binzhou (2) | 2.13 | Tangshan (6) | 2.13 | Anyang (5) | 2.17 | Tangshan (6) | 1.96 | Panzhihua (2) | 1.53 | Tangshan (6) | 1.37 |
| Zibo (4) | 2.11 | Zibo (4) | 2.11 | Yuncheng (3) | 2.06 | Anyang (5) | 1.85 | Anyang (5) | 1.53 | Datong (2) | 1.35 |
| Benxi (1) | 2.09 | Binzhou (2) | 2.10 | Jincheng (4) | 2.00 | Dekehasake (2) | 1.83 | Dekehasake (2) | 1.46 | Changzhi (5) | 1.25 |
| Tangshan (6) | 2.06 | Naqu area (1) | 2.09 | Luoyang (1) | 2.00 | Zibo (4) | 1.76 | Loudi (1) | 1.45 | Ganzhou (2) | 1.24 |
| Changzhi (5) | 2.05 | Anyang (5) | 2.06 | Changzhi (5) | 2.00 | Yuncheng (3) | 1.71 | Datong (2) | 1.42 | Jincheng (4) | 1.23 |
| Liaocheng (1) | 2.00 | Zhoukou (1) | 2.02 | Hebi (2) | 1.96 | Hebi (2) | 1.68 | Ganzhou (2) | 1.42 | Anyang (5) | 1.21 |
| Yanan (1) | 1.93 | Yuncheng (3) | 1.96 | Zibo (4) | 1.90 | Changzhi (5) | 1.67 | Xining (1) | 1.41 | Panzhihua (2) | 1.20 |
| Baoding (1) | 1.93 | Changzhi (5) | 1.96 | Yilihasake (1) | 1.85 | Xingtai | 1.67 | Jincheng(4) | 1.38 | Penglai (2) | 1.19 |

References

1. China National Environmental Monitoring Centre.http://www.cnemc.cn/.
2. China Hangzhou Zhenqi Environmental Protection.[https://www.zq12369.com/.](https://www.zq12369.com/)

**Table S5: Statistical analysis of the NO2 mass concentration in China from 2014 to 2019**

| NO2 (μg·m-3) | 2014 | 2015 | 2016 | 2017 | 2018 | 2019 |
| --- | --- | --- | --- | --- | --- | --- |
| Minimum value | 12.31 | 7.72 | 8.96 | 6.84 | 6.99 | 6.92 |
| Maximum value | 56.49 | 59.19 | 60.37 | 58.08 | 53.85 | 50.35 |
| Average value | 31.2 | 23.51 | 23.97 | 23.95 | 21.29 | 20.17 |
| Standard deviation | 4.3 | 6.09 | 5.8 | 6.45 | 6.21 | 5.68 |

**Top 10 cities and their regions by the NO2 concentration annually**

| 2014 NO2 | | 2015 NO2 | | 2016 NO2 | | 2017 NO2 | | 2018 NO2 | | 2019 NO2 | |
| --- | --- | --- | --- | --- | --- | --- | --- | --- | --- | --- | --- |
| City | Concentrati on/(μg·m^-3^) | City | Concentrati on/(μg·m^-3^) | City | Concentrati on/(μg·m^-3^) | City | Concentrati on/(μg·m^-3^) | City | Concentrati on/(μg·m^-3^) | City | Concentrati on/(μg·m^-3^) |
| Zibo (3) | 65 | Zibo (3) | 61 | Xingtai (6) | 61 | Tangshan (6) | 58 | Huzhou (3) | 54 | Laiwu (2) | 51 |
| Tangshan (6) | 58 | Tangshan (6) | 60 | Tangshan (6) | 58 | Xian (3) | 58 | Tangshan (6) | 54 | Tangshan (6) | 49 |
| Linyi (1) | 57 | Xingtai (6) | 59 | Baoding (3) | 57 | Xingtai (6) | 56 | Xian (3) | 53 | Yunlin (1) | 47 |
| Xingtai (6) | 56 | Zhengzhou (5) | 55 | Handan (2) | 54 | Huzhou (3) | 55 | Changzhou (1) | 49 | Xian (3) | 46 |
| Jinan (3) | 53 | Baoding (3) | 53 | Huzhou (3) | 54 | Xianyang (2) | 53 | Tianjin (2) | 49 | Taiyuan (2) | 44 |
| Baoding (3) | 52 | Linxiazhou (1) | 53 | Shijiazhuang (2) | 54 | Tianjin (2) | 53 | Xingtai (6) | 47 | Lvliang (1) | 44 |
| Beijing (1) | 52 | Suzhou (2) | 51 | Zhengzhou (5) | 53 | Zhengzhou (5) | 52 | Xinxiang (2) | 47 | Huzhou(2) | 44 |
| Suzhou (2) | 51 | Jinan (3) | 51 | Zibo (3) | 52 | Weinan (1) | 51 | Xianyang (2) | 47 | Jinan (3) | 43 |
| Laiwu (2) | 51 | Zhuji (1) | 51 | Hebi (1) | 52 | Handan (2) | 51 | Taiyuan (2) | 46 | Xingtai (6) | 42 |
| Qinhuangdao (2) | 50 | Xinxiang (2) | 51 | Wulumuqi (1) | 52 | Shijiazhuang (2) | 50 | Zhengzhou (5) | 46 | Zhengzhou (5) | 42 |

References

1. China National Environmental Monitoring Centre.http://www.cnemc.cn/.
2. China Hangzhou Zhenqi Environmental Protection.[https://www.zq12369.com/.](https://www.zq12369.com/)

**Table S6: Statistical analysis of the O3 mass concentration in China from 2014 to 2019**

| O3 (μg·m-3) | 2014 | 2015 | 2016 | 2017 | 2018 | 2019 |
| --- | --- | --- | --- | --- | --- | --- |
| Minimum value | 30.83 | 28.42 | 32.47 | 37.92 | 7.76 | 6.93 |
| Maximum value | 82.33 | 92.65 | 95.03 | 103.52 | 103.01 | 103.05 |
| Average value | 59.42 | 59.12 | 59.98 | 67.82 | 62.53 | 61.67 |
| Standard deviation | 4.83 | 6.66 | 6.78 | 8.38 | 10.11 | 11.98 |

**Top 10 cities and regions by the O3 concentration each year**

| 2014 O3 | | 2015 O3 | | 2016 O3 | | 2017 O3 | | 2018 O3 | | 2019 O3 | |
| --- | --- | --- | --- | --- | --- | --- | --- | --- | --- | --- | --- |
| City | Concentrati on/(μg·m^-3^) | City | Concentrati on/(μg·m^-3^) | City | Concentrati on/(μg·m^-3^) | City | Concentrati on/(μg·m^-3^) | City | Concentrati on/(μg·m^-3^) | City | Concentrati on/(μg·m^-3^) |
| Rongcheng(3) | 93 | Haixizhou (3) | 97 | Haibeizhou (4) | 95 | Enshizhou (1) | 37 | Alashanmeng (4) | 105 | Haixizhou (3) | 104 |
| Weihai(4) | 92 | Alashanmeng (4) | 94 | Alashanmeng (4) | 95 | Xiangxi (1) | 38 | Guoluozhou (3) | 90 | Alashanmeng (4) | 102 |
| Penglai(1) | 87 | Hainanzhou (3) | 94 | Hainanzhou (3) | 83 | Qiandongnanzhou (1) | 38 | Kezhou (1) | 89 | Haibeizhou (4) | 96 |
| Linan(1) | 85 | Haibeizhou (4) | 90 | Yixing (1) | 82 | Xishuangbanna (1) | 40 | Weihai (4) | 89 | Weihai (4) | 92 |
| Jiaonan(2) | 82 | Yanzheng (1) | 82 | Guoluozhou (3) | 81 | Qianxinanzhou (1) | 40 | Haixizhou (3) | 88 | Hainanzhou (3) | 89 |
| Weifang(1) | 81 | Jiayuguan (2) | 81 | Yingkou (2) | 81 | Nujiang (1) | 41 | Jiaozuo (2) | 87 | Jiaozuo (2) | 89 |
| Wendeng(1) | 81 | Guoluozhou (3) | 81 | Jiayuguan (2) | 81 | Chongqing (1) | 42 | Haibeizhou (4) | 87 | Rongcheng (3) | 89 |
| Dongying(1) | 80 | Yingkou (2) | 80 | Eerduosi (2) | 80 | Wuzhou (1) | 42 | Rongcheng (3) | 85 | Jinchang (1) | 86 |
| Eerduosi(2) | 80 | Lasa (1) | 80 | Jiaonan (1) | 80 | Hechi (1) | 43 | Qingyang (1) | 85 | Changzhi (2) | 85 |
| Qingdao(1) | 80 | Weihai (4) | 79 | Dalian (1) | 79 | Pingxiang (1) | 43 | Changzhi (2) | 84 | Jincheng (1) | 84 |

References

1. China National Environmental Monitoring Centre.http://www.cnemc.cn/.
2. China Hangzhou Zhenqi Environmental Protection.[https://www.zq12369.com/.](https://www.zq12369.com/)

**Table S7: Statistical analysis of the SO2 mass concentration in China from 2014 to 2019**

| SO2 (μg·m-3) | 2014 | 2015 | 2016 | 2017 | 2018 | 2019 |
| --- | --- | --- | --- | --- | --- | --- |
| Minimum value | 2.23 | 2.81 | 17.01 | 2.37 | 3.19 | 3.36 |
| Maximum value | 83.3 | 75.61 | 87.08 | 82.42 | 47.56 | 36.92 |
| Average value | 24.43 | 20.02 | 17.01 | 14.67 | 11.59 | 9.47 |
| Standard deviation | 8.46 | 8.47 | 7.55 | 6.21 | 4.05 | 3.18 |

**Top 10 cities and their regions by the SO2 concentration on a yearly basis**

| 2014 SO2 | | 2015 SO2 | | 2016 SO2 | | 2017 SO2 | | 2018 SO2 | | 2019 SO2 | |
| --- | --- | --- | --- | --- | --- | --- | --- | --- | --- | --- | --- |
| City | Concentrati on/(μg·m^-3^) | City | Concentrati on/(μg·m^-3^) | City | Concentrati on/(μg·m^-3^) | City | Concentrati on/(μg·m^-3^) | City | Concentrati on/(μg·m^-3^) | City | Concentrati on/(μg·m^-3^) |
| Zibo (2) | 108 | Zibo (2) | 82 | Jinzhong (4) | 87 | Jinzhong (4) | 83 | Linfen (6) | 48 | Baiyin (2) | 39 |
| Yangquan (3) | 85 | Shuozhou (3) | 76 | Linfen (6) | 82 | Linfen (6) | 79 | Baiyin (2) | 42 | Wuhai (4) | 33 |
| Laiwu (2) | 81 | Hetiandiqu (1) | 72 | Shuozhou (3) | 67 | Lvliang (5) | 68 | Lvliang (5) | 39 | Laiwu (2) | 32 |
| Taiyuan (4) | 66 | Jinzhong (4) | 72 | Shijiazui (5) | 67 | Shijiazui (5) | 51 | Panzhihua (2) | 38 | Shuozhou (3) | 29 |
| Jining (1) | 64 | Shijiazui (5) | 71 | Jincheng (1) | 66 | Taiyuan (4) | 51 | Jinzhou(2) | 37 | Panzhihua (2) | 29 |
| Dongying (1) | 64 | Taiyuan (4) | 68 | Yuncheng (2) | 66 | Yinchuang (2) | 51 | Shijiazui (5) | 37 | Xinzhou (2) | 29 |
| Zaozhuang (1) | 63 | Lvliang (5) | 68 | Taiyuan(4) | 65 | Yuncheng (2) | 51 | Jinzhong (4) | 36 | Lvliang (5) | 29 |
| Linfen (6) | 63 | Zhangqiu (1) | 64 | Lvliang (5) | 62 | Wuhai (4) | 51 | Yinchuang (2) | 36 | Datong (1) | 28 |
| Shijiazui (5) | 59 | Linfen (6) | 63 | Yangquan (3) | 61 | Yangquan (3) | 49 | Huludao (1) | 36 | Linfen (6) | 27 |
| Shenyang (1) | 57 | Wuhai (4) | 62 | Changzhi (1) | 61 | Xinzhou (2) | 48 | Wuhai (4) | 34 | Jinzhou (2) | 27 |

References

1. China National Environmental Monitoring Centre.http://www.cnemc.cn/.
2. China Hangzhou Zhenqi Environmental Protection.[https://www.zq12369.com/.](https://www.zq12369.com/)

**Table S8: Correlation between the particulate matter and AQI levels from 2014 to 2019**

| Between particles | Fitting equation | Correlation coefficient R | Goodness of fit  R2 | Degrees of freedom n-2 | Significance level | | Mass concentration ratio |
| --- | --- | --- | --- | --- | --- | --- | --- |
|  |  |  |  |  | R_0_.05 | R_0_.01 |  |
| x: PM2.5;y: AQI | y = 1.24x + 23.96 | 0.91 | 0.83 | 50638 | 0.062 | 0.081 | - |
| x: PM2.5;y: PM10 | y = 1.24x + 35.63 | 0.88 | 0.77 | 50638 | 0.062 | 0.081 | 0.46 |
| x: AQI;y: PM10 | y = 0.98x + 12.55 | 0.95 | 0.91 | 50638 | 0.062 | 0.081 | - |

References

1. China National Environmental Monitoring Centre.http://www.cnemc.cn/.
2. China Hangzhou Zhenqi Environmental Protection.[https://www.zq12369.com/.](https://www.zq12369.com/)
4. Liu Jie,Yang,Peng,Lv,Wensheng, Pollution characteristics of particulate and interpolation analysis of its spatial distribution in the Beijing [J]. Journal of University of Scienceand Technology Beijing,2014, 36(9):1269-1279.
5. Liu Jie,Yang,Peng,Lv,Wensheng,Liu Agudamu,Liu Junxiu,Study on the variation of mass concentration of atmospheric particles in the 6th district of Beijing City Journal of Safety and Environmen,2015,15(6):333-339.

**Table S9: Correlation between the particulate matter and gaseous pollutant concentrations from 2014 to 2019**

| Particulate matter and gaseous pollutants | Fitting equation | Correlation coefficient R | Goodness of fit  R2 | Degrees of freedom n-2 | Significance level | |
| --- | --- | --- | --- | --- | --- | --- |
|  |  |  |  |  | R_0_.05 | R_0_.01 |
| x: CO;y: PM2.5 | y = -21.83x + 59.67 | -0.39 | 0.16 | 50638 | 0.062 | 0.081 |
| x: CO;y: AQI | y = -29.37x + 100.47 | -0.39 | 0.15 | 50638 | 0.062 | 0.081 |
| x: CO;y: PM10 | y = -15.37x + 97.50 | -0.20 | 0.04 | 50638 | 0.062 | 0.081 |
| x: NO2;y: PM2.5 | y = 1.31x + 5.78 | 0.82 | 0.68 | 50638 | 0.062 | 0.081 |
| x: NO2;y: AQI | y = 1.57x + 32.74 | 0.72 | 0.52 | 50638 | 0.062 | 0.081 |
| x: NO2;y: PM10 | y = 1.40x + 48.14 | 0.62 | 0.39 | 50638 | 0.062 | 0.081 |
| x: O3;y: PM2.5 | y = -0.99x + 98.38 | -0.52 | 0.27 | 50638 | 0.062 | 0.081 |
| x: O3;y: AQI | y = -0.70x + 113.51 | -0.27 | 0.07 | 50638 | 0.062 | 0.081 |
| x: O3;y: PM10 | y = -1.22x + 156.95 | -0.46 | 0.21 | 50638 | 0.062 | 0.081 |
| x: SO2;y: PM2.5 | y = 1.09x + 19.73 | 0.97 | 0.95 | 50638 | 0.062 | 0.081 |
| x: SO2;y: AQI | y = 1.33x + 48.81 | 0.88 | 0.77 | 50638 | 0.062 | 0.081 |
| x: SO2;y: PM10 | y = 1.27x + 61.17 | 0.81 | 0.66 | 50638 | 0.062 | 0.081 |

References

1. China National Environmental Monitoring Centre.http://www.cnemc.cn/.
2. China Hangzhou Zhenqi Environmental Protection.[https://www.zq12369.com/.](https://www.zq12369.com/)
6. Pandis S N; Harley R A, Cass G R, et al. Secondary organic aerosol formation and transport[J]. Atmospheric Environment Part A, 1992, 26(13):2269-2282.
7. West J J, Ansari A S, Pandis S N. Marginal PMz.s:nonliner aerosol mass response to sulfate reductions in the eastern United States[J]. Journal of the Air&Waste Manage Association, 1999, 49(12):1415-1424.
8. Chan Y C, Simpson R W, Mctainsh G H, et al. Characterization of chemical species in PM2.5 and PM10 aerosols in Brisbane, Australia[J]. Atmospheric Environment, 1997, 31(22):3773-3785.
9. Yu S, Xiaoyan T, Chen F, et al. Source apportionment on fine particles in Beijing[J]. Chinese Journal of Environmental Science, 2002, 23(6):11-16.
10. Liu Jie, Zhang Xiaoling, Xu Xiaofeng, et al. Comparative analysis of suburban SO2, NOX, O3 and PM2.5 changes in Beijing [J]. Environmental Science, 2008, 29(4):1059-1065.

**Table S10: Correlation analysis of the gaseous pollutants from 2014 to 2019**

| Between gaseous pollutants | Fitting equation | Correlation coefficient R | Goodness of fit  R2 | Degrees of freedom n-2 | Significance level | |
| --- | --- | --- | --- | --- | --- | --- |
|  |  |  |  |  | R_0_.05 | R_0_.01 |
| x: CO;y: NO2 | y = -7.95x + 32.15 | -0.23 | 0.05 | 50638 | 0.062 | 0.081 |
| x: CO;y: O3 | y = -9.30x + 71.28 | -0.32 | 0.10 | 50638 | 0.062 | 0.081 |
| x: CO;y: SO2 | y = -15.81x + 32.38 | -0.32 | 0.10 | 50638 | 0.062 | 0.081 |
| x: NO2;y: O3 | y = -0.24x + 67.44 | -0.28 | 0.08 | 50638 | 0.062 | 0.081 |
| x:NO2;y: SO2 | y = 1.29x - 14.82 | 0.90 | 0.81 | 50638 | 0.062 | 0.081 |
| x: O3;y: SO2 | y = -0.81x + 66.36 | -0.48 | 0.23 | 50638 | 0.062 | 0.081 |

References

1. China National Environmental Monitoring Centre.http://www.cnemc.cn/.
2. China Hangzhou Zhenqi Environmental Protection.[https://www.zq12369.com/.](https://www.zq12369.com/)
11. Communique of the State of Beijing Environment in 2018.
12. Liu Jie,Yang,Peng,Lv,Wensheng,LIU Agudamu,LIU Junxiu,Study on the

variation of mass concentration of atmospheric particles in the 6th district of Beijing City Journal of Safety and Environmen,2015,15(6):333-339.

**Figure S1: Spatiotemporal changes in the annual average PM2.5 concentration in China from 2014 to 2019**

From 2014 to 2019, a high PM2.5 mass concentration generally occurred in Beijing-Tianjin-Hebei and its surroundings in the eastern part of northern China and in Xinjiang in the western part of northern China.

**Figure S1. Spatiotemporal changes in the annual average PM2.5 concentration in**

## China from 2014 to 2019

**Figure S2: Spatiotemporal changes in the annual average AQI value in China from 2014 to 2019**

From 2014 to 2019, a high AQI also generally occurred in the Beijing-Tianjin-Hebei region and its surroundings in the eastern part of northern China and in Xinjiang in the western part of northern China.

**Figure S2. Spatiotemporal changes in the annual average AQI value in China**

## from 2014 to 2019

**Figure S3: Spatiotemporal changes in the annual average PM10 concentration in**

**China from 2014 to 2019**

From 2014 to 2019, a high PM10 mass concentration generally occurred in the

Beijing-Tianjin-Hebei region and its surrounding areas in the eastern part of northern

China and Xinjiang in the western part of northern China.

**Figure S3. Spatiotemporal changes in the annual average PM10 concentration in**

## China from 2014 to 2019

**Figure S4: Spatiotemporal changes in the annual average CO concentration in**

**China from 2014 to 2019**

From 2014 to 2019, a higher CO mass concentration was generally observed in the

Beijing-Tianjin-Hebei region and its surrounding areas in the eastern part of northern China and Xinjiang in the western part of northern China (it is worth noting that a high CO mass concentration was observed in Tibet in 2018 and 2019).

**Figure S4. Spatiotemporal changes in the annual average CO concentration in China from 2014 to 2019**

**Figure S5: Spatiotemporal changes in the annual average NO2 concentration in**

**China from 2014 to 2019**

From 2014 to 2019, a higher NO2 mass concentration generally occurred in the

Beijing-Tianjin-Hebei region and its surroundings in the eastern part of northern China and in Xinjiang in the western part of northern China.

**Figure S5. Spatiotemporal changes in the annual average NO2 concentration in**

## China from 2014 to 2019

**Figure S6: Spatiotemporal changes in the annual average O3 concentration in**

**China from 2014 to 2019**

A higher O3 mass concentration has stably occurred in Inner Mongolia, Qinghai, and Gansu in northern China from 2014 to 2019.

**Figure S6. Spatiotemporal changes in the annual average O3 concentration in**

## China from 2014 to 2019

**Figure S7: Spatiotemporal changes in the annual average SO2 concentration in**

**China from 2014 to 2019**

A	higher	SO2	mass	concentration	was	generally	observed	in	the

Beijing-Tianjin-Hebei region and its surrounding areas in the eastern part of northern

China and in Xinjiang in the northwest of mainland China.

**Figure S7. Spatiotemporal changes in the annual average SO2 concentration in**

## China from 2014 to 2019

References

1. China National Environmental Monitoring Centre.http://www.cnemc.cn/.
2. China Hangzhou Zhenqi Environmental Protection.[https://www.zq12369.com/.](https://www.zq12369.com/)

Figure WORD	[Click here to access/download;Figure;Figure.docx](https://www.editorialmanager.com/pone/download.aspx?id=26278037&guid=5b5af045-ef55-41bc-994d-5ed4f3291c24&scheme=1)

**Figure 0: Administrative planning map of China**

## Figure 0: Administrative planning map of China

**Figure 1: Spatiotemporal changes in the annual average PM2.5 concentration in China from 2014 to 2019**

From 2014 to 2019, a high PM2.5 mass concentration generally occurred in Beijing-Tianjin-Hebei and its surroundings in the eastern part of northern China and in Xinjiang in the western part of northern China.

**Figure 1. Spatiotemporal changes in the annual average PM2.5 concentration in**

## China from 2014 to 2019

**Figure 2: Spatiotemporal changes in the annual average AQI value in China from 2014 to 2019**

From 2014 to 2019, a high AQI also generally occurred in the Beijing-Tianjin-Hebei region and its surroundings in the eastern part of northern China and in Xinjiang in the western part of northern China.

**Figure 2. Spatiotemporal changes in the annual average AQI value in China**

## from 2014 to 2019

**Figure 3: Spatiotemporal changes in the annual average PM10 concentration in**

**China from 2014 to 2019**

From 2014 to 2019, a high PM10 mass concentration generally occurred in the Beijing-Tianjin-Hebei region and its surrounding areas in the eastern part of northern China and Xinjiang in the western part of northern China.

**Figure 3. Spatiotemporal changes in the annual average PM10 concentration in**

## China from 2014 to 2019

**Figure 4: Spatiotemporal changes in the annual average CO concentration in**

**China from 2014 to 2019**

From 2014 to 2019, a higher CO mass concentration was generally observed in the

Beijing-Tianjin-Hebei region and its surrounding areas in the eastern part of northern China and Xinjiang in the western part of northern China (it is worth noting that a high CO mass concentration was observed in Tibet in 2018 and 2019).

**Figure 4. Spatiotemporal changes in the annual average CO concentration in**

## China from 2014 to 2019

**Figure 5: Spatiotemporal changes in the annual average NO2 concentration in**

**China from 2014 to 2019**

From 2014 to 2019, a higher NO2 mass concentration generally occurred in the

Beijing-Tianjin-Hebei region and its surroundings in the eastern part of northern

China and in Xinjiang in the western part of northern China.

**Figure 5. Spatiotemporal changes in the annual average NO2 concentration in**

## China from 2014 to 2019

**Figure 6: Spatiotemporal changes in the annual average O3 concentration in**

**China from 2014 to 2019**

A higher O3 mass concentration has stably occurred in Inner Mongolia, Qinghai, and Gansu in northern China from 2014 to 2019.

**Figure 6. Spatiotemporal changes in the annual average O3 concentration in**

## China from 2014 to 2019

**Figure 7: Spatiotemporal changes in the annual average SO2 concentration in**

**China from 2014 to 2019**

A	higher	SO2	mass	concentration	was	generally	observed	in	the

Beijing-Tianjin-Hebei region and its surrounding areas in the eastern part of northern China and in Xinjiang in the northwest of mainland China.

## Figure 7. Spatiotemporal changes in the annual average SO2 concentration in China from 2014 to 2019

[Click here to access/download](https://www.editorialmanager.com/pone/download.aspx?id=26278451&guid=a34b3e46-13bc-4791-a963-2ad7ebb10000&scheme=1)

[Supporting Information - Compressed/ZIP File Archive Supporting Information PM2.5.rar](https://www.editorialmanager.com/pone/download.aspx?id=26278451&guid=a34b3e46-13bc-4791-a963-2ad7ebb10000&scheme=1)

[Click here to access/download](https://www.editorialmanager.com/pone/download.aspx?id=26278452&guid=caf2274a-01b9-4b0c-ae4c-88f71a4bddc3&scheme=1)

[Supporting Information - Compressed/ZIP File Archive Supporting Information AQI.zip](https://www.editorialmanager.com/pone/download.aspx?id=26278452&guid=caf2274a-01b9-4b0c-ae4c-88f71a4bddc3&scheme=1)

[Click here to access/download](https://www.editorialmanager.com/pone/download.aspx?id=26278453&guid=b87873cd-04a2-4fa4-810e-c61529288afb&scheme=1)

[Supporting Information - Compressed/ZIP File Archive Supporting Information PM10.zip](https://www.editorialmanager.com/pone/download.aspx?id=26278453&guid=b87873cd-04a2-4fa4-810e-c61529288afb&scheme=1)

[Click here to access/download](https://www.editorialmanager.com/pone/download.aspx?id=26278454&guid=f1f31f7f-0fc0-4618-9cdb-74436a08c74b&scheme=1)

[Supporting Information - Compressed/ZIP File Archive Supporting Information CO.zip](https://www.editorialmanager.com/pone/download.aspx?id=26278454&guid=f1f31f7f-0fc0-4618-9cdb-74436a08c74b&scheme=1)

[Click here to access/download](https://www.editorialmanager.com/pone/download.aspx?id=26278455&guid=dd36af52-7d46-4d73-abb6-e5e33bdb66e5&scheme=1)

[Supporting Information - Compressed/ZIP File Archive Supporting Information NO2.zip](https://www.editorialmanager.com/pone/download.aspx?id=26278455&guid=dd36af52-7d46-4d73-abb6-e5e33bdb66e5&scheme=1)

[Click here to access/download](https://www.editorialmanager.com/pone/download.aspx?id=26278456&guid=c216320b-2df7-4eab-88a7-64d8762425d0&scheme=1)

[Supporting Information - Compressed/ZIP File Archive Supporting Information O3.zip](https://www.editorialmanager.com/pone/download.aspx?id=26278456&guid=c216320b-2df7-4eab-88a7-64d8762425d0&scheme=1)

[Click here to access/download](https://www.editorialmanager.com/pone/download.aspx?id=26278457&guid=a1178922-260b-42b8-910f-73f40edcbaf7&scheme=1)

[Supporting Information - Compressed/ZIP File Archive Supporting Information SO2.zip](https://www.editorialmanager.com/pone/download.aspx?id=26278457&guid=a1178922-260b-42b8-910f-73f40edcbaf7&scheme=1)

[Click here to access/download](https://www.editorialmanager.com/pone/download.aspx?id=26278298&guid=0ca35459-9cd4-40ce-82c9-b9bb9fd89650&scheme=1)

[Supporting Information - Compressed/ZIP File Archive Figure. TIFF.zip](https://www.editorialmanager.com/pone/download.aspx?id=26278298&guid=0ca35459-9cd4-40ce-82c9-b9bb9fd89650&scheme=1)

Revised Manuscript with Track Changes

PM2.5 mass concentration ~~of PM2.5 was~~ generally ~~distributed~~ occurred in Beijing-Tianjin-Hebei and its surroundings in the eastern part of northern China and in Xinjiang in the western part of northern China. As ~~shown~~ indicated ~~in~~ in Table 1, the average ~~value of the~~ mass concentration of PM2.5 (unit: μg ~~·~~ m ) from 2014 to 2019 is 44.84, 42.03, 40.69, 35.17, 32.11, and 29.13. The PM2.5 concentration monotonically ~~decline~~ decreases ~~ofin PM2.5 concentration~~ from 2014 to 2019. As ~~shown~~ summarized in Table 1-1, the 10 cities with the worst PM2.5 pollution are ~~screened out~~highlighted ~~of~~ among the 367 cities each year, and these 10 cities are sorted by the annual PM2.5 concentration ~~each year~~. According to the statistics, nearly 5/6 of these ~~screened~~ highlighted cities are located in Beijing-Tianjin-Hebei and its surrounding areas, and ~~nearly 1/6~~the remainder are located in the Xinjiang region. Xingtai and~~,~~ Baoding~~,~~ in the Beijing-Tianjin-Hebei r~~R~~egion of China ~~have~~ ~~been~~are ~~selected as~~ cities with high PM2.5 ~~concentration~~concentrations ~~in China~~ for 6 consecutive years~~.~~, while Kashgar and Hotan in the Xinjiang r~~R~~egion ~~were~~ ~~selected~~are ~~as a city~~ cities with ~~a~~ high PM2.5 ~~concentration~~concentrations ~~in China~~ for 5 consecutive years.

-

3

**Formatted:**

Font:

12

pt,

Superscript

**Formatted:**

Font:

12

pt

**Formatted:**

Font:

12

pt,

Not

Bold

~~NoteNotes:~~ Beijing-Tianjin-Hebei~~:~~ encompasses Beijing, Tianjin, and Hebei Provinces~~;~~, and the surrounding areas of Beijing-Tianjin-Hebei include the regions bordered by Beijing, Tianjin, and Hebei, ~~,~~such as Shandong, Henan, Shanxi, and

Liaoning Provinces.

153

154

155

156

157

**Figure 1:**

**~~Spatio~~**

**~~-~~**

**~~temporal~~**

**Spatiotemporal**

**changes**

**~~of~~**

**in**

**the**

**annual average**

158

**PM2.5**

**concentration**

**in China**

**~~'s~~**

**~~land~~**

**from 2014 to 2019**

159

160

**Table 1: Statistical**

**~~Analysis~~**

**analysis**

**of**

**the**

**PM2.5**

**~~Mass~~**

**mass**

**~~Concentration~~**

161

**Formatted:**

Font:

)

(

Default

Times

New

Roman,

12

pt

**Commented [Ed7]:**

In this figure, please make the

following change(s):

"PM2.5 Of China Land In 2014" to "PM2.5 Concentration

in China in 2014"

**Formatted:**

Font:

)

Default

(

Times

New

Roman,

12

pt

**Formatted:**

Font:

(

Default

)

Times

New

Roman,

12

pt

**Formatted:**

Font:

)

(

Default

Times

New

Roman,

12

pt

**Formatted:**

Font:

(

)

Default

Times

New

Roman,

12

pt

**Formatted:**

Font:

12

pt

China and Xinjiang in the western part of northern China.

~~As shown in~~

Table 3

~~,~~

217

indicates

that

the average PM10 mass concentration (unit: μg·m

-

3

for each year from

)

218

2014

to 2019 is 88.51, 88.2, 87.53, 77.96, 82.09,

and

66.31

,

reflecting a

~~the~~

219

monotonic decline

in

the

~~of~~

PM10 concentration

~~from 2014 to 2019~~

.

~~As shown in~~

In

220

Table 3

-

1

, 10 cities with the most severe PM10 pollution are

~~screened~~

identified

out

221

of

the

367

cities

~~each year~~

annually

, and the

se

~~n~~

~~the~~

cities are

10

then

sorted according

222

to the PM10 concentration. According to

the

statistical analysis

results

, nearly 1/2 of

223

these

~~screened~~

identified

cities are located in the Beijing

-

Tianjin

-

Hebei region and its

224

surroundings,

and nearly 1/2 of the cities are

located

in the

Xinjiang region. Handan

225

in

the

Beijing

-

Tianjin

-

Hebei region and Kashgar, Hetian, Aksu, and Kezhou in

the

226

Xinjiang region

in China

have been

~~selected~~

identified

as cities with high

~~er~~

PM10

227

concentrations

~~in China~~

for 5 to 6 years.

228

229

230

231

**Formatted:**

Font:

12

pt,

Superscript

**Formatted:**

Font:

12

pt

**Formatted:**

Font:

(

Default

)

Times

New

Roman,

12

pt

**Formatted:**

Line

spacing:

Double

**Formatted:**

Font:

)

(

Default

Times

New

Roman,

12

pt

mass to	the

consecutive years. Beijing,

It is worth noting that

the

CO

~~appeared~~

concentration in Tibet was high

~~in~~

282

~~Tibet~~

~~in 2018~~

in 2018

and 2019

~~;~~

, and the

CO

concentration

~~has been~~

283

~~decreasing~~

decreased

from 2014 to 2018,

~~and~~

after which

it

~~then~~

~~the concentration~~

~~has~~

284

increased from 2018 and 2019. These two

~~points~~

trends

are different from

that of

the

285

matter

particulate

distribution

spatiotemporal

~~matter~~

~~particulate~~

~~the~~

~~of~~

286

~~mentioned~~

described

above. Regarding this point, we will perform a statistical analysis

287

on the correlation between the

particulate matter and CO

spatiotemporal distribution

s

288

~~of particulate matter and CO~~

to further

~~verify~~

support

our

~~point of view~~

finding

.

289

290

291

292

293

**Formatted:**

Indent:

First

line:

3

ch,

Line

spacing:

Double

**Formatted:**

Font:

)

Default

(

Times

New

Roman,

12

pt

**Formatted:**

Font:

(

Default

)

Times

New

Roman,

12

pt

**Formatted:**

Line

spacing:

Double

**Formatted:**

Font:

(

)

Default

Times

New

Roman,

12

pt

in areas where the economy is developed and the vehicle population is large

(

for

328

example

,

in the Yangtze River Delta such as Sha

nghai, in the Pearl River Delta such

329

as Guangzhou

~~, etc.~~

,

~~an~~

and

in the Sichuan Basin such as Chongqing

, which

are all

330

economically developed areas). Motor vehicle exhaust

~~,~~

is

one of the reasons

~~to~~

331

~~maintain~~

causing

a high

NO2

concentration

~~of NO2~~

[29

-

30].

From this

~~point of~~

332

~~view~~

perspective

, the spati

~~al~~

~~-~~

o

temporal distribution of NO2 is slightly different from

333

those

of

~~the~~

atmospheric particulate matter PM2.5

~~, atmospheric particulate matter~~

and

334

PM10

~~,~~

and AQI.

335

However, the temporal and spatial

~~distribution~~

dist

ributions

of NO2

,

~~and~~

336

atmospheric particulate matter PM2.5

~~, atmospheric particulate matter~~

and

PM10

~~,~~

and

337

AQI are

~~almost the same~~

very similar

in

the

Beijing

-

Tianjin

-

Hebei region and its

338

surrounding areas and

in

Xinjiang. We will

~~discuss~~

examine

the correlations

~~of~~

339

between

spati

~~al~~

~~-~~

o

temporal distribution

s

~~between~~

of

pollution

~~polluted~~

gas NO2 and

340

the

atmospheric particulate matter below.

~~The~~

A

statistical analysis of the correlations

341

will

further validate

~~s~~

our

~~views~~

findings

.

342

343

344

**Formatted:**

Font:

)

(

Default

Times

New

Roman,

12

pt

**Formatted:**

Line

spacing:

Double

GDP increased by 52% in 2014 ~~compared to 2010~~. The ~~strength~~ magnitude of the ~~country's~~ consumption in China of the accumulated resources ~~in~~ per unit of land area ~~of~~ ~~per unit,per unit time~~over time and the intensity of pollutant emissions are very high [14].

~~The~~ In the Beijing-Tianjin-Hebei region, ~~takes~~ Beijing is selected as an example. The analysis results of the~~Beijing ’s Beijing’s~~ air pollution sources in

Beijing released ~~in 2014~~ in 2014 [15] ~~show~~ reveal that ~~about~~approximately 28% to

36% of the atmospheric PM2.5 ~~sources~~ come from regional ~~transmission~~transport. Local emissions account for ~~about~~approximately 64%-72%, including 31.1% ~~of~~ from motor vehicles, 22.4% ~~of~~ from coal, 18.1% ~~of~~ from industrial production, 14.3% ~~of~~ from dust~~,~~ and 14.1% from other activities, such as catering, livestock and poultry breeding, and ~~auto repair accounted~~ automobile repair~~, accounting for 14.1%~~. ~~It can~~ ~~be seen that vehicle~~ Vehicle exhaust is the most important source of local pollution. The structures of the primary ~~industry~~, secondary ~~industry,~~ and tertiary industries~~y of~~ in Beijing were adjusted from 0.9: 24. 0: 75. 1 ~~in 2010~~ in 2010 to 0.7: 21. 4: 77. 9 in 2014. The tertiary industry ~~areis dominated~~dominates, and compared to 2010, ~~their~~ its proportion has increased by nearly 3% ~~compared with 2010~~, indicating that ~~Beijing's~~ the industrial structure of Beijing is still ~~optimizing~~being optimized. The ~~source of~~ air pollution is mainly caused by the industrial production process of the secondary industry and vehicle exhaust emissions.

**Formatted:**

Font:

12

pt,

Not

Bold

~~Notes:~~ The primary industry mainly refers to those industries that produce foodstuffs and other biological materials, including crops, forestry, animal husbandry, aquaculture and other industries that directly target natural objects. The secondary industry mainly refers to the processing and manufacturing industry, which ~~uses~~ consumes the basic materials provided by nature and the primary industry ~~for~~ during processing. The tertiary industry refers to those ~~other~~ industries other than the primary

and	secondary	industries~~,~~	and	has	a	wide

nonmaterial~~-material~~ production sectors such as transportation, communications, commerce, catering, finance, education, and public services.

The results of a new round of PM2.5 source analysis completed by the Beijing Environmental Protection Monitoring Center ~~in 2018~~ in 2018 were released to the public, and~~.~~ t~~T~~he main conclusions of this study indicate that 2/3 of the main sources of PM2.5 in Beijing throughout the year are local and 1/3 are regional transmissions. From the characteristics of ~~the city ’scity’s~~the current local atmospheric PM2.5 sources in Beijing, in terms of the ~~local emissions~~ contribution of local emissions, mobile sources ~~are~~ account for 45%, ~~flying~~ fugitive dust sources account for ~~are~~ 16%, industrial sources account for ~~are~~ 12%, living surface sources account for ~~are~~ 12%, coal sources account for ~~are~~ 3%, and agricultural, natural ~~sources~~and~~,~~ other~~s~~ sources account for ~~about~~approximately 12% of the PM2.5 concentration. The results show that at this stage, the coal-fired source has basically ~~withdrawn from the ranks~~ ~~of~~stopped being the ~~city's~~ main source of atmospheric PM2.5 in Beijing [58].

mainly

range,

including

**Formatted:**

Font:

12

pt

**Formatted:**

Font:

(

Default

)

Times

New

Roman,

12

pt

**Formatted:**

Font:

12

pt

~~T~~In the Beijing-Tianjin-Hebei region, ~~takes~~ Tianjin is selected as an example.

The analysis results of the air pollution sources in Tianjin ~~in 2014~~ in 2014 showed [16] that the regional transmission of atmospheric PM2.5 sources accounted for 22%-34% of the pollution. The remaining 66% to 78% came from local emissions. Among them, dust sources accounted for 30%, coal-fired sources accounted for 27%, motorized sources accounted for 20%, industrial production sources accounted for 17%, and other sources accounted for 6%. Dust is the primary source of pollution, followed by coal combustion and motor vehicles. The r~~R~~oads, storage yards, construction sites, etc. in Tianjin are the main sources of dust. In addition, construction projects in the railway, chemical, and power industries will also generate dust. In 2014, the three industrial structures in Tianjin were adjusted from 1.6: 53.1: 45.3 ~~in 2010~~ in 2010 to 1.3: 49.4: 49.3. The secondary industry ~~dropped~~ decreased ~~by~~ 3.7% ~~percentage points~~, and the tertiary industry increased ~~by~~ 4% ~~percentage points~~. Until 2014, the proportions of the secondary and tertiary industries in Tianjin ~~was basically~~ ~~flat~~practically remained stable. ~~Comparing~~ In comparing the data, it is found that the proportion of the ~~Tianjin's~~ secondary industry in Tianjin is still the highest despite ~~a~~ its decline. Thermal power, iron and steel, petrochemical, cement, building materials, etc., as typical ~~heavy~~ high-pollution emission industries, are the key sources ~~of~~ ~~pollution caused by~~causing the air pollution in Tianjin.

**Formatted:**

Font:

(

Default

)

Times

New

Roman,

12

pt

**Formatted:**

Font:

12

pt

The ~~A~~analytical results of the air pollution sources in Hebei Province in the Beijing-Tianjin-Hebei region ~~show~~ reveal that [17], ~~taking~~ with Shijiazhuang city as an example, regional transmission accounts for 23% to 30% of the PM2.5 ~~sources~~pollution, and local ~~pollution~~ emissions account~~s~~ for 70% to 77%, of which

coal combustion accounts for 28.5%, industrial production accounts for 25.2%, fugitive dust accounts for 22.5%, motor vehicles account for 15.0%, and other sources such as biomass burning~~,~~ and catering~~, etc.~~ account for 8.8% of the PM2.5 pollution. Coal combustion is the primary source of the air pollutants in Shijiazhuang and even in Hebei Province. ~~Large~~ The high coal consumption and irrational coal combustion ~~structure~~structure~~s~~ are the main causes of soot pollution. In 2014, the three industrial structures in Hebei Province were adjusted from 12.57: 52.5: 34.93 ~~in 2010~~ in 2010 to 11.7: 51.1: 37.2. The primary industry ~~declined~~ decreased slightly, and the secondary industry decreased ~~by~~ 1.4 %~~percentage points~~, ~~and~~ while the tertiary industry increased ~~by~~ approximately 2-3 %~~percentage pointpoints~~. The proportion of the secondary industry in Hebei Province is still higher than 50%. This irrational industrial structure makes Hebei Province the region with the worst air pollution in the Beijing-Tianjin-Hebei. In addition, Hebei has a ~~large~~ high proportion of high-pollution and high-energy~~- consuming~~ consumption industries, including steel, cement, electricity, glass, pharmaceuticals, metallurgy, petrochemicals, and building materials. Among them, the coal consumption in the steel, building materials and power industries accounted for 89.6% of the ~~province's~~ provincial total energy consumption, and the air pollutant emissions ~~in~~ from the steel, cement, power, and glass industries accounted for ~~about~~approximately 60% of the ~~Hebei's~~ total pollution in Hebei~~.~~ [18]. As the most important pollution industry in Hebei, steel is the focus of air pollution control.

~~accumulation[19~~

regional pollution accumulation easily occurs

[19

-

20]

. Wind has a

656

certain diffusion and purification ability,

and

the

wind speed and pollutant

657

concentration are basically negatively correlated.

~~Taking~~

With

Urumqi as an example,

658

the frequency of static

~~wind~~

atmospheric conditions

in the heating period is as high as

659

~~about~~

approximately

26

%, and in December and January

,

it is as high as 30% or more.

660

Even if there is wind, the wind speed is very

~~small~~

low

and rar

ely exceeds level 3 [21].

661

Such meteorological conditions are extremely detrimental to

~~the spread of~~

pollutant

~~s~~

662

dispersal

. If there are several

consecutive

days of static

~~wind~~

weather

conditions

,

~~the~~

663

pollutants

will

continue to accumulate, and the air

quality will deteriorate sharply

664

[22].

665

**Sand storm**

**s**

666

Sandstorms are a typical weather phenomenon in Xinjiang. Sandstorms and floating

667

dust occur throughout the year. Sandstorms and floating dust weather

conditions

will

668

cause high

~~concentrations of~~

atmospheric particulate matter

concentrations

, which

669

will seriously affect

~~all~~

the

~~regions~~

~~regions’~~

regional

air quality in cities [23]. The

670

source region of western China, with the Taklimakan Desert as the main

~~body~~

region

,

671

and the source region of northern

China, with the Badain Jaran Desert as the center,

672

contributed 70% of the total dust emissions in Asia.

The s

~~S~~

andstorms in Northwest

673

China are dominated by dry settlement

[24]

.

~~Regional~~

The regional

distribution of

the

674

sandstorms in Northwest China is very

uneven

,

while the sandstorm frequency in

the

675

**Formatted:**

Font:

14

pt

**Formatted:**

Line

spacing:

Double

**Formatted:**

Font:

12

pt

**Formatted:**

Font:

)

Default

(

Times

New

Roman,

12

pt

**Formatted:**

Font:

12

pt

~~conditions~~

. The PM2.5 released from food sources is mainly from the cooking process

738

~~of~~

involving

edible oils and

foods. Under high

-

temperature conditions, particulate

739

matter

is

formed by chemical reactions such as thermal oxidation and

~~thermal~~

740

decomposition. Most

~~of the~~

food and beverage sources

~~are~~

occur

in densely populated

741

areas, and pollutants are emitted near the ground, which has an

~~important~~

notable

742

impact on the health of the population. According to activity

-

level survey

743

information, there were a total of 3,443 catering companies in Turpan in 2017

. The

744

total PM10 emissions were 301.6 tons

,

and the total PM2.5 emissions were 241.3 tons.

745

In addition, the exhaust gas from restaurant

~~fume~~

s contains a large number of

~~volatile~~

746

~~organic pollutants~~

~~pollutant~~

VOCs, among which oxygenates and olefins have

a hi

gh

747

photochemical activity

,

~~,~~

which

are precursors to

~~form~~

secondary particulate matter

~~,~~

748

~~and have~~

and have

~~an important~~

a notable

impact on atmospheric particulate

749

pollution [28].

750

**Mobile source**

751

At present, vehicle exhaust is one of the main sources of

the

atmospheric

752

particulate matter in China [29

-

30]

, especially

~~PM2.5~~

fine particulate matter

PM2.5

.

753

Motor vehicles also emit gaseous pollutants such as NOx and VOCs, which are

754

converted into secondary particulate matter through photochemical reactions.

755

Co

mpared

~~with~~

to

coarse particles, fine particles have a larger specific surface area,

756

~~which~~

thus capable of

absorb

~~s~~

ing

and carr

ying

~~ies~~

more toxic substances, which

~~is~~

can

757

**Formatted:**

Font:

14

pt

**Formatted:**

Font:

12

pt

and in the Sichuan Basin, such as Chongqing ~~etc.~~.

The O3 level has consistently been ~~stably concentrated~~high in Inner Mongolia,

Qinghai, Gansu and Shandong ~~for 6 years~~ from 2014 to 2019.

There is often a significant correlation between the mass concentrations of the different types of particulate matter. ~~the~~The ~~coefficient~~ R coefficient of the correlation between PM2.5 and PM10 is 0.88. There is a particularly significant linear positive correlation between these particulates, which indicates that ~~the two~~they ~~have~~ experience similar changes in the ambient air pollution and ~~basically~~ practically follow the same migration and transformation laws.

There is a significant positive linear ~~positive~~ correlation between the atmospheric particulate matter, AQI and pollut~~ant~~ion gases ~~(~~NO2~~,~~ and SO2~~)~~. The correlation coefficient between the atmospheric particulate matter (PM2.5~~,~~ and PM10)~~，AQI~~, AQI and SO2 is ~~(~~0.97, 0.81, and 0.88~~);~~, respectively, and the correlation coefficient between the atmospheric particulate matter (PM2.5~~,~~ and PM10), AQI and NO2 is ~~(~~0.82, 0.62, and 0.72, respectively, which is~~),~~ due to the mutual conversion caused by the chemical reaction between the particulate matter and gaseous

**Formatted:**

Indent:

First

line:

3.5

ch,

Line

spacing:

Double

**Formatted:**

Indent:

First

line:

3

ch,

Line

spacing:

Double

pollutants.

There ~~is a~~exist certain positive and negative correlations between the gaseous pollutants. There is a particularly significant linear positive correlation between N02 and SO2 (the correlation coefficient is 0.90), indicating that these two pollutants follow the same evolution law, and their influencing factors may be similar. N02 and

3. U.S. Geological Survey.https://www.usgs.gov/.

4.

University of Scienceand Technology Beijing,2014, 36(9):1269-1279.

5.

Beijing City Journal of Safety and Environmen,2015,15(6):333-339.

6.

7.

Liu Jie,Yang,Peng,Lv,Wensheng, Pollution characteristics of particulate and

interpolation analysis of

its spatial distribution in the Beijing [J]. Journal of

Liu Jie,Yang,Peng,Lv,Wensheng,L

iu

~~IU~~

Agudamu,L

iu

~~IU~~

Junxiu,Study on the

variation of mass concentration of atmospheric particles in the 6th district of

Pandis S N; Harley R A, Cass G R, et al. Secondary organic aerosol formation

and transpo

rt[J]. Atmospheric Environment Part A, 1992, 26(13):2269

-

2282.

West J J, Ansari A S, Pandis S N. Marginal PMz.s:nonliner aerosol mass response

to sulfate reductions in the eastern United States[J]. Journal of the Air&Waste

Chan Y C, Simpson R W, Mctainsh G H, et al. Characterization of chemical

species in PM2.5 and PM10 aerosols in Brisbane, Australia[J]. Atmospheric

10.

Liu Jie, Zhang Xiaoling, Xu Xiaofeng, et al. Comparative analysis of suburban

SO2, NOX, O3 and PM2.5 changes in Beijing [J]. Environmental Science, 2008,

**Formatted:**

Font:

12

pt

**Formatted:**

Font:

12

pt

Manage Association, 1999, 49(12):1415-1424.

8.

Environment, 1997, 31(22):3773-3785.

9. Yu S, Xiaoyan T, Chen F, et al. Source apportionment on fine particles in Beijing[J]. Chinese Journal of Environmental Science, 2002, 23(6):11-16.

29(4):1059-1065.

11. Communique of the State of Beijing Environment in 2018.
12. Liu Jie,Yang,Peng,Lv,Wensheng,LIU Agudamu,LIU Junxiu,Study on the variation of mass concentration of atmospheric particles in the 6th district of Beijing City Journal of Safety and Environmen,2015,15(6):333-339.
13. Wang Yanhuan. Analysis of the external causes of urban smog and its impact on the public[D]. Hefei: University of Science and Technology of China，2015.
14. Mao Xianqiang, Xing Youkai, Hu Tao, et al. Analysis of environmental and economic pathways for coordinated emission reduction of sulfur, nitrogen and carbon in China's power industry[j].Chinese Environmental Science,2012, (4) :

748-756.

15. Communique of the State of Beijing Environment in 2014.
16. Communique of the State of Tianjin Environment in 2014.
17. Communique of the State of Hebei Environment in 2014.
18. Mao Hengqing.Effects of meteorological conditions on the diffusion of atmospheric pollutants [J] .Shandong Meteorology, 1991, 4:2.
19. Li Yan, Wang Youqiang, Liu Qiang.Effects of weather and climate conditions on the distribution of atmospheric pollutants [J]. Anhui Agricultural Sciences, 2009.

37(4): 1781-1782.

20. Law of the People's Republic of China on Air Pollution Control, 2000.
21. Shi Haoling. Analysis of Air Pollution Status and Major Pollutants Sources in Urumqi (M). Xinjiang Agricultural University. 2012.
22. Tao Jianhong.Climatic Characteristics and Impact of Sand and Dust Weather in Northwest China [D] .Nanjing University of Information Technology,2007.
23. Zhang Xiaoqi.Re-review on the source area of loess material and its transportation method in the Loess Plateau of China [J] .Quaternary Research,2007, 27(2): 181-186.
24. Wang Cunzhong, Niu Shengjie, Wang Lanning.Characteristics of sandstorm changes in China in the past 50 years [J] .China Desert,2010, 30(4):933-939.
25. 2000-2010 Statistical Yearbook of Chinese Cities, Statistical Yearbook of Autonomous Regions, Statistical Yearbook of Urumqi City, Statistical Yearbook of Urumqi County, Yearbook of Water Resources of Autonomous Regions [R].
26. Liu Qiaojing.Pollution characteristics and source analysis of atmospheric particulate matter in Turpan city (M). Zhejiang University. 2018.
27. Zhao X, Hu Q, Wang X, et al. Composition profiles of organic aerosols from

Chinese residential cooking: case study in urban Guangzhou, south China [J].Journal of Atmospheric Chemistry. 2015, 72(1): 1-18.

28. Kong Shaofei, Bai Zhipeng, Research Progress of Motor Vehicle Exhaust

Composition Spectrum in Source Analysis of Atmospheric Particulate Matter

[J] .Environmental Science and Technology. 2013(10): 26-33.

29. Yao Z, Shen X, Ye Y, et al. On-road emission characteristics of VOCs from diesel trucks in Beijing, China[J]. Atmospheric Environment. 2015, 103: 87-93.
30. Sun Guojin.Characteristics of PM2.5 and NOx Emissions from Motor Vehicles and Countermeasures for Emission Reduction [D] .Zhejiang University, 2013.
31. Louie P K K, Ho J W K, Tsang R C W, et al. VOCs and OVOCs distribution and control policy implications in Pearl Environment. 2013, 76: 125-135.Delta region, China [J]. Atmospheric Environment. 2013, 76: 125-135.
32. Shen Xianbao.Research on the characteristics of fine particulates in smoke plume of diesel vehicles [D]. Tsinghua University, 2015.
33. Tan Hua, Liu Juan, Shen Yin, et al. Research on air pollutants emission inventory of dock port farming machinery [J] .Environmental Science and Management. 2013 (06): 82-87.
34. Lu Jun, Huang Cheng, Hu Yuyao, et al. Inventory of atmospheric pollutants from non-road mobile machinery in typical cities in the Yangtze River Delta region [J]. Environmental Science.2017, 38(7): 2738-2746.
35. Li Zhan, Yang Huigai, Jiang Yan, Yin Yuanchang, Wang Hongqian, Wang Bo, Wang Bin,The Study on Spatial Distribution of Atmospheric Contaminant

Concentrations and Optimization of Air Quality Monitoring Sites in Beijing City[j]. Environmental Monitoring in China2015, 31(1):74-78.

36. Luo Xiongbiao. Sources and Control of Ozone Pollutants [J]. Resource

References

1017

1

.

China National Environmental Monitoring Centre.http://www.cnemc.cn/.

1018

2

.

Environmental

Zhenqi

Hangzhou

China

1019

Protection.

[/](https://www.zq12369.com/)

[https://www.zq12369.com](https://www.zq12369.com/)

[.](https://www.zq12369.com/)

1020

.

11

Communique of the State of

Beijing Environment in 2018

.

1021

.

12

Liu Jie,Yang,Peng,Lv,Wensheng,LIU Agudamu,LIU Junxiu,Study on the

1022

variation of mass concentration of atmospheric particles in the 6th district of Beijing

1023

City Journal of Safety and Environmen,2015,15(6):333

-

339.

1024

1025

**Figure**

**S**

**1:**

**Spatiotemporal**

**changes**

**in**

**the annual average PM2.5**

**concentration**

1026

**in China from 2014 to 2019**

1027

F

rom 2014 to 2019, a high PM2.5 mass concentration generally occurred in

1028

Beijing

-

Tianjin

-

Hebei and its surroundings in the eastern part of northern China and

1029

in

Xinjiang in the western part of northern China.

1030

1031

**Formatted:**

Font:

12

pt,

Not

Bold

**Formatted:**

No

bullets

or

numbering

**Formatted:**

Line

spacing:

Double,

No

bullets

or

numbering

**Formatted:**

No

bullets

or

numbering

**Formatted:**

Font:

)

Default

(

+

Body

(

Calibri),

10.5

pt

**Formatted:**

Line

spacing:

Double,

No

bullets

or

numbering

**Formatted:**

Justified

1032

**Figure**

**S**

**1**

**.**

**Spatiotemporal**

**changes**

**in**

**the annual average PM2.5**

**concentration**

**in**

1033

**China from 2014 to 2019**

1034

1035

**Figure**

**S**

**2:**

**Spatiotemporal**

**changes**

**in the**

**annual average AQI value in China**

1036

**from 2014 to 2019**

1037

F

rom 2014 to 2019, a high AQI also generally occurred in the Beijing

-

Tianjin

-

Hebei

1038

region and its surroundings in the eastern part of northern China and in Xinjiang in

1039

the western part of northern China.

1040

1041

1042

**Figure**

**S**

**2**

**.**

**Spatiotemporal**

**changes**

**in the**

**annual average AQI value in China**

1043

**from 2014 to 2019**

1044

**Formatted:**

Left

**Formatted:**

Justified

1045

**Figure**

**S**

**3:**

**Spatiotemporal**

**changes**

**in**

**the annual average PM10**

**concentration**

**in**

1046

**China from 2014 to 2019**

1047

F

rom 2014 to 2019, a high PM10 mass concentration generally occurred in the

1048

Beijing

-

Tianjin

-

Hebei

region

and its surrounding areas in the eastern part of northern

1049

China and Xinjiang in the western part of northern China.

1050

1051

1052

**Figure**

**S**

**3**

**.**

**Spatiotemporal**

**changes**

**in**

**the annual average PM10**

**concentration**

**in**

1053

**China from 2014 to 2019**

1054

1055

**Figure**

**S**

**4:**

**Spatiotemporal**

**changes**

**in**

**the annual average CO**

**concentration**

**in**

1056

**China from 2014 to 2019**

1057

F

rom 2014 to 2019, a higher CO mass concentration was generally observed in

1058

the Beijing

-

Tianjin

-

Hebei region

and its surrounding areas in the eastern part of

1059

**Formatted:**

Left

**Formatted:**

Justified

**Formatted:**

Left

**Formatted:**

Line

spacing:

Double,

No

bullets

or

numbering

northern

China and Xinjiang in the western part of northern China

(

it

is worth noting

1060

that a high CO mass concentration was observed in Tibet in 2018 and 2019).

1061

1062

1063

**Figure**

**S**

**4**

**.**

**Spatiotemporal**

**changes**

**in**

**the annual average CO**

**concentration**

**in**

1064

**China from 2014 to**

**2019**

1065

1066

**Figure**

**S**

**5:**

**Spatiotemporal**

**changes**

**in**

**the annual average NO2**

**concentration**

**in**

1067

**China from 2014 to 2019**

1068

F

rom 2014 to 2019, a higher NO2 mass concentration

generally occurred in the

1069

Beijing

-

Tianjin

-

Hebei region and its surroundings

in the eastern part of

northern

1070

China and in Xinjiang in the western part of northern China.

1071

1072

**Formatted:**

Line

spacing:

Double,

No

bullets

or

numbering

**Formatted:**

Left

**Formatted:**

Line

spacing:

Double,

No

bullets

or

numbering

1073

**Figure**

**S**

**5**

**.**

**Spatiotemporal**

**changes**

**in**

**the annual average NO2**

**concentration**

**in**

1074

**China from 2014 to 2019**

1075

1076

**Figure**

**S**

**6:**

**Spatiotemporal**

**changes**

**in**

**the annual average O3**

**concentration**

**in**

1077

**China from 2014 to 2019**

1078

A

higher O3 mass concentration has

stably occurred

in Inner Mongolia, Qinghai,

1079

and Gansu in northern China from 2014 to 2019

.

1080

1081

1082

**Figure**

**S**

**6**

**.**

**Spatiotemporal**

**changes**

**in**

**the annual average O3**

**concentration**

**in**

1083

**China from 2014 to 2019**

1084

1085

**Formatted:**

Left

**Formatted:**

Line

spacing:

Double,

No

bullets

or

numbering

**Figure**

**S**

**7:**

**Spatiotemporal**

**changes**

**in**

**the annual average SO2**

**concentration**

**in**

1086

**China from 2014 to 2019**

1087

A

higher

SO2

the

in

observed

generally

was

concentration

mass

1088

Beijing

-

Tianjin

-

Hebei region and its surrounding areas

in the eastern part of northern

1089

China and in Xinjiang in the northwest of mainland China.

1090

1091

1092

**Figure**

**S**

**7**

**.**

**Spatiotemporal**

**changes**

**in**

**the annual average SO2**

**concentration**

1093

**in China from 2014 to 2019**

1094

References

1095

1

.

China National Environmental Monitoring

Centre.http://www.cnemc.cn/.

1096

~~36.~~

2

.

China

Hangzhou

Zhenqi

Environmental

1097

Protection.

[/](https://www.zq12369.com/)

[https://www.zq12369.com](https://www.zq12369.com/)

1098

**Formatted:**

Left

**Formatted:**

Justified

**Formatted:**

Centered,

Line

spacing:

Double,

No

bullets

or

numbering

**Formatted:**

Left,

Line

spacing:

Double,

No

bullets

or

numbering

**Formatted:**

No

bullets

or

numbering

**Formatted:**

Line

spacing:

Double,

No

bullets

or

numbering

**Formatted:**

Font:

12

pt,

Bold,

Font

color:

Auto

Response to Reviewers

Dear Zongbo Shi

Academic Editor

PLOS ONE,

Revision required [PONE-D-19-35106] - [EMID:b2ec848001643238]

Please find attached a revised version of our manuscript Study on the Spatiotemporal Characteristics of the Air Pollutants in China from 2014 to 2019.

The reviews's comments were highly insightful and enabled us to greatly improve the quality of our manuscript.

We hope that the revisions in the manuscript and our accompanying responses will be sufficient to make our manuscript suitable for publication in the Journal of PLOS ONE.

*Reviewer #1: The main issue with this manuscript is the quality of the language. The authors clearly struggle with both grammar and choice of vocabulary, making it quite difficult to follow their arguments. Since there is a lot of ambiguous phrasing with unclear word choices, the questions of whether the manuscript is technically sound and whether the data supports the conclusions cannot be fully answered at this point. There are several sections which are completely unintelligible to me. I appreciate that it can be quite difficult to write in a non-native language and that some mistakes are to be expected. However, in this case it really impedes proper understanding of the scientific content. I highly recommend that the authors try to get some assistance with the language when revising the manuscript.*

Response: We agree with this suggestion, we have improved the English writing by American Journal Experts in the revised manuscript.

*Additional comments:*

*While the authors have made all the data publicly available, the origin of the data is not clearly described. I am assuming that the data was extracted from databases of national/regional measurement networks, however this is not clear from the description and the reference links provided go to Chinese webpages, making them inaccessible to a large percentage of PLOS One readers. In addition, units are missing, and it would be helpful to provide the English names of the different locations in addition to the Chinese characters.*

Response: We agree with this suggestion, we provided his English name and his website(page 2-3, lines 41-54). In this article, the air pollution parameter data of 367 cities from 2014 to 2019 are sourced from the China National Environmental Monitoring Centre (English name).The website of the China Environmental Monitoring Centre is <http://www.cnemc.cn/> or [http://www.cnemc.cn/en/.](http://www.cnemc.cn/en/)

And provided the data acquisition address of the China Environmental Monitoring Centre,making them inaccessible to a large percentage of PLOS One readers.The data acquisition address of the China Environmental Monitoring Centre is http://106.37.208.233:20035/. On this page, you can obtain real-time data on 7 air pollution parameters (PM2.5, PM10, air quality index (AQI), CO, NO2, O3, and SO2), including the mass concentration, of 367 cities, or you can go to <https://www.zq12369.com/> to acquire real-time historical data from the China Environmental Monitoring Centre.

*Additional comments:*

*The general idea behind the statistical analysis seems valid, but again there is a lack of information here regarding the input parameters of the radial basis function interpolation (e.g which basis function was used?). If the authors feel that this level of detail will not be of interest to most readers, it should at least go into the supplement.*

Response: We agree with this suggestion,information here regarding the input parameters of the radial basis function interpolation (page 4-5, lines 66-87).

This paper uses ArcGIS software to perform radial basis function (RBF) interpolation on the obtained air pollutant parameter data based on the abovementioned map of China.

This paper uses RBF interpolation. RBF interpolation is conducted to generate a smooth surface based on the large number of data points. These functions produce good results for gently changing surfaces, such as the elevation. There are a variety of RBFs to choose from in the RBF interpolation method. The selected RBF in this study is a regular spline function. First, the spline function is explained, after which the regular spline function is described. The interpolation method applied via the spline function tool estimates values using a mathematical function minimizing the total curvature of the surface. This produces a smooth surface that only passes through the input points. The algorithm of the spline function tool uses the following surface interpolation equation:

where j = 1, 2, ..., N, N is the number of points, 𝜆_𝑗_ is the coefficient obtained by solving a system of linear equations, and 𝑟_𝑗_ is the distance between point (x, y) and the j-th point.

The definitions of T(x, y) and R(r) will differ depending on the RBF selected. The regular spline function is defined as:

(𝑥, 𝑦) = 𝑎_1_ + 𝑎_2_𝑥 + 𝑎_3_𝑦

where 𝑎_𝑖_ is the coefficient obtained by reconstructing the system of linear equations, and

*Additional comments:*

*The results of the analysis are not put into* context *and there are no comparisons with other studies of air pollution trends.*

Response: We agree with this suggestion,rewritten the results of the analysis and put them into context (page 33-34, lines 619-658).

The PM2.5, PM10, AQI, CO, NO2, and SO2 levels in China exhibit a stable spatial distribution and persistence over time. PM2.5, PM10, AQI, CO, NO2, and SO2 are mostly concentrated in Beijing-Tianjin-Hebei and its surroundings and in Xinjiang from 2014 to 2019. The average concentration monotonically decreases (except CO).

A high NO2 level also occurs in areas where the economy is well developed and the vehicle population is large, including in the Yangtze River Delta, such as Shanghai, in the Pearl River Delta, such as Guangzhou, and in the Sichuan Basin, such as Chongqing.

The O3 level has consistently been high in Inner Mongolia, Qinghai, Gansu and Shandong from 2014 to 2019.

There is often a significant correlation between the mass concentrations of the different types of particulate matter. The R coefficient of the correlation between PM2.5 and PM10 is 0.88. There is a particularly significant linear positive correlation between these particulates, which indicates that they experience similar changes in the ambient air pollution and practically follow the same migration and transformation laws.

There is a significant positive linear correlation between the atmospheric

particulate matter, AQI and pollution gases NO2 and SO2. The correlation coefficient between the atmospheric particulate matter (PM2.5 and PM10), AQI and SO2 is 0.97, 0.81, and 0.88, respectively, and the correlation coefficient between the atmospheric particulate matter (PM2.5 and PM10), AQI and NO2 is 0.82, 0.62, and 0.72, respectively, which is due to the mutual conversion caused by the chemical reaction between the particulate matter and gaseous pollutants.

There exist certain positive and negative correlations between the

gaseous pollutants. There is a particularly significant linear positive correlation between N02 and SO2 (the correlation coefficient is 0.90), indicating that these two pollutants follow the same evolution law, and their influencing factors may be similar. N02 and SO2 are initially influenced by factors such as coal combustion and motor vehicle emissions, and there is no correlation between O3 and the other three gaseous pollutants.

The pollution sources in Beijing-Tianjin-Hebei area mainly include

decomposition, coal burning, industrial production, and dust. The industrial structure has been adjusted in favor of the tertiary industry, and high-pollution, high-energy consumption secondary industries have been reduced but still account for a large proportion. Regarding the pollution and landforms in Xinjiang, there is a close relationship between static weather conditions, sandstorms, inversion layer, precipitation, dust and industrial sources, human activities, and mobile and nonroad mobile sources.

And comparisons with other studies of air pollution trends (page 19-23, lines 341-414).

**Study on the Correlation Between the Particulate Matter and the AQI**

There is often a significant correlation between the mass concentrations

of different types of particulate matter. Table 8 summarizes the correlation between the hourly PM2.5 and PM10 mass concentrations from 2014 to 2019. The slope of the fitting equation between PM2.5 and PM10 is approximately 1, namely, 1.24. The correlation coefficient R is 0.88. The goodness of fit parameter R^2^ is 0.77. The mass concentration ratio of PM2.5 to PM10 is 46%, which is close to half, indicating that the degree of PM2.5 pollution in China is more severe. There is a particularly notable linear positive correlation between the two types of particulate matter, which indicates that they cause similar changes in ambient air pollution and practically follow the same migration and transformation laws. This is related to Liu Jie's research on the correlation of atmospheric particulates, and the results are consistent [4-5].

**Study on the Correlation Between the Atmospheric Particulate Matter, AQI and**

**Gaseous Pollutants**

From the correlation study between the particulate matter and gaseous pollutants, as summarized in Table 9, there is a significant linear positive correlation between the atmospheric particulate matter, AQI and pollution gases (NO2 and SO2). The correlation coefficient between the atmospheric particulate matter (PM2.5 and PM10), AQI and SO2 is 0.97, 0.81, and 0.88, respectively, while the correlation coefficient between the atmospheric particulate matter (PM2.5 and PM10), AQI and NO2 is 0.82, 0.62, and 0.72, respectively. The above results are also consistent with the findings of Li Zhan et al. [6]. The atmospheric particulate matter (PM2.5 and PM10) and AQI are positively correlated with the atmospheric pollution gases NO2 and SO2, which usually occurs due to the mutual conversion caused by the chemical reaction between the particulate matter and the gaseous pollutants [7- 8]. It was found that nitrate (sulfate) produced by NO2 (SO2) would be transformed into secondary particulate matter (PM2.5 and PM10) over time, and with increasing NO2 and SO2 atmospheric pollution, the atmospheric particulate matter PM2.5 and PM10 also increased. One of the reasons for the increase in atmospheric pollutants NO2 and SO2 is the large vehicle flows and high exhaust emissions [9-10].

**Study on the Correlation Between the Gaseous Pollutants**

The study of the mutual evolution of the gaseous pollutants is more complicated. Generally, there is no simple law describing their mass concentrations in the short term, but the correlation can be studied through the long-term change trend of their mass concentrations to determine their individual relationships. As indicated in Table 10, there are certain positive and negative correlations between the gaseous pollutants. In Table 10, there is a particularly significant linear positive correlation between N02 and SO2 (a correlation coefficient of 0.90), indicating that these two pollutants follow the same evolution law, and their influencing factors may be similar.

According to the analysis of the influencing factors of these two pollutants [11], the initial pollution of these two types can be determined. Both of these pollutants are influenced by factors such as coal combustion and motor vehicle emissions. There is no correlation between O3 and the other three gaseous pollutants [12]. Therefore, the evolution trends between the atmospheric particles, those between the atmospheric particles and gaseous pollutants and those between the gaseous pollutants are more complex. In addition, in-depth research is needed in many aspects, such as chemical reactions.

*Reviewer #2: This paper describes the spatial-temporal variation of atmospheric pollutants in the mainland of China during 2014 to 2019. The data in this paper covers hundreds of cities in China and 6 years. From this standpoint the article is useful and can be used to document levels at present day. However, I am not quite sure if the article fits the scope of the PLOS ONE because it looks like a data analysis report. I would suggest the author to expand the discussion on the reason of the variations.*

Response: We agree with this suggestion, so we have expanded the discussion on the reason of the variations(page 23-32, lines 416-617).

**Study on the Correlation Between the Particulate Matter and the AQI**

There is often a significant correlation between the mass concentrations of different types of particulate matter. Table 8 summarizes the correlation between the hourly PM2.5 and PM10 mass concentrations from 2014 to 2019. The slope of the fitting equation between PM2.5 and PM10 is approximately 1, namely, 1.24. The correlation coefficient R is 0.88. The goodness of fit parameter R^2^ is 0.77. The mass concentration ratio of PM2.5 to PM10 is 46%, which is close to half, indicating that the degree of PM2.5 pollution in China is more severe. There is a particularly notable linear positive correlation between the two types of particulate matter, which indicates that they cause similar changes in ambient air pollution and practically follow the same migration and transformation laws. This is related to Liu Jie's research on the correlation of atmospheric particulates, and the results are consistent [4-5].

**Study on the Correlation Between the Atmospheric Particulate Matter, AQI and**

**Gaseous Pollutants**

From the correlation study between the particulate matter and gaseous pollutants, as summarized in Table 9, there is a significant linear positive correlation between the atmospheric particulate matter, AQI and pollution gases (NO2 and SO2). The correlation coefficient between the atmospheric particulate matter (PM2.5 and PM10), AQI and SO2 is 0.97, 0.81, and 0.88, respectively, while the correlation coefficient between the atmospheric particulate matter (PM2.5 and PM10), AQI and NO2 is 0.82, 0.62, and 0.72, respectively. The above results are also consistent with the findings of Li Zhan et al. [6]. The atmospheric particulate matter (PM2.5 and PM10) and AQI are positively correlated with the atmospheric pollution gases NO2 and SO2, which usually occurs due to the mutual conversion caused by the chemical reaction between the particulate matter and the gaseous pollutants [7- 8]. It was found that nitrate (sulfate) produced by NO2 (SO2) would be transformed into secondary particulate matter (PM2.5 and PM10) over time, and with increasing NO2 and SO2 atmospheric pollution, the atmospheric particulate matter PM2.5 and PM10 also increased. One of the reasons for the increase in atmospheric pollutants NO2 and SO2 is the large vehicle flows and high exhaust emissions [9-10].

**Study on the Correlation Between the Gaseous Pollutants**

The study of the mutual evolution of the gaseous pollutants is more complicated. Generally, there is no simple law describing their mass concentrations in the short term, but the correlation can be studied through the long-term change trend of their mass concentrations to determine their individual relationships. As indicated in Table 10, there are certain positive and negative correlations between the gaseous pollutants. In Table 10, there is a particularly significant linear positive correlation between N02 and SO2 (a correlation coefficient of 0.90), indicating that these two pollutants follow the same evolution law, and their influencing factors may be similar. According to the analysis of the influencing factors of these two pollutants [11], the initial pollution of these two types can be determined. Both of these pollutants are influenced by factors such as coal combustion and motor vehicle emissions. There is no correlation between O3 and the other three gaseous pollutants [12]. Therefore, the evolution trends between the atmospheric particles, those between the atmospheric particles and gaseous pollutants and those between the gaseous pollutants are more complex. In addition, in-depth research is needed in many aspects, such as chemical reactions.

Causes of Air Pollution in the Beijing-Tianjin-Hebei Region

The unique geographical environment of the Beijing-Tianjin-Hebei region coupled with the rapid development of urban agglomerations and the formation of local atmospheric circulation formation conditions make result in the air pollution in the Beijing-Tianjin-Hebei region show exhibiting a certain particular spatiotemporal distribution distribution [13]. In 2014, the energy consumption level of

Beijing-Tianjin-Hebei accounted for 12% of the country's total energy consumption level of China. There are still more numerous heavyhigh-energy consumption heavy industries in the Beijing-Tianjin-Hebei region. Compared to 2010, In 2014, the overall GDP increased by 52% in 2014 compared to 2010. The strength magnitude of the country's consumption in China of the accumulated resources in per unit of land area of per unit,per unit timeover time and the intensity of pollutant emissions are very high [14].

The In the Beijing-Tianjin-Hebei region, takes Beijing is selected as an example. The analysis results of theBeijing ’s Beijing’s air pollution sources in Beijing released in 2014 in 2014 [15] show reveal that aboutapproximately 28% to 36% of the atmospheric PM2.5 sources come from regional transmissiontransport. Local emissions account for aboutapproximately 64%-72%, including 31.1% of from motor vehicles, 22.4% of from coal, 18.1% of from industrial production, 14.3% of from dust, and 14.1% from other activities, such as catering, livestock and poultry breeding, and auto repair accounted automobile repair, accounting for 14.1%. It can be seen that vehicle Vehicle exhaust is the most important source of local pollution. The structures of the primary industry, secondary industry, and tertiary industriesy of in Beijing were adjusted from 0.9: 24. 0: 75. 1 in 2010 in 2010 to 0.7: 21. 4: 77. 9 in 2014. The tertiary industry areis dominateddominates, and compared to 2010, their its proportion has increased by nearly 3% compared with 2010, indicating that Beijing's the industrial structure of Beijing is still optimizingbeing optimized. The source of air pollution is mainly caused by the industrial production process of the secondary industry and vehicle exhaust emissions.

Notes: The primary industry mainly refers to those industries that produce foodstuffs and other biological materials, including crops, forestry, animal husbandry, aquaculture and other industries that directly target natural objects. The secondary industry mainly refers to the processing and manufacturing industry, which uses consumes the basic materials provided by nature and the primary industry for during processing. The tertiary industry refers to those other industries other than the primary and secondary industries, and has a wide range, mainly including nonmaterial-material production sectors such as transportation, communications, commerce, catering, finance, education, and public services.

The results of a new round of PM2.5 source analysis completed by the Beijing Environmental Protection Monitoring Center in 2018 in 2018 were released to the public, and. tThe main conclusions of this study indicate that 2/3 of the main sources of PM2.5 in Beijing throughout the year are local and 1/3 are regional transmissions. From the characteristics of the city ’scity’sthe current local atmospheric PM2.5 sources in Beijing, in terms of the local emissions contribution of local emissions, mobile sources are account for 45%, flying fugitive dust sources account for are 16%, industrial sources account for are 12%, living surface sources account for are 12%, coal sources account for are 3%, and agricultural, natural sourcesand, others sources account for aboutapproximately 12% of the PM2.5 concentration. The results show that at this stage, the coal-fired source has basically withdrawn from the ranks ofstopped being the city's main source of atmospheric PM2.5 in Beijing [58].

TIn the Beijing-Tianjin-Hebei region, takes Tianjin is selected as an example. The analysis results of the air pollution sources in Tianjin in 2014 in 2014 showed [16] that the regional transmission of atmospheric PM2.5 sources accounted for 22%-34% of the pollution. The remaining 66% to 78% came from local emissions. Among them, dust sources accounted for 30%, coal-fired sources accounted for 27%, motorized sources accounted for 20%, industrial production sources accounted for 17%, and other sources accounted for 6%. Dust is the primary source of pollution, followed by coal combustion and motor vehicles. The rRoads, storage yards, construction sites, etc. in Tianjin are the main sources of dust. In addition, construction projects in the railway, chemical, and power industries will also generate dust. In 2014, the three industrial structures in Tianjin were adjusted from 1.6: 53.1: 45.3 in 2010 in 2010 to 1.3: 49.4: 49.3. The secondary industry dropped decreased by 3.7% percentage points, and the tertiary industry increased by 4% percentage points. Until 2014, the proportions of the secondary and tertiary industries in Tianjin was basically flatpractically remained stable. Comparing In comparing the data, it is found that the proportion of the Tianjin's secondary industry in Tianjin is still the highest despite a its decline. Thermal power, iron and steel, petrochemical, cement, building materials, etc., as typical heavy high-pollution emission industries, are the key sources of pollution caused bycausing the air pollution in Tianjin.

The Aanalytical results of the air pollution sources in Hebei Province in the Beijing-Tianjin-Hebei region show reveal that [17], taking with Shijiazhuang city as an example, regional transmission accounts for 23% to 30% of the PM2.5 sourcespollution, and local pollution emissions accounts for 70% to 77%, of which coal combustion accounts for 28.5%, industrial production accounts for 25.2%, fugitive dust accounts for 22.5%, motor vehicles account for 15.0%, and other sources such as biomass burning, and catering, etc. account for 8.8% of the PM2.5 pollution. Coal combustion is the primary source of the air pollutants in Shijiazhuang and even in Hebei Province. Large The high coal consumption and irrational coal combustion structurestructures are the main causes of soot pollution. In 2014, the three industrial structures in Hebei Province were adjusted from 12.57: 52.5: 34.93 in 2010 in 2010 to

11.7: 51.1: 37.2. The primary industry declined decreased slightly, and the secondary industry decreased by 1.4 %percentage points, and while the tertiary industry increased by approximately 2-3 %percentage pointpoints. The proportion of the secondary industry in Hebei Province is still higher than 50%. This irrational industrial structure makes Hebei Province the region with the worst air pollution in the Beijing-Tianjin-Hebei. In addition, Hebei has a large high proportion of high-pollution and high-energy- consuming consumption industries, including steel, cement, electricity, glass, pharmaceuticals, metallurgy, petrochemicals, and building materials. Among them, the coal consumption in the steel, building materials and power industries accounted for 89.6% of the province's provincial total energy consumption, and the air pollutant emissions in from the steel, cement, power, and glass industries accounted for aboutapproximately 60% of the Hebei's total pollution in Hebei. [18]. As the most important pollution industry in Hebei, steel is the focus of air pollution control.

Causes of Air Pollution in Xinjiang

Landform analysis

Xinjiang is far from the sea, surrounded by mountains and basins, and deserts and grasslands are widely distributed. Taking With the topography of Urumqi as an example, its southeast and west sides are surrounded by mountains with an average elevation of 680 metersm. The slope drop gradient is largehigh, with the south is being high, while the north is low, and the middle is are low. The topographical conditions are very highly detrimental to the atmospheric circulation of the atmosphere and pollutant the diffusion of pollutants. The Urumqi region is located in the northern part of the Tianshan Mountains and the southern edge of the Jungle Basin. It is the city farthest from the ocean in the world. It has a typical desert continental climate and a fragile ecological environment. The vegetation coverage around across the urban area is low, and there are many bare groundsareas, which are liable to form dusty weather conditions.

Small Weak winds

The frequent occurrence of calm and small weak winds in Xinjiang (wind speeds greater higher than or equal to 0.5 m/s are and less lower than 1.5 m/s are characterize small weak winds), which is an important factor for the poor air environmental quality factor. In the case of small weak winds, the horizontal movement of the atmosphere is slow, which is not conducive to the lateral diffusion of atmospheric pollutants, and it is easy to form regional pollution accumulation[19regional pollution accumulation easily occurs [19-20]. Wind has a certain diffusion and purification ability, and the wind speed and pollutant concentration are basically negatively correlated. Taking With Urumqi as an example, the frequency of static wind atmospheric conditions in the heating period is as high as aboutapproximately 26%, and in December and January, it is as high as 30% or more. Even if there is wind, the wind speed is very small low and rarely exceeds level 3 [21]. Such meteorological conditions are extremely detrimental to the spread of pollutants dispersal. If there are several consecutive days of static wind weather conditions, the pollutants will continue to accumulate, and the air quality will deteriorate sharply [22].

Sand storms

Sandstorms are a typical weather phenomenon in Xinjiang. Sandstorms and floating dust occur throughout the year. Sandstorms and floating dust weather conditions will cause high concentrations of atmospheric particulate matter concentrations, which will seriously affect all the regionsregions’regional air quality in cities [23]. The source region of western China, with the Taklimakan Desert as the main bodyregion, and the source region of northern China, with the Badain Jaran Desert as the center, contributed 70% of the total dust emissions in Asia. The sSandstorms in Northwest China are dominated by dry settlement [24].Regional The regional distribution of the sandstorms in Northwest China is very uneven, while the sandstorm frequency in the northern region of Xinjiang is basically unchanged, and that in the rest of the region is decreasing [25].

Inversion layer

Among the weather types in Urumqi, there are mostly neutral and stable weather types of weather. The frequency of temperature inversion is as high as 90% in winter. The severe air pollution in winter is directly related to thisthese stable meteorological conditions. The frequently occurring inversion layer that occurs frequently is like is similar to a large pot lid, covering the city, making thereby trapping atmospheric pollutants trapped in theat low altitudealtitudes and making them difficult to spreaddisperse. There is a clear positive correlation between inverse temperature inversion and pollutants pollution [22].

Precipitation

Taking Thethe Urumqi region, as an example, it is located in an arid and semiarid-arid environment with little precipitation and large high evaporation. Precipitation has a purifying effect on the air and can effectively purify urban air.

There is a clear negative correlation between precipitation and pollutantspollution, which is one of the key factors determining the air quality of air. For the SO2, NO2 and PM10 levels in Urumqi., The the daily average concentration and precipitation are analyzed. The analysis results show that the wet removal ability of rain is greater higher than that of snow. rainfallRainfall and snowfall both have wet removal abilityabilities for these three air pollutants [26],, and the strongest highest removal ability is for S02., This is followed by that for PM10.

Dust source

In 2017, with the new area took Turfan City city as as an example of a new area, the emission source of PM10 emission level was 1.169 million tons, and while that of the PM2.5 emissions was 132,000 tons. The source of sSoil dust is the primary source of the particulate emissions in Turpan. Turpan is a sparsely populated area. According to statistics (Turpan Statistical Yearbook (2017)) statistics, the area of land other than urban construction land,and transportation land, water areas and water conservancy facility sites is covers 68,900 square kilometers, with a large area of bare soil. In addition, the Turpan Basin is surrounded by Gobis and deserts. Gobis and deserts account for 76.73% of the area, and there is less vegetation and green land. When windy and sand weather conditions occurs, it is very easy to cause large dust, and frequent dust storms are easily formed. The dust Dust is not only the a source of atmospheric particulate matters, but is also receptors a receptor of particulate matter, resulting in natural dust fall of ground soil dust and sand dust transmission, contributecontributing greatly to the concentration of particulate matter concentration in the atmosphere [27].

Industrial source

Taking With Turpan Ccity in 2017 as an example, in 2017, the PM10 emissions from industrial sources were 19,200 tons, and the PM2.5 emissions were totaled 4,900 tons,the. The main PM10 contribution sources of PM10 are non-metallicthe nonmetallic mineral products industry and power thermal power production, accounting for 81.4% and 15.2%, respectively, of the PM10 pollution;. The PM2.5 contribution of the non-metallicnonmetallic mineral products industry contribution rate is 85.9%, The and the contributions rate of power thermal power production and petroleum processing is are comparable. [27].

Source of life

Taking With Turfan Ccity in 2017 as an example, in 2017, the PM10 emissions of PM10 in thefrom living sourcesourceshuman activities was 94.58 million tons, and the PM2.5 emission amount of PM2.5 was 73.18 million tons. The main sources of the emissions of atmospheric particulate matter PM10 and PM2.5 in the living sourceterms of human activities were was coal combustion and PM 10 and PM 2.5 emissions. Civilian coal combustion technology is poor, and there isare no scientific and effective emission control measures, and while the amount of fine particulate matter PM2.5 emitted by from coal combustion is even higher than that of emitted from industrial activities. During the heating season, civilian coal combustion and central heating caused a large amount of pollutants to be emitted, and a large number amounts of primary aerosols, heavy metals, and water-soluble ions were are directly emitted directly as primary particulate matter. At the same time, gaseous precursors such as SO2 and NO2 were are released. Adverse weather conditions It is easy to form seasily result in severe pollutioneverely polluted weather under adverse weather conditions. The PM2.5 released from food sources is mainly from the cooking process of involving edible oils and foods. Under high- temperature conditions, particulate matter is formed by chemical reactions such as thermal oxidation and thermal decomposition. Most of the food and beverage sources are occur in densely populated areas, and pollutants are emitted near the ground, which has an important notable impact on the health of the population. According to activity- level survey information, there were a total of 3,443 catering companies in Turpan in 2017. The total PM10 emissions were 301.6 tons, and the total PM2.5 emissions were 241.3 tons. In addition, the exhaust gas from restaurant fumes contains a large number of volatile organic pollutantspollutant VOCs, among which oxygenates and olefins have a high photochemical activity,, which are precursors to form secondary particulate matter, and have and have an importanta notable impact on atmospheric particulate pollution [28].

Mobile source

At present, vehicle exhaust is one of the main sources of the atmospheric particulate matter in China [29-30], especially PM2.5 fine particulate matter PM2.5. Motor vehicles also emit gaseous pollutants such as NOx and VOCs, which are converted into secondary particulate matter through photochemical reactions. Compared with to coarse particles, fine particles have a larger specific surface area, which thus capable of absorbsing and carryingies more toxic substances, which is can be harmful to human health [29-32]. The rRoad sources in Turpan in 2017 in 2017 emitted 249.19 tons of PM10 and 224.5 tons of PM2.5, respectively [33].

OffNonroad-road mobile source

Turpan's The nonroad-road mobile sources in Turpan are mainly divided

into three categories: engineering machinery, agricultural machinery and civilian aviation aircraft. Compared with to road mobile sources, nonroad-road mobile sources usually use diesel and heavy oil as their the main fuels. They have the characteristics of a high fuel consumption, long service life, low technical level and low maintenance rate. Most of the pollutants emitted are NOx and particulate matter [34-35].

*Journal Requirements:*

*4. We note that Figures 1-7 in your submission contain satellite images which may be copyrighted. All PLOS content is published under the Creative Commons Attribution License (CC BY 4.0), which means that the manuscript, images, and Supporting Information files will be freely available online, and any third party is permitted to access, download, copy, distribute, and use these materials in any way, even commercially, with proper attribution. For these reasons, we cannot publish previously copyrighted maps or satellite images created using proprietary data, such as Google software (Google Maps, Street View, and Earth). For more information, see our copyright guidelines: http://journals.plos.org/plosone/s/licenses-and-copyright.*

We used resources for replacing copyrighted map figures, USGS National Map Viewer (public domain): http://viewer.nationalmap.gov/viewer/ (page 3, lines 46-48)_._
