# Supplementary material for: The spatiotemporal characteristics of the air pollutants in China from 2015 to 2019
Source: PLoS One. 2020 Aug 21;15(8):e0227469. doi: 10.1371/journal.pone.0227469 (PMC7444542; doi:10.1371/journal.pone.0227469)
Supplement: Attachment — Submitted filename: Response to Reviewers.docx [file pone.0227469.s004.docx]

Dear Zongbo Shi

Academic Editor

PLOS ONE,

Revision required [PONE-D-19-35106R1]

Please find attached a revised version of our manuscript Study on the Spatiotemporal Characteristics of the Air Pollutants in China from 2015 to 2019.

**Your reviews's comments were highly insightful and enabled us to greatly improve the quality of our manuscript.**

We hope that the revisions in the manuscript and our accompanying responses will be sufficient to make our manuscript suitable for publication in the Journal of PLOS ONE.

1. *Re-write the Introduction: Please see more detailed comments in the attached file.*

Yes, We agree with you. We have seen more detailed comments in the attached file and corrected one by one, We have Re-write the Introduction according to rules and made major changes.

2. *Delete the discussions on the causes of air pollution in Jing-Jin-Ji region and Xinjiang - some of these may go to Introduction but it does not fit in the discussions. Some of the discussions in Xinjiang, for example with regards to particular topographic and meteorological conditions may be useful information for discussions.*

Yes, We agree with you. We have deleted the discussions on the causes of air pollution in Jing-Jin-Ji region and Xinjiang.

3. *It is suggested that the discussions focus on the following areas: - Spatial distribution: Read this paper carefully (Drivers of improved PM2.5 air quality in China from 2013 to 2017 : https://www.pnas.org/content/116/49/24463); Your data showed some different patterns in comparison to Figure 2. For example, there are higher pollution in Xinjiang than in Fig. 2 of Zhang et al., 2019; It appears that the pollution level in Sichun Basin is also lower in your data. So one of the things you need to discuss is why ? Please note that Zhang et al. (2019) used chemical transport model, which can be uncertain if the emissions are inaccurate. You need to read how Zhang et al. (2019) evaluated their model results and whether your data showing higher concentrations in Xinjiang is real or biased due to limited number of monitoring stations?*

Thank you for recommending Zhang's article for us. Your recommendation is very helpful to us.

Hong Guo et al. **also found** that the **maximum** annual mean values of both PM2.5 and PM10 were in **Xinjiang** Province, which was determined to be primarily the result of mineral dust from the Taklimakan Desert [18].

Zhang, Y. L. et al. showed that in western China, dust particles are very important for PM2.5, and the current **control strategy** of PM2.5 (that is, reducing VOC and PM emissions from fossil/nonfossil combustion) will **only partially reduce** the PM2.5 pollution in western region [15].

Debin Lu et al. showed that the high PM2.5 concentration in the **northwest** of China was mainly affected **by sand and dust (meteorologically driven)**, while it was mainly caused by **human activities** in the **eastern** region [28].

Shixian Zhai et al. there is also a large **meteorologically driven** interannual variability in PM_2.5_ that complicates trend attribution, increases in the **Fenwei Plain** can be attributed to **meteorology** **rather than** to relaxation of emission controls [63].

Qiang Zhang also cited Shixian Zhai's article, We agree with Shixian Zhai. And We use monitoring data, Qiang Zhang is a simulation. Hong Guo et al. **also found** that the **maximum** annual mean values of both PM2.5 and PM10 were in **Xinjiang** Province.

Qiang Zhang did not comment on Xinjiang, the Weihe River Basin, and the Qinghai-Tibet Plateau which are heavily influenced by meteorological factors. Qiang Zhang commented on the Beijing-Tianjin-Hebei Yangtze River Delta and Pearl River Delta.

Krasnov, H. et al. analyzed the spatiotemporal behavior of PM concentration in the city of Beersheva in southern Israel, an arid desert area where sandstorms are very frequent; and during strong storms, the hourly ranges of PM10 concentration were from 100 μg/m3 to greater than 1280 μg/m3. Higher concentrations were found in parts of the city near the source of dust [22].

Zou, X. K. et al. found that sand and dust storms during the spring in northwestern China, where deserts, semideserts, and grasslands are distributed, were negatively correlated with NDVI [23].

All of the above findings are consistent with our results showing high concentrations of PM2.5 and PM10 in Xinjiang.

We directly used the monitoring data to count the Sichuan Basin and got consistent results with you.

4. *Temporal change: You may try to look at the different regions (BTH and surrounding regions; Xinjiang Basin; Sichun Basin; Weihe Plateau; Northeastern China; South China) and evaluate the absolute and relative changes in concentrations of different pollutants from 2014-2019. This may then be linked to economic growth and clean air actions. You will need to refer to lots of papers. Again you may benefit from comparing with Zhang et al. (2019) paper.*

We agree with you, thank you for your help.

We divide China into 11 regions. The Northern region includes Beijing, Tianjin and Hebei, Shandong, Shanxi, and Henan; the Eastern (Yangtze River Delta) region includes Jiangsu, Zhejiang, and Fujian; the Central region includes Anhui, Hubei, Hunan, and Jiangxi; the Southern (Pearl River Delta) region includes Guangdong, Guangxi, and Hainan. The Weihe River Basin includes Shan Xi, Gansu, and Ningxia; Xinjiang is its own region; the Sichuan Basins is a region; Inner Mongolia is a region; Qinghai-Tibet is a region; Yungui is a region; and the Northeast region includes Heilongjiang, Jilin, and Liaoning.

And we evaluate the absolute and relative changes in concentrations of different pollutants from 2015-2019. Then belinked to economic growth and clean air actions. We refer to lots of papers and compared with Zhang et al. (2019) paper.

5. *Please read the comments by reviewers again and address them - For example, there are a lot of air quality trend papers published recently, which you should read carefully and compare your results with what was published.*

We agree with you, thank you for your help.

We readed a lot of air quality trend papers published recently, and carefully compare your results with what was published.

6. Please note that you need to re-write your abstract as well. Please refer to this website: https://journals.plos.org/plosone/s/submission-guidelines

We agree with you, thank you for your help. We rewrited the abstract and made major changes

**References**

1. State Council of the People’s Republic of China, Notice of the general office of the state council on issuing the air pollution prevention and control action plan. <http://www.gov.cn/zwgk/2013-09/12/content_2486773.htm.>
2. Q. Zhang, Yixuan Zheng, Dan Tong, Min Shao, Shuxiao Wang, Yuanhang Zhang et al. Drivers of improved PM_2.5_ air quality in China from 2013 to 2017. PNAS 2019; 116 (49): 24463-24469.
3. Siyi. Cai et al. The impact of the “Air Pollution Prevention and Control Action Plan” on PM_2.5_ concentrations in Jing-Jin-Ji region during 2012–2020. Sci. Total Environ. 2017; 580: 197-209.
4. X. Jiang Chaopeng Hong, Yixuan Zheng, Bo Zheng, Dabo Guan, Andy Gouldson et al., To what extent can China’s near-term air pollution control policy protect air quality and human health? A case study of the Pearl River Delta region. Environ. Res. Lett. 2015; 10: 104006.
5. Y. Zheng Tao Xue, Qiang Zhang, Guannan Geng, Dan Tong, Xin Li et al. Air quality improvements and health benefits from China’s clean air action since 2013. Environ. Res. Lett 2017; 12: 114020.
6. T. Xue et al. Rapid improvement of PM_2.5_ pollution and associated health benefits in China during 2013–2017. Sci. China Earth Sci 2019; 62: 1847–1856.
7. Peng J, Chen S, Lü H, Liu Y and Wu J. Spatiotemporal patterns of remotely sensed PM_2.5_ concentration in China from 1999 to 2011. Remote Sens. Environ. 2016; 174: 109–21.
8. Ma Z, Hu X, Sayer A M, Levy R, Zhang Q, Xue Y, Tong S, Bi J, Huang L and Liu Y Satellite-based spatiotemporal trends in PM_2.5_ concentrations: China, 2004-2013 Environ. Health Perspect. 2016a; 124: 184–92.
9. Krotkov N A et al. Aura OMI observations of regional SO_2_ and NO_2_ pollution changes from 2005 to 2015 Atmos. Chem. Phys 2016; 16: 4605–29.
10. Verstraeten W W, Neu J L, Williams J E, Bowman K W, Worden J R and Boersma K F Rapid increases in tropospheric ozone production and export from China. Nat. Geosci 2015; 8 690–5.
11. Zhou, Y. Shuiyuan Cheng, Dongsheng Chen, Jianlei Lang, GangWang, Tingting Xu et al. Temporal and Spatial Characteristics of Ambient Air Quality in Beijing, China. Aerosol and Air Quality Research 2015; 15: 1868–1880.
12. Guo, H. et al. Assessment of PM_2.5_ concentrations and exposure throughout China using ground observations. The Science of the total environment 2017; 601-602: 1024-1030.
13. Chen, T., He, J., Lu, X., She, J. & Guan, Z. Spatial and Temporal Variations of PM_2.5_ and Its Relation to Meteorological Factors in the Urban Area of Nanjing, China. International journal of environmental research and public health 2016; 13.
14. Wang, W. N. et al. Assessing Spatial and Temporal Patterns of Observed Ground-level Ozone in China. Scientific reports 2017; 7: 3651.
15. Zhang, Y. L. & Cao, F. Fine particulate matter (PM_2.5_) in China at a city level. Scientific reports 2015; 5, 14884.
16. Cheng, N. et al. Ground-Level NO2 in Urban Beijing: Trends, Distribution, and Effects of Emission Reduction Measures. Aerosol and Air Quality Research 2018; 18, 343-356.
17. B Silver1, C L Reddington, S R Arnold and D V Spracklen. Substantial changes in air pollution across China during 2015-2017. Environmental Research Letters 2018; 13, 11.
18. Hong Guo, Xingfa Gu, Guoxia Ma, Shuaiyi Shi, Wannan Wang, Xin Zuo & Xiaochuan Zhang. Spatial and temporal variations of air quality and six air pollutants in China during 2015–2017. Scientific Reports volume 2019; 9: 15201.
19. Rohde R A and Muller R A. Air pollution in China: mapping of concentrations and sources PLoS ONE 2015; 10: e0135749.
20. Liang X, Li S, Zhang S, Huang H and Chen S X. PM_2.5_ data reliability, consistency, and air quality assessment in five Chinese cities. J. Geophys. Res. 2015; 121: 10.
21. Leung D M, Tai A P K, Mickley L J, Moch J M, Van Donkelaar A, Shen L and Martin R V. Synoptic meteorological modes of variability for fine particulate matter (PM_2.5_) air quality in major metropolitan regions of China Atmos. Chem. Phys 2018; 18 6733-48.
22. Krasnov, H., Kloog, I., Friger, M. & Katra, I. The Spatio-Temporal Distribution of Particulate Matter during Natural Dust Episodes at an Urban Scale. PloS one 2016; 11: e0160800.
23. Zou, X. K. & Zhai, P. M. Relationship between vegetation coverage and spring dust storms over northern China. J. Geophys. Res. 2004; 109: D03104.
24. Qingyu Guan, Ao Cai, Feifei Wang, Liqin Yang, Chuangqi Xu, Zeyu Liu. Spatio-temporal variability of particulate matter in the key part of Gansu Province, Western China. Environmental Pollution 2017; Volume 230: Pages 189-198.
25. Pengfei Chen, Shichang Kang, Junhua Yang, Tao Pu, Chaoliu Li, Junming Guo, Lekhendra Tripathee. Spatial and Temporal Variations of Gaseous and Particulate Pollutants in Six Sites in Tibet, China, during 2016-2017. Aerosol and Air Quality Research 2019; 19: 516–527.
26. Ministry of Ecology and Environment of the People’s Republic of China, 2017 Report on the state of the ecology and environment in China. http://english.mee.gov.cn/Resources/Reports/soe/SOEE2017/201808/P020180801597738742758.pdf. Accessed 28 October 2019.
27. Wang, Y., Ying, Q. Hu, J. & Zhang, H. Spatial and temporal variations of six criteria air pollutants in 31 provincial capital cities in China during 2013-2014. Environment international 2015; 73: 413–422.
28. Debin Lu, Jianhua Xu, Dongyang Yang, Jianan Zhao. Spatio-temporal variation and influence factors of PM_2.5_ concentrations in China from 1998 to 2014. Atmospheric Pollution Research 2017; Volume 8, Issue 6: Pages 1151-1159.
29. Janssen L, Buringh E, Van Der Meulen A, Van Den Hout K. A method to estimate the distribution of various fractions of PM_10_ in ambient air in the Netherlands. Atmos Environ 1999; 33 (20): 3325–3334.
30. Dongsheng Zhan, Mei-Po Kwan, Wenzhong Zhang, Xiaofen Yu, Bin Meng, Qianqian Liu. The driving factors of air quality index in China. Journal of Cleaner Production 2018; Volume 197, Part 1: Pages 1342-1351.
31. Chuangin Fang, Haimeng Liu, Guangdong Li, Dongqi Sun, Zhuang Miao. Estimating the Impact of Urbanization on Air Quality in China Using Spatial Regression Models. Sustainability 2015; 7(11): 15570-15592.
32. Y. Q. Wang. The contribution from distant dust sources to the atmospheric particulate matter loadings at XiAn, China during spring. Science of The Total Environment 2006; Volume 368, Issues 2-3: 875-883.
33. Xiaofeng Zhao, Chunlei Deng, Xianjin Huang, Mei-Po Kwan. Driving forces and the spatial patterns of industrial sulfur dioxide discharge in China 2017; Volume 577: Pages 279-288.
34. Caiwang Zhang, Chuanfeng Zhao, Yanping Li, Xiaolin Wu, Kaiyang Zhang, Jing Gao, Qi Qiao, Yuanzhe Ren, Xin Zhang, Fahe Chai. Spatial and temporal distribution of NO2 and SO2 in Inner Mongolia urban agglomeration obtained from satellite remote sensing and ground observations. Atmospheric Environment 2018; Volume 188: Pages 50-59.
35. Jinwei Huo, Degang Yang, Wenbiao Zhang, Fei Wang, Guiling Wang, Qain Fu. Analysis of influencing factors of CO_2_ emissions in Xinjiang under the context of different policies. Environmental Science & Policy 2015; Volume 45: Pages 20-29.
36. Yuanzheng Cui, Jintai Lin, Chunqiao Song, Mengyao Liu, Yingying Yan, Yuan Xu, and Bo Huang. Rapid growth in nitrogen dioxide pollution over Western China, 2005–2013. Atmos. Chem. Phys 2016; 16: 6207–6221.
37. Krotkov N A et al. Aura OMI observations of regional SO_2_ and NO_2_ pollution changes from 2005 to 2015 Atmos. Chem. Phys 2016; 16: 4605–29.
38. B. Zhao et al., A modeling study of the nonlinear response of fine particles to air pollutant emissions in the Beijing–Tianjin–Hebei region. Atmos. Chem. Phys 2017; 17: 12031–12050.
39. L. Ran, W. L. Lin, Y. Z. Deji, B. La, P. M. Tering, X. B. Xu, and W, Wang. Surface gas pollutants in Lhasa, a highland city of Tibet – current levels and pollution implications. Atmos 2014; Chem. Phys., 14: 10721–10730 .
40. Zheng Y, Xue T, Zhang Q, Geng G, Tong D, Li X and He K. Air quality improvements and health benefits from China’s clean air action since 2013. Environ. Res. Lett 2017; 12.
41. Lin C. Q, Liu G, Lau A K H, Li Y, Li C C, Fung J C H and Lao X Q. High-resolution satellite remote sensing of provincial PM2.5 trends in China from 2001 to 2015 Atmos. Environ 2018; 180: 110–6.
42. The Statistical Communique of the People’s Republic of China (SCPRC). Available at http://data.stats.gov.cn/easyquery.htm?cn=C01&zb=A0C05&sj=2015.
43. The Statistical Communique of the People’s Republic of China (SCPRC). Available at http://data.stats.gov.cn/easyquery.htm?cn=C01&zb=A0C05&sj=2016.
44. The Statistical Communique of the People’s Republic of China (SCPRC). Available at <http://data.stats.gov.cn/easyquery.htm?cn=C01&zb=A0C05&sj=2017>.
45. Xiufeng Yin, Benjamin de Foy, Kunpeng Wu, Chuan Feng, Shichang Kang, Qianggong Zhang. Gaseous and particulate pollutants in Lhasa, Tibet during 2013–2017: Spatial variability, temporal variations and implications. Environmental Pollution 2019; 253: Pages 68-77.
46. Xionghui Qiu, Lei Duan, Siyi Cai, Qian Yu, Shuxiao Wang, Fahe Chai, Jian Gao, Yanping Li, Zhaoming Xu. Effect of current emission abatement strategies on air quality improvement in China: A case study of Baotou, a typical industrial city in Inner Mongolia. Journal of Environmental Sciences 2017; Volume 57: Pages 383-390.
47. B.Zheng, Dan Tong, Meng Li, Fei Liu, Chaopeng Hong, Guannan Geng, Haiyan Li et al., Trends in China’s anthropogenic emissions since 2010 as the consequence of clean air actions. Atmos. Chem. Phys 2018; 18: 14095–14111.
48. Jing Huang, Xiaochuan Pan, Xinbiao Guo, Guoxing, Li. Health impact of China's Air Pollution Prevention and Control Action Plan: an analysis of national air quality monitoring and mortality data. The Lancet Planetary Health 2018; Volume 2, Issue 7: Pages e 313 - e 323
49. D.G Streets. S. T Waldhoff. Present and future emissions of air pollutants in China: SO_2_, NOx, and CO. Atmospheric Environment 2000; Volume 34, Issue 3: Pages 363-374.
50. Liu F, Zhang Q, Van Der A R J, Zheng B, Tong D, Yan L, Zheng Y and He K. Recent reduction in NOx emissions over China: synthesis of satellite observations and emission inventories Environ. Res. Lett. 2016; 11: 114002.
51. Van Der A R J, Mijling B, Ding J, Elissavet Koukouli M, Liu F, Li Q, Mao H and Theys N. Cleaning up the air: effectiveness of air quality policy for SO2 and NOx emissions in China Atmos. Chem. Phys 2017; 17: 1775–89.
52. Ke. Li et al. Anthropogenic drivers of 2013–2017 trends in summer surface ozone in China. Proc. Natl. Acad. Sci. U.S.A 2019; 116: 422–427.
53. Jin X and Holloway T. Spatial and temporal variability of ozone sensitivity over China observed from the Ozone Monitoring Instrument J. Geophys. Res 2015; 120 7229–46.
54. Chang K L, Petropavlovskikh I, Copper O R, Schultz M G and Wang T. Regional trend analysis of surface ozone observations from monitoring networks in eastern North America, Europe and East Asia Elem. Sci 2017; 5: 50.
55. Fleming Z L et al. Tropospheric ozone assessment report: present-day ozone distribution and trends relevant to human health Elem. Sci. Anth 2018; 6: 12.
56. S. Zhai et al., Fine particulate matter (PM_2.5_) trends in China, 2013–2018: Separating contributions from anthropogenic emissions and meteorology. Atmos. Chem. Phys 2019; 19: 11031–11041.
57. D. Ding, J. Xing, S. Wang, K. Liu, J. Hao, Estimated contributions of emissions controls, meteorological factors, population growth, and changes in baseline mortality to reductions in ambient PM2.5 and PM2.5-related mortality in China, 2013–2017. Environ. Health Perspect 2019; 127.
58. ML Bell, DL Davis, T Fletcher. A retrospective assessment of mortality from the London smog episode of 1952: the role of influenza and pollution.Environ Health Perspect 2004; 112: 6-8.
59. DL Davis, ML Bell, T Fletcher. A look back at the London smog of 1952 and the half century since. Environ Health Perspect 2002; 110: A734-A735.
60. CA Pope 3rd, M Ezzati, DW Dockery. Fine-particulate air pollution and life expectancy in the United States N Engl J Med 2009; 360: 376-386.
61. EE Dooley. Fifty years later: clearing the air over the London smog. Environ Health Perspect 2002; 110: A748.
62. US Environmental Protection Agency Benefits and costs of the Clean Air Act 1990–2020, the second prospective study. https://www.epa.gov/clean-air-act-overview/benefits-and-costs-clean-air-act-1990-2020-second-prospective-study.
63. S. Zhai et al., Fine particulate matter (PM2.5) trends in China, 2013–2018: Separating contributions from anthropogenic emissions and meteorology. Atmos. Chem. Phys. 19, 11031–11041 (2019).
