# Supplementary material for: The spatiotemporal characteristics of the air pollutants in China from 2015 to 2019
Source: PLoS One. 2020 Aug 21;15(8):e0227469. doi: 10.1371/journal.pone.0227469 (PMC7444542; doi:10.1371/journal.pone.0227469)
Supplement: Attachment — Submitted filename: Response to Reviewers.docx [file pone.0227469.s005.docx]

Dear Zongbo Shi

Academic Editor

PLOS ONE,

Minor revision required [PONE-D-19-35106R3]

Please find attached a revised version of our manuscript The spatiotemporal characteristics of the air pollutants in China from 2015 to 2019.

**Your reviews's comments were highly insightful and enabled us to greatly improve the quality of our manuscript again.**

*Abstract: remove reference (i.e. [1])
particulate matter PM2.5 – delete particulate matter
particulate matter PM10 – delete particulate matter
Delete: “Since 2015, environmental monitoring 15 stations in 366 cities have regularly released air quality data. The volume of data 16 released since 2015 far exceeds those available in 2014 (189 cities) and 2013 (74 17 cities), and this increased number of environmental monitoring stations can better 18 reflect China's atmospheric environment.”*

**We agree with you, we have deleted them.**

*“Therefore, we propose that it is meaningful to” change to “here, we”*

**We agree with you, we have changed it.**

*In Materials and Methods, please provide a detailed explanation how the data analyses were carried out.*

**We agree with you, we have provided a detailed explanation how the data analyses were carried out in lines 80-125.**

*“Xinjiang is its own region; the Sichuan Basins is a region; Inner Mongolia is a region; Qinghai-Tibet is a region; Yungui is a region; and the Northeast region includes Heilongjiang, Jilin, and Liaoning” change to “Other regions include Xijiang, Sichun Basin, Inner Mogolia, Qinghai-Tibet, Yungui (Yunan and Guichzou) and Northeastern China (Heilongjiang, Jilin, and Liaoning)”*

**We agree with you, we have changed them in lines 135-137.**

*Line 147: thee – change to the*

**We agree with you, we have changed it.**

*“Qingyu Guan et al. the PM10 concentrations during dust storm events were, respectively, 19, 43 and 17 times higher than the levels before dust events in cities of 167 Weihe River Basin [24] “ – why this is relevant?*

**We agree with you, we have deleted it.**

*Proper citation needed: Hong Guo et al. – this should be Guo et al.; comments apply to the rest of the paper; Q. Zhang et al. also is wrong – use Zhang et al.; Janssen L.'s – change to Janssen et al. (20??)*

**We agree with you, we have changed them.**

*Line 137: I suggest you to look again at Zhang et al. (2019) paper; if I am not wrong, there are some discrepancies with your figure and if so you really need to highlight the differences. #*

**We agree with you. We think that Zhang et al. are right. Because we have fewer monitoring points in Xinjiang in this research, we have highlighted the differences and clarified the limitations of our research in lines 181-189.**

**Although our results are consistent with Guo et al. [18] and Hao et al. [19], who reported PM_2.5_ is most serious in the Xinjiang Province. But we think our research results have certain limitations, this is because fewer monitoring points were used in Xinjiang in the previous studies(we, Guo et al. and Fan et al.); thus, our research results have certain limitations. We have clarified the limitations of our research results in lines 181-189 and removed the objectionable figures.**

**[18] Hong Guo, Xingfa Gu, Guoxia Ma, Shuaiyi Shi, Wannan Wang, Xin Zuo & Xiaochuan Zhang. Spatial and temporal variations of air quality and six air pollutants in China during 2015-2017. Scientific Reports 2019; 9, 15201; https://www.nature.com/articles/s41598-019-50655-6.**

**[19] Hao Fan, Chuanfeng Zhao, Yikun Yang. A comprehensive analysis of the spatio-temporal variation of urban air pollution in China during 2014–2018. Atmospheric Environment 2020; 220, 117066; <https://doi.org/10.1016/j.atmosenv.2019.117066.>**

*Line 173 – 177: It would be interesting to see the trend of PM2.5/PM10 ratio from 2015 to 2019; is there a trend? But in any case, report the annual average values*

**We agree with you, we have deleted it.**

*Use proper punctuation: in many places you use “;” at the end of the sentences. In some places, you started a sentence with lower case: for example, line 189, you use “thus” rather than “Thus”.*

**We agree with you, we have changed them.**

*There are many grammatical errors including some of those pointed out above. This has to be corrected and if this continues, I will have to recommend “rejection” eventually.*

**Thank you very much for your help and understanding. We invited Premium Editing of American Journal Experts (AJE) to polish the article. We carefully checked the grammatical errors in the article. Now there is no grammatical errors in the article.**
*In several places, a single paragraph described the findings of a particular paper; this causes fragmentation. Please revise this and put these paragraphs together to deliver a meaningful message. If you are suggesting that these results are similar to what you have showed, you can say that: Our results are consistent with literature. For example, xxx et al. (2019) showed that ….; xxx et al. (2018) showed that ….
Similarly, in line 276-281: you cited two papers but did not say why these are cited? Are you trying to say that these results support your conclusion? If so, you need to say something like: these results are consistent with the trend we found (Fig. xxx).*

**We agree with you, we have said something like: our results are consistent with literature. For example, xxx et al. showed that ….; xxx et al. showed that …, these results are consistent with the trend we found.**

*The discussions from line 706 are very confusing. It is difficult to find the logic here. A majority of this is irrelevant so should be removed or distributed elsewhere. For example, for those discussion on PM2.5 – this should go to the part on PM2.5 in “change over time”; Similarly, discussions on SO2 etc. goes to separate sections above.*

**We agree with you, we have removed majority from line 706.**

*Remove all of these irrelevant discussions on “clean air acts” in US and UK.*

**We agree with you, we have removed all of these irrelevant discussions on “clean air acts” in US and UK.**

*Line 716-724: delete; or you can have a look at Zhang et al. (2019) paper to see and Vu et al. (2019, Atmos Chem Phys) paper to see the effects of meteorology. In the longer term, meteorology is not so important, and the emission change is the dominant contributor to air quality changes. This discussion could be useful*

**We agree with you, we have deleted Line 716-724. Thank you for your recommendation and help. Papers of Zhang et al. (2019), Vu et al. (2019, Atmos Chem Phys) had a great help for us.**

*5. Is the manuscript presented in an intelligible fashion and written in standard English?*

*PLOS ONE does not copyedit accepted manuscripts, so the language in submitted articles must be clear, correct, and unambiguous. Any typographical or grammatical errors should be corrected at revision, so please note any specific errors here.*

*Reviewer #2: No*

**We agree with you. We invited Premium Editing of American Journal Experts (AJE) to polish the article. We carefully checked the grammatical errors in the article.**

*Reviewer #2: This paper is highly improved in this version. The structure of the paper is clear now and more discussion was added in. The data in this paper covers hundreds of cities in China, it provides a whole picture of the evolution of air quality in China in last 6 years. From this standpoint the article is useful and can be used to document levels at present day. Several improvements are needed to be addressed before the manuscript can be considered for publication after minor revision.*

1) *I would suggest the author give the (possible) reason for the variation of each pollutant. This would help the government to improve the air quality in right direction.*

**We agree with you. We have given the (possible) reasons for the variations of each pollutant in lines 688-708.**

**The possible reasons for the changes in PM_2.5_, SO_2_, NO_2_, and CO are related to the strengthening of industrial emission standards. For example, the emission standards of thermal power plants and all emission-intensive industrial sectors (such as steel and cement) have been strengthened. By the end of 2017, more than 95% of China's coal-fired power plants were equipped with flue gas desulfurization (FGD) and selective catalytic reduction (SCR) or selective non-catalytic reduction (SNCR) systems and 71% of the coal-fired power generation capacity reached the "ultra-low emission" standard. In addition, industrial boiler were upgraded and small coal-fired boilers were eliminated, which was important because large-scale operation boilers are widely equipped with SO_2_ and particulate matter control devices. The elimination of backward industries phases out obsolete or inefficient technology in various industries and allows for structural adjustments. In addition, clean fuel was promoted in the residential sector and advanced stoves and clean coal nationwide were promoted from 2013 to 2016. In 2017, the use of natural gas and electricity to replace coal was further promoted, which affected 6 million households nationwide, of which 4.8 million were located in the Beijing-Tianjin-Hebei area and surrounding areas. The benefits of promoting clean fuels in the residential sector are also obvious throughout the country, and the transportation sector elevated emission standards and imposed mandatory elimination of old vehicles that do not meet emission standards [2].**

**Such changes would help the government improve the air quality.**

2) *Line 96-97 on page 5, please rephrase the sentence of “From 2015 to 2019, the national annual mean and annual average median of PM2.5 (unit: μg/m3) were 42.7 and 41, respectively.” I think it should be “The national annual mean and annual average median of PM2.5 (unit: μg/m3) were 42.7 and 41 in 2015 and 2019, respectively.”*

**I am sorry. We Calculated mean and median. So we said, from 2015 to 2019, the national annual mean and annual average median of PM_2.5_ (unit: μg/m^3^) were 42.7 and 41.0, respectively. AJE also checked here again.**

3) *Line 836 on page 42. The first author should be “B Silver”.*

**We agree with you, we have changed it.**

**Dear Zongbo Shi**

**Academic Editor**

**PLOS ONE**

**Thank you for your help and understanding during these times.**

**Your reviews's comments were highly insightful and enabled us to greatly improve the quality of our manuscript again.**
