# Supplementary material for: The spatiotemporal characteristics of the air pollutants in China from 2015 to 2019
Source: PLoS One. 2020 Aug 21;15(8):e0227469. doi: 10.1371/journal.pone.0227469 (PMC7444542; doi:10.1371/journal.pone.0227469)
Supplement: Attachment — Submitted filename: Response to Reviewers.docx [file pone.0227469.s006.docx]

Dear Zongbo Shi

Academic Editor

PLOS ONE,

Minor revision required [PONE-D-19-35106R4]

Please find attached a revised version of our manuscript The spatiotemporal characteristics of the air pollutants in China from 2015 to 2019.

**Your reviews's comments were highly insightful and enabled us to greatly improve the quality of our manuscript again.**

*Line 11: “simulated data “ – change to “model simulations”*

**We agree with you, we have changed it.**

*Line 14: “various” – change to “different”*

**We agree with you, we have changed it.**

*Line 16: add “This led to a major improvement in air quality”*

**We agree with you, we have added it.**

*Line 16 – 17: change to “We use the hourly Air Quality Index (AQI) and mass concentrations of PM10, CO, NO2, O3, and SO2 in 362 cities from 2015 to 2019”*

**We agree with you, we have changed it.**

*Line 35: “through the simulation methods” changed to “using models”*

**We agree with you, we have changed it.**

*Line 42: “34% SO2, 28% NOx, 26% PM2.5, and 10% volatile organic compounds” change to “SO2 by 34%, NOx by 28%, PM2.5 by 26%, and volatile organic compounds by 10%”*

**We agree with you, we have changed it.**

*Line 45: “inversion of” change to “retrieved”*

**We agree with you, we have changed it.**

*Line 54: “upper ozone monitor (OMI)” should this be “The Ozone Monitoring Instrument (OMI)”?*

**We agree with you, we have changed it.**

*Line 76: delete “In addition, we also found that NO2 has improved as of 2019”*

**We agree with you, we have deleted it.**

*Line 77: delete “propose that it is meaningful to”*

**We agree with you, we have deleted it.**

*Line 87: delete “scientific”*

**We agree with you, we have deleted it.**

*Line 137: add “concentration” after Annual mean*

**We agree with you, we have added it.**

*Throughout the manuscript, please add unit to the reported concentration (but no AQI). For example “From a regional perspective, the Northern (58.2), Xinjiang (52.5), and Central (46.6)” should be changed to “From a regional perspective, the Northern (58.2 µg m-3), Xinjiang (52.5 µg m-3), and Central 144 (46.6 µg m-3)”*

*Line 156: “From 2015 to 2019, the national annual mean PM10 (unit: μg/m3 156 ) was 78.6.” This is incorrect; this has to be changed to “From 2015 to 2019, the national annual mean PM10 was 78.6 µg m-3.”*

**We agree with you, we have added unit.**

*Line 160: “with highest annual mean.” Change to “with highest annual mean concentration.”*

**We agree with you, we have changed it.**

*Line 171-172: “Hebei Province, Henan Province and Shandong Province” change to “Hebei, Henan and Shandong Provinces”*

**We agree with you, we have changed it.**

*Line 178: “Our results showing that higher annual mean PM2.5 concentrations occurred in” : This does not look right; you said earlier PM2.5 is highest in Northern China; now you said that Xinjiang is highest; I think you mean “Our results showing that higher annual mean PM10 concentrations occurred in Xinjiang”*

**We agree with you, we have changed it.**

*Line 184-186: “considerably different from Zhang et al. because fewer monitoring points were used in Xinjiang in this study, thus, our research results have certain limitations.” Change to “somewhat different from Zhang et al.. This may be attributed to model uncertainty or the limited number of monitoring stations available in Xinjiang in this study.”*

**We agree with you, we have changed it.**

*Line 187-199: suggest to delete this paragraph*

**We agree with you, we have deleted this paragraph.**

*Line 201: “Sanya (14.6), has an annual mean PM2.5 (unit: μg/m3 )” : do remember to revise this: “Sanya, has an annual mean PM2.5 of 14 μg m-3 )”*

**Yes, we have revised this.**

*Line 204: delete “The major cities”*

**We agree with you, we have deleted it.**

*Line 275: “Our results are consistent with the literature.” You have said this many times. Delete line 275 – 282: This is about CO2 and it has nothing to do with CO*

**We agree with you, we have deleted it.**

*Line 291: “that” change to “the”*

**We agree with you, we have changed it.**

*Line 299-303: “The fast developing resource and pollution intensive industries along with the ‘Go West’ movement and weak emission controls [30] contributed to the higher rate of increase in NO2 over the Western region from 2005-2013 than over that over the Southwestern, Northern, Eastern, and Southern regions”*

**We agree with you, we have changed it.**

*Line 312: “We found that NO2 has the opposite trend in cities in the same region.” Change to “We found that NO2 shows a different trend in cities in the same region.”*

**We agree with you, we have changed it.**

*Line 316: delete “so neighboring areas may exhibit the opposite trend (for example, Hong Kong and the 318 Pearl River Delta)”*

**We agree with you, we have deleted it.**

*Line 324: “were the regions with the highest annual mean (average median) across the country” change to “had a higher annual mean (median) concentration ”*

**We agree with you, we have changed it.**

*Line 329-333: delete this paragraph. Add a sentence to the end of the last paragraph: “High O3 concentrations in the city of Lhasa of Tibet may be associated with stronger photochemical reactions, vertical mixing and downward transport of stratospheric air mass”*

**We agree with you, we have deleted it and added the sentence to the end of the last paragraph.**

*Line 352: “(Table 2-5), (S1-9 Table), (Fig 1-2)” change to “(Table 2-5, Table S1-9; Fig 1-2)”*

**We agree with you, we have changed them.**

*Line 356: “Our results are consistent with the literature. For example, Guo et al. reported that the number of cities where PM2.5 meets the air quality guidelines of WHO (annual mean 0-10 μg/m3) increased from 0 in 2015 to 1 in 2017, the number of cities where PM2.5 reached target 1 of the transition period of WHO (annual mean 25-35 μg/m3 360 ) increased from 57 in 2015 to 77 in 2017, and the annual mean of 309 cities decreased in 2017 compared with 2015 [18].” Change to “In comparison with 2017 [18], there are more cities which meets the WHO guidelines and the WHO target 1 transition period (annual mean 25-35 μg/m3)”*

**We agree with you, we have changed it.**

*Line 361: “In addition,” change to: “our results are comparable with literature. For example, “*

**We agree with you, we have changed it.**

*Line 372: “main reason” change to “key factor”*

**We agree with you, we have changed it.**

*Line 376: delete “across the country”*

**We agree with you, we have deleted it.**

*Line 377: “value”: what does this mean? Is it “average change”?*

**Yes, it is “average change”, we have changed it.**

*Line 378: “The Ali area (-20%) of the Qinghai-Tibet; Hegang (-33.9%) and 379 Baicheng (-18.8%) in the Northeast region; and Haimen (-14.4%) and Jinhua (-12.7%) 380 in the Eastern region are the cities with a faster decline.” Change to “Ali (-20%) of the Qinghai-Tibet, Hegang (-33.9%) and Baicheng (-18.8%) in the Northeast region, and Haimen (-14.4%) and Jinhua (-12.7%) in the Eastern region are the cities with a faster decline.”*

**We agree with you, we have changed it.**

*Line 381: delete “Our results are consistent with the literature. For example,”*

**We agree with you, we have deleted it.**

*Line 393: “but also from the low emission sources (such as residential coal combustion)” change to “as well as residential sources”*

**We agree with you, we have changed it.**

*Line 405: “almost the” change to “second highest”*

**We agree with you, we have changed it.**

*Line 407: “Our results are consistent with the literature. For example,” change to “This is at least partially due to the natural PM sources, such as dust.”: merge this paragraph with the previous one*

**We agree with you, we have changed it and merged this paragraph with the previous one.**

*Line 418-491: “The above findings relate to the relative change of the annual mean of PM2.5 in 419 the five years from 2015 to 2019.” This sentence makes no sense. What “above findings”?*

**We agree with you, we have deleted them.**

*Line 412: delete “Our results 422 are consistent with the literature. For example,”*

**We agree with you, we have deleted it.**

*Line 442: correct the references to figures / tables*

**We agree with you, we have corrected it.**

*Line 443: change “0-20” to “20”*

**We agree with you, we have changed it.**

*Line 448: PLEASE STOP USING “Our results are consistent with the literature. For example,”: You did this for 18 times. This is how a discussion works. Please discuss the results similarly as suggested earlier for PM2.5. Using literature to help you to explain the results, not to say that yours are consistent with literature.*

**We agree with you, we have stoped using this sentence.**

*Line 459-461: Make a similar change as suggested earlier for PM2.5*

**We agree with you, we have changed it.**

*Line 465: “almost lowest” change to “lower”*

**We agree with you, we have changed it.**

*Line 675: “Possible reasons for the variations of each pollutant” change to “Factors contributing to the decrease in air pollutant levels”*

**We agree with you, we have changed it.**

*Line 676: “The possible reasons for the changes in PM2.5, SO2, NO2, and CO are related to the strengthening of industrial emission standards. For example,” Change to “A number of policy actions contributed to the decrease in PM2.5, SO2, NO2, and CO levels. Firstly,”*

**We agree with you, we have changed it.**

*Line 695: delete*

**We agree with you, we have deleted it.**

*Line 700: “the whole of China is dominated by PM2.5 fine particles, and the spatial distribution shows a concentrated distribution (concentrated in the Northern region, Xinjiang, the Central region, the Weihe River Basin, etc.)” change to “PM2.5 is the most important air pollutant in most regions in China, particularly in the Northern China, Xinjiang, Central China, and the Weihe River Basin)”*

**We agree with you, we have changed it.**

**Dear Zongbo Shi**

**Academic Editor**

**PLOS ONE**

**Thank you for your help and understanding during these times.**

**Your reviews's comments were highly insightful and enabled us to greatly improve the quality of our manuscript again.**
